# Control Strategies for the DAB Based PV Interface System

**DOI:** 10.1371/journal.pone.0161856

**Published:** 2016-08-25

**Authors:** Hadi M. El-Helw, Mohamed Al-Hasheem, Mostafa I. Marei

**Affiliations:** 1 Electrical and Control Department, College of Engineering & Technology , Arab Academy for Science, Technology & Maritime Transport, Cairo, Egypt; 2 Electrical Power and Machines Department, Faculty of Engineering, Ain shams University, Cairo, Egypt; Chongqing University, CHINA

## Abstract

This paper presents an interface system based on the Dual Active Bridge (DAB) converter for Photovoltaic (PV) arrays. Two control strategies are proposed for the DAB converter to harvest the maximum power from the PV array. The first strategy is based on a simple PI controller to regulate the terminal PV voltage through the phase shift angle of the DAB converter. The Perturb and Observe (P&O) Maximum Power Point Tracking (MPPT) technique is utilized to set the reference of the PV terminal voltage. The second strategy presented in this paper employs the Artificial Neural Network (ANN) to directly set the phase shift angle of the DAB converter that results in harvesting maximum power. This feed-forward strategy overcomes the stability issues of the feedback strategy. The proposed PV interface systems are modeled and simulated using MATLAB/SIMULINK and the EMTDC/PSCAD software packages. The simulation results reveal accurate and fast response of the proposed systems. The dynamic performance of the proposed feed-forward strategy outdoes that of the feedback strategy in terms of accuracy and response time. Moreover, an experimental prototype is built to test and validate the proposed PV interface system.

## I. Introduction

Nowadays, renewable energy resources play an important role in replacing conventional fossil fuel energy resources. Photovoltaic (PV) energy is one of the very promising renewable energy resources which grew rapidly in the past few years. The main challenge with a PV array is the variation of its operating voltage that results in maximum power extraction due to the variation of the weather conditions. In the last decades, the PV interfacing systems received a great deal of attention.

Searching for the desired PV operating voltage that results in maximum power is known as Maximum Power Point Tracking (MPPT) of a PV array. The MPPT is achieved by controlling the power electronics converters which interface the PV array to the grid. There are numerous algorithms that can be employed for the MPPT of the PV array such as the Perturb and Observe (Hill climbing) method [1], the Incremental Conductance method [2–5], the Fractional open circuit voltage method [6–7], the Fractional short circuit Current method, and those based on Artificial Intelligence (AI) methods [8].

There are numerus algorithms of AI, such as; Fuzzy logic [9–12], Neural Networks [13–14], adaptive unscented kalman filtering [15], and etc. Both conventional and AI based MPPT methods have their advantages and drawbacks. Conventional methods are famous for their easy implementation and compatibility to operate with any PV array, while they suffer from relatively slower response compared to the AI methods [8, 16]. On the other hand, AI methods show very fast response under any operating condition changes, give very accurate results, and they are able to work under instant temperature or solar irradiance changes efficiently. The drawbacks of the AI based MPPT methods include design complexity and need for fast processors to be implemented on real time [8, 16]. The MPPT based on ANN owns the advantage of providing the optimum operating point of the PV array without requiring extensive knowledge for the PV parameter. However, it must be clarified that as most PV arrays shows unlike output characteristics, an ANN should be explicitly trained for the PV array with which it will be utilized [17].

In recent years, the use of High-Frequency (HF) transformers in place of power transformers is considered to be the developing trend of next-generation of power conversion. The HF link overwhelms the traditional interface system in terms of size, cost, and power density [18–20]. In addition, when the switching frequency is above 20 kHz, noise can be greatly reduced [21]. One of the most promising HF DC/DC converters is the Dual Active Bridge (DAB) converter [22].

This paper presents an interface system for the PV array based on the DAB converter. The main advantage of the proposed DAB converter is the isolation between the PV side and the load side. Moreover, the use of high frequency converter results in a reduction of the size of the interface system. Two control strategies are proposed in order to control the DAB converter. The first strategy is based on the feedback control to regulate the PV terminal voltage by adjusting the phase shift angle of the DAB converter. The Perturb and Observe (P&O) MPPT scheme is adopted to set the reference value of the PV terminal voltage. On the other hand, an ANN based MPPT is employed for the second proposed strategy to set directly the phase shift angle of the DAB. The second control scheme outdoes the first one by being a feed-forward strategy where the stability is guaranteed. The Matlab/Simulink and the EMTDC/PSCAD software packages are used to model and simulate the proposed PV interface systems. The results of the two control strategies are compared. Furthermore, a hardware prototype is used to validate the proposed PV interface system.

## II. The Dual Active Bridge Converter (DAB)

**Fig 1:**
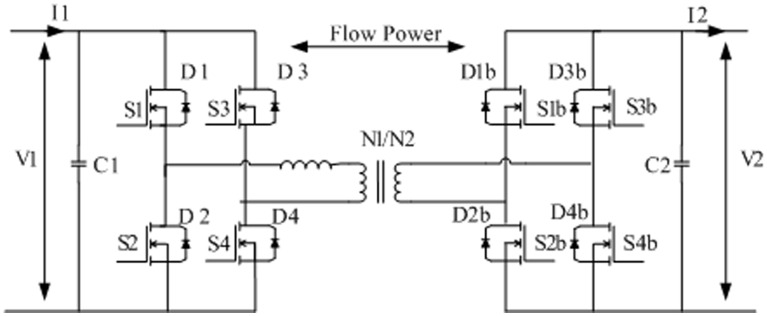
Dual Active Bridge Converter.

The DAB converter comprises of a high frequency transformer which is located between two H-Bridges as shown in Fig 1. The two H-bridges are controlled to generate high frequency square-wave voltages with the same frequency and fixed duty cycle of 50%. In order to control the power flow through the high frequency transformer, the phase shift between the two square wave voltages is controlled. According to which bridge produces the leading square wave, the DAB converter can control the direction of the power flow. Once the square wave voltage generated at terminal 1 leads that generated at terminal 2, the power flows from the supply connected to terminal 1 to terminal 2 and vice versa. The relationship between the DC-side terminal voltages of the DAB converter and the output power is given by [6]:

$$P=\frac{V_{1}V_{2}}{a\omega L}\varphi \left(1-\frac{\left|\varphi \right|}{\pi }\right)$$ (1)

where V_1_ and V_2_ are the DC-side voltages of the two bridges, φ is the phase shift angle between the AC voltages produced from the two H-bridge converters, ɑ is the turns ratio of the transformer, L is the total series inductance, and *ω = 2πf* is the angular frequency where f is the operating frequency of the converters.

## III. The Proposed Control Strategies for the DAB Based PV Interface system

**Fig 2:**
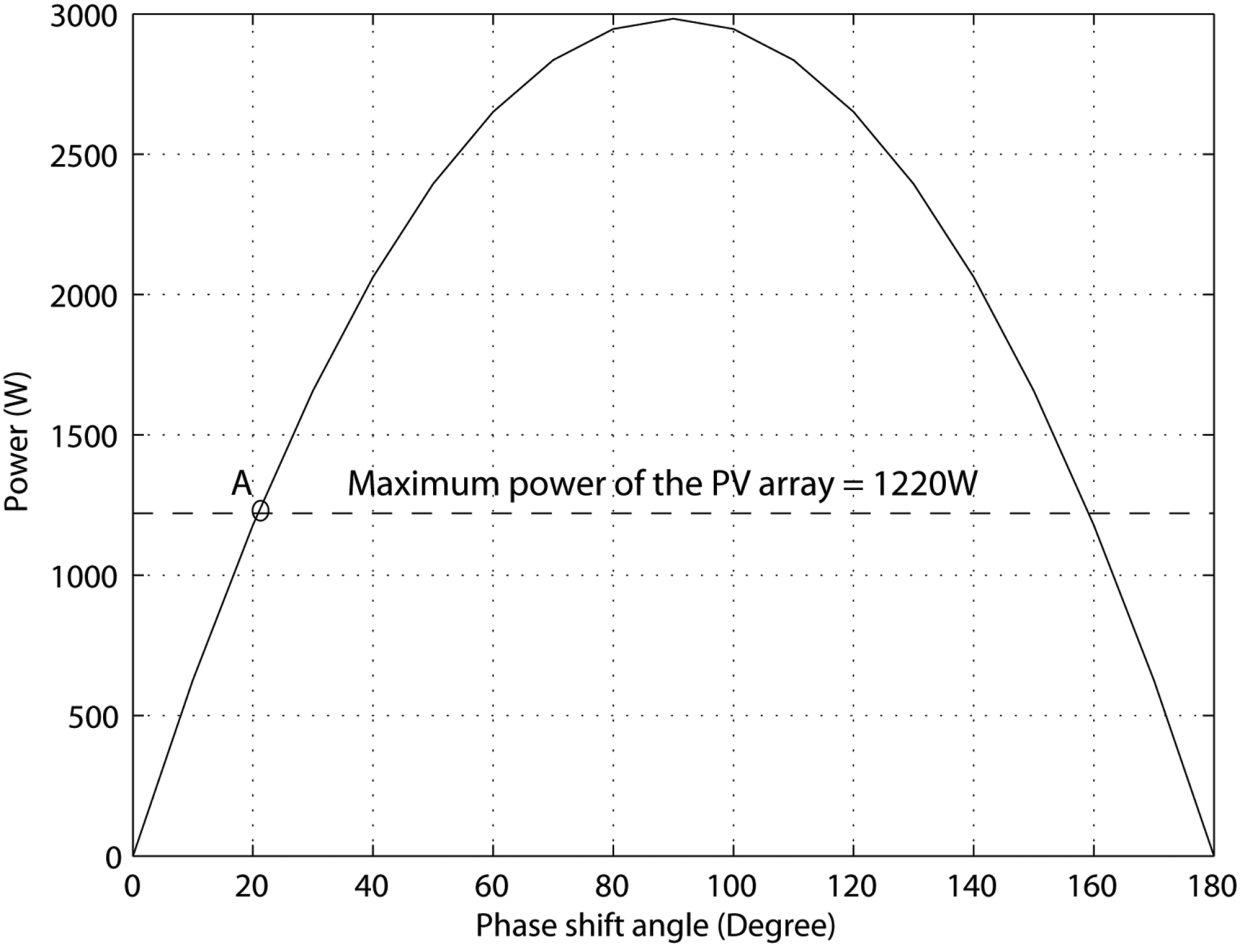
The DAB characteristics.

To extract the maximum power from the PV array, the characteristic of the PV array must meet the DAB characteristic at an operating point. With the aid of (1) and the system parameters of Table 1, Fig 2 is drawn to illustrate the power characteristic of the DAB converter as a function of the phase shift angle φ. It is worth mentioned that the DAB characteristics is obtained where the input voltage V1 is set at the value corresponding to the maximum power point of the PV array at nominal conditions, 110 V. As a result, the operating point at which the maximum power harvested from the PV array is indicated at point A of Fig 2 where φ = 21°. Since this value cannot be exceeded, it is set as a limit value for the proposed controller [23]. In the next two subsections, the proposed control algorithms are described.

**Table 1:** Parameters of the DAB.

Quantity	Value
DC link capacitor C2	2 mF
DC link Voltage V2	400V
Turns ratio N1/N2	1/4
Coupling inductance of the DAB including the transformer	461 μH
DC link capacitor C1	4000 μF
Switching frequency of the DAB	1 kHz

### A. The proposed feedback strategy using the P&O algorithm

**Fig 3:**
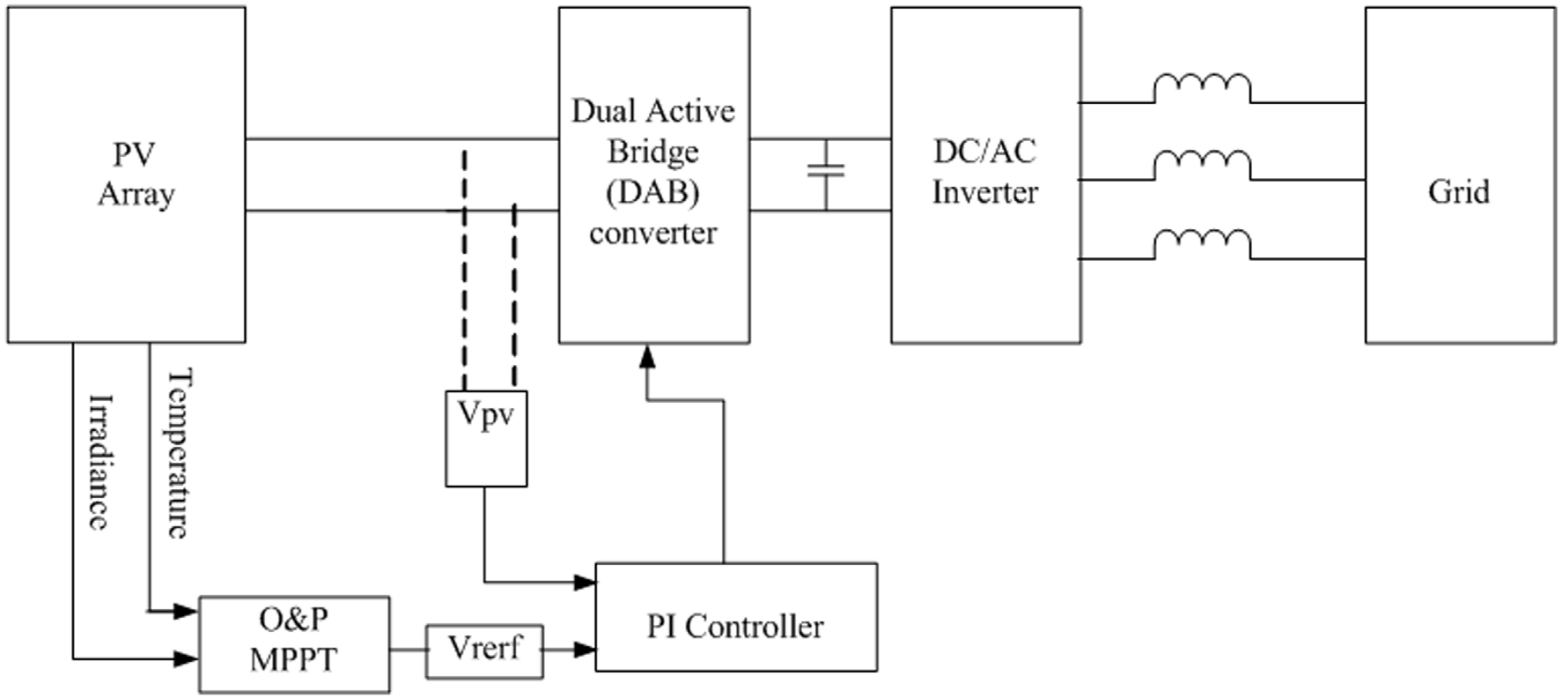
Block diagram of the proposed feedback strategy for DAB based PV interface system.

A Schematic diagram of the proposed feedback strategy for the DAB based PV interface system using the P&O MPPT method is illustrated in Fig 3. The maximum power point is achieved by controlling the phase shift angle *φ*. The P&O MPPT method is employed to determine the desired reference of the PV terminal voltage that results in maximum power point. This reference voltage is compared with the actual PV voltage and the error is processed by a simple PI controller with the parameters given in Table 2. The PI controller adjusts the angel *φ* needed to operate the DAB converter. A limiter is set on *φ* as described to prevent unstable operation. Finally, the grid-connected inverter is controlled to regulate the DC-link voltage, at the output side of the DAB V_2_, at the desired setting. This action results in delivery of the maximum power harvested from the PV to the grid.

**Table 2:** Parameters of the PI controller.

Proportional gain	0.01
Integral gain	0.001

### B. The proposed feed-forward strategy based on ANN

**Fig 4:**
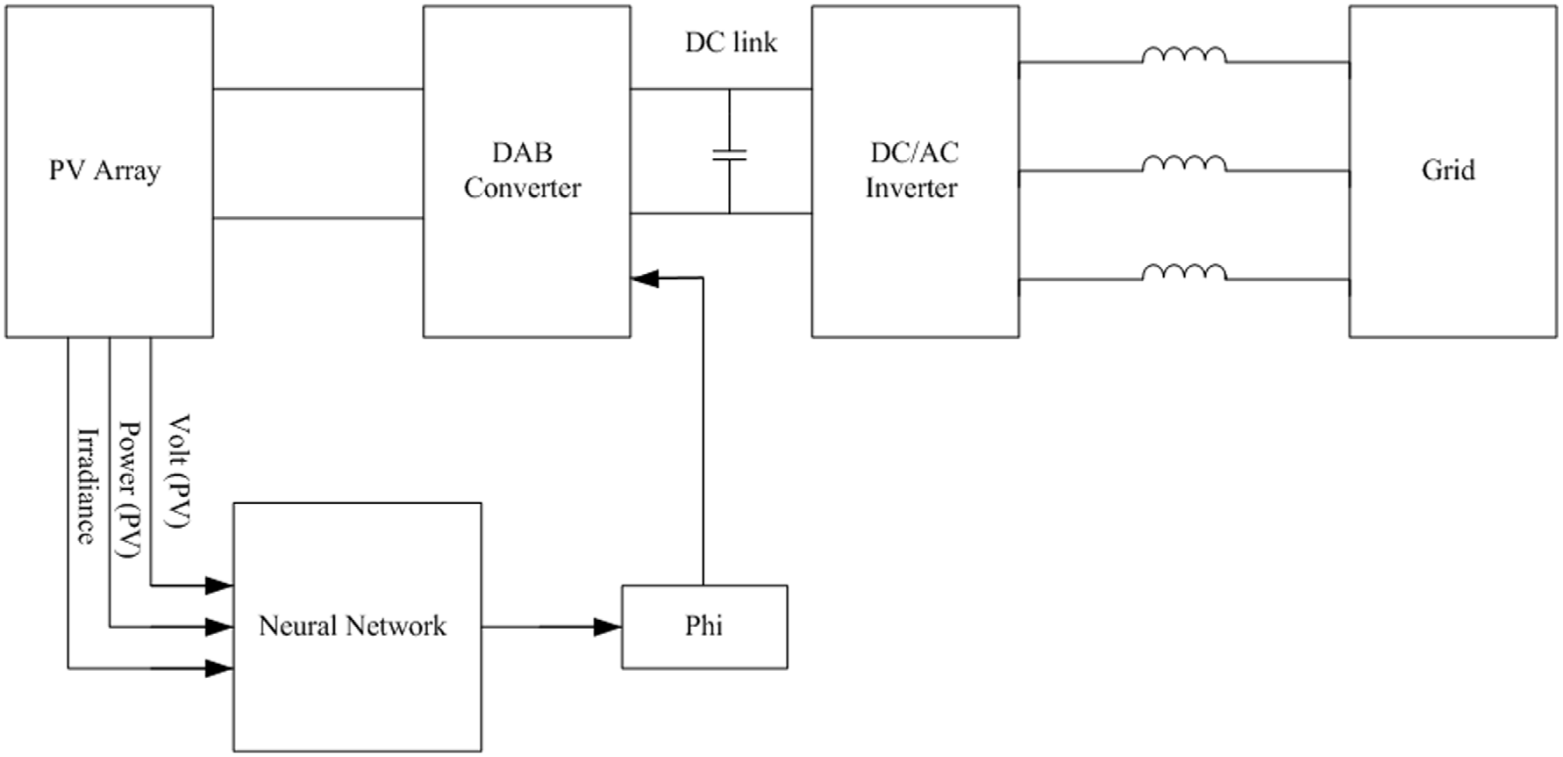
Block diagram of the proposed feed-forward strategy for DAB based PV interface system.

**Fig 5:**
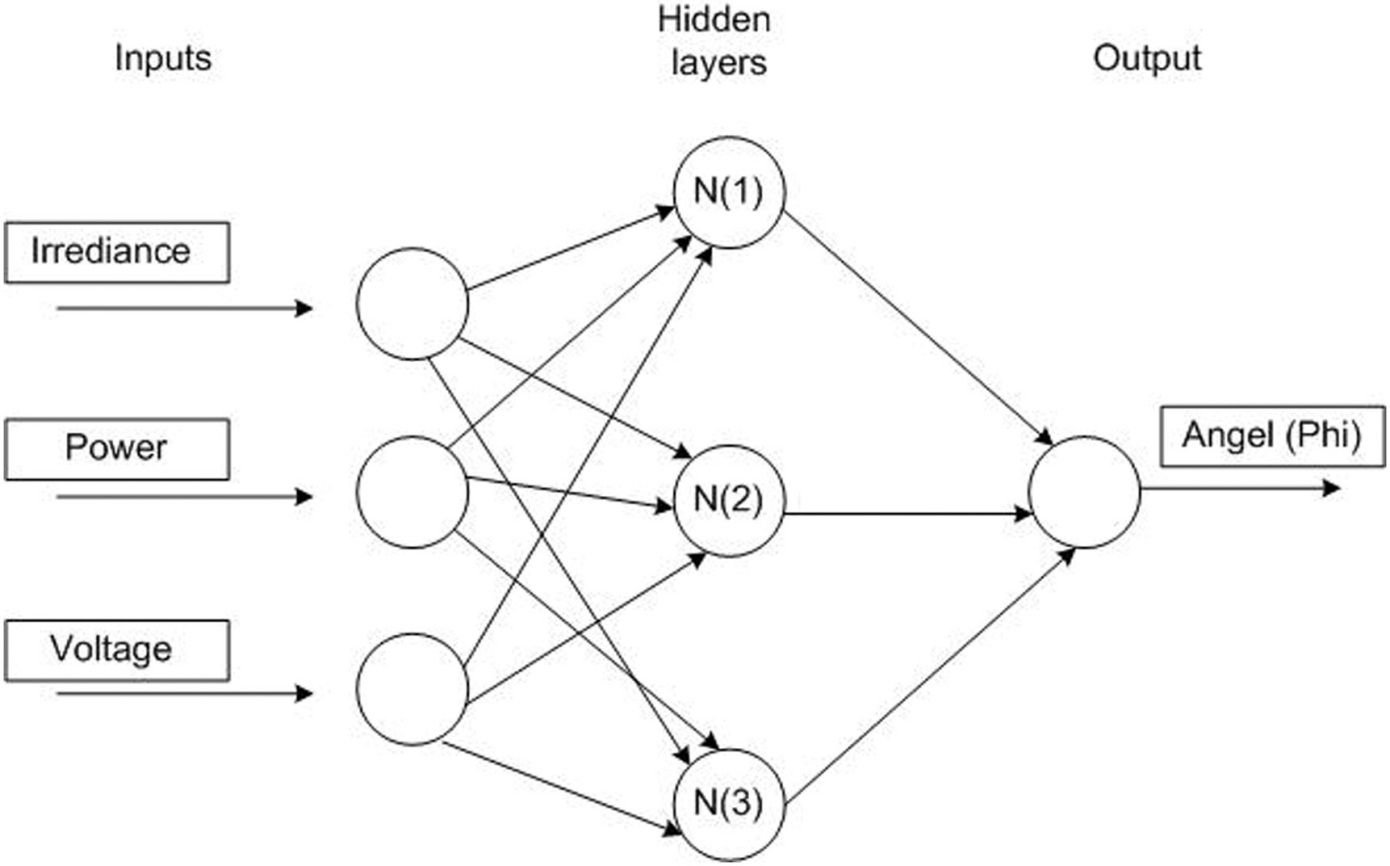
ANN structure.

A schematic diagram of the proposed feed-forward strategy for the DAB based PV interface system utilizing ANN is presented in the Fig 4. The proposed ANN consists of three layers; input layer, hidden layer, and output layer as shown in Fig 5. The ANN has three inputs; solar irradiance, and array power and voltage, where its output is the phase angle φ of the DAB converter. The hidden layer has three neurons with tan-sigmoid activation function and the output layer has only one neuron with pure-linear activation function.

**Fig 6:**
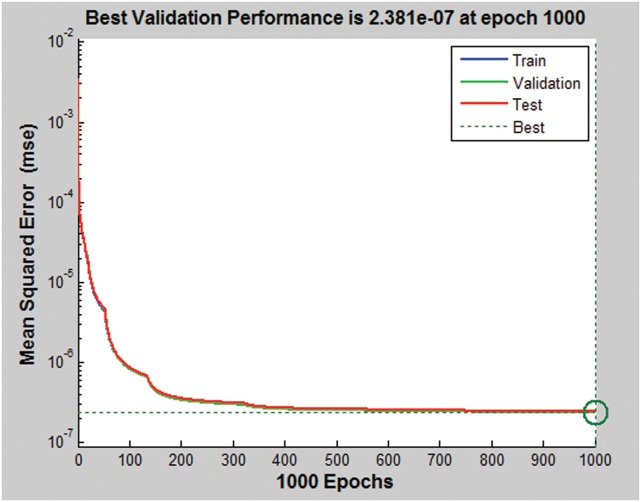
The performance of ANN.

The first step in designing an ANN is to collect historical data on the problem that is being solved using the network. In case of MPPT of DAB based PV system, array consist of voltage and power, solar irradiance, and the corresponding maximum power point phase angle are required to train the network. The collected training points are obtained from the PV model under the MATLAB/Simulink to teach the network how to perform at different conditions. Around 19394 training points are extracted from the Matlab/Simulink Model, see S1 Table. Note that the Matlab/Simulink modeled was verified by the first author in the reference number [19]. The MATLAB/ANN toolbox is used to train the network. Some of the collected points are kept as test points. The function of test points is to test the performance of the designed ANN after its training is finished. Fig 6 illustrates the performance of the ANN.

## IV. Simulation Results and Discussion

Different case studies are conducted to evaluate the dynamic performance of the proposed control strategies for the DAB based PV interface system. The PV array, considered in this paper, comprises of two parallel strings where each string consists of two PV modules connected in series. Table 3 indicates the SUNPOWER PV module parameters supplied from the manufacturer. This PV array can produce at nominal conditions, 25°C and 1000 W/m^2^, a maximum power of 1220 W.

**Table 3:** PV module parameters at nominal operation conditions (25°C- 1000 W/m^2^).

Quantity	Value
Current of the PV module at the maximum power point	5.58A
Voltage of the PV module at the maximum power point	54.7V
Maximum power of the PV module	305.2 W
Short circuit current of the PV module	5.96A
Open circuit voltage of the PV module	64.2V
Leakage current of the PV module, Io	1.17x10-8 A
Nominal photovoltaic current, IPVn	5.960A
Diode ideality constant, α	1.3
Parallel resistance, Rp	993.51 Ω
Series resistance, Rs	0.379

### A. Simulation of the proposed feedback strategy

**Fig 7:**
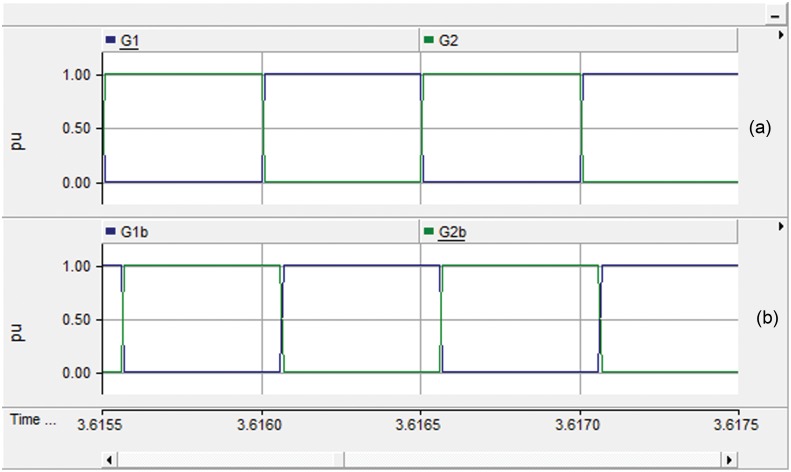
Gate pulses; (a) PV-side bridge and (b) DC-link-side bridge.

The proposed feedback strategy presented in Fig 3 is modeled and simulated using the EMTDC/PSCAD software package. The system parameters are shown in Tables 1 and 3. Firstly, the behavior of the DAB converter is investigated under nominal operating condition of the PV module. Fig 7 displays the gate pulses for each bridge of the DAB converter. The gate pulses for transistors S1 and S4 of the PV-side bridge are shown in Fig 7(a). Fig 7(b) illustrates the gating signal for the DC-link-side Bridge which is lagging the PV-side bridge as expected. The phase shift angel φ which is generated from the PI controller depends on the operating point on the PV curve.

**Fig 8:**
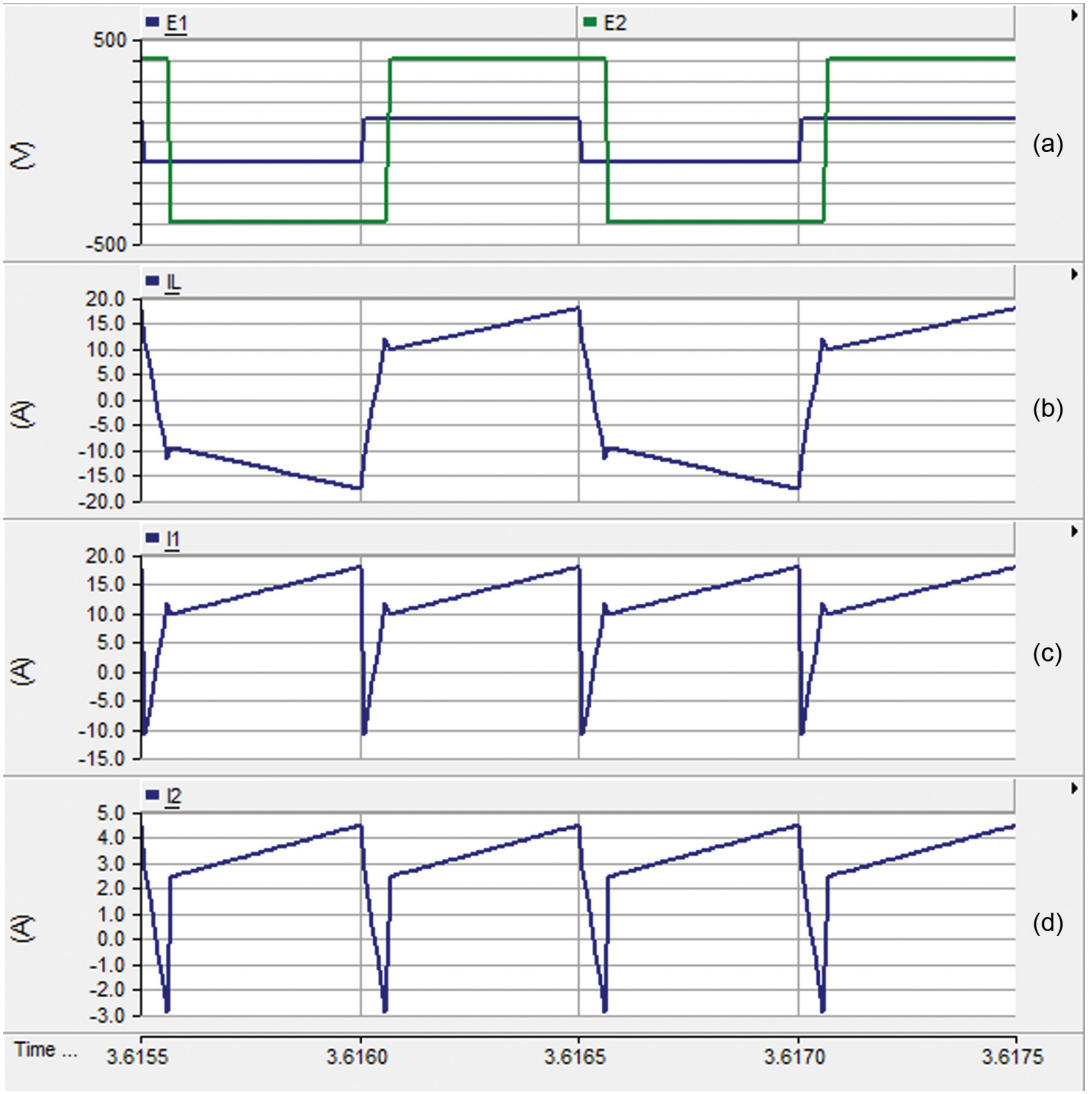
Performance of the DAB converter: (a) the voltages at the terminals of the high frequency transformer, (b) the input current to the high-frequency transformer, (c) the input current to the DAB, and (d) the output current from the DAB.

Fig 8 investigate the performance of the DAB converter. The voltage at the primary and the secondary windings of the high frequency transformer are traced in Fig 8(a). The phase shift, shown in Fig 7, and the 1:4 turns ratio of the transformer are evident from this result. Fig 8(b) portrays the input current to the high frequency transformer. The input and output currents of the DAB converter are illustrated in Fig 8(c) and 8(d), respectively.

**Fig 9:**
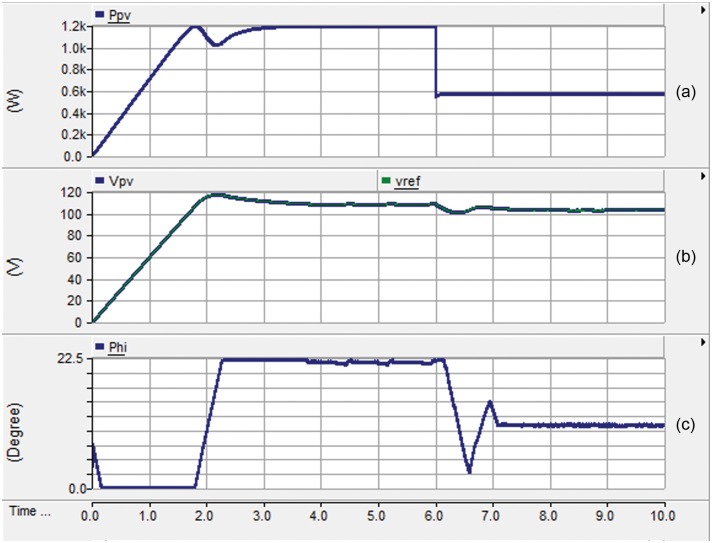
Dynamic performance of the proposed feedback strategy: (a) the harvested power from the PV, (b) the actual and reference voltage of PV, and (c) the phase shift angle φ.

Secondly, the dynamic performance of the proposed feedback strategy for the PV interface system is investigated. Fig 9(a) portrays the harvested power from the PV array where the irradiance is reduced from 1000 W/m^2^ to 500W/m^2^ at t = 6s. This hypothetical abrupt reduction of the irradiance is to evaluate the dynamic performance of the proposed system. It is obvious that the proposed interface system succeeds in harvesting the maximum power from the PV even at the dynamic condition. As expected, the harvested maximum power is reduced to half of the rated value when the irradiance is reduced to half of its nominal value. The PI controller is tightly regulating the terminal PV voltage at its reference value set by the P&O MPPT technique as demonstrated in Fig 9(b). This action results from the fast response of the proposed controller in adjusting the phase shift angle φ to control the DAB converter as illustrated in Fig 9(c).

### B. Simulation of the proposed feed-forward strategy

**Fig 10:**
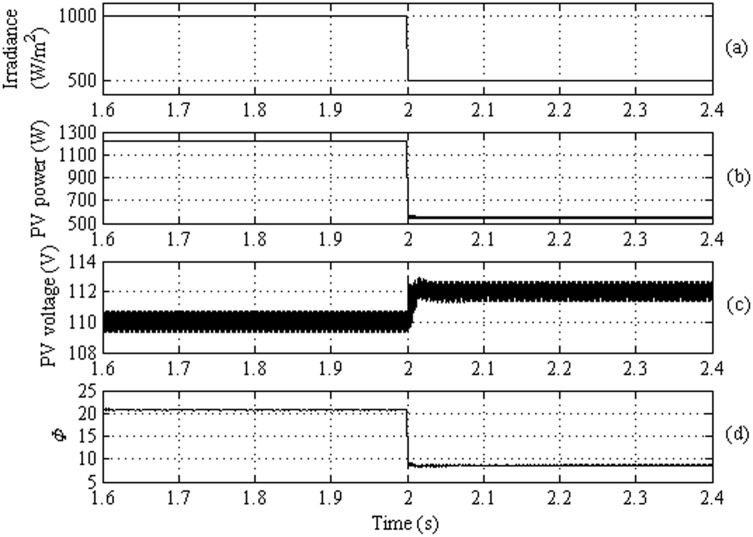
Dynamic Performance of the proposed feed-forward strategy: (a) the PV irradiance, (b) the PV power, (c) the PV terminal voltage, and (d) the phase shift angle φ.

This section is dedicated to investigate the dynamic behavior of the proposed feed-forward strategy for the DAB based PV interface system. The MATLAB/SIMULINK software is used to simulate system presented in Fig 4. Similar to what have been done in the case of feedback strategy, a sudden change in the irradiance from 1000 W/m^2^ to 500 W/m^2^ is applied at t = 2sec, as shown in Fig 10(a). Fig 10(b) illustrates the dynamic change in the harvested power which results in response to the irradiance change. Fig 10(c) portrays the operating DC voltage at the terminal of the PV array. It is obvious that the variation in the PV voltage is small due to the steep power-voltage characteristic of the PV module. The corresponding phase shift angle φ which is predicted using the proposed ANN based MPPT technique is exhibited in Fig 10(d). This result revealed that the proposed feed-forward strategy based on the ANN succeeds in controlling φ instantly without overshoot to generate the maximum power from the PV system.

**Fig 11:**
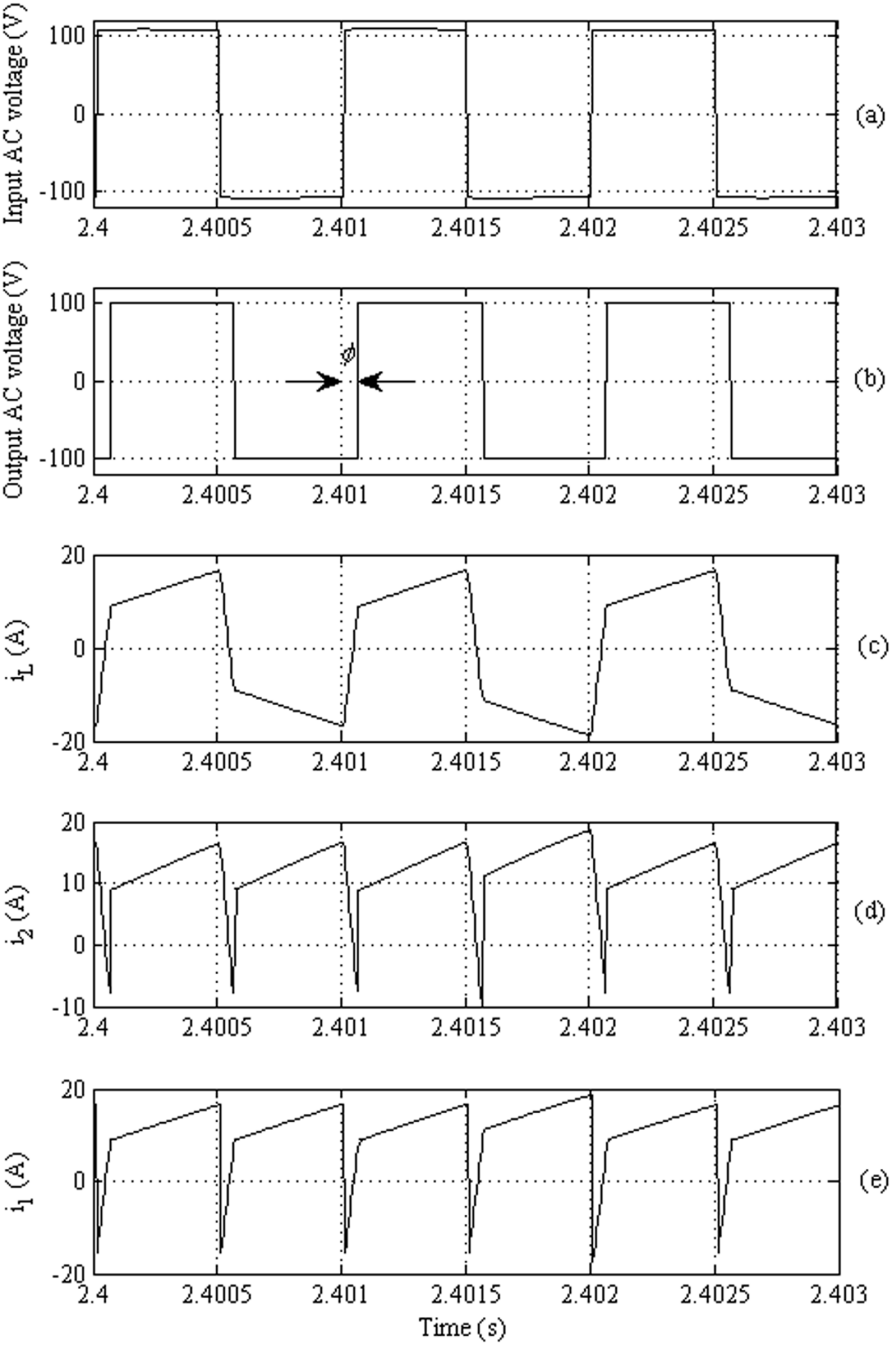
Performance of the DAB converter: (a) The input AC voltage, (b) the output AC voltage, (c) the input current to the high-frequency transformer, (d) the output current to the DAB (e) The input current from the DAB.

Fig 11(a) and 11(b) demonstrate the phase shift between the input and output AC voltages across the HF transformer, respectively. Fig 11(c) illustrates the coil current which follows the same waveform of Fig 8(b). Fig 11(d) and 11(e) plot the output and input currents of the DAB which agree with the results shown in Fig 8.

Tables 4 and 5 explore the steady state performance of the proposed feedback and feed-forward strategies, respectively. It is clear that the proposed feed-forward strategy based on ANN is more accurate in tracking the maximum power compared to the feedback strategy. Moreover, the response time of the feed-forward strategy is faster than the feedback strategy as can be revealed from Figs 9 and 10. The ANN determines the required angle φ to harvest the maximum power at the same instant of irradiance change. The PI controller takes longer time to settle because it cannot be tuned to accommodate for all dynamic changes. On the other hand, the ANN can be trained for the various dynamics of the system to predict the angle φ directly.

**Table 4:** Steady state performance of the feedback strategy.

Irradiance	Power	φ
1000 W/m2	1200 W	21.5°
500 W/m2	573 W	10.5°

**Table 5:** Steady state performance of the feed-forward strategy.

Irradiance	Power	φ
1000 W/m2	1220 W	20.91°
500 W/m2	580 W	9.5°

## V. Experimental Results

**Fig 12:**
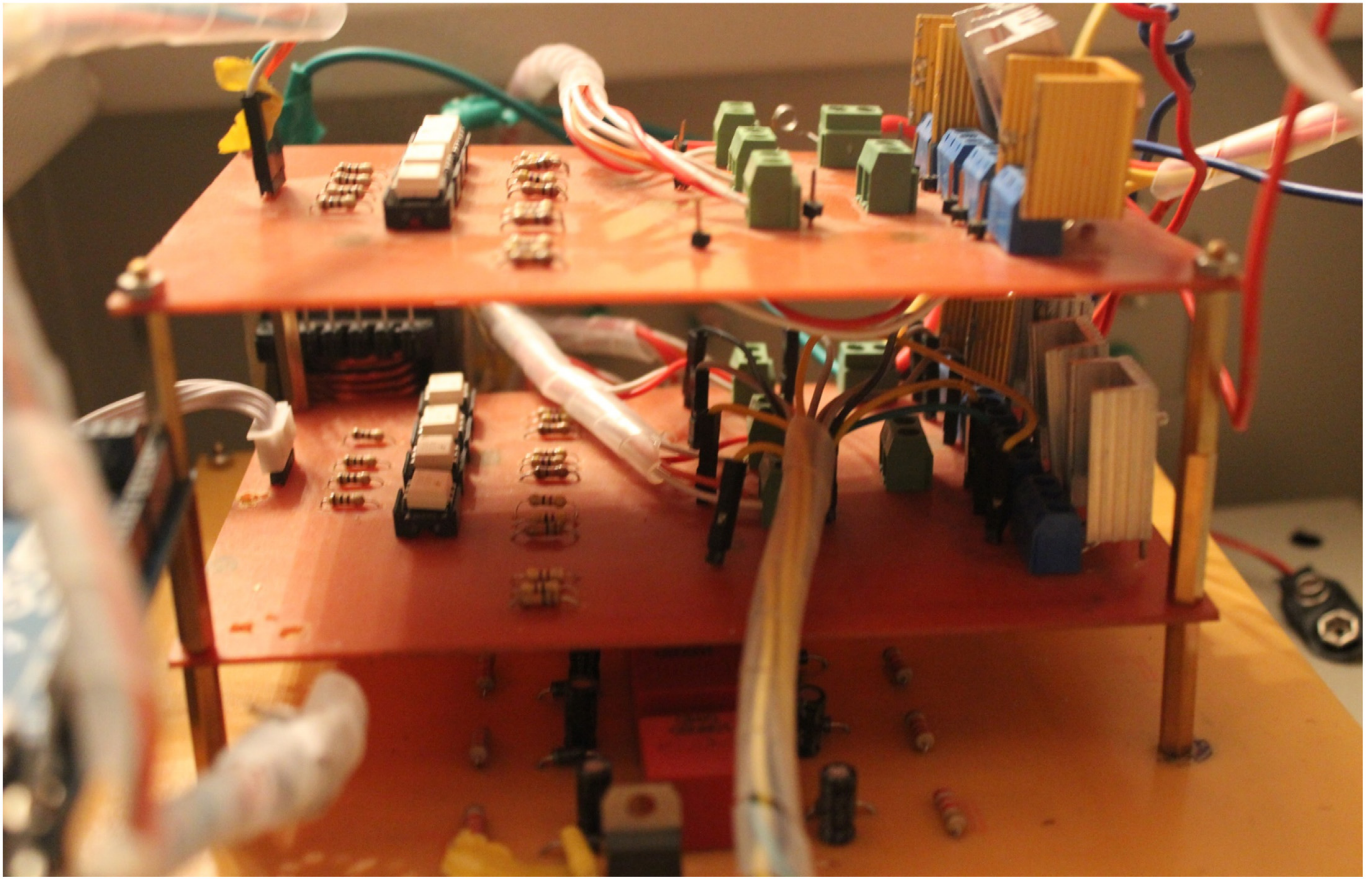
The experimental prototype of the DAB converter.

**Fig 13:**
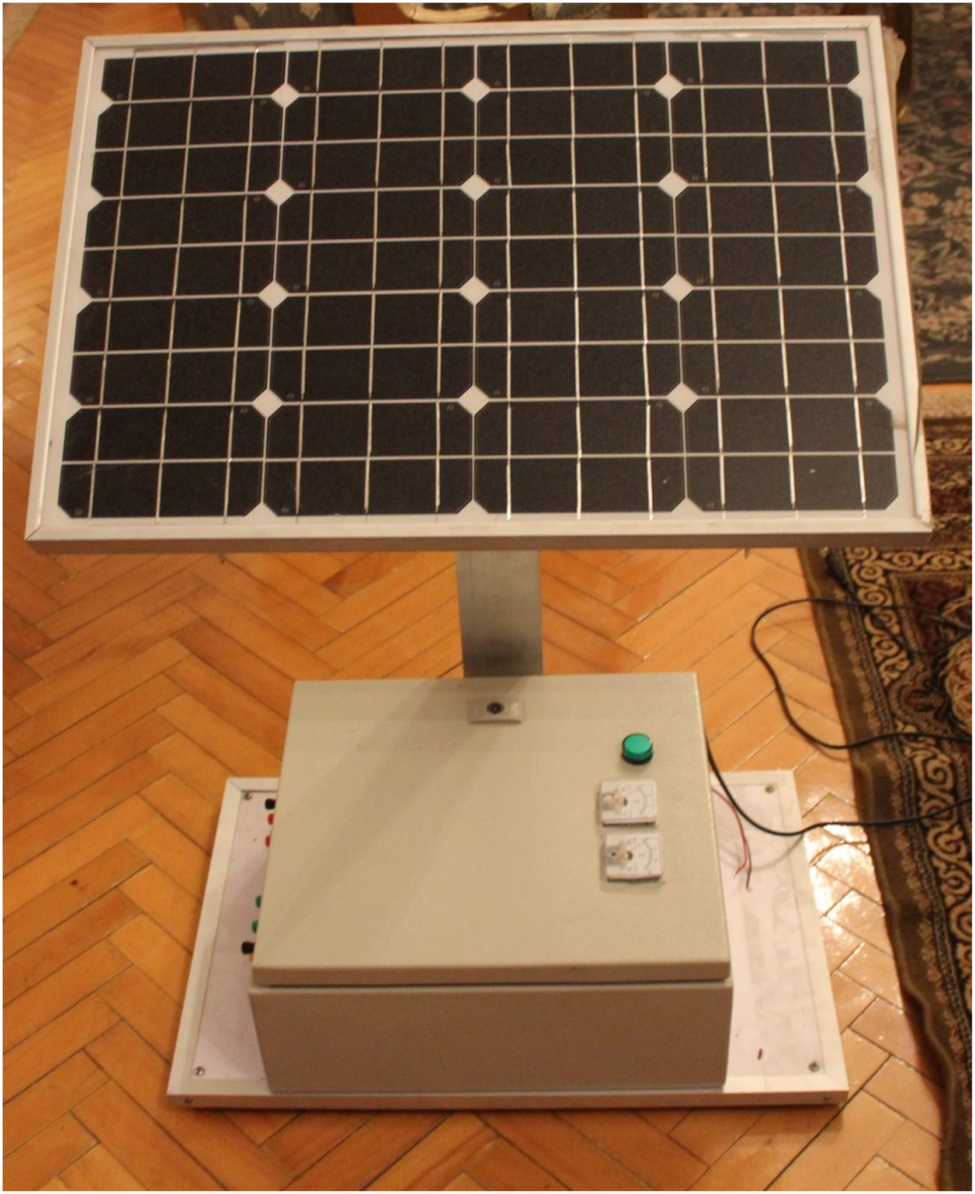
The experimental prototype of the DAB based PV interface system.

**Fig 14:**
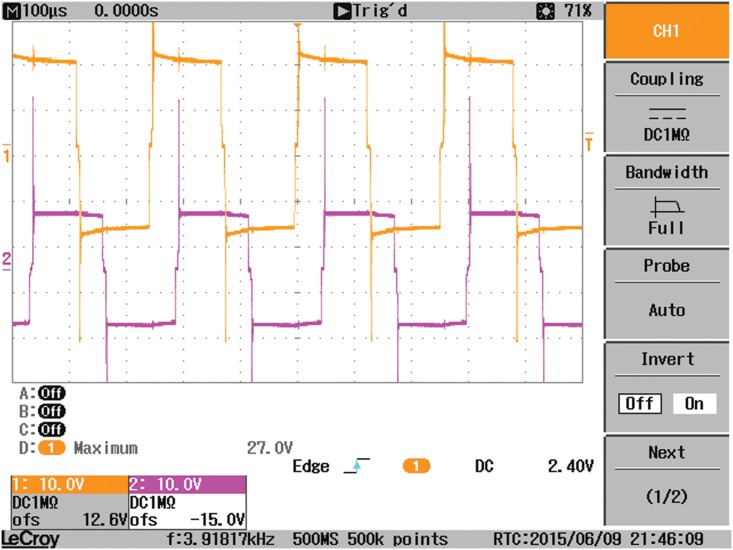
The voltages at the terminals of the coil connecting the two bridges of the DAB, V1 and V2.

A laboratory prototype of the DAB converter is implemented using the MOSFET switches IRF540. A coil is utilized to represent the high frequency transformer. A battery is connected to the output-side of the DAB to represent the grid. All tests have been carried out in sunny days where the PV module is connected to the input-side of the DAB converter. The experimental prototype of the DAB converter is shown in Fig 12. It is consist of three boards, one for the Switch Mode Power Supply (SMPS) and the other two boards are similar for the two H-bridges of the DAB converters. A SMPS is built to provide six isolated fifteen voltage supplies for the driving circuits of the MOSFET switches used in the DAB converter. Each H-bridge with the associated driving circuits is implemented in one board. The control signals for the different switches are obtained from the Arduino(MEGA 2560) microcontroller. The overall experimental prototype of the DAB based PV interface system is displayed in Fig 13. The parameters of the experimental setup are presented in Table 6. The input voltage to the DAB converter from the PV module varies from 18V, at the maximum power point, to 21V, at open circuit condition. The voltages at the terminals of the coil connecting the two bridges of the DAB, V_1_ and V_2_, are portrayed in Fig 14. Since the magnitude of V_1_ is around 18V, the phase shift angle φ between the two square waveforms, V_1_ and V_2_, succeeded to operate the DAB converter to harvest the maximum power from the PV module.

**Table 6:** Parameters of the experimental setup.

Quantity	Value
PV voltage: V1	18 to 22 V
Battery Voltage V2	12V
Coupling inductance of the DAB	1.4mH
Switching frequency of the DAB	3.9 kHz
Load resistance	21 Ω
Load capacitor C2	2200 μF

**Fig 15:**
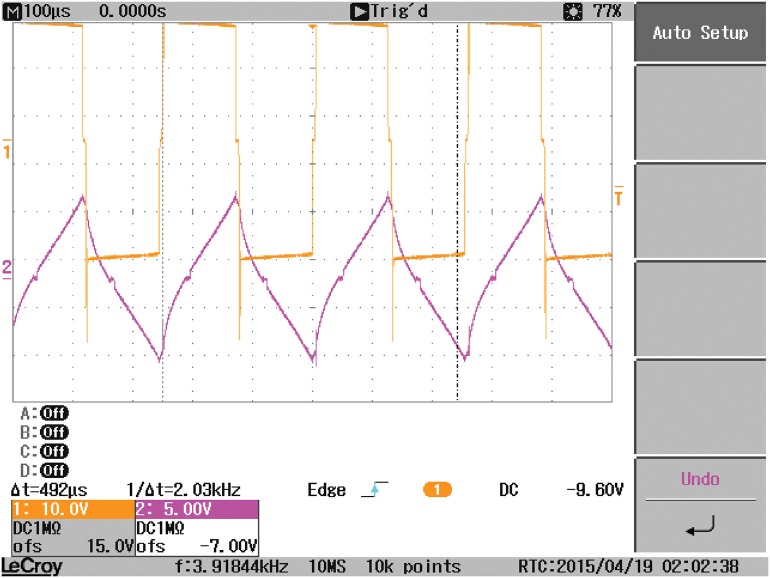
The voltages at the terminals of the coil connecting the two bridges of the DAB, V1 and V2 when a resistive load is connected to the output-side.

**Fig 16:**
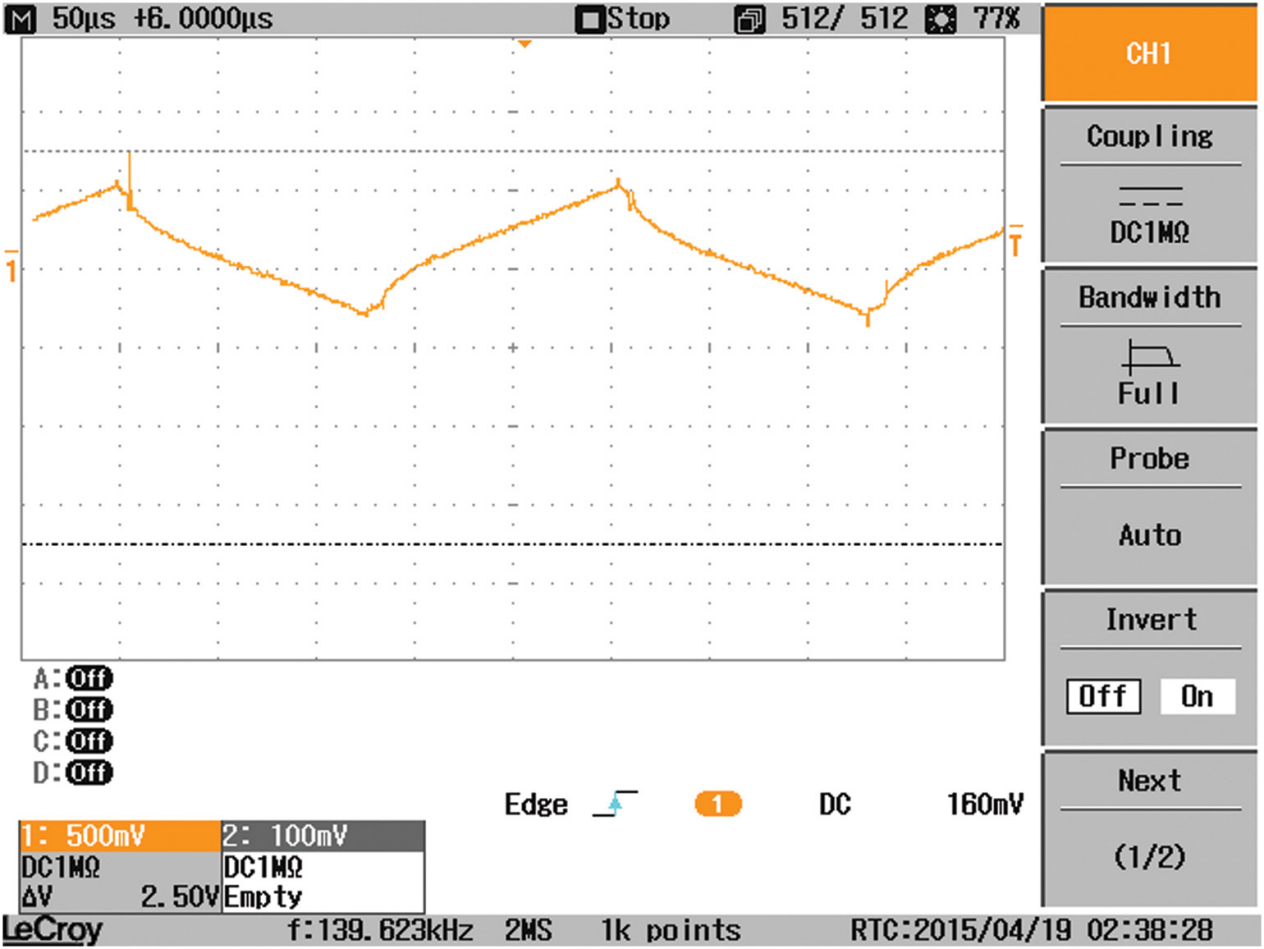
Coil current when a resistive load is connected.

**Fig 17:**
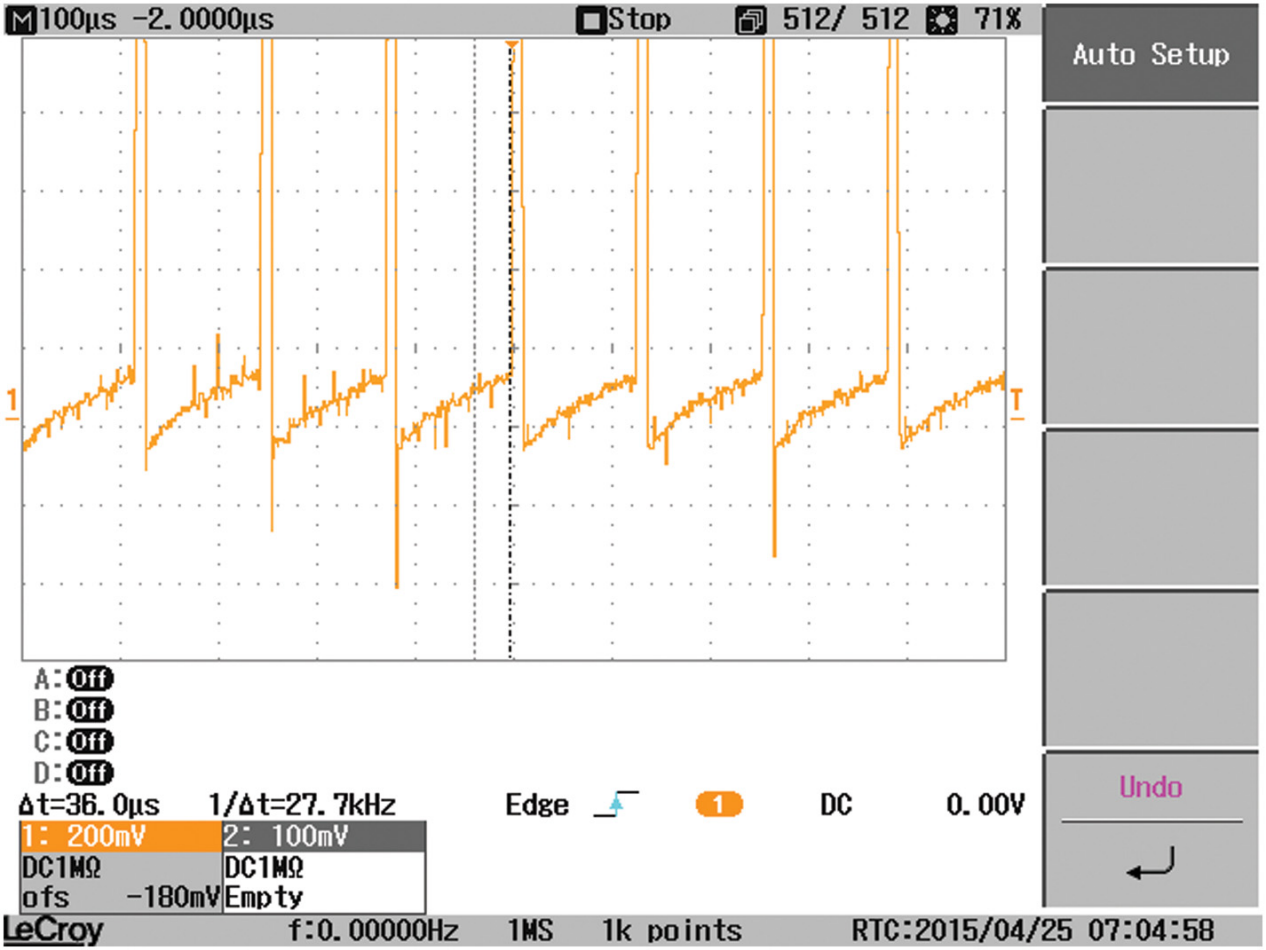
The input current I1 to the DAB converter when a resistive load is connected.

**Fig 18:**
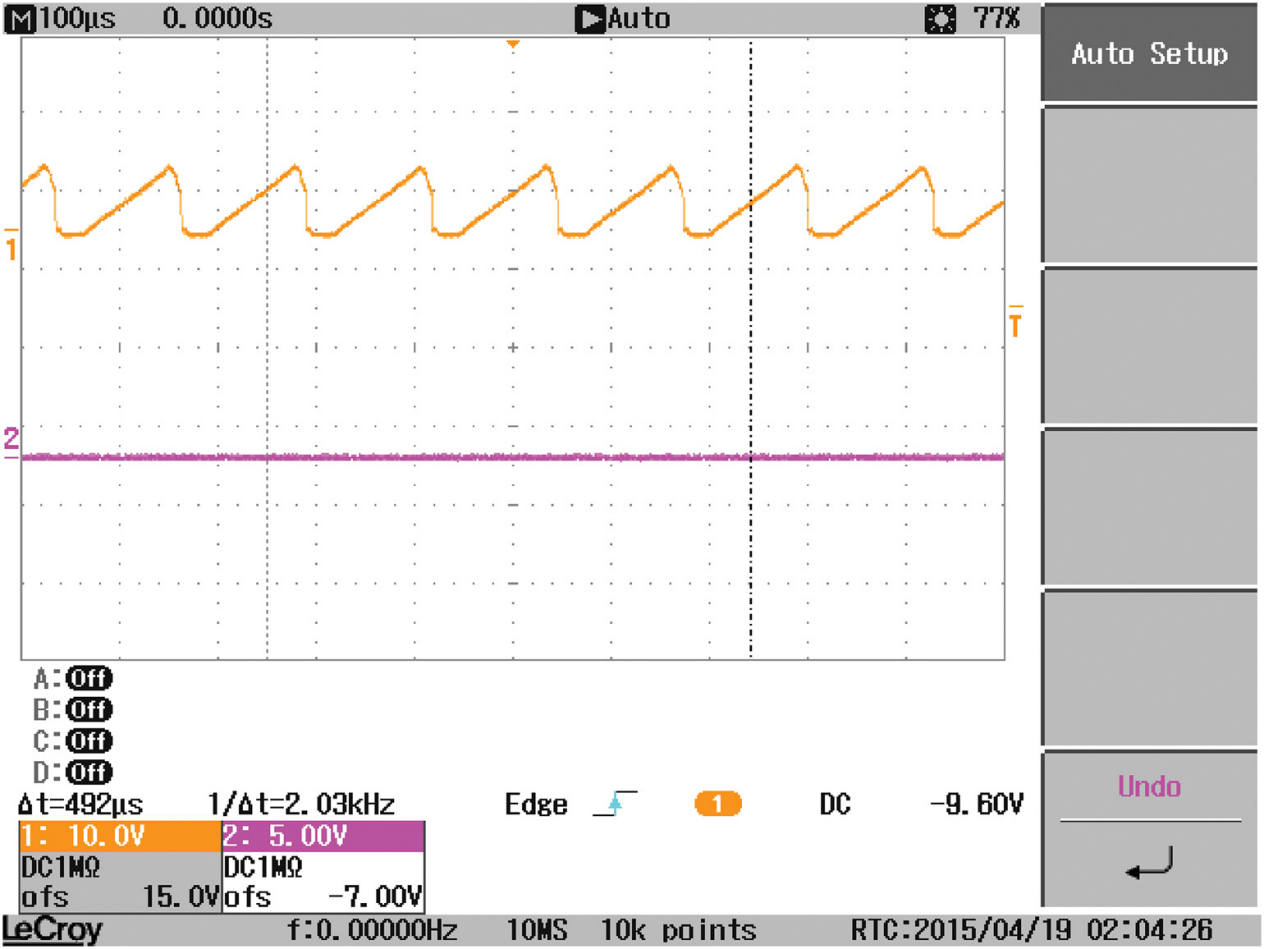
The load current when a resistive load is connected.

Another study case is carried out where the output-side of the DAB converter is connected to a resistive load, in parallel with a capacitor C2, instead of the battery. As a result, the waveform of V_2_ is changed as demonstrated in Fig 15. The waveforms of the coil current, input current I_1_, and the load current are affected by the type of the load connected as indicated in Figs 16, 17 and 18, respectively.

## VI. Conclusions

In this paper, an interface system based on the DAB converter is proposed for PV arrays. Moreover, two control strategies are presented for the proposed PV interface system. The first strategy is based on a feedback scheme which employs a PI controller to regulate the terminal PV voltage at a reference value determined by the P&O technique for MPPT. The action of the PI controller is the phase shift angle of the DAB converter. The second strategy utilizes a feed-forward scheme based on ANN to directly set the phase shift angle for the DAB converter that results in MPPT. The first technique is modeled and simulated under the EMTDC/PSCAD environment while the second one is modelled and simulated under the MATLAB/SIMULINK. The simulation results show that the feed-forward approach based on ANN has a faster response and accurate performance compared to the feedback technique. Furthermore, an experimental prototype is implemented to test and validate the operation of the proposed DAB converter for interfacing a PV array.

## Supporting Information

S1 Table The training data of the artificial neural network. (DOCX)

## References

[1] Villalva M. G., Gazoli J. R. and Ruppert F E. “Analysis and simulation of the P&O MPPT algorithm using alinearized array model”. Power electronics conference, 2009, Brazil. pp 189–195

[2] Safari A. and Mekhilef S.. “Simulation and hardware implementation of incremental conductance MPPT with direct control method using cuk converter”. IEEE Transaction on industrial electronics. 2011 Vol. 58 No 4 pp 1154–1161.

[3] Safari A. and Mekhilef S. “Implementation of incremental conductance method with direct control”. IEEE region 10 conference ICON, 2011, Malaysia. Pp 944–948.

[4] Menniti D., Burgio A., Sorrentino N. and Pinnarelli A. “An incremental conductance method with variable step size for MPPT design and implementation”. 10th international conference on electrical power and quality and utilization. 2009, Poland. pp 1–5.

[5] Xuesong Z., Daichum S., Yoijie M. and Deshu C. “The simulation and design for MPPT of PV system based on Incremental conductance Method”. WASE international conference on information engineering, 2010. pp 314–317.

[6] Ahmed J. “A fractional open circuit voltage based maximum power point tracker for photovoltaic arrays”. 2nd international conference on software technology and engineering. 2010. pp V1-247 - V1-250.

[7] Adly M., el-sherif H. and Ibrahim M. “Maximum power point tracker for a PV cell using fuzzy agent adapted by the fractional open Circuit Voltage Technique”. IEEE international conference on fuzzy systems, 2011, Taiwan. pp-1918–1922.

[8] Salah C.B. and Ouali M.. “Comparison of fuzzy logic and neural network in maximum power point tracker for PV systems,” Electric Power Systems Research, Vol. 81, 2011, pp. 43–50.

[9] Diaz N., Luna A. and Duarte O. “Improved MPPT short-circuit current method by a fuzzy shor-circuit current estimator”. Energy converstion conference and exposition, 2011, Colombia. pp 211–218.

[10] Diaz N., Hernandez J. and Duarte O.. “Fuzzy MPP method improved by a short circuit current estimator, Applied to a grid- connected PV system”. IEEE 12th work shop on control and modeling for power electronics. 2010, Colombia pp 1–6.

[11] Subiyanto A. Mohamed and M.A.Hannan. “Maximum Power Point Tracking in Grid Connected PV System using A Novel Fuzzy Logic Controller”. IEEE student conference on research and development, 2009. pp 349–352

[12] Kotha V. “Maximum Power Point Tracking With Fuzzy Logic Approach for Grid Connected Photovoltaic System,” International Conference on Industrial and Information Systems (ICIIS), 2010, India

[13] Sulaiman S.I., Abdul Rahman T.K., Musirin I. and Shaari S. “Optimizing Three-layer Neural Network Model for Grid-Connected Photovoltaic output prediction”. Conference on innovative technologies in intelligent systems and industrial applications.2009. Malaysia. pp 338–343

[14] Punitha K., Devaraj D., and Sakthivel S., “Artificial neural network based modified incremental conductance algorithm for maximum power point tracking in photovoltaic system under partial shading conditions,” Energy, Vol. 62, 2013, pp. 330–340.

[15] Sum F., Hu X., Zou Y., and Li S. “Adaptive unscented Kalman filtering for state of charge estimation of a lithium-ion battery for electric vechicles”, Energy, Vol. 36, no 5, pp. 3531–3540, 2011.

[16] Lei Z., Zhenpo W., Xiaosong H., Dorrell D. G., “Residual Capacity Estimation for ultracapacitors in electric vehicles using artificial neural network”, The International Federation of Automatic Control, 2014, South Africa.

[17] Esram T. and Chapman P.L.. “Comparison of Photovoltaic Array Maximum Power Point Tracking Techniques”. IEEE Transactions on energy conversion, 2007 Vol.22, NO. 2, pp 439–449.

[18] Marei M.I., El-Sayad N., and El-Sattar A.A. “PV interface system with LVRT capability based on a current controlled HFAC link converter,” Sustainable Energy Technologies and Assessments, Vol. 9, 3 2015, pp. 55–62.

[19] El-helw H., Hassanien M., Ashour H. A., “Maximum power point tracking for irregular irradiance of a photovoltaic array”, 12th International Conference on Environment and Electrical

[20] Villalva M. G., Gazoli J. R. and Ruppert F E. “Analysis and simulation of the P&O MPPT algorithm using alinearized array model”. Power electronics conference, 2009, Brazil. pp 189–195.

[21] Femia N., Petrone G., Spagnuolo G., and Vitelli M., “Optimizing duty-cycle perturbation of P&O MPPT technique”. Power electronics specialists conference, 2004, Italy. Vol.3, pp 1939–1944.

[22] dos Santos W. M.,Martins D. C., “Dual Active Bridge converteras gyrator”, IEEE Third International Conference on Sustainable Energy Technologies (ICSET), 2012

[23] Marei M. I., El-Helw H. and Al-Hasheem M., “A grid-connected PV interface system based on the DAB-converter”, Environment and Electrical Engineering (EEEIC), 2015 IEEE 15th International Conference, Italy.pp161-165

