# Supplementary material for: Control Strategies for the DAB Based PV Interface System
Source: PLoS One. 2016 Aug 25;11(8):e0161856. doi: 10.1371/journal.pone.0161856 (PMC4999225; doi:10.1371/journal.pone.0161856)
Supplement: S1 Table — (DOCX) [file pone.0161856.s001.docx]

S1 Table The training data of the artificial neural network

| **Volt** | **Power** | **Irradiance** | **Phi** |
| --- | --- | --- | --- |
| 0.0256800000000000 | 0.306103500881718 | 1000 | 0.394725109858583 |
| 0.0513600000000000 | 0.612205674268447 | 1000 | 0.394724110214149 |
| 0.0770400000000000 | 0.918306520160156 | 1000 | 0.394723110570549 |
| 0.102720000000000 | 1.22440603855681 | 1000 | 0.394722110927784 |
| 0.128400000000000 | 1.53050422945839 | 1000 | 0.394721111285853 |
| 0.154080000000000 | 1.83660109286484 | 1000 | 0.394720111644756 |
| 0.179760000000000 | 2.14269662877615 | 1000 | 0.394719112004494 |
| 0.205440000000000 | 2.44879083724784 | 1000 | 0.394718112375524 |
| 0.231120000000000 | 2.75488371811320 | 1000 | 0.394717112726471 |
| 0.256800000000000 | 3.06097527153887 | 1000 | 0.394716113088711 |
| 0.282480000000000 | 3.36706549748626 | 1000 | 0.394715113454111 |
| 0.308160000000000 | 3.67315439590435 | 1000 | 0.394714113815692 |
| 0.333840000000000 | 3.97924196684409 | 1000 | 0.394713114180433 |
| 0.359520000000000 | 4.28532821024929 | 1000 | 0.394712114541796 |
| 0.385200000000000 | 4.59141312623740 | 1000 | 0.394711114912417 |
| 0.410880000000000 | 4.89749671469090 | 1000 | 0.394710115279660 |
| 0.436560000000000 | 5.20357897554085 | 1000 | 0.394709115638160 |
| 0.462240000000000 | 5.50965990911144 | 1000 | 0.394708116016645 |
| 0.487920000000000 | 5.81573951519093 | 1000 | 0.394707116395307 |
| 0.513600000000000 | 6.12181779354980 | 1000 | 0.394706116756964 |
| 0.539280000000000 | 6.42789474452557 | 1000 | 0.394705117128373 |
| 0.564960000000000 | 6.73397036805308 | 1000 | 0.394704117503860 |
| 0.590640000000000 | 7.04004466399012 | 1000 | 0.394703117873692 |
| 0.616320000000000 | 7.34611763247883 | 1000 | 0.394702118247600 |
| 0.642000000000000 | 7.65218927344526 | 1000 | 0.394701118620744 |
| 0.667680000000000 | 7.95825958696896 | 1000 | 0.394700118997916 |
| 0.693360000000000 | 8.26432857320599 | 1000 | 0.394699119387471 |
| 0.719040000000000 | 8.57039623136816 | 1000 | 0.394698119745776 |
| 0.744720000000000 | 8.87646256248534 | 1000 | 0.394697120129637 |
| 0.770400000000000 | 9.18252756616219 | 1000 | 0.394696120516735 |
| 0.796080000000001 | 9.48859124201665 | 1000 | 0.394695120888280 |
| 0.821760000000001 | 9.79465359053831 | 1000 | 0.394694121268851 |
| 0.847440000000001 | 10.1007146116484 | 1000 | 0.394693121654110 |
| 0.873120000000001 | 10.4067743048077 | 1000 | 0.394692122019830 |
| 0.898800000000001 | 10.7128326711269 | 1000 | 0.394691122415556 |
| 0.924480000000001 | 11.0188897096643 | 1000 | 0.394690122799465 |
| 0.950160000000001 | 11.3249454207052 | 1000 | 0.394689123184187 |
| 0.975840000000001 | 11.6309998042500 | 1000 | 0.394688123569751 |
| 1.00152000000000 | 11.9370528602101 | 1000 | 0.394687123952731 |
| 1.02720000000000 | 12.2431045888508 | 1000 | 0.394686124343374 |
| 1.05288000000000 | 12.5491549901198 | 1000 | 0.394685124739263 |
| 1.07856000000000 | 12.8552040632853 | 1000 | 0.394684125113840 |
| 1.10424000000000 | 13.1612518095293 | 1000 | 0.394683125510047 |
| 1.12992000000000 | 13.4672982282221 | 1000 | 0.394682125904921 |
| 1.15560000000000 | 13.7733433191660 | 1000 | 0.394681126291987 |
| 1.18128000000000 | 14.0793870827395 | 1000 | 0.394680126684204 |
| 1.20696000000000 | 14.3854295188520 | 1000 | 0.394679127078390 |
| 1.23264000000000 | 14.6914706270224 | 1000 | 0.394678127459383 |
| 1.25832000000000 | 14.9975104088347 | 1000 | 0.394677127876727 |
| 1.28400000000000 | 15.3035488620094 | 1000 | 0.394676128259633 |
| 1.30968000000000 | 15.6095859881578 | 1000 | 0.394675128657758 |
| 1.33536000000000 | 15.9156217870157 | 1000 | 0.394674129062618 |
| 1.36104000000000 | 16.2216562576935 | 1000 | 0.394673129448609 |
| 1.38672000000000 | 16.5276894014477 | 1000 | 0.394672129851862 |
| 1.41240000000000 | 16.8337212177224 | 1000 | 0.394671130256262 |
| 1.43808000000000 | 17.1397517061565 | 1000 | 0.394670130652086 |
| 1.46376000000000 | 17.4457808672654 | 1000 | 0.394669131053443 |
| 1.48944000000000 | 17.7518087009544 | 1000 | 0.394668131457633 |
| 1.51512000000000 | 18.0578352066389 | 1000 | 0.394667131849634 |
| 1.54080000000000 | 18.3638603860218 | 1000 | 0.394666132272837 |
| 1.56648000000000 | 18.6698842367104 | 1000 | 0.394665132666684 |
| 1.59216000000000 | 18.9759067598988 | 1000 | 0.394664133061636 |
| 1.61784000000000 | 19.2819279564798 | 1000 | 0.394663133479025 |
| 1.64352000000000 | 19.5879478251085 | 1000 | 0.394662133886194 |
| 1.66920000000000 | 19.8939663656814 | 1000 | 0.394661134281250 |
| 1.69488000000000 | 20.1999835800957 | 1000 | 0.394660134708079 |
| 1.72056000000000 | 20.5059994660107 | 1000 | 0.394659135112680 |
| 1.74624000000000 | 20.8120140252166 | 1000 | 0.394658135535718 |
| 1.77192000000000 | 21.1180272559157 | 1000 | 0.394657135937194 |
| 1.79760000000000 | 21.4240391591147 | 1000 | 0.394656136339727 |
| 1.82328000000000 | 21.7300497355822 | 1000 | 0.394655136759609 |
| 1.84896000000000 | 22.0360589842311 | 1000 | 0.394654137173414 |
| 1.87464000000000 | 22.3420669049578 | 1000 | 0.394653137579293 |
| 1.90032000000000 | 22.6480734994098 | 1000 | 0.394652138011127 |
| 1.92600000000000 | 22.9540787651913 | 1000 | 0.394651138419807 |
| 1.95168000000000 | 23.2600827033208 | 1000 | 0.394650138826459 |
| 1.97736000000000 | 23.5660853152850 | 1000 | 0.394649139260276 |
| 2.00304000000000 | 23.8720865984929 | 1000 | 0.394648139670218 |
| 2.02872000000000 | 24.1780865554657 | 1000 | 0.394647140105287 |
| 2.05440000000000 | 24.4840851836770 | 1000 | 0.394646140517013 |
| 2.08008000000000 | 24.7900824843883 | 1000 | 0.394645140929747 |
| 2.10576000000000 | 25.0960784583390 | 1000 | 0.394644141357064 |
| 2.13144000000000 | 25.4020731045107 | 1000 | 0.394643141779986 |
| 2.15712000000000 | 25.7080664231842 | 1000 | 0.394642142203737 |
| 2.18280000000000 | 26.0140584147930 | 1000 | 0.394641142635996 |
| 2.20848000000000 | 26.3200490780364 | 1000 | 0.394640143053722 |
| 2.23416000000000 | 26.6260384137254 | 1000 | 0.394639143471484 |
| 2.25984000000000 | 26.9320264228951 | 1000 | 0.394638143907020 |
| 2.28552000000000 | 27.2380131040768 | 1000 | 0.394637144334910 |
| 2.31120000000000 | 27.5439984577600 | 1000 | 0.394636144763628 |
| 2.33688000000000 | 27.8499824841776 | 1000 | 0.394635145197029 |
| 2.36256000000000 | 28.1559651823971 | 1000 | 0.394634145619724 |
| 2.38824000000000 | 28.4619465531172 | 1000 | 0.394633146043398 |
| 2.41392000000000 | 28.7679265975068 | 1000 | 0.394632146486776 |
| 2.43960000000000 | 29.0739053136967 | 1000 | 0.394631146919631 |
| 2.46528000000001 | 29.3798827023878 | 1000 | 0.394630147353314 |
| 2.49096000000000 | 29.6858587636084 | 1000 | 0.394629147788263 |
| 2.51664000000001 | 29.9918334981398 | 1000 | 0.394628148236472 |
| 2.54232000000000 | 30.2978069038637 | 1000 | 0.394627148665344 |
| 2.56800000000001 | 30.6037789820879 | 1000 | 0.394626149095178 |
| 2.59368000000001 | 30.9097497341258 | 1000 | 0.394625149545555 |
| 2.61936000000001 | 31.2157191573511 | 1000 | 0.394624149977118 |
| 2.64504000000001 | 31.5216872530758 | 1000 | 0.394623150409627 |
| 2.67072000000001 | 31.8276540226196 | 1000 | 0.394622150862184 |
| 2.69640000000001 | 32.1336194631530 | 1000 | 0.394621151293661 |
| 2.72208000000001 | 32.4395835768532 | 1000 | 0.394620151735610 |
| 2.74776000000001 | 32.7455463636186 | 1000 | 0.394619152186331 |
| 2.77344000000001 | 33.0515078217553 | 1000 | 0.394618152621985 |
| 2.79912000000001 | 33.3574679538969 | 1000 | 0.394617153079395 |
| 2.82480000000001 | 33.6634267571227 | 1000 | 0.394616153518006 |
| 2.85048000000001 | 33.9693842328478 | 1000 | 0.394615153957548 |
| 2.87616000000001 | 34.2753403824047 | 1000 | 0.394614154415935 |
| 2.90184000000001 | 34.5812952031298 | 1000 | 0.394613154857191 |
| 2.92752000000001 | 34.8872486964719 | 1000 | 0.394612155300930 |
| 2.95320000000001 | 35.1932008634138 | 1000 | 0.394611155759977 |
| 2.97888000000001 | 35.4991517016378 | 1000 | 0.394610156203869 |
| 3.00456000000001 | 35.8051012125832 | 1000 | 0.394609156651545 |
| 3.03024000000001 | 36.1110493955827 | 1000 | 0.394608157094411 |
| 3.05592000000001 | 36.4169962526465 | 1000 | 0.394607157558050 |
| 3.08160000000001 | 36.7229417808677 | 1000 | 0.394606158005485 |
| 3.10728000000001 | 37.0288859829328 | 1000 | 0.394605158470571 |
| 3.13296000000001 | 37.3348288561531 | 1000 | 0.394604158919710 |
| 3.15864000000001 | 37.6407704018722 | 1000 | 0.394603159369760 |
| 3.18432000000001 | 37.9467106200901 | 1000 | 0.394602159820719 |
| 3.21000000000001 | 38.2526495121574 | 1000 | 0.394601160288858 |
| 3.23568000000001 | 38.5585870759104 | 1000 | 0.394600160747928 |
| 3.26136000000001 | 38.8645233124423 | 1000 | 0.394599161211126 |
| 3.28704000000001 | 39.1704582208121 | 1000 | 0.394598161667303 |
| 3.31272000000001 | 39.4763918027257 | 1000 | 0.394597162136558 |
| 3.33840000000001 | 39.7823240559388 | 1000 | 0.394596162592610 |
| 3.36408000000001 | 40.0882549816503 | 1000 | 0.394595163049563 |
| 3.38976000000001 | 40.3941845812194 | 1000 | 0.394594163522923 |
| 3.41544000000001 | 40.7001128505683 | 1000 | 0.394593163966167 |
| 3.44112000000001 | 41.0060397951362 | 1000 | 0.394592164441115 |
| 3.46680000000001 | 41.3119654108413 | 1000 | 0.394591164901548 |
| 3.49248000000001 | 41.6178897003875 | 1000 | 0.394590165377747 |
| 3.51816000000001 | 41.9238126615799 | 1000 | 0.394589165845256 |
| 3.54384000000001 | 42.2297342956792 | 1000 | 0.394588166318043 |
| 3.56952000000001 | 42.5356546027465 | 1000 | 0.394587166796672 |
| 3.59520000000001 | 42.8415735809457 | 1000 | 0.394586167261287 |
| 3.62088000000001 | 43.1474912302731 | 1000 | 0.394585167712165 |
| 3.64656000000001 | 43.4534075548378 | 1000 | 0.394584168193191 |
| 3.67224000000001 | 43.7593225505298 | 1000 | 0.394583168660468 |
| 3.69792000000001 | 44.0652362198888 | 1000 | 0.394582169140860 |
| 3.72360000000001 | 44.3711485610696 | 1000 | 0.394581169614950 |
| 3.74928000000001 | 44.6770595753414 | 1000 | 0.394580170095975 |
| 3.77496000000001 | 44.9829692609260 | 1000 | 0.394579170565595 |
| 3.80064000000001 | 45.2888776192093 | 1000 | 0.394578171038161 |
| 3.82632000000001 | 45.5947846498901 | 1000 | 0.394577171510586 |
| 3.85200000000001 | 45.9006903544447 | 1000 | 0.394576171997710 |
| 3.87768000000001 | 46.2065947314998 | 1000 | 0.394575172485563 |
| 3.90336000000001 | 46.5124977796730 | 1000 | 0.394574172960452 |
| 3.92904000000001 | 46.8183995017238 | 1000 | 0.394573173449808 |
| 3.95472000000001 | 47.1242998935123 | 1000 | 0.394572173912882 |
| 3.98040000000001 | 47.4301989605591 | 1000 | 0.394571174403836 |
| 4.00608000000001 | 47.7360966987229 | 1000 | 0.394570174882174 |
| 4.03176000000001 | 48.0419931107662 | 1000 | 0.394569175374648 |
| 4.05744000000001 | 48.3478881925399 | 1000 | 0.394568175841463 |
| 4.08312000000001 | 48.6537819509642 | 1000 | 0.394567176348659 |
| 4.10880000000001 | 48.9596743791157 | 1000 | 0.394566176830334 |
| 4.13448000000001 | 49.2655654797653 | 1000 | 0.394565177312895 |
| 4.16016000000001 | 49.5714552542981 | 1000 | 0.394564177809219 |
| 4.18584000000001 | 49.8773436999416 | 1000 | 0.394563178293454 |
| 4.21152000000001 | 50.1832308194699 | 1000 | 0.394562178791306 |
| 4.23720000000001 | 50.4891166101071 | 1000 | 0.394561179277213 |
| 4.26288000000001 | 50.7950010732392 | 1000 | 0.394560179763977 |
| 4.28856000000001 | 51.1008842074758 | 1000 | 0.394559180239060 |
| 4.31424000000001 | 51.4067660183864 | 1000 | 0.394558180752609 |
| 4.33992000000001 | 51.7126464990080 | 1000 | 0.394557181241912 |
| 4.36560000000001 | 52.0185256521270 | 1000 | 0.394556181732095 |
| 4.39128000000001 | 52.3244034784578 | 1000 | 0.394555182229447 |
| 4.41696000000001 | 52.6302799772473 | 1000 | 0.394554182727265 |
| 4.44264000000001 | 52.9361551492498 | 1000 | 0.394553183232122 |
| 4.46832000000001 | 53.2420289909555 | 1000 | 0.394552183713537 |
| 4.49400000000001 | 53.5479015079523 | 1000 | 0.394551184220007 |
| 4.51968000000001 | 53.8537726960464 | 1000 | 0.394550184715240 |
| 4.54536000000001 | 54.1596425575571 | 1000 | 0.394549185219171 |
| 4.57104000000001 | 54.4655110925221 | 1000 | 0.394548185731988 |
| 4.59672000000001 | 54.7713782967051 | 1000 | 0.394547186217916 |
| 4.62240000000001 | 55.0772441747817 | 1000 | 0.394546186716535 |
| 4.64808000000001 | 55.3831087267554 | 1000 | 0.394545187227676 |
| 4.67376000000001 | 55.6889719498247 | 1000 | 0.394544187727968 |
| 4.69944000000001 | 55.9948338467907 | 1000 | 0.394543188240653 |
| 4.72512000000001 | 56.3006944148508 | 1000 | 0.394542188742605 |
| 4.75080000000001 | 56.6065536554044 | 1000 | 0.394541189245404 |
| 4.77648000000001 | 56.9124115684544 | 1000 | 0.394540189749072 |
| 4.80216000000001 | 57.2182681554034 | 1000 | 0.394539190264904 |
| 4.82784000000001 | 57.5241234148483 | 1000 | 0.394538190781481 |
| 4.85352000000001 | 57.8299773467906 | 1000 | 0.394537191298816 |
| 4.87920000000001 | 58.1358299484125 | 1000 | 0.394536191794580 |
| 4.90488000000001 | 58.4416812253451 | 1000 | 0.394535192313518 |
| 4.93056000000001 | 58.7475311733633 | 1000 | 0.394534192822146 |
| 4.95624000000001 | 59.0533797938773 | 1000 | 0.394533193331639 |
| 4.98192000000001 | 59.3592270897036 | 1000 | 0.394532193863859 |
| 5.00760000000001 | 59.6650730552059 | 1000 | 0.394531194374945 |
| 5.03328000000001 | 59.9709176932036 | 1000 | 0.394530194886894 |
| 5.05896000000001 | 60.2767610036940 | 1000 | 0.394529195399686 |
| 5.08464000000001 | 60.5826029866798 | 1000 | 0.394528195913340 |
| 5.11032000000001 | 60.8884436421580 | 1000 | 0.394527196427835 |
| 5.13600000000001 | 61.1942829701314 | 1000 | 0.394526196943190 |
| 5.16168000000001 | 61.5001209720107 | 1000 | 0.394525197469976 |
| 5.18736000000001 | 61.8059576449706 | 1000 | 0.394524197986972 |
| 5.21304000000001 | 62.1117929918401 | 1000 | 0.394523198515322 |
| 5.23872000000001 | 62.4176270112027 | 1000 | 0.394522199044414 |
| 5.26440000000001 | 62.7234597002297 | 1000 | 0.394521199553474 |
| 5.29008000000001 | 63.0292910645799 | 1000 | 0.394520200084169 |
| 5.31576000000001 | 63.3351211000091 | 1000 | 0.394519200605323 |
| 5.34144000000001 | 63.6409498107644 | 1000 | 0.394518201147833 |
| 5.36712000000001 | 63.9467771897616 | 1000 | 0.394517201660366 |
| 5.39280000000001 | 64.2526032426703 | 1000 | 0.394516202184014 |
| 5.41848000000001 | 64.5584279694892 | 1000 | 0.394515202718623 |
| 5.44416000000001 | 64.8642513673840 | 1000 | 0.394514203243918 |
| 5.46984000000001 | 65.1700734391898 | 1000 | 0.394513203780084 |
| 5.49552000000001 | 65.4758941820703 | 1000 | 0.394512204307025 |
| 5.52120000000001 | 65.7817135974419 | 1000 | 0.394511204834800 |
| 5.54688000000001 | 66.0875316867270 | 1000 | 0.394510205373326 |
| 5.57256000000001 | 66.3933484470852 | 1000 | 0.394509205902755 |
| 5.59824000000001 | 66.6991638813562 | 1000 | 0.394508206442840 |
| 5.62392000000001 | 67.0049779866992 | 1000 | 0.394507206973914 |
| 5.64960000000001 | 67.3107907645327 | 1000 | 0.394506207505819 |
| 5.67528000000001 | 67.6166022148596 | 1000 | 0.394505208038576 |
| 5.70096000000001 | 67.9224123391007 | 1000 | 0.394504208581823 |
| 5.72664000000001 | 68.2282211344102 | 1000 | 0.394503209116211 |
| 5.75232000000001 | 68.5340286022127 | 1000 | 0.394502209651451 |
| 5.77800000000001 | 68.8398347439306 | 1000 | 0.394501210197058 |
| 5.80368000000001 | 69.1456395567166 | 1000 | 0.394500210733937 |
| 5.82936000000001 | 69.4514430419924 | 1000 | 0.394499211271646 |
| 5.85504000000001 | 69.7572452011875 | 1000 | 0.394498211819624 |
| 5.88072000000001 | 70.0630460328731 | 1000 | 0.394497212368354 |
| 5.90640000000001 | 70.3688455341974 | 1000 | 0.394496212899167 |
| 5.93208000000001 | 70.6746437108659 | 1000 | 0.394495213449497 |
| 5.95776000000001 | 70.9804405585978 | 1000 | 0.394494213991320 |
| 5.98344000000001 | 71.2862360802506 | 1000 | 0.394493214543222 |
| 6.00912000000001 | 71.5920302743934 | 1000 | 0.394492215095877 |
| 6.03480000000001 | 71.8978231367408 | 1000 | 0.394491215621829 |
| 6.06048000000001 | 72.2036146744352 | 1000 | 0.394490216166997 |
| 6.08616000000001 | 72.5094048874817 | 1000 | 0.394489216731182 |
| 6.11184000000001 | 72.8151937701575 | 1000 | 0.394488217277949 |
| 6.13752000000001 | 73.1209813267544 | 1000 | 0.394487217834566 |
| 6.16320000000001 | 73.4267675544122 | 1000 | 0.394486218382975 |
| 6.18888000000001 | 73.7325524559902 | 1000 | 0.394485218941155 |
| 6.21456000000002 | 74.0383360286282 | 1000 | 0.394484219491198 |
| 6.24024000000001 | 74.3441182737544 | 1000 | 0.394483220042064 |
| 6.26592000000001 | 74.6498991928045 | 1000 | 0.394482220602616 |
| 6.29160000000001 | 74.9556787814769 | 1000 | 0.394481221146306 |
| 6.31728000000001 | 75.2614570455053 | 1000 | 0.394480221708445 |
| 6.34296000000002 | 75.5672339805911 | 1000 | 0.394479222262617 |
| 6.36864000000001 | 75.8730095910334 | 1000 | 0.394478222835029 |
| 6.39432000000001 | 76.1787838710982 | 1000 | 0.394477223390797 |
| 6.42000000000002 | 76.4845568222154 | 1000 | 0.394476223938745 |
| 6.44568000000001 | 76.7903284486931 | 1000 | 0.394475224504818 |
| 6.47136000000002 | 77.0960987462230 | 1000 | 0.394474225063070 |
| 6.49704000000001 | 77.4018677176777 | 1000 | 0.394473225630699 |
| 6.52272000000002 | 77.7076353601865 | 1000 | 0.394472226190587 |
| 6.54840000000002 | 78.0134016751823 | 1000 | 0.394471226751295 |
| 6.57408000000002 | 78.3191666626679 | 1000 | 0.394470227312840 |
| 6.59976000000002 | 78.6249303255152 | 1000 | 0.394469227892050 |
| 6.62544000000002 | 78.9306926565398 | 1000 | 0.394468228446799 |
| 6.65112000000002 | 79.2364536614884 | 1000 | 0.394467229010789 |
| 6.67680000000002 | 79.5422133403633 | 1000 | 0.394466229583938 |
| 6.70248000000002 | 79.8479716917281 | 1000 | 0.394465230157862 |
| 6.72816000000002 | 80.1537287127013 | 1000 | 0.394464230716002 |
| 6.75384000000002 | 80.4594844076023 | 1000 | 0.394463231283278 |
| 6.77952000000002 | 80.7652387764291 | 1000 | 0.394462231859583 |
| 6.80520000000002 | 81.0709918177452 | 1000 | 0.394461232436664 |
| 6.83088000000002 | 81.3767435301071 | 1000 | 0.394460233006350 |
| 6.85656000000002 | 81.6824939163968 | 1000 | 0.394459233584986 |
| 6.88224000000002 | 81.9882429751755 | 1000 | 0.394458234164398 |
| 6.90792000000002 | 82.2939907049987 | 1000 | 0.394457234736501 |
| 6.93360000000002 | 82.5997371073099 | 1000 | 0.394456235309436 |
| 6.95928000000002 | 82.9054821835483 | 1000 | 0.394455235891200 |
| 6.98496000000002 | 83.2112259308328 | 1000 | 0.394454236465755 |
| 7.01064000000002 | 83.5169683506020 | 1000 | 0.394453237041125 |
| 7.03632000000002 | 83.8227094428575 | 1000 | 0.394452237617319 |
| 7.06200000000002 | 84.1284492090436 | 1000 | 0.394451238202245 |
| 7.08768000000002 | 84.4341876462712 | 1000 | 0.394450238780055 |
| 7.11336000000002 | 84.7399247559861 | 1000 | 0.394449239358696 |
| 7.13904000000002 | 85.0456605381851 | 1000 | 0.394448239938150 |
| 7.16472000000002 | 85.3513949928710 | 1000 | 0.394447240518433 |
| 7.19040000000002 | 85.6571281214857 | 1000 | 0.394446241107298 |
| 7.21608000000002 | 85.9628599211411 | 1000 | 0.394445241689188 |
| 7.24176000000002 | 86.2685903961737 | 1000 | 0.394444242287340 |
| 7.26744000000002 | 86.5743195407998 | 1000 | 0.394443242870819 |
| 7.29312000000002 | 86.8800473564664 | 1000 | 0.394442243447459 |
| 7.31880000000002 | 87.1857738475079 | 1000 | 0.394441244040243 |
| 7.34448000000002 | 87.4914990095897 | 1000 | 0.394440244626187 |
| 7.37016000000002 | 87.7972228441544 | 1000 | 0.394439245212942 |
| 7.39584000000002 | 88.1029453526504 | 1000 | 0.394438245808080 |
| 7.42152000000002 | 88.4086665321854 | 1000 | 0.394437246396455 |
| 7.44720000000002 | 88.7143863856505 | 1000 | 0.394436246993154 |
| 7.47288000000002 | 89.0201049116015 | 1000 | 0.394435247590630 |
| 7.49856000000002 | 89.3258221071393 | 1000 | 0.394434248173933 |
| 7.52424000000002 | 89.6315379795066 | 1000 | 0.394433248780436 |
| 7.54992000000002 | 89.9372525200112 | 1000 | 0.394432249365396 |
| 7.57560000000002 | 90.2429657373452 | 1000 | 0.394431249973406 |
| 7.60128000000002 | 90.5486776228161 | 1000 | 0.394430250560022 |
| 7.62696000000002 | 90.8543881822219 | 1000 | 0.394429251154863 |
| 7.65264000000002 | 91.1600974155574 | 1000 | 0.394428251757829 |
| 7.67832000000002 | 91.4658053199263 | 1000 | 0.394427252354263 |
| 7.70400000000002 | 91.7715118967779 | 1000 | 0.394426252951510 |
| 7.72968000000002 | 92.0772171461119 | 1000 | 0.394425253549572 |
| 7.75536000000002 | 92.3829210679313 | 1000 | 0.394424254148463 |
| 7.78104000000002 | 92.6886236636809 | 1000 | 0.394423254755362 |
| 7.80672000000002 | 92.9943249319130 | 1000 | 0.394422255363029 |
| 7.83240000000002 | 93.3000248711762 | 1000 | 0.394421255964299 |
| 7.85808000000002 | 93.6057234829241 | 1000 | 0.394420256566397 |
| 7.88376000000002 | 93.9114207700545 | 1000 | 0.394419257183533 |
| 7.90944000000002 | 94.2171167253109 | 1000 | 0.394418257780098 |
| 7.93512000000002 | 94.5228113545007 | 1000 | 0.394417258384595 |
| 7.96080000000002 | 94.8285046576242 | 1000 | 0.394416258996958 |
| 7.98648000000002 | 95.1341966317793 | 1000 | 0.394415259603070 |
| 8.01216000000002 | 95.4398872798654 | 1000 | 0.394414260216991 |
| 8.03784000000002 | 95.7455765975261 | 1000 | 0.394413260817697 |
| 8.06352000000002 | 96.0512645905741 | 1000 | 0.394412261433196 |
| 8.08920000000002 | 96.3569512546491 | 1000 | 0.394411262042513 |
| 8.11488000000002 | 96.6626365926614 | 1000 | 0.394410262659582 |
| 8.14056000000002 | 96.9683206031514 | 1000 | 0.394409263277405 |
| 8.16624000000002 | 97.2740032832127 | 1000 | 0.394408263882221 |
| 8.19192000000002 | 97.5796846386629 | 1000 | 0.394407264501620 |
| 8.21760000000002 | 97.8853646665961 | 1000 | 0.394406265121799 |
| 8.24328000000002 | 98.1910433655512 | 1000 | 0.394405265735908 |
| 8.26896000000002 | 98.4967207369856 | 1000 | 0.394404266350823 |
| 8.29464000000002 | 98.8023967808991 | 1000 | 0.394403266966546 |
| 8.32032000000002 | 99.1080714987476 | 1000 | 0.394402267589841 |
| 8.34600000000002 | 99.4137448876221 | 1000 | 0.394401268207171 |
| 8.37168000000002 | 99.7194169504287 | 1000 | 0.394400268832020 |
| 8.39736000000002 | 100.025087684258 | 1000 | 0.394399269450929 |
| 8.42304000000002 | 100.330757090565 | 1000 | 0.394398270070644 |
| 8.44872000000002 | 100.636425170811 | 1000 | 0.394397270697845 |
| 8.47440000000002 | 100.942091920618 | 1000 | 0.394396271312503 |
| 8.50008000000002 | 101.247757347274 | 1000 | 0.394395271947892 |
| 8.52576000000002 | 101.553421442036 | 1000 | 0.394394272564178 |
| 8.55144000000002 | 101.859084212191 | 1000 | 0.394393273194489 |
| 8.57712000000002 | 102.164745653368 | 1000 | 0.394392273819007 |
| 8.60280000000002 | 102.470405767020 | 1000 | 0.394391274444314 |
| 8.62848000000002 | 102.776064556065 | 1000 | 0.394390275083496 |
| 8.65416000000002 | 103.081722014671 | 1000 | 0.394389275710373 |
| 8.67984000000002 | 103.387378144295 | 1000 | 0.394388276331554 |
| 8.70552000000002 | 103.693032949316 | 1000 | 0.394387276966547 |
| 8.73120000000002 | 103.998686425352 | 1000 | 0.394386277595831 |
| 8.75688000000002 | 104.304338575323 | 1000 | 0.394385278232360 |
| 8.78256000000002 | 104.609989396310 | 1000 | 0.394384278863228 |
| 8.80824000000002 | 104.915638889777 | 1000 | 0.394383279494911 |
| 8.83392000000002 | 105.221287057176 | 1000 | 0.394382280133771 |
| 8.85960000000002 | 105.526933897051 | 1000 | 0.394381280773396 |
| 8.88528000000002 | 105.832579406481 | 1000 | 0.394380281401077 |
| 8.91096000000002 | 106.138223591307 | 1000 | 0.394379282042266 |
| 8.93664000000002 | 106.443866445690 | 1000 | 0.394378282671593 |
| 8.96232000000002 | 106.749507975465 | 1000 | 0.394377283314347 |
| 8.98800000000002 | 107.055148179177 | 1000 | 0.394376283964149 |
| 9.01368000000002 | 107.360787050980 | 1000 | 0.394375284595877 |
| 9.03936000000002 | 107.666424596718 | 1000 | 0.394374285234688 |
| 9.06504000000002 | 107.972060814934 | 1000 | 0.394373285874310 |
| 9.09072000000002 | 108.277695708543 | 1000 | 0.394372286527151 |
| 9.11640000000002 | 108.583329270241 | 1000 | 0.394371287162120 |
| 9.14208000000002 | 108.888961508799 | 1000 | 0.394370287816472 |
| 9.16776000000002 | 109.194592413984 | 1000 | 0.394369288446895 |
| 9.19344000000002 | 109.500221996026 | 1000 | 0.394368289096622 |
| 9.21912000000002 | 109.805850252004 | 1000 | 0.394367289753247 |
| 9.24480000000002 | 110.111477176067 | 1000 | 0.394366290392246 |
| 9.27048000000002 | 110.417102774065 | 1000 | 0.394365291038175 |
| 9.29616000000002 | 110.722727047465 | 1000 | 0.394364291697083 |
| 9.32184000000002 | 111.028349988944 | 1000 | 0.394363292338499 |
| 9.34752000000002 | 111.333971604358 | 1000 | 0.394362292986795 |
| 9.37320000000002 | 111.639591892244 | 1000 | 0.394361293635885 |
| 9.39888000000002 | 111.945210854065 | 1000 | 0.394360294291791 |
| 9.42456000000002 | 112.250828486897 | 1000 | 0.394359294942464 |
| 9.45024000000002 | 112.556444792197 | 1000 | 0.394358295593918 |
| 9.47592000000002 | 112.862059772897 | 1000 | 0.394357296258115 |
| 9.50160000000002 | 113.167673421674 | 1000 | 0.394356296905162 |
| 9.52728000000002 | 113.473285745854 | 1000 | 0.394355297564933 |
| 9.55296000000002 | 113.778896742501 | 1000 | 0.394354298225453 |
| 9.57864000000002 | 114.084506410154 | 1000 | 0.394353298880819 |
| 9.60432000000002 | 114.390114750277 | 1000 | 0.394352299536976 |
| 9.63000000000002 | 114.695721762869 | 1000 | 0.394351300193923 |
| 9.65568000000002 | 115.001327449399 | 1000 | 0.394350300857542 |
| 9.68136000000002 | 115.306931806930 | 1000 | 0.394349301516054 |
| 9.70704000000002 | 115.612534838397 | 1000 | 0.394348302181195 |
| 9.73272000000002 | 115.918136540866 | 1000 | 0.394347302841271 |
| 9.75840000000002 | 116.223736915803 | 1000 | 0.394346303502136 |
| 9.78408000000002 | 116.529335963212 | 1000 | 0.394345304163801 |
| 9.80976000000002 | 116.834933684553 | 1000 | 0.394344304832023 |
| 9.83544000000002 | 117.140530076896 | 1000 | 0.394343305495238 |
| 9.86112000000002 | 117.446125143173 | 1000 | 0.394342306164991 |
| 9.88680000000002 | 117.751718880453 | 1000 | 0.394341306829778 |
| 9.91248000000002 | 118.057311290198 | 1000 | 0.394340307495339 |
| 9.93816000000002 | 118.362902373878 | 1000 | 0.394339308167396 |
| 9.96384000000002 | 118.668492128557 | 1000 | 0.394338308834515 |
| 9.98952000000003 | 118.974080557171 | 1000 | 0.394337309508101 |
| 10.0152000000000 | 119.279667655318 | 1000 | 0.394336310171123 |
| 10.0408800000000 | 119.585253428865 | 1000 | 0.394335310846251 |
| 10.0665600000000 | 119.890837873409 | 1000 | 0.394334311516497 |
| 10.0922400000000 | 120.196420990419 | 1000 | 0.394333312187528 |
| 10.1179200000000 | 120.502002782832 | 1000 | 0.394332312870565 |
| 10.1436000000000 | 120.807583243308 | 1000 | 0.394331313537549 |
| 10.1692800000000 | 121.113162379183 | 1000 | 0.394330314216501 |
| 10.1949600000000 | 121.418740187523 | 1000 | 0.394329314896208 |
| 10.2206400000000 | 121.724316666859 | 1000 | 0.394328315571114 |
| 10.2463200000000 | 122.029891817193 | 1000 | 0.394327316241269 |
| 10.2720000000000 | 122.335465644396 | 1000 | 0.394326316928814 |
| 10.2976800000000 | 122.641038141124 | 1000 | 0.394325317606052 |
| 10.3233600000000 | 122.946609310316 | 1000 | 0.394324318284071 |
| 10.3490400000000 | 123.252179151972 | 1000 | 0.394323318962871 |
| 10.3747200000000 | 123.557747666093 | 1000 | 0.394322319642462 |
| 10.4004000000000 | 123.863314854145 | 1000 | 0.394321320328287 |
| 10.4260800000000 | 124.168880713189 | 1000 | 0.394320321009414 |
| 10.4517600000000 | 124.474445246166 | 1000 | 0.394319321696759 |
| 10.4774400000000 | 124.780008450136 | 1000 | 0.394318322379431 |
| 10.5031200000000 | 125.085570325099 | 1000 | 0.394317323057479 |
| 10.5288000000000 | 125.391130875462 | 1000 | 0.394316323747121 |
| 10.5544800000000 | 125.696690099759 | 1000 | 0.394315324442905 |
| 10.5801600000000 | 126.002247992103 | 1000 | 0.394314325123288 |
| 10.6058400000000 | 126.307804559854 | 1000 | 0.394313325815210 |
| 10.6315200000000 | 126.613359797120 | 1000 | 0.394312326497172 |
| 10.6572000000000 | 126.918913708318 | 1000 | 0.394311327185273 |
| 10.6828800000000 | 127.224466293447 | 1000 | 0.394310327879476 |
| 10.7085600000000 | 127.530017549565 | 1000 | 0.394309328569117 |
| 10.7342400000000 | 127.835567479618 | 1000 | 0.394308329264845 |
| 10.7599200000000 | 128.141116080655 | 1000 | 0.394307329956023 |
| 10.7856000000000 | 128.446663355624 | 1000 | 0.394306330653253 |
| 10.8112800000000 | 128.752209301580 | 1000 | 0.394305331345967 |
| 10.8369600000000 | 129.057753919994 | 1000 | 0.394304332039456 |
| 10.8626400000000 | 129.363297212342 | 1000 | 0.394303332738969 |
| 10.8883200000000 | 129.668839175673 | 1000 | 0.394302333433991 |
| 10.9140000000000 | 129.974379812935 | 1000 | 0.394301334135004 |
| 10.9396800000000 | 130.279919121181 | 1000 | 0.394300334831559 |
| 10.9653600000000 | 130.585457100415 | 1000 | 0.394299335523703 |
| 10.9910400000000 | 130.890993756522 | 1000 | 0.394298336232178 |
| 11.0167200000000 | 131.196529082140 | 1000 | 0.394297336931036 |
| 11.0424000000000 | 131.502063078740 | 1000 | 0.394296337625505 |
| 11.0680800000000 | 131.807595752218 | 1000 | 0.394295338336210 |
| 11.0937600000000 | 132.113127095208 | 1000 | 0.394294339037378 |
| 11.1194400000000 | 132.418657110650 | 1000 | 0.394293339739304 |
| 11.1451200000000 | 132.724185798548 | 1000 | 0.394292340442000 |
| 11.1708000000000 | 133.029713160375 | 1000 | 0.394291341150566 |
| 11.1964800000000 | 133.335239194657 | 1000 | 0.394290341859878 |
| 11.2221600000000 | 133.640763899922 | 1000 | 0.394289342564866 |
| 11.2478400000000 | 133.946287279114 | 1000 | 0.394288343275680 |
| 11.2735200000000 | 134.251809327810 | 1000 | 0.394287343977123 |
| 11.2992000000000 | 134.557330051910 | 1000 | 0.394286344689449 |
| 11.3248800000000 | 134.862849446991 | 1000 | 0.394285345397493 |
| 11.3505600000000 | 135.168367514522 | 1000 | 0.394284346106293 |
| 11.3762400000000 | 135.473884255982 | 1000 | 0.394283346820872 |
| 11.4019200000000 | 135.779399666943 | 1000 | 0.394282347526189 |
| 11.4276000000000 | 136.084913751832 | 1000 | 0.394281348237284 |
| 11.4532800000000 | 136.390426512127 | 1000 | 0.394280348959115 |
| 11.4789600000000 | 136.695937940445 | 1000 | 0.394279349666747 |
| 11.5046400000000 | 137.001448044165 | 1000 | 0.394278350385082 |
| 11.5303200000000 | 137.306956820336 | 1000 | 0.394277351104159 |
| 11.5560000000000 | 137.612464266007 | 1000 | 0.394276351814101 |
| 11.5816800000000 | 137.917970387082 | 1000 | 0.394275352534691 |
| 11.6073600000000 | 138.223475180606 | 1000 | 0.394274353256012 |
| 11.6330400000000 | 138.528978645103 | 1000 | 0.394273353973167 |
| 11.6587200000000 | 138.834480782050 | 1000 | 0.394272354691083 |
| 11.6844000000000 | 139.139981591449 | 1000 | 0.394271355409769 |
| 11.7100800000000 | 139.445481074770 | 1000 | 0.394270356134081 |
| 11.7357600000000 | 139.750979229064 | 1000 | 0.394269356854267 |
| 11.7614400000000 | 140.056476055806 | 1000 | 0.394268357575212 |
| 11.7871200000000 | 140.361971554997 | 1000 | 0.394267358296916 |
| 11.8128000000000 | 140.667465728115 | 1000 | 0.394266359024223 |
| 11.8384799999999 | 140.972958573677 | 1000 | 0.394265359752257 |
| 11.8641599999999 | 141.278450088733 | 1000 | 0.394264360471400 |
| 11.8898399999999 | 141.583940279189 | 1000 | 0.394263361200930 |
| 11.9155199999999 | 141.889429142093 | 1000 | 0.394262361931195 |
| 11.9411999999999 | 142.194916675968 | 1000 | 0.394261362657423 |
| 11.9668799999999 | 142.500402882286 | 1000 | 0.394260363384396 |
| 11.9925599999999 | 142.805887762527 | 1000 | 0.394259364116890 |
| 12.0182399999999 | 143.111371315214 | 1000 | 0.394258364850118 |
| 12.0439199999999 | 143.416853537392 | 1000 | 0.394257365574602 |
| 12.0695999999999 | 143.722334434969 | 1000 | 0.394256366309320 |
| 12.0952799999999 | 144.027814002033 | 1000 | 0.394255367035321 |
| 12.1209599999999 | 144.333292243019 | 1000 | 0.394254367766811 |
| 12.1466399999999 | 144.638769159405 | 1000 | 0.394253368508465 |
| 12.1723199999999 | 144.944244745281 | 1000 | 0.394252369241450 |
| 12.1979999999999 | 145.249719003597 | 1000 | 0.394251369975177 |
| 12.2236799999999 | 145.555191935834 | 1000 | 0.394250370714333 |
| 12.2493599999999 | 145.860663539035 | 1000 | 0.394249371449553 |
| 12.2750399999999 | 146.166133816156 | 1000 | 0.394248372190182 |
| 12.3007199999999 | 146.471602764243 | 1000 | 0.394247372926903 |
| 12.3263999999999 | 146.777070386247 | 1000 | 0.394246373669004 |
| 12.3520799999999 | 147.082536676254 | 1000 | 0.394245374397943 |
| 12.3777599999999 | 147.388001643140 | 1000 | 0.394244375141532 |
| 12.4034399999999 | 147.693465283948 | 1000 | 0.394243375890472 |
| 12.4291199999999 | 147.998927592755 | 1000 | 0.394242376626306 |
| 12.4547999999999 | 148.304388575480 | 1000 | 0.394241377367499 |
| 12.4804799999999 | 148.609848230645 | 1000 | 0.394240378109439 |
| 12.5061599999999 | 148.915306559728 | 1000 | 0.394239378856700 |
| 12.5318399999999 | 149.220763559772 | 1000 | 0.394238379600132 |
| 12.5575199999999 | 149.526219233732 | 1000 | 0.394237380348857 |
| 12.5831999999999 | 149.831673578649 | 1000 | 0.394236381093761 |
| 12.6088799999999 | 150.137126596003 | 1000 | 0.394235381839410 |
| 12.6345599999999 | 150.442578287275 | 1000 | 0.394234382590333 |
| 12.6602399999999 | 150.748028649505 | 1000 | 0.394233383337470 |
| 12.6859199999999 | 151.053477684169 | 1000 | 0.394232384085340 |
| 12.7115999999999 | 151.358925391268 | 1000 | 0.394231384833953 |
| 12.7372799999999 | 151.664371770803 | 1000 | 0.394230385583309 |
| 12.7629599999999 | 151.969816825738 | 1000 | 0.394229386342387 |
| 12.7886399999999 | 152.275260550142 | 1000 | 0.394228387093207 |
| 12.8143199999999 | 152.580702945500 | 1000 | 0.394227387840300 |
| 12.8399999999999 | 152.886144014773 | 1000 | 0.394226388592610 |
| 12.8656799999999 | 153.191583757960 | 1000 | 0.394225389350111 |
| 12.8913599999999 | 153.497022173583 | 1000 | 0.394224390108343 |
| 12.9170399999999 | 153.802459260155 | 1000 | 0.394223390862853 |
| 12.9427199999999 | 154.107895020641 | 1000 | 0.394222391622528 |
| 12.9683999999999 | 154.413329452077 | 1000 | 0.394221392378505 |
| 12.9940799999999 | 154.718762554463 | 1000 | 0.394220393130812 |
| 13.0197599999999 | 155.024194335211 | 1000 | 0.394219393901482 |
| 13.0454399999999 | 155.329624780976 | 1000 | 0.394218394650869 |
| 13.0711199999999 | 155.635053903618 | 1000 | 0.394217395414176 |
| 13.0967999999999 | 155.940481698690 | 1000 | 0.394216396178202 |
| 13.1224799999999 | 156.245908163230 | 1000 | 0.394215396934221 |
| 13.1481599999999 | 156.551333303161 | 1000 | 0.394214397699699 |
| 13.1738399999999 | 156.856757115521 | 1000 | 0.394213398465895 |
| 13.1995199999999 | 157.162179597344 | 1000 | 0.394212399224122 |
| 13.2251999999999 | 157.467600754560 | 1000 | 0.394211399991767 |
| 13.2508799999999 | 157.773020582724 | 1000 | 0.394210400755811 |
| 13.2765599999999 | 158.078439084795 | 1000 | 0.394209401524895 |
| 13.3022399999999 | 158.383856257810 | 1000 | 0.394208402290384 |
| 13.3279199999999 | 158.689272103251 | 1000 | 0.394207403056604 |
| 13.3535999999999 | 158.994686622601 | 1000 | 0.394206403827849 |
| 13.3792799999999 | 159.300099812896 | 1000 | 0.394205404595528 |
| 13.4049599999999 | 159.605511675614 | 1000 | 0.394204405363929 |
| 13.4306399999999 | 159.910922212239 | 1000 | 0.394203406137328 |
| 13.4563199999999 | 160.216331421288 | 1000 | 0.394202406911439 |
| 13.4819999999999 | 160.521739298314 | 1000 | 0.394201407673509 |
| 13.5076799999999 | 160.827145853694 | 1000 | 0.394200408453312 |
| 13.5333599999999 | 161.132551078530 | 1000 | 0.394199409225334 |
| 13.5590399999999 | 161.437954975789 | 1000 | 0.394198409998082 |
| 13.5847199999999 | 161.743357545469 | 1000 | 0.394197410771555 |
| 13.6103999999999 | 162.048758790541 | 1000 | 0.394196411554192 |
| 13.6360799999999 | 162.354158703580 | 1000 | 0.394195412324891 |
| 13.6617599999999 | 162.659557290524 | 1000 | 0.394194413100530 |
| 13.6874399999999 | 162.964954551372 | 1000 | 0.394193413881083 |
| 13.7131199999999 | 163.270350483155 | 1000 | 0.394192414658159 |
| 13.7387999999999 | 163.575745088845 | 1000 | 0.394191415440142 |
| 13.7644799999999 | 163.881138363982 | 1000 | 0.394190416214485 |
| 13.7901599999999 | 164.186530315992 | 1000 | 0.394189417002049 |
| 13.8158399999999 | 164.491920937450 | 1000 | 0.394188417781995 |
| 13.8415199999999 | 164.797310231328 | 1000 | 0.394187418562669 |
| 13.8671999999999 | 165.102698197620 | 1000 | 0.394186419344054 |
| 13.8928799999999 | 165.408084836329 | 1000 | 0.394185420126157 |
| 13.9185599999999 | 165.713470148939 | 1000 | 0.394184420913103 |
| 13.9442399999999 | 166.018854130995 | 1000 | 0.394183421692518 |
| 13.9699199999999 | 166.324236789924 | 1000 | 0.394182422485002 |
| 13.9955999999999 | 166.629618116809 | 1000 | 0.394181423265857 |
| 14.0212799999999 | 166.934998119079 | 1000 | 0.394180424055633 |
| 14.0469599999999 | 167.240376792277 | 1000 | 0.394179424842022 |
| 14.0726399999999 | 167.545754137889 | 1000 | 0.394178425629125 |
| 14.0983199999999 | 167.851130157402 | 1000 | 0.394177426421024 |
| 14.1239999999999 | 168.156504849324 | 1000 | 0.394176427213615 |
| 14.1496799999999 | 168.461878210687 | 1000 | 0.394175427998785 |
| 14.1753599999999 | 168.767250245946 | 1000 | 0.394174428788734 |
| 14.2010399999999 | 169.072620955105 | 1000 | 0.394173429583447 |
| 14.2267199999999 | 169.377990335186 | 1000 | 0.394172430374812 |
| 14.2523999999999 | 169.683358387677 | 1000 | 0.394171431166887 |
| 14.2780799999999 | 169.988725114063 | 1000 | 0.394170431963696 |
| 14.3037599999999 | 170.294090512858 | 1000 | 0.394169432761200 |
| 14.3294399999998 | 170.599454582578 | 1000 | 0.394168433555397 |
| 14.3551199999998 | 170.904817326189 | 1000 | 0.394167434354298 |
| 14.3807999999998 | 171.210178740721 | 1000 | 0.394166435149896 |
| 14.4064799999998 | 171.515538826173 | 1000 | 0.394165435942212 |
| 14.4321599999998 | 171.820897587005 | 1000 | 0.394164436743212 |
| 14.4578399999998 | 172.126255017272 | 1000 | 0.394163437536962 |
| 14.4835199999998 | 172.431611124402 | 1000 | 0.394162438343327 |
| 14.5091999999998 | 172.736965900963 | 1000 | 0.394161439142447 |
| 14.5348799999998 | 173.042319349928 | 1000 | 0.394160439942270 |
| 14.5605599999998 | 173.347671472786 | 1000 | 0.394159440746753 |
| 14.5862399999998 | 173.653022265071 | 1000 | 0.394158441544035 |
| 14.6119199999998 | 173.958371732733 | 1000 | 0.394157442349900 |
| 14.6375999999998 | 174.263719871309 | 1000 | 0.394156443152527 |
| 14.6632799999998 | 174.569066683774 | 1000 | 0.394155443959774 |
| 14.6889599999998 | 174.874412168644 | 1000 | 0.394154444767717 |
| 14.7146399999998 | 175.179756324423 | 1000 | 0.394153445572430 |
| 14.7403199999998 | 175.485099151115 | 1000 | 0.394152446373941 |
| 14.7659999999998 | 175.790440651694 | 1000 | 0.394151447180058 |
| 14.7916799999998 | 176.095780826162 | 1000 | 0.394150447990767 |
| 14.8173599999998 | 176.401119671538 | 1000 | 0.394149448798272 |
| 14.8430399999998 | 176.706457190799 | 1000 | 0.394148449610347 |
| 14.8687199999998 | 177.011793380970 | 1000 | 0.394147450419237 |
| 14.8943999999998 | 177.317128245025 | 1000 | 0.394146451232684 |
| 14.9200799999998 | 177.622461778504 | 1000 | 0.394145452039109 |
| 14.9457599999998 | 177.927793988839 | 1000 | 0.394144452857779 |
| 14.9714399999998 | 178.233124867104 | 1000 | 0.394143453665590 |
| 14.9971199999998 | 178.538454420741 | 1000 | 0.394142454481777 |
| 15.0227999999998 | 178.843782645283 | 1000 | 0.394141455294814 |
| 15.0484799999998 | 179.149109542221 | 1000 | 0.394140456108550 |
| 15.0741599999998 | 179.454435111548 | 1000 | 0.394139456922968 |
| 15.0998399999998 | 179.759759354756 | 1000 | 0.394138457741887 |
| 15.1255199999998 | 180.065082268868 | 1000 | 0.394137458557677 |
| 15.1511999999998 | 180.370403855373 | 1000 | 0.394136459374164 |
| 15.1768799999998 | 180.675724114265 | 1000 | 0.394135460191330 |
| 15.2025599999998 | 180.981043047035 | 1000 | 0.394134461012969 |
| 15.2282399999998 | 181.286360650706 | 1000 | 0.394133461831502 |
| 15.2539199999998 | 181.591676928255 | 1000 | 0.394132462654494 |
| 15.2795999999998 | 181.896991876705 | 1000 | 0.394131463474400 |
| 15.3052799999998 | 182.202305499029 | 1000 | 0.394130464298742 |
| 15.3309599999998 | 182.507617790761 | 1000 | 0.394129465116247 |
| 15.3566399999998 | 182.812928757858 | 1000 | 0.394128465941943 |
| 15.3823199999998 | 183.118238395851 | 1000 | 0.394127466764567 |
| 15.4079999999998 | 183.423546706232 | 1000 | 0.394126467587881 |
| 15.4336799999998 | 183.728853691972 | 1000 | 0.394125468419327 |
| 15.4593599999998 | 184.034159345628 | 1000 | 0.394124469240257 |
| 15.4850399999998 | 184.339463673157 | 1000 | 0.394123470065596 |
| 15.5107199999998 | 184.644766674560 | 1000 | 0.394122470895334 |
| 15.5363999999998 | 184.950068345363 | 1000 | 0.394121471718317 |
| 15.5620799999998 | 185.255368691527 | 1000 | 0.394120472549390 |
| 15.5877599999998 | 185.560667710071 | 1000 | 0.394119473381128 |
| 15.6134399999998 | 185.865965399508 | 1000 | 0.394118474209848 |
| 15.6391199999998 | 186.171261762808 | 1000 | 0.394117475042911 |
| 15.6647999999998 | 186.476556797002 | 1000 | 0.394116475872974 |
| 15.6904799999998 | 186.781850503575 | 1000 | 0.394115476703711 |
| 15.7161599999998 | 187.087142882519 | 1000 | 0.394114477535106 |
| 15.7418399999998 | 187.392433933845 | 1000 | 0.394113478367188 |
| 15.7675199999998 | 187.697723657541 | 1000 | 0.394112479199926 |
| 15.7931999999998 | 188.003012055109 | 1000 | 0.394111480036997 |
| 15.8188799999998 | 188.308299123561 | 1000 | 0.394110480871085 |
| 15.8445599999998 | 188.613584864381 | 1000 | 0.394109481705826 |
| 15.8702399999998 | 188.918869280561 | 1000 | 0.394108482548512 |
| 15.8959199999998 | 189.224152363146 | 1000 | 0.394107483377329 |
| 15.9215999999998 | 189.529434124071 | 1000 | 0.394106484221325 |
| 15.9472799999998 | 189.834714554386 | 1000 | 0.394105485058739 |
| 15.9729599999998 | 190.139993657066 | 1000 | 0.394104485896803 |
| 15.9986399999998 | 190.445271432121 | 1000 | 0.394103486735546 |
| 16.0243199999998 | 190.750547881031 | 1000 | 0.394102487578534 |
| 16.0499999999998 | 191.055823000824 | 1000 | 0.394101488418599 |
| 16.0756799999998 | 191.361096792985 | 1000 | 0.394100489259324 |
| 16.1013599999998 | 191.666369257507 | 1000 | 0.394099490100697 |
| 16.1270399999998 | 191.971640394401 | 1000 | 0.394098490942745 |
| 16.1527199999998 | 192.276910205146 | 1000 | 0.394097491789007 |
| 16.1783999999998 | 192.582178688262 | 1000 | 0.394096492635931 |
| 16.2040799999998 | 192.887445842252 | 1000 | 0.394095493479944 |
| 16.2297599999998 | 193.192711667107 | 1000 | 0.394094494321049 |
| 16.2554399999998 | 193.497976167314 | 1000 | 0.394093495169929 |
| 16.2811199999998 | 193.803239338386 | 1000 | 0.394092496015898 |
| 16.3067999999998 | 194.108501183316 | 1000 | 0.394091496866072 |
| 16.3324799999998 | 194.413761699116 | 1000 | 0.394090497713359 |
| 16.3581599999998 | 194.719020887270 | 1000 | 0.394089498561286 |
| 16.3838399999998 | 195.024278749280 | 1000 | 0.394088499413398 |
| 16.4095199999998 | 195.329535282158 | 1000 | 0.394087500262638 |
| 16.4351999999998 | 195.634790485895 | 1000 | 0.394086501109005 |
| 16.4608799999998 | 195.940044363487 | 1000 | 0.394085501959551 |
| 16.4865599999998 | 196.245296916413 | 1000 | 0.394084502817728 |
| 16.5122399999998 | 196.550548138716 | 1000 | 0.394083503669561 |
| 16.5379199999998 | 196.855798033374 | 1000 | 0.394082504522041 |
| 16.5635999999998 | 197.161046601873 | 1000 | 0.394081505378636 |
| 16.5892799999998 | 197.466293841238 | 1000 | 0.394080506232404 |
| 16.6149599999998 | 197.771539752950 | 1000 | 0.394079507086803 |
| 16.6406399999998 | 198.076784337019 | 1000 | 0.394078507941860 |
| 16.6663199999998 | 198.382027594931 | 1000 | 0.394077508801022 |
| 16.6919999999998 | 198.687269522203 | 1000 | 0.394076509653891 |
| 16.7176799999998 | 198.992510126308 | 1000 | 0.394075510517776 |
| 16.7433599999998 | 199.297749396785 | 1000 | 0.394074511368486 |
| 16.7690399999997 | 199.602987345585 | 1000 | 0.394073512233633 |
| 16.7947199999998 | 199.908223963748 | 1000 | 0.394072513092527 |
| 16.8203999999997 | 200.213459254251 | 1000 | 0.394071513952045 |
| 16.8460799999997 | 200.518693217105 | 1000 | 0.394070514812215 |
| 16.8717599999997 | 200.823925853790 | 1000 | 0.394069515676427 |
| 16.8974399999997 | 201.129157161332 | 1000 | 0.394068516537864 |
| 16.9231199999997 | 201.434387142710 | 1000 | 0.394067517403346 |
| 16.9487999999997 | 201.739615793438 | 1000 | 0.394066518262627 |
| 16.9744799999997 | 202.044843118007 | 1000 | 0.394065519125965 |
| 17.0001599999997 | 202.350069117896 | 1000 | 0.394064519996711 |
| 17.0258399999997 | 202.655293787144 | 1000 | 0.394063520861303 |
| 17.0515199999997 | 202.960517130225 | 1000 | 0.394062521729909 |
| 17.0771999999997 | 203.265739144144 | 1000 | 0.394061522595741 |
| 17.1028799999997 | 203.570959830406 | 1000 | 0.394060523462215 |
| 17.1285599999997 | 203.876179189004 | 1000 | 0.394059524329317 |
| 17.1542399999997 | 204.181397219932 | 1000 | 0.394058525197032 |
| 17.1799199999997 | 204.486613923201 | 1000 | 0.394057526065386 |
| 17.2055999999997 | 204.791829298797 | 1000 | 0.394056526934351 |
| 17.2312799999997 | 205.097043348225 | 1000 | 0.394055527807305 |
| 17.2569599999997 | 205.402256066998 | 1000 | 0.394054528674180 |
| 17.2826399999997 | 205.707467461084 | 1000 | 0.394053529548355 |
| 17.3083199999997 | 206.012677527506 | 1000 | 0.394052530423154 |
| 17.3339999999997 | 206.317886264757 | 1000 | 0.394051531295221 |
| 17.3596799999997 | 206.623093675837 | 1000 | 0.394050532171244 |
| 17.3853599999997 | 206.928299754763 | 1000 | 0.394049533037913 |
| 17.4110399999997 | 207.233504510491 | 1000 | 0.394048533915154 |
| 17.4367199999997 | 207.538707938551 | 1000 | 0.394047534793016 |
| 17.4623999999997 | 207.843910037436 | 1000 | 0.394046535668163 |
| 17.4880799999997 | 208.149110807156 | 1000 | 0.394045536540636 |
| 17.5137599999997 | 208.454310252188 | 1000 | 0.394044537420326 |
| 17.5394399999997 | 208.759508369537 | 1000 | 0.394043538300606 |
| 17.5651199999997 | 209.064705157718 | 1000 | 0.394042539178214 |
| 17.5907999999997 | 209.369900619708 | 1000 | 0.394041540059702 |
| 17.6164799999997 | 209.675094751035 | 1000 | 0.394040540935246 |
| 17.6421599999997 | 209.980287557669 | 1000 | 0.394039541817956 |
| 17.6678399999997 | 210.285479035120 | 1000 | 0.394038542697982 |
| 17.6935199999997 | 210.590669186388 | 1000 | 0.394037543581892 |
| 17.7191999999997 | 210.895858008472 | 1000 | 0.394036544463125 |
| 17.7448799999997 | 211.201045502876 | 1000 | 0.394035545344976 |
| 17.7705599999997 | 211.506231669594 | 1000 | 0.394034546227430 |
| 17.7962399999997 | 211.811416510114 | 1000 | 0.394033547113720 |
| 17.8219199999997 | 212.116600021456 | 1000 | 0.394032547997374 |
| 17.8475999999997 | 212.421782205110 | 1000 | 0.394031548881629 |
| 17.8732799999997 | 212.726963061067 | 1000 | 0.394030549766469 |
| 17.8989599999997 | 213.032142590835 | 1000 | 0.394029550655150 |
| 17.9246399999997 | 213.337320789915 | 1000 | 0.394028551537958 |
| 17.9503199999997 | 213.642497662802 | 1000 | 0.394027552424604 |
| 17.9759999999997 | 213.947673209491 | 1000 | 0.394026553315062 |
| 18.0016799999997 | 214.252847426983 | 1000 | 0.394025554202879 |
| 18.0273599999997 | 214.558020316786 | 1000 | 0.394024555091303 |
| 18.0530399999997 | 214.863191881876 | 1000 | 0.394023555986710 |
| 18.0787199999997 | 215.168362116284 | 1000 | 0.394022556876309 |
| 18.1043999999997 | 215.473531021497 | 1000 | 0.394021557763305 |
| 18.1300799999997 | 215.778698600500 | 1000 | 0.394020558654075 |
| 18.1557599999997 | 216.083864854798 | 1000 | 0.394019559551813 |
| 18.1814399999997 | 216.389029776902 | 1000 | 0.394018560440570 |
| 18.2071199999997 | 216.694193374301 | 1000 | 0.394017561336283 |
| 18.2327999999997 | 216.999355642499 | 1000 | 0.394016562229403 |
| 18.2584799999997 | 217.304516584482 | 1000 | 0.394015563126259 |
| 18.2841599999997 | 217.609676195774 | 1000 | 0.394014564017379 |
| 18.3098399999997 | 217.914834480848 | 1000 | 0.394013564912233 |
| 18.3355199999997 | 218.219991439715 | 1000 | 0.394012565810834 |
| 18.3611999999997 | 218.525147069377 | 1000 | 0.394011566706856 |
| 18.3868799999997 | 218.830301372819 | 1000 | 0.394010567606589 |
| 18.4125599999997 | 219.135454345564 | 1000 | 0.394009568500622 |
| 18.4382399999997 | 219.440605992086 | 1000 | 0.394008569398363 |
| 18.4639199999997 | 219.745756312396 | 1000 | 0.394007570299824 |
| 18.4895999999997 | 220.050905304992 | 1000 | 0.394006571201849 |
| 18.5152799999997 | 220.356052968370 | 1000 | 0.394005572101303 |
| 18.5409599999997 | 220.661199302541 | 1000 | 0.394004572998222 |
| 18.5666399999997 | 220.966344311986 | 1000 | 0.394003573901950 |
| 18.5923199999997 | 221.271487993706 | 1000 | 0.394002574806225 |
| 18.6179999999997 | 221.576630344719 | 1000 | 0.394001575704860 |
| 18.6436799999997 | 221.881771369502 | 1000 | 0.394000576607161 |
| 18.6693599999997 | 222.186911068064 | 1000 | 0.393999577513137 |
| 18.6950399999997 | 222.492049437407 | 1000 | 0.393998578416576 |
| 18.7207199999997 | 222.797186480515 | 1000 | 0.393997579323655 |
| 18.7463999999997 | 223.102322192910 | 1000 | 0.393996580225130 |
| 18.7720799999997 | 223.407456579069 | 1000 | 0.393995581130243 |
| 18.7977599999997 | 223.712589639002 | 1000 | 0.393994582039006 |
| 18.8234399999997 | 224.017721371207 | 1000 | 0.393993582948320 |
| 18.8491199999997 | 224.322851774178 | 1000 | 0.393992583855101 |
| 18.8747999999997 | 224.627980849424 | 1000 | 0.393991584762451 |
| 18.9004799999997 | 224.933108595434 | 1000 | 0.393990585667283 |
| 18.9261599999997 | 225.238235016709 | 1000 | 0.393989586578803 |
| 18.9518399999997 | 225.543360108753 | 1000 | 0.393988587487816 |
| 18.9775199999997 | 225.848483874552 | 1000 | 0.393987588400416 |
| 19.0031999999997 | 226.153606311122 | 1000 | 0.393986589310526 |
| 19.0288799999997 | 226.458727419947 | 1000 | 0.393985590221173 |
| 19.0545599999997 | 226.763847201039 | 1000 | 0.393984591132380 |
| 19.0802399999997 | 227.068965655886 | 1000 | 0.393983592047166 |
| 19.1059199999997 | 227.374082782986 | 1000 | 0.393982592962478 |
| 19.1315999999997 | 227.679198579353 | 1000 | 0.393981593872289 |
| 19.1572799999997 | 227.984313050965 | 1000 | 0.393980594788680 |
| 19.1829599999997 | 228.289426191842 | 1000 | 0.393979595699583 |
| 19.2086399999997 | 228.594538006468 | 1000 | 0.393978596614048 |
| 19.2343199999996 | 228.899648494838 | 1000 | 0.393977597532049 |
| 19.2599999999996 | 229.204757655464 | 1000 | 0.393976598450593 |
| 19.2856799999996 | 229.509865486842 | 1000 | 0.393975599366662 |
| 19.3113599999996 | 229.814971990462 | 1000 | 0.393974600283256 |
| 19.3370399999996 | 230.120077167832 | 1000 | 0.393973601203390 |
| 19.3627199999996 | 230.425181014446 | 1000 | 0.393972602118058 |
| 19.3883999999996 | 230.730283537802 | 1000 | 0.393971603042234 |
| 19.4140799999996 | 231.035384728909 | 1000 | 0.393970603957979 |
| 19.4397599999996 | 231.340484593749 | 1000 | 0.393969604877227 |
| 19.4654399999996 | 231.645583130835 | 1000 | 0.393968605797015 |
| 19.4911199999996 | 231.950680338655 | 1000 | 0.393967606714348 |
| 19.5167999999996 | 232.255776221715 | 1000 | 0.393966607638157 |
| 19.5424799999996 | 232.560870775514 | 1000 | 0.393965608559520 |
| 19.5681599999996 | 232.865964004537 | 1000 | 0.393964609487311 |
| 19.5938399999996 | 233.171055902804 | 1000 | 0.393963610409710 |
| 19.6195199999996 | 233.476146471799 | 1000 | 0.393962611329666 |
| 19.6451999999996 | 233.781235716027 | 1000 | 0.393961612256054 |
| 19.6708799999996 | 234.086323629489 | 1000 | 0.393960613177065 |
| 19.6965599999996 | 234.391410219667 | 1000 | 0.393959614107406 |
| 19.7222399999996 | 234.696495479082 | 1000 | 0.393958615032387 |
| 19.7479199999996 | 235.001579409218 | 1000 | 0.393957615954936 |
| 19.7735999999996 | 235.306662014580 | 1000 | 0.393956616883874 |
| 19.7992799999996 | 235.611743290667 | 1000 | 0.393955617810389 |
| 19.8249599999996 | 235.916823238970 | 1000 | 0.393954618737402 |
| 19.8506399999996 | 236.221901859498 | 1000 | 0.393953619664934 |
| 19.8763199999996 | 236.526979153738 | 1000 | 0.393952620595874 |
| 19.9019999999996 | 236.832055118702 | 1000 | 0.393951621524413 |
| 19.9276799999996 | 237.137129755882 | 1000 | 0.393950622453456 |
| 19.9533599999996 | 237.442203063772 | 1000 | 0.393949623380086 |
| 19.9790399999996 | 237.747275048377 | 1000 | 0.393948624315932 |
| 20.0047199999996 | 238.052345702196 | 1000 | 0.393947625246473 |
| 20.0303999999996 | 238.357415026720 | 1000 | 0.393946626174608 |
| 20.0560799999996 | 238.662483024958 | 1000 | 0.393945627106146 |
| 20.0817599999996 | 238.967549696895 | 1000 | 0.393944628041051 |
| 20.1074399999996 | 239.272615039546 | 1000 | 0.393943628973574 |
| 20.1331199999996 | 239.577679055900 | 1000 | 0.393942629909466 |
| 20.1587999999996 | 239.882741742951 | 1000 | 0.393941630842955 |
| 20.1844799999996 | 240.187803102210 | 1000 | 0.393940631776946 |
| 20.2101599999996 | 240.492863135162 | 1000 | 0.393939632714279 |
| 20.2358399999996 | 240.797921837321 | 1000 | 0.393938633646376 |
| 20.2615199999996 | 241.102979214674 | 1000 | 0.393937634584684 |
| 20.2871999999996 | 241.408035262717 | 1000 | 0.393936635520601 |
| 20.3128799999996 | 241.713089981462 | 1000 | 0.393935636454159 |
| 20.3385599999996 | 242.018143375391 | 1000 | 0.393934637393890 |
| 20.3642399999996 | 242.323195440017 | 1000 | 0.393933638331259 |
| 20.3899199999996 | 242.628246178332 | 1000 | 0.393932639271947 |
| 20.4155999999996 | 242.933295587330 | 1000 | 0.393931640210254 |
| 20.4412799999996 | 243.238343670019 | 1000 | 0.393930641151881 |
| 20.4669599999996 | 243.543390423388 | 1000 | 0.393929642091131 |
| 20.4926399999996 | 243.848435847446 | 1000 | 0.393928643028036 |
| 20.5183199999996 | 244.153479946685 | 1000 | 0.393927643971066 |
| 20.5439999999996 | 244.458522715099 | 1000 | 0.393926644908905 |
| 20.5696799999996 | 244.763564158696 | 1000 | 0.393925645852861 |
| 20.5953599999996 | 245.068604272964 | 1000 | 0.393924646794452 |
| 20.6210399999996 | 245.373643057914 | 1000 | 0.393923647733706 |
| 20.6467199999996 | 245.678680518037 | 1000 | 0.393922648679044 |
| 20.6723999999996 | 245.983716648826 | 1000 | 0.393921649622020 |
| 20.6980799999996 | 246.288751453291 | 1000 | 0.393920650568266 |
| 20.7237599999996 | 246.593784928419 | 1000 | 0.393919651512154 |
| 20.7494399999996 | 246.898817075721 | 1000 | 0.393918652456509 |
| 20.7751199999996 | 247.203847896687 | 1000 | 0.393917653404108 |
| 20.8007999999996 | 247.508877386812 | 1000 | 0.393916654346568 |
| 20.8264799999996 | 247.813905552105 | 1000 | 0.393915655295064 |
| 20.8521599999996 | 248.118932388058 | 1000 | 0.393914656241221 |
| 20.8778399999996 | 248.423957896164 | 1000 | 0.393913657187815 |
| 20.9035199999996 | 248.728982076433 | 1000 | 0.393912658134866 |
| 20.9291999999996 | 249.034004928852 | 1000 | 0.393911659082350 |
| 20.9548799999996 | 249.339026454931 | 1000 | 0.393910660033055 |
| 20.9805599999996 | 249.644046651661 | 1000 | 0.393909660981430 |
| 21.0062399999996 | 249.949065520536 | 1000 | 0.393908661930234 |
| 21.0319199999996 | 250.254083060065 | 1000 | 0.393907662876729 |
| 21.0575999999996 | 250.559099274736 | 1000 | 0.393906663829163 |
| 21.0832799999996 | 250.864114160058 | 1000 | 0.393905664779286 |
| 21.1089599999996 | 251.169127719023 | 1000 | 0.393904665732588 |
| 21.1346399999996 | 251.474139947123 | 1000 | 0.393903666680816 |
| 21.1603199999996 | 251.779150850370 | 1000 | 0.393902667634971 |
| 21.1859999999996 | 252.084160425749 | 1000 | 0.393901668589537 |
| 21.2116799999996 | 252.389168670270 | 1000 | 0.393900669539065 |
| 21.2373599999996 | 252.694175588426 | 1000 | 0.393899670491754 |
| 21.2630399999996 | 252.999181180209 | 1000 | 0.393898671447582 |
| 21.2887199999996 | 253.304185442628 | 1000 | 0.393897672401112 |
| 21.3143999999996 | 253.609188377171 | 1000 | 0.393896673355049 |
| 21.3400799999996 | 253.914189983847 | 1000 | 0.393895674309414 |
| 21.3657599999996 | 254.219190262648 | 1000 | 0.393894675264195 |
| 21.3914399999996 | 254.524189215068 | 1000 | 0.393893676222089 |
| 21.4171199999996 | 254.829186836615 | 1000 | 0.393892677174984 |
| 21.4427999999996 | 255.134183131776 | 1000 | 0.393891678130990 |
| 21.4684799999996 | 255.439178099061 | 1000 | 0.393890679087415 |
| 21.4941599999996 | 255.744171739963 | 1000 | 0.393889680046945 |
| 21.5198399999996 | 256.049164049973 | 1000 | 0.393888681001472 |
| 21.5455199999996 | 256.354155035102 | 1000 | 0.393887681961803 |
| 21.5711999999996 | 256.659144690841 | 1000 | 0.393886682919840 |
| 21.5968799999996 | 256.964133017182 | 1000 | 0.393885683875580 |
| 21.6225599999996 | 257.269120020138 | 1000 | 0.393884684839783 |
| 21.6482399999996 | 257.574105690691 | 1000 | 0.393883685796319 |
| 21.6739199999995 | 257.879090036354 | 1000 | 0.393882686758620 |
| 21.6995999999995 | 258.184073052617 | 1000 | 0.393881687718632 |
| 21.7252799999995 | 258.489054740974 | 1000 | 0.393880688679025 |
| 21.7509599999995 | 258.794035102935 | 1000 | 0.393879689642483 |
| 21.7766399999995 | 259.099014133982 | 1000 | 0.393878690600983 |
| 21.8023199999995 | 259.403991838630 | 1000 | 0.393877691562546 |
| 21.8279999999995 | 259.708968216870 | 1000 | 0.393876692527150 |
| 21.8536799999995 | 260.013943265693 | 1000 | 0.393875693489461 |
| 21.8793599999995 | 260.318916985107 | 1000 | 0.393874694449511 |
| 21.9050399999995 | 260.623889378101 | 1000 | 0.393873695412583 |
| 21.9307199999995 | 260.928860444687 | 1000 | 0.393872696378686 |
| 21.9563999999995 | 261.233830183353 | 1000 | 0.393871697345156 |
| 21.9820799999995 | 261.538798592591 | 1000 | 0.393870698309342 |
| 22.0077599999995 | 261.843765673912 | 1000 | 0.393869699273907 |
| 22.0334399999995 | 262.148731427302 | 1000 | 0.393868700238829 |
| 22.0591199999995 | 262.453695852770 | 1000 | 0.393867701204127 |
| 22.0847999999995 | 262.758658950310 | 1000 | 0.393866702169789 |
| 22.1104799999995 | 263.063620721414 | 1000 | 0.393865703138427 |
| 22.1361599999995 | 263.368581161587 | 1000 | 0.393864704102185 |
| 22.1618399999995 | 263.673540273819 | 1000 | 0.393863705066297 |
| 22.1875199999995 | 263.978498061123 | 1000 | 0.393862706036012 |
| 22.2131999999995 | 264.283454520486 | 1000 | 0.393861707006075 |
| 22.2388799999995 | 264.588409648899 | 1000 | 0.393860707971253 |
| 22.2645599999995 | 264.893363452377 | 1000 | 0.393859708942012 |
| 22.2902399999995 | 265.198315927909 | 1000 | 0.393858709913112 |
| 22.3159199999995 | 265.503267070981 | 1000 | 0.393857710876738 |
| 22.3415999999995 | 265.808216890614 | 1000 | 0.393856711848525 |
| 22.3672799999995 | 266.113165382288 | 1000 | 0.393855712820636 |
| 22.3929599999995 | 266.418112543010 | 1000 | 0.393854713787908 |
| 22.4186399999995 | 266.723058380280 | 1000 | 0.393853714763290 |
| 22.4443199999995 | 267.028002883578 | 1000 | 0.393852715728641 |
| 22.4699999999995 | 267.332946063425 | 1000 | 0.393851716702098 |
| 22.4956799999995 | 267.637887915303 | 1000 | 0.393850717675868 |
| 22.5213599999995 | 267.942828434715 | 1000 | 0.393849718642240 |
| 22.5470399999995 | 268.247767630664 | 1000 | 0.393848719616675 |
| 22.5727199999995 | 268.552705495633 | 1000 | 0.393847720586275 |
| 22.5983999999995 | 268.857642035639 | 1000 | 0.393846721561352 |
| 22.6240799999995 | 269.162577246162 | 1000 | 0.393845722534167 |
| 22.6497599999995 | 269.467511130215 | 1000 | 0.393844723509875 |
| 22.6754399999995 | 269.772443683285 | 1000 | 0.393843724480772 |
| 22.7011199999995 | 270.077374911371 | 1000 | 0.393842725457094 |
| 22.7267999999995 | 270.382304809976 | 1000 | 0.393841726431179 |
| 22.7524799999995 | 270.687233380588 | 1000 | 0.393840727405563 |
| 22.7781599999995 | 270.992160623217 | 1000 | 0.393839728380265 |
| 22.8038399999995 | 271.297086536353 | 1000 | 0.393838729352726 |
| 22.8295199999995 | 271.602011124493 | 1000 | 0.393837730330573 |
| 22.8551999999995 | 271.906934383141 | 1000 | 0.393836731306185 |
| 22.8808799999995 | 272.211856312281 | 1000 | 0.393835732279548 |
| 22.9065599999995 | 272.516776917934 | 1000 | 0.393834733260829 |
| 22.9322399999995 | 272.821696191076 | 1000 | 0.393833734234797 |
| 22.9579199999995 | 273.126614137708 | 1000 | 0.393832735211581 |
| 22.9835999999995 | 273.431530757841 | 1000 | 0.393831736191193 |
| 23.0092799999995 | 273.736446046958 | 1000 | 0.393830737166039 |
| 23.0349599999995 | 274.041360011061 | 1000 | 0.393829738146208 |
| 23.0606399999995 | 274.346272645651 | 1000 | 0.393828739124147 |
| 23.0863199999995 | 274.651183950714 | 1000 | 0.393827740099841 |
| 23.1119999999995 | 274.956093930768 | 1000 | 0.393826741080856 |
| 23.1376799999995 | 275.261002581296 | 1000 | 0.393825742059630 |
| 23.1633599999995 | 275.565909903792 | 1000 | 0.393824743038669 |
| 23.1890399999995 | 275.870815899769 | 1000 | 0.393823744020494 |
| 23.2147199999995 | 276.175720564703 | 1000 | 0.393822744997570 |
| 23.2403999999995 | 276.480623903113 | 1000 | 0.393821745977427 |
| 23.2660799999995 | 276.785525913486 | 1000 | 0.393820746957549 |
| 23.2917599999995 | 277.090426595815 | 1000 | 0.393819747937923 |
| 23.3174399999995 | 277.395325950109 | 1000 | 0.393818748918568 |
| 23.3431199999995 | 277.700223976353 | 1000 | 0.393817749899460 |
| 23.3687999999995 | 278.005120676060 | 1000 | 0.393816750883103 |
| 23.3944799999995 | 278.310016046214 | 1000 | 0.393815751864511 |
| 23.4201599999995 | 278.614910088311 | 1000 | 0.393814752846159 |
| 23.4458399999995 | 278.919802802360 | 1000 | 0.393813753828065 |
| 23.4715199999995 | 279.224694188345 | 1000 | 0.393812754810207 |
| 23.4971999999995 | 279.529584246277 | 1000 | 0.393811755792602 |
| 23.5228799999995 | 279.834472974643 | 1000 | 0.393810756772768 |
| 23.5485599999995 | 280.139360376441 | 1000 | 0.393809757755635 |
| 23.5742399999995 | 280.444246451681 | 1000 | 0.393808758741212 |
| 23.5999199999995 | 280.749131197341 | 1000 | 0.393807759724546 |
| 23.6255999999995 | 281.054014614934 | 1000 | 0.393806760708120 |
| 23.6512799999995 | 281.358896704450 | 1000 | 0.393805761691923 |
| 23.6769599999995 | 281.663777465882 | 1000 | 0.393804762675941 |
| 23.7026399999995 | 281.968656900742 | 1000 | 0.393803763662645 |
| 23.7283199999995 | 282.273535004510 | 1000 | 0.393802764644667 |
| 23.7539999999995 | 282.578411781689 | 1000 | 0.393801765629348 |
| 23.7796799999995 | 282.883287230784 | 1000 | 0.393800766614255 |
| 23.8053599999995 | 283.188161351779 | 1000 | 0.393799767599366 |
| 23.8310399999995 | 283.493034146189 | 1000 | 0.393798768587135 |
| 23.8567199999995 | 283.797905610997 | 1000 | 0.393797769572672 |
| 23.8823999999995 | 284.102775747697 | 1000 | 0.393796770558404 |
| 23.9080799999995 | 284.407644554795 | 1000 | 0.393795771541919 |
| 23.9337599999995 | 284.712512035284 | 1000 | 0.393794772528057 |
| 23.9594399999995 | 285.017378187668 | 1000 | 0.393793773514403 |
| 23.9851199999995 | 285.322243011940 | 1000 | 0.393792774500944 |
| 24.0107999999995 | 285.627106509593 | 1000 | 0.393791775490088 |
| 24.0364799999995 | 285.931968677630 | 1000 | 0.393790776477009 |
| 24.0621599999995 | 286.236829516035 | 1000 | 0.393789777461692 |
| 24.0878399999995 | 286.541689029329 | 1000 | 0.393788778451399 |
| 24.1135199999994 | 286.846547212991 | 1000 | 0.393787779438874 |
| 24.1391999999994 | 287.151404067012 | 1000 | 0.393786780424110 |
| 24.1648799999994 | 287.456259595913 | 1000 | 0.393785781414347 |
| 24.1905599999994 | 287.761113793663 | 1000 | 0.393784782399939 |
| 24.2162399999994 | 288.065966666287 | 1000 | 0.393783783390516 |
| 24.2419199999994 | 288.370818209265 | 1000 | 0.393782784378859 |
| 24.2675999999994 | 288.675668422586 | 1000 | 0.393781785364963 |
| 24.2932799999994 | 288.980517309268 | 1000 | 0.393780786353637 |
| 24.3189599999994 | 289.285364869295 | 1000 | 0.393779787344851 |
| 24.3446399999994 | 289.590211098165 | 1000 | 0.393778788331452 |
| 24.3703199999994 | 289.895056001885 | 1000 | 0.393777789322983 |
| 24.3959999999994 | 290.199899574429 | 1000 | 0.393776790309885 |
| 24.4216799999994 | 290.504741821822 | 1000 | 0.393775791301712 |
| 24.4473599999994 | 290.809582739537 | 1000 | 0.393774792291292 |
| 24.4730399999994 | 291.114422327582 | 1000 | 0.393773793278649 |
| 24.4987199999994 | 291.419260590461 | 1000 | 0.393772794270898 |
| 24.5243999999994 | 291.724097522148 | 1000 | 0.393771795258529 |
| 24.5500799999994 | 292.028933130174 | 1000 | 0.393770796253413 |
| 24.5757599999994 | 292.333767405502 | 1000 | 0.393769797241322 |
| 24.6014399999994 | 292.638600352636 | 1000 | 0.393768798229356 |
| 24.6271199999994 | 292.943431974595 | 1000 | 0.393767799222251 |
| 24.6527999999994 | 293.248262265345 | 1000 | 0.393766800210539 |
| 24.6784799999994 | 293.553091230912 | 1000 | 0.393765801203673 |
| 24.7041599999994 | 293.857918866774 | 1000 | 0.393764802194565 |
| 24.7298399999994 | 294.162745172922 | 1000 | 0.393763803183212 |
| 24.7555199999994 | 294.467570153877 | 1000 | 0.393762804176681 |
| 24.7811999999994 | 294.772393805111 | 1000 | 0.393761805167899 |
| 24.8068799999994 | 295.077216128137 | 1000 | 0.393760806159231 |
| 24.8325599999994 | 295.382037121441 | 1000 | 0.393759807148324 |
| 24.8582399999994 | 295.686856788022 | 1000 | 0.393758808139851 |
| 24.8839199999994 | 295.991675126386 | 1000 | 0.393757809131483 |
| 24.9095999999994 | 296.296492138019 | 1000 | 0.393756810125534 |
| 24.9352799999994 | 296.601307818419 | 1000 | 0.393755811115016 |
| 24.9609599999994 | 296.906122172088 | 1000 | 0.393754812106920 |
| 24.9866399999994 | 297.210935199018 | 1000 | 0.393753813101226 |
| 25.0123199999994 | 297.515746894702 | 1000 | 0.393752814090967 |
| 25.0379999999994 | 297.820557262135 | 1000 | 0.393751815080782 |
| 25.0636799999994 | 298.125366302829 | 1000 | 0.393750816073006 |
| 25.0893599999994 | 298.430174015272 | 1000 | 0.393749817065302 |
| 25.1150399999994 | 298.734980399452 | 1000 | 0.393748818057657 |
| 25.1407199999994 | 299.039785455379 | 1000 | 0.393747819050087 |
| 25.1663999999994 | 299.344589184541 | 1000 | 0.393746820044881 |
| 25.1920799999994 | 299.649391585443 | 1000 | 0.393745821039739 |
| 25.2177599999994 | 299.954192655064 | 1000 | 0.393744822030036 |
| 25.2434399999994 | 300.258992396405 | 1000 | 0.393743823020381 |
| 25.2691199999994 | 300.563790810979 | 1000 | 0.393742824013091 |
| 25.2947999999994 | 300.868587898777 | 1000 | 0.393741825008147 |
| 25.3204799999994 | 301.173383655273 | 1000 | 0.393740825998638 |
| 25.3461599999994 | 301.478178086496 | 1000 | 0.393739826993772 |
| 25.3718399999994 | 301.782971187916 | 1000 | 0.393738827986637 |
| 25.3975199999994 | 302.087762958034 | 1000 | 0.393737828974964 |
| 25.4231999999994 | 302.392553404368 | 1000 | 0.393736829970194 |
| 25.4488799999994 | 302.697342520886 | 1000 | 0.393735830963152 |
| 25.4745599999994 | 303.002130307598 | 1000 | 0.393734831953858 |
| 25.5002399999994 | 303.306916767497 | 1000 | 0.393733832946862 |
| 25.5259199999994 | 303.611701899086 | 1000 | 0.393732833939892 |
| 25.5515999999994 | 303.916485703861 | 1000 | 0.393731834935212 |
| 25.5772799999994 | 304.221268178801 | 1000 | 0.393730835928254 |
| 25.6029599999994 | 304.526049323915 | 1000 | 0.393729836919039 |
| 25.6286399999994 | 304.830829140691 | 1000 | 0.393728837909824 |
| 25.6543199999994 | 305.135607633655 | 1000 | 0.393727838907425 |
| 25.6799999999994 | 305.440384793763 | 1000 | 0.393726839898218 |
| 25.7056799999994 | 305.745160627028 | 1000 | 0.393725840891262 |
| 25.7313599999994 | 306.049935131951 | 1000 | 0.393724841884305 |
| 25.7570399999994 | 306.354708307012 | 1000 | 0.393723842875066 |
| 25.7827199999994 | 306.659480155229 | 1000 | 0.393722843868081 |
| 25.8083999999994 | 306.964250675087 | 1000 | 0.393721844861074 |
| 25.8340799999994 | 307.269019866576 | 1000 | 0.393720845854035 |
| 25.8597599999994 | 307.573787729704 | 1000 | 0.393719846846977 |
| 25.8854399999994 | 307.878554264454 | 1000 | 0.393718847839878 |
| 25.9111199999994 | 308.183319470834 | 1000 | 0.393717848832754 |
| 25.9367999999994 | 308.488083348835 | 1000 | 0.393716849825592 |
| 25.9624799999994 | 308.792845899951 | 1000 | 0.393715850820619 |
| 25.9881599999994 | 309.097607116663 | 1000 | 0.393714851806650 |
| 26.0138399999994 | 309.402367012505 | 1000 | 0.393713852803820 |
| 26.0395199999994 | 309.707125575439 | 1000 | 0.393712853794234 |
| 26.0651999999994 | 310.011882812984 | 1000 | 0.393711854789061 |
| 26.0908799999994 | 310.316638717600 | 1000 | 0.393710855777124 |
| 26.1165599999994 | 310.621393296825 | 1000 | 0.393709856769596 |
| 26.1422399999994 | 310.926146549142 | 1000 | 0.393708857764228 |
| 26.1679199999994 | 311.230898471530 | 1000 | 0.393707858756552 |
| 26.1935999999994 | 311.535649065501 | 1000 | 0.393706859748810 |
| 26.2192799999994 | 311.840398329533 | 1000 | 0.393705860738761 |
| 26.2449599999994 | 312.145146266647 | 1000 | 0.393704861730860 |
| 26.2706399999994 | 312.449892873819 | 1000 | 0.393703862720659 |
| 26.2963199999994 | 312.754638155558 | 1000 | 0.393702863714789 |
| 26.3219999999994 | 313.059382105846 | 1000 | 0.393701864704409 |
| 26.3476799999994 | 313.364124730691 | 1000 | 0.393700865698345 |
| 26.3733599999994 | 313.668866024077 | 1000 | 0.393699866687771 |
| 26.3990399999994 | 313.973605990512 | 1000 | 0.393698867679301 |
| 26.4247199999994 | 314.278344628478 | 1000 | 0.393697868670713 |
| 26.4503999999994 | 314.583081936477 | 1000 | 0.393696869659822 |
| 26.4760799999994 | 314.887817917506 | 1000 | 0.393695870651008 |
| 26.5017599999994 | 315.192552571570 | 1000 | 0.393694871644279 |
| 26.5274399999994 | 315.497285894142 | 1000 | 0.393693872633033 |
| 26.5531199999994 | 315.802017888222 | 1000 | 0.393692873621654 |
| 26.5787999999993 | 316.106748555325 | 1000 | 0.393691874612347 |
| 26.6044799999994 | 316.411477893927 | 1000 | 0.393690875602894 |
| 26.6301599999993 | 316.716205904036 | 1000 | 0.393689876593311 |
| 26.6558399999993 | 317.020932585642 | 1000 | 0.393688877583584 |
| 26.6815199999993 | 317.325657937226 | 1000 | 0.393687878571519 |
| 26.7071999999993 | 317.630381961810 | 1000 | 0.393686879561494 |
| 26.7328799999993 | 317.935104659375 | 1000 | 0.393685880553482 |
| 26.7585599999993 | 318.239826025411 | 1000 | 0.393684881540965 |
| 26.7842399999993 | 318.544546064426 | 1000 | 0.393683882530460 |
| 26.8099199999993 | 318.849264774902 | 1000 | 0.393682883519779 |
| 26.8355999999993 | 319.153982156847 | 1000 | 0.393681884508935 |
| 26.8612799999993 | 319.458698208736 | 1000 | 0.393680885495738 |
| 26.8869599999993 | 319.763412932085 | 1000 | 0.393679886482374 |
| 26.9126399999993 | 320.068126329894 | 1000 | 0.393678887473156 |
| 26.9383199999993 | 320.372838396127 | 1000 | 0.393677888459414 |
| 26.9639999999993 | 320.677549135311 | 1000 | 0.393676889447651 |
| 26.9896799999993 | 320.982258545927 | 1000 | 0.393675890435691 |
| 27.0153599999993 | 321.286966627965 | 1000 | 0.393674891423521 |
| 27.0410399999993 | 321.591673379926 | 1000 | 0.393673892409002 |
| 27.0667199999993 | 321.896378804804 | 1000 | 0.393672893396419 |
| 27.0923999999993 | 322.201082902609 | 1000 | 0.393671894385781 |
| 27.1180799999993 | 322.505785668807 | 1000 | 0.393670895370626 |
| 27.1437599999993 | 322.810487107908 | 1000 | 0.393669896357388 |
| 27.1694399999993 | 323.115187216906 | 1000 | 0.393668897341790 |
| 27.1951199999993 | 323.419885998797 | 1000 | 0.393667898328097 |
| 27.2207999999993 | 323.724583452080 | 1000 | 0.393666899314177 |
| 27.2464799999993 | 324.029279576745 | 1000 | 0.393665900300018 |
| 27.2721599999993 | 324.333974372781 | 1000 | 0.393664901285605 |
| 27.2978399999993 | 324.638667840193 | 1000 | 0.393663902270953 |
| 27.3235199999993 | 324.943359975952 | 1000 | 0.393662903251777 |
| 27.3491999999993 | 325.248050786090 | 1000 | 0.393661904236618 |
| 27.3748799999993 | 325.552740267584 | 1000 | 0.393660905221198 |
| 27.4005599999993 | 325.857428420422 | 1000 | 0.393659906205502 |
| 27.4262399999993 | 326.162115243102 | 1000 | 0.393658907187422 |
| 27.4519199999993 | 326.466800738623 | 1000 | 0.393657908171182 |
| 27.4775999999993 | 326.771484905483 | 1000 | 0.393656909154671 |
| 27.5032799999993 | 327.076167743671 | 1000 | 0.393655910137876 |
| 27.5289599999993 | 327.380849253175 | 1000 | 0.393654911120784 |
| 27.5546399999993 | 327.685529430988 | 1000 | 0.393653912099179 |
| 27.5803199999993 | 327.990208286135 | 1000 | 0.393652913085722 |
| 27.6059999999993 | 328.294885806565 | 1000 | 0.393651914063525 |
| 27.6316799999993 | 328.599562001310 | 1000 | 0.393650915045243 |
| 27.6573599999993 | 328.904236867343 | 1000 | 0.393649916026641 |
| 27.6830399999993 | 329.208910403164 | 1000 | 0.393648917005627 |
| 27.7087199999993 | 329.513582611775 | 1000 | 0.393647917986398 |
| 27.7343999999993 | 329.818253491657 | 1000 | 0.393646918966834 |
| 27.7600799999993 | 330.122923041310 | 1000 | 0.393645919944850 |
| 27.7857599999993 | 330.427591263731 | 1000 | 0.393644920924621 |
| 27.8114399999993 | 330.732258158925 | 1000 | 0.393643921906156 |
| 27.8371199999993 | 331.036923722358 | 1000 | 0.393642922883156 |
| 27.8627999999993 | 331.341587960049 | 1000 | 0.393641923863984 |
| 27.8884799999993 | 331.646250862959 | 1000 | 0.393640924836107 |
| 27.9141599999993 | 331.950912443130 | 1000 | 0.393639925816220 |
| 27.9398399999993 | 332.255572693030 | 1000 | 0.393638926793889 |
| 27.9655199999993 | 332.560231611140 | 1000 | 0.393637927767023 |
| 27.9911999999993 | 332.864889203478 | 1000 | 0.393636928743944 |
| 28.0168799999993 | 333.169545468542 | 1000 | 0.393635929722574 |
| 28.0425599999993 | 333.474200400285 | 1000 | 0.393634930694575 |
| 28.0682399999993 | 333.778854006249 | 1000 | 0.393633931670355 |
| 28.0939199999993 | 334.083506284917 | 1000 | 0.393632932647814 |
| 28.1195999999993 | 334.388157231751 | 1000 | 0.393631933620719 |
| 28.1452799999993 | 334.692806851282 | 1000 | 0.393630934595302 |
| 28.1709599999993 | 334.997455140475 | 1000 | 0.393629935567400 |
| 28.1966399999993 | 335.302102102353 | 1000 | 0.393628936541162 |
| 28.2223199999993 | 335.606747733886 | 1000 | 0.393627937512441 |
| 28.2479999999993 | 335.911392038079 | 1000 | 0.393626938485353 |
| 28.2736799999993 | 336.216035013430 | 1000 | 0.393625939457845 |
| 28.2993599999993 | 336.520676658411 | 1000 | 0.393624940427835 |
| 28.3250399999993 | 336.825316976046 | 1000 | 0.393623941399455 |
| 28.3507199999993 | 337.129955963305 | 1000 | 0.393622942368575 |
| 28.3763999999993 | 337.434593623192 | 1000 | 0.393621943339293 |
| 28.4020799999993 | 337.739229951188 | 1000 | 0.393620944305463 |
| 28.4277599999993 | 338.043864954816 | 1000 | 0.393619945277319 |
| 28.4534399999993 | 338.348498626540 | 1000 | 0.393618946244619 |
| 28.4791199999993 | 338.653130969365 | 1000 | 0.393617947211452 |
| 28.5047999999993 | 338.957761986293 | 1000 | 0.393616948181892 |
| 28.5304799999993 | 339.262391672806 | 1000 | 0.393615949149813 |
| 28.5561599999993 | 339.567020028892 | 1000 | 0.393614950115207 |
| 28.5818399999993 | 339.871647056045 | 1000 | 0.393613951080104 |
| 28.6075199999993 | 340.176272755780 | 1000 | 0.393612952046552 |
| 28.6331999999993 | 340.480897126569 | 1000 | 0.393611953012489 |
| 28.6588799999993 | 340.785520166909 | 1000 | 0.393610953975892 |
| 28.6845599999993 | 341.090141879805 | 1000 | 0.393609954940817 |
| 28.7102399999993 | 341.394762263735 | 1000 | 0.393608955905214 |
| 28.7359199999993 | 341.699381318704 | 1000 | 0.393607956869094 |
| 28.7615999999993 | 342.003999043186 | 1000 | 0.393606957830410 |
| 28.7872799999993 | 342.308615437185 | 1000 | 0.393605958789177 |
| 28.8129599999993 | 342.613230506722 | 1000 | 0.393604959753477 |
| 28.8386399999993 | 342.917844244242 | 1000 | 0.393603960713177 |
| 28.8643199999993 | 343.222456654275 | 1000 | 0.393602961674356 |
| 28.8899999999993 | 343.527067733786 | 1000 | 0.393601962632950 |
| 28.9156799999993 | 343.831677484287 | 1000 | 0.393600963590992 |
| 28.9413599999993 | 344.136285908783 | 1000 | 0.393599964552496 |
| 28.9670399999993 | 344.440892999718 | 1000 | 0.393598965507377 |
| 28.9927199999993 | 344.745498764640 | 1000 | 0.393597966465712 |
| 29.0183999999993 | 345.050103199004 | 1000 | 0.393596967421444 |
| 29.0440799999992 | 345.354706304322 | 1000 | 0.393595968376595 |
| 29.0697599999993 | 345.659308082091 | 1000 | 0.393594969333156 |
| 29.0954399999992 | 345.963908530789 | 1000 | 0.393593970289105 |
| 29.1211199999992 | 346.268507650420 | 1000 | 0.393592971244452 |
| 29.1467999999992 | 346.573105439457 | 1000 | 0.393591972197175 |
| 29.1724799999992 | 346.877701899412 | 1000 | 0.393590973149288 |
| 29.1981599999992 | 347.182297031781 | 1000 | 0.393589974102773 |
| 29.2238399999992 | 347.486890835042 | 1000 | 0.393588975055617 |
| 29.2495199999992 | 347.791483307691 | 1000 | 0.393587976005836 |
| 29.2751999999992 | 348.096074451217 | 1000 | 0.393586976955405 |
| 29.3008799999992 | 348.400664267132 | 1000 | 0.393585977906324 |
| 29.3265599999992 | 348.705252750899 | 1000 | 0.393584978852610 |
| 29.3522399999992 | 349.009839908536 | 1000 | 0.393583979802203 |
| 29.3779199999992 | 349.314425737032 | 1000 | 0.393582980751134 |
| 29.4035999999992 | 349.619010236374 | 1000 | 0.393581981699389 |
| 29.4292799999992 | 349.923593406546 | 1000 | 0.393580982646955 |
| 29.4549599999992 | 350.228175244536 | 1000 | 0.393579983589882 |
| 29.4806399999992 | 350.532755756360 | 1000 | 0.393578984536069 |
| 29.5063199999992 | 350.837334937494 | 1000 | 0.393577985479588 |
| 29.5319999999992 | 351.141912790944 | 1000 | 0.393576986424377 |
| 29.5576799999992 | 351.446489313676 | 1000 | 0.393575987366473 |
| 29.5833599999992 | 351.751064507206 | 1000 | 0.393574988307862 |
| 29.6090399999992 | 352.055638371511 | 1000 | 0.393573989248520 |
| 29.6347199999992 | 352.360210908105 | 1000 | 0.393572990190426 |
| 29.6603999999992 | 352.664782113957 | 1000 | 0.393571991129627 |
| 29.6860799999992 | 352.969351989052 | 1000 | 0.393570992066116 |
| 29.7117599999992 | 353.273920540939 | 1000 | 0.393569993009717 |
| 29.7374399999992 | 353.578487757526 | 1000 | 0.393568993944705 |
| 29.7631199999992 | 353.883053647871 | 1000 | 0.393567994882861 |
| 29.7887999999992 | 354.187618207432 | 1000 | 0.393566995818289 |
| 29.8144799999992 | 354.492181439214 | 1000 | 0.393565996754892 |
| 29.8401599999992 | 354.796743340202 | 1000 | 0.393564997688765 |
| 29.8658399999992 | 355.101303911885 | 1000 | 0.393563998621844 |
| 29.8915199999992 | 355.405863155775 | 1000 | 0.393562999556088 |
| 29.9171999999992 | 355.710421070349 | 1000 | 0.393562000489530 |
| 29.9428799999992 | 356.014977654083 | 1000 | 0.393561001420206 |
| 29.9685599999992 | 356.319532906982 | 1000 | 0.393560002348132 |
| 29.9942399999992 | 356.624086835060 | 1000 | 0.393559003281066 |
| 30.0199199999992 | 356.928639427758 | 1000 | 0.393558004205405 |
| 30.0455999999992 | 357.233190695625 | 1000 | 0.393557005134740 |
| 30.0712799999992 | 357.537740632610 | 1000 | 0.393556006061284 |
| 30.0969599999992 | 357.842289243243 | 1000 | 0.393555006990864 |
| 30.1226399999992 | 358.146836519958 | 1000 | 0.393554007913770 |
| 30.1483199999992 | 358.451382470304 | 1000 | 0.393553008839694 |
| 30.1739999999992 | 358.755927089738 | 1000 | 0.393552009762815 |
| 30.1996799999992 | 359.060470381265 | 1000 | 0.393551010686985 |
| 30.2253599999992 | 359.365012341869 | 1000 | 0.393550011608349 |
| 30.2510399999992 | 359.669552973039 | 1000 | 0.393549012528817 |
| 30.2767199999992 | 359.974092274776 | 1000 | 0.393548013448398 |
| 30.3023999999992 | 360.278630247067 | 1000 | 0.393547014367077 |
| 30.3280799999992 | 360.583166889897 | 1000 | 0.393546015284839 |
| 30.3537599999992 | 360.887702206286 | 1000 | 0.393545016205534 |
| 30.3794399999992 | 361.192236188674 | 1000 | 0.393544017119541 |
| 30.4051199999992 | 361.496768843082 | 1000 | 0.393543018034532 |
| 30.4307999999992 | 361.801300168003 | 1000 | 0.393542018948592 |
| 30.4564799999992 | 362.105830164927 | 1000 | 0.393541019863614 |
| 30.4821599999992 | 362.410358832346 | 1000 | 0.393540020777689 |
| 30.5078399999992 | 362.714886168736 | 1000 | 0.393539021688890 |
| 30.5335199999992 | 363.019412174081 | 1000 | 0.393538022597207 |
| 30.5591999999992 | 363.323936852912 | 1000 | 0.393537023508377 |
| 30.5848799999992 | 363.628460199170 | 1000 | 0.393536024414742 |
| 30.6105599999992 | 363.932982218895 | 1000 | 0.393535025323939 |
| 30.6362399999992 | 364.237502906035 | 1000 | 0.393534026228330 |
| 30.6619199999992 | 364.542022265100 | 1000 | 0.393533027133615 |
| 30.6875999999992 | 364.846540297605 | 1000 | 0.393532028041698 |
| 30.7132799999992 | 365.151056997488 | 1000 | 0.393531028944950 |
| 30.7389599999992 | 365.455572367772 | 1000 | 0.393530029847185 |
| 30.7646399999992 | 365.760086406930 | 1000 | 0.393529030746490 |
| 30.7903199999992 | 366.064599119477 | 1000 | 0.393528031648538 |
| 30.8159999999992 | 366.369110500884 | 1000 | 0.393527032547649 |
| 30.8416799999992 | 366.673620552641 | 1000 | 0.393526033445697 |
| 30.8673599999992 | 366.978129276258 | 1000 | 0.393525034344578 |
| 30.8930399999992 | 367.282636668701 | 1000 | 0.393524035240496 |
| 30.9187199999992 | 367.587142729953 | 1000 | 0.393523036133440 |
| 30.9443999999992 | 367.891647463036 | 1000 | 0.393522037027194 |
| 30.9700799999992 | 368.196150867928 | 1000 | 0.393521037921728 |
| 30.9957599999992 | 368.500652941610 | 1000 | 0.393520038813282 |
| 31.0214399999992 | 368.805153684068 | 1000 | 0.393519039701845 |
| 31.0471199999992 | 369.109653099813 | 1000 | 0.393518040593043 |
| 31.0727999999992 | 369.414151184318 | 1000 | 0.393517041481241 |
| 31.0984799999992 | 369.718647940581 | 1000 | 0.393516042370174 |
| 31.1241599999992 | 370.023143364074 | 1000 | 0.393515043254220 |
| 31.1498399999992 | 370.327637460819 | 1000 | 0.393514044140865 |
| 31.1755199999992 | 370.632130226270 | 1000 | 0.393513045024469 |
| 31.2011999999992 | 370.936621661940 | 1000 | 0.393512045906914 |
| 31.2268799999992 | 371.241111767805 | 1000 | 0.393511046788178 |
| 31.2525599999992 | 371.545600543866 | 1000 | 0.393510047668266 |
| 31.2782399999992 | 371.850087991618 | 1000 | 0.393509048549029 |
| 31.3039199999992 | 372.154574106512 | 1000 | 0.393508049424854 |
| 31.3295999999992 | 372.459058891572 | 1000 | 0.393507050299484 |
| 31.3552799999992 | 372.763542351309 | 1000 | 0.393506051178482 |
| 31.3809599999992 | 373.068024476646 | 1000 | 0.393505052050665 |
| 31.4066399999992 | 373.372505275136 | 1000 | 0.393504052925343 |
| 31.4323199999992 | 373.676984742225 | 1000 | 0.393503053796912 |
| 31.4579999999992 | 373.981462877913 | 1000 | 0.393502054665386 |
| 31.4836799999992 | 374.285939686715 | 1000 | 0.393501055536312 |
| 31.5093599999992 | 374.590415162572 | 1000 | 0.393500056402258 |
| 31.5350399999991 | 374.894889310016 | 1000 | 0.393499057268790 |
| 31.5607199999991 | 375.199362127513 | 1000 | 0.393498058134032 |
| 31.5863999999991 | 375.503833615064 | 1000 | 0.393497058997990 |
| 31.6120799999991 | 375.808303771142 | 1000 | 0.393496059858802 |
| 31.6377599999991 | 376.112772601776 | 1000 | 0.393495060723841 |
| 31.6634399999991 | 376.417240097882 | 1000 | 0.393494061582013 |
| 31.6891199999991 | 376.721706264002 | 1000 | 0.393493062438877 |
| 31.7147999999991 | 377.026171103133 | 1000 | 0.393492063298089 |
| 31.7404799999991 | 377.330634610734 | 1000 | 0.393491064154115 |
| 31.7661599999991 | 377.635096789813 | 1000 | 0.393490065010627 |
| 31.7918399999991 | 377.939557635815 | 1000 | 0.393489065862088 |
| 31.8175199999991 | 378.244017151774 | 1000 | 0.393488066712196 |
| 31.8431999999991 | 378.548475339176 | 1000 | 0.393487067562761 |
| 31.8688799999991 | 378.852932198009 | 1000 | 0.393486068413769 |
| 31.8945599999991 | 379.157387722220 | 1000 | 0.393485069257888 |
| 31.9202399999991 | 379.461841919337 | 1000 | 0.393484070104248 |
| 31.9459199999991 | 379.766294787861 | 1000 | 0.393483070951035 |
| 31.9715999999991 | 380.070746323237 | 1000 | 0.393482071792743 |
| 31.9972799999991 | 380.375196531495 | 1000 | 0.393481072636669 |
| 32.0229599999991 | 380.679645406580 | 1000 | 0.393480073475504 |
| 32.0486399999991 | 380.984092954511 | 1000 | 0.393479074316518 |
| 32.0743199999991 | 381.288539169257 | 1000 | 0.393478075152443 |
| 32.0999999999991 | 381.592984055326 | 1000 | 0.393477075988719 |
| 32.1256799999991 | 381.897427611194 | 1000 | 0.393476076823515 |
| 32.1513599999991 | 382.201869835338 | 1000 | 0.393475077655006 |
| 32.1770399999991 | 382.506310730756 | 1000 | 0.393474078486802 |
| 32.2027199999991 | 382.810750295947 | 1000 | 0.393473079317103 |
| 32.2283999999991 | 383.115188530888 | 1000 | 0.393472080145888 |
| 32.2540799999991 | 383.419625435566 | 1000 | 0.393471080973145 |
| 32.2797599999991 | 383.724061009967 | 1000 | 0.393470081798866 |
| 32.3054399999991 | 384.028495254068 | 1000 | 0.393469082623027 |
| 32.3311199999991 | 384.332928166369 | 1000 | 0.393468083443841 |
| 32.3567999999991 | 384.637359748355 | 1000 | 0.393467084263092 |
| 32.3824799999991 | 384.941790003035 | 1000 | 0.393466085084376 |
| 32.4081599999991 | 385.246218925864 | 1000 | 0.393465085902271 |
| 32.4338399999991 | 385.550646518327 | 1000 | 0.393464086718557 |
| 32.4595199999991 | 385.855072778925 | 1000 | 0.393463087531455 |
| 32.4851999999991 | 386.159497709142 | 1000 | 0.393462088342742 |
| 32.5108799999991 | 386.463921311989 | 1000 | 0.393461089155998 |
| 32.5365599999991 | 386.768343582918 | 1000 | 0.393460089965823 |
| 32.5622399999991 | 387.072764523415 | 1000 | 0.393459090773989 |
| 32.5879199999991 | 387.377184130470 | 1000 | 0.393458091576933 |
| 32.6135999999991 | 387.681602410099 | 1000 | 0.393457092381799 |
| 32.6392799999991 | 387.986019357757 | 1000 | 0.393456093183201 |
| 32.6649599999991 | 388.290434977964 | 1000 | 0.393455093986496 |
| 32.6906399999991 | 388.594849264648 | 1000 | 0.393454094784508 |
| 32.7163199999991 | 388.899262220844 | 1000 | 0.393453095580832 |
| 32.7419999999991 | 389.203673849546 | 1000 | 0.393452096379009 |
| 32.7676799999991 | 389.508084144698 | 1000 | 0.393451097171895 |
| 32.7933599999991 | 389.812493110817 | 1000 | 0.393450097964828 |
| 32.8190399999991 | 390.116900744869 | 1000 | 0.393449098754237 |
| 32.8447199999991 | 390.421307049849 | 1000 | 0.393448099543654 |
| 32.8703999999991 | 390.725712022745 | 1000 | 0.393447100329543 |
| 32.8960799999991 | 391.030115668064 | 1000 | 0.393446101117206 |
| 32.9217599999991 | 391.334517979747 | 1000 | 0.393445101899531 |
| 32.9474399999991 | 391.638918960803 | 1000 | 0.393444102680061 |
| 32.9731199999991 | 391.943318611204 | 1000 | 0.393443103458770 |
| 32.9987999999991 | 392.247716932472 | 1000 | 0.393442104237444 |
| 33.0244799999991 | 392.552113923069 | 1000 | 0.393441105014288 |
| 33.0501599999991 | 392.856509582981 | 1000 | 0.393440105789290 |
| 33.0758399999991 | 393.160903910681 | 1000 | 0.393439106560675 |
| 33.1015199999991 | 393.465296909166 | 1000 | 0.393438107331949 |
| 33.1271999999991 | 393.769688576932 | 1000 | 0.393437108101362 |
| 33.1528799999991 | 394.074078912444 | 1000 | 0.393436108867130 |
| 33.1785599999991 | 394.378467915685 | 1000 | 0.393435109629244 |
| 33.2042399999991 | 394.682855589662 | 1000 | 0.393434110391215 |
| 33.2299199999991 | 394.987241935860 | 1000 | 0.393433111154771 |
| 33.2555999999991 | 395.291626948244 | 1000 | 0.393432111912897 |
| 33.2812799999991 | 395.596010631319 | 1000 | 0.393431112670839 |
| 33.3069599999991 | 395.900392983560 | 1000 | 0.393430113426830 |
| 33.3326399999991 | 396.204774003439 | 1000 | 0.393429114179106 |
| 33.3583199999991 | 396.509153692441 | 1000 | 0.393428114929397 |
| 33.3839999999991 | 396.813532050573 | 1000 | 0.393427115677719 |
| 33.4096799999991 | 397.117909079321 | 1000 | 0.393426116425793 |
| 33.4353599999991 | 397.422284777157 | 1000 | 0.393425117171860 |
| 33.4610399999991 | 397.726659142553 | 1000 | 0.393424117914162 |
| 33.4867199999991 | 398.031032178505 | 1000 | 0.393423118656164 |
| 33.5123999999991 | 398.335403881999 | 1000 | 0.393422119394395 |
| 33.5380799999991 | 398.639774254518 | 1000 | 0.393421120130574 |
| 33.5637599999991 | 398.944143297558 | 1000 | 0.393420120866429 |
| 33.5894399999991 | 399.248511008078 | 1000 | 0.393419121598467 |
| 33.6151199999991 | 399.552877389076 | 1000 | 0.393418122330142 |
| 33.6407999999991 | 399.857242436023 | 1000 | 0.393417123056257 |
| 33.6664799999991 | 400.161606154938 | 1000 | 0.393416123783733 |
| 33.6921599999991 | 400.465968539758 | 1000 | 0.393415124505617 |
| 33.7178399999991 | 400.770329596513 | 1000 | 0.393414125228833 |
| 33.7435199999991 | 401.074689322151 | 1000 | 0.393413125949885 |
| 33.7691999999991 | 401.379047713657 | 1000 | 0.393412126665332 |
| 33.7948799999991 | 401.683404777049 | 1000 | 0.393411127382066 |
| 33.8205599999991 | 401.987760507775 | 1000 | 0.393410128094888 |
| 33.8462399999991 | 402.292114908842 | 1000 | 0.393409128807240 |
| 33.8719199999991 | 402.596467977197 | 1000 | 0.393408129515644 |
| 33.8975999999990 | 402.900819715871 | 1000 | 0.393407130223565 |
| 33.9232799999991 | 403.205170121811 | 1000 | 0.393406130927529 |
| 33.9489599999991 | 403.509519195002 | 1000 | 0.393405131627530 |
| 33.9746399999991 | 403.813866938448 | 1000 | 0.393404132326994 |
| 34.0003199999990 | 404.118213352121 | 1000 | 0.393403133025893 |
| 34.0259999999991 | 404.422558434515 | 1000 | 0.393402133722521 |
| 34.0516799999990 | 404.726902184090 | 1000 | 0.393401134415137 |
| 34.0773599999990 | 405.031244600828 | 1000 | 0.393400135103732 |
| 34.1030399999990 | 405.335585690759 | 1000 | 0.393399135795146 |
| 34.1287199999990 | 405.639925444783 | 1000 | 0.393398136479076 |
| 34.1543999999990 | 405.944263868952 | 1000 | 0.393397137162387 |
| 34.1800799999990 | 406.248600963237 | 1000 | 0.393396137845048 |
| 34.2057599999990 | 406.552936724598 | 1000 | 0.393395138523627 |
| 34.2314399999990 | 406.857271154527 | 1000 | 0.393394139199823 |
| 34.2571199999990 | 407.161604251483 | 1000 | 0.393393139871903 |
| 34.2827999999990 | 407.465936020007 | 1000 | 0.393392140544995 |
| 34.3084799999990 | 407.770266454022 | 1000 | 0.393391141212258 |
| 34.3341599999990 | 408.074595559557 | 1000 | 0.393390141880488 |
| 34.3598399999990 | 408.378923332057 | 1000 | 0.393389142544567 |
| 34.3855199999990 | 408.683249771494 | 1000 | 0.393388143204470 |
| 34.4111999999990 | 408.987574882408 | 1000 | 0.393387143865308 |
| 34.4368799999990 | 409.291898660232 | 1000 | 0.393386144521957 |
| 34.4625599999990 | 409.596221106460 | 1000 | 0.393385145176104 |
| 34.4882399999990 | 409.900542221073 | 1000 | 0.393384145827733 |
| 34.5139199999990 | 410.204862004040 | 1000 | 0.393383146476819 |
| 34.5395999999990 | 410.509180453855 | 1000 | 0.393382147121684 |
| 34.5652799999990 | 410.813497575022 | 1000 | 0.393381147767374 |
| 34.5909599999990 | 411.117813361475 | 1000 | 0.393380148407113 |
| 34.6166399999990 | 411.422127819242 | 1000 | 0.393379149047644 |
| 34.6423199999990 | 411.726440943756 | 1000 | 0.393378149683874 |
| 34.6679999999990 | 412.030752736534 | 1000 | 0.393377150317506 |
| 34.6936799999990 | 412.335063197544 | 1000 | 0.393376150948511 |
| 34.7193599999990 | 412.639372325255 | 1000 | 0.393375151575194 |
| 34.7450399999990 | 412.943680124183 | 1000 | 0.393374152202586 |
| 34.7707199999990 | 413.247986588248 | 1000 | 0.393373152823935 |
| 34.7963999999990 | 413.552291721991 | 1000 | 0.393372153444295 |
| 34.8220799999990 | 413.856595523869 | 1000 | 0.393371154061959 |
| 34.8477599999990 | 414.160897993860 | 1000 | 0.393370154676911 |
| 34.8734399999990 | 414.465199131945 | 1000 | 0.393369155289138 |
| 34.8991199999990 | 414.769498938092 | 1000 | 0.393368155898612 |
| 34.9247999999990 | 415.073797412304 | 1000 | 0.393367156505345 |
| 34.9504799999990 | 415.378094554550 | 1000 | 0.393366157109309 |
| 34.9761599999990 | 415.682390363297 | 1000 | 0.393365157708820 |
| 35.0018399999990 | 415.986684843060 | 1000 | 0.393364158308873 |
| 35.0275199999990 | 416.290977987759 | 1000 | 0.393363158902763 |
| 35.0531999999990 | 416.595269801934 | 1000 | 0.393362159495507 |
| 35.0788799999990 | 416.899560282527 | 1000 | 0.393361160083745 |
| 35.1045599999990 | 417.203849432541 | 1000 | 0.393360160670792 |
| 35.1302399999990 | 417.508137248932 | 1000 | 0.393359161253304 |
| 35.1559199999990 | 417.812423734691 | 1000 | 0.393358161834580 |
| 35.1815999999990 | 418.116708888308 | 1000 | 0.393357162412968 |
| 35.2072799999990 | 418.420992708238 | 1000 | 0.393356162986780 |
| 35.2329599999990 | 418.725275197485 | 1000 | 0.393355163559320 |
| 35.2586399999990 | 419.029556354513 | 1000 | 0.393354164128913 |
| 35.2843199999990 | 419.333836177791 | 1000 | 0.393353164693889 |
| 35.3099999999990 | 419.638114671820 | 1000 | 0.393352165259184 |
| 35.3356799999990 | 419.942391830555 | 1000 | 0.393351165818190 |
| 35.3613599999990 | 420.246667658498 | 1000 | 0.393350166375843 |
| 35.3870399999990 | 420.550942152603 | 1000 | 0.393349166928820 |
| 35.4127199999990 | 420.855215315871 | 1000 | 0.393348167480410 |
| 35.4383999999990 | 421.159487145245 | 1000 | 0.393347168027283 |
| 35.4640799999990 | 421.463757642239 | 1000 | 0.393346168571100 |
| 35.4897599999990 | 421.768026809843 | 1000 | 0.393345169115126 |
| 35.5154399999990 | 422.072294640473 | 1000 | 0.393344169651111 |
| 35.5411199999990 | 422.376561143181 | 1000 | 0.393343170188915 |
| 35.5667999999990 | 422.680826310369 | 1000 | 0.393342170720283 |
| 35.5924799999990 | 422.985090145065 | 1000 | 0.393341171248516 |
| 35.6181599999990 | 423.289352648745 | 1000 | 0.393340171775225 |
| 35.6438399999990 | 423.593613818362 | 1000 | 0.393339172297113 |
| 35.6695199999990 | 423.897873656919 | 1000 | 0.393338172817442 |
| 35.6951999999990 | 424.202132161353 | 1000 | 0.393337173332906 |
| 35.7208799999990 | 424.506389334693 | 1000 | 0.393336173846790 |
| 35.7465599999990 | 424.810645173877 | 1000 | 0.393335174355791 |
| 35.7722399999990 | 425.114899680394 | 1000 | 0.393334174861530 |
| 35.7979199999990 | 425.419152857246 | 1000 | 0.393333175367256 |
| 35.8235999999990 | 425.723404696835 | 1000 | 0.393332175864775 |
| 35.8492799999990 | 426.027655205210 | 1000 | 0.393331176360628 |
| 35.8749599999990 | 426.331904380824 | 1000 | 0.393330176853152 |
| 35.9006399999990 | 426.636152225166 | 1000 | 0.393329177343959 |
| 35.9263199999990 | 426.940398735185 | 1000 | 0.393328177829774 |
| 35.9519999999990 | 427.244643913871 | 1000 | 0.393327178313823 |
| 35.9776799999990 | 427.548887758199 | 1000 | 0.393326178792862 |
| 36.0033599999990 | 427.853130269644 | 1000 | 0.393325179268488 |
| 36.0290399999990 | 428.157371448183 | 1000 | 0.393324179740684 |
| 36.0547199999990 | 428.461611295303 | 1000 | 0.393323180211053 |
| 36.0803999999990 | 428.765849806430 | 1000 | 0.393322180674702 |
| 36.1060799999990 | 429.070086989125 | 1000 | 0.393321181139740 |
| 36.1317599999990 | 429.374322835790 | 1000 | 0.393320181598039 |
| 36.1574399999990 | 429.678557349423 | 1000 | 0.393319182052821 |
| 36.1831199999990 | 429.982790530001 | 1000 | 0.393318182504069 |
| 36.2087999999990 | 430.287022377485 | 1000 | 0.393317182951751 |
| 36.2344799999990 | 430.591252891874 | 1000 | 0.393316183395876 |
| 36.2601599999990 | 430.895482073131 | 1000 | 0.393315183836413 |
| 36.2858399999990 | 431.199709921230 | 1000 | 0.393314184273345 |
| 36.3115199999990 | 431.503936437658 | 1000 | 0.393313184708262 |
| 36.3371999999990 | 431.808161619352 | 1000 | 0.393312185137915 |
| 36.3628799999989 | 432.112385466310 | 1000 | 0.393311185562314 |
| 36.3885599999990 | 432.416607981519 | 1000 | 0.393310185984643 |
| 36.4142399999990 | 432.720829163440 | 1000 | 0.393309186403277 |
| 36.4399199999990 | 433.025049012048 | 1000 | 0.393308186818196 |
| 36.4655999999989 | 433.329267527303 | 1000 | 0.393307187229370 |
| 36.4912799999989 | 433.633484710716 | 1000 | 0.393306187638408 |
| 36.5169599999989 | 433.937700557709 | 1000 | 0.393305188040473 |
| 36.5426399999989 | 434.241915072795 | 1000 | 0.393304188440351 |
| 36.5683199999989 | 434.546128254433 | 1000 | 0.393303188836423 |
| 36.5939999999989 | 434.850340104098 | 1000 | 0.393302189230253 |
| 36.6196799999989 | 435.154550617248 | 1000 | 0.393301189617059 |
| 36.6453599999989 | 435.458759798382 | 1000 | 0.393300190001598 |
| 36.6710399999989 | 435.762967645959 | 1000 | 0.393299190382255 |
| 36.6967199999989 | 436.067174158440 | 1000 | 0.393298190757419 |
| 36.7223999999989 | 436.371379338811 | 1000 | 0.393297191130243 |
| 36.7480799999989 | 436.675583187067 | 1000 | 0.393296191500731 |
| 36.7737599999989 | 436.979785698630 | 1000 | 0.393295191864080 |
| 36.7994399999989 | 437.283986876499 | 1000 | 0.393294192223454 |
| 36.8251199999989 | 437.588186723670 | 1000 | 0.393293192582006 |
| 36.8507999999989 | 437.892385235565 | 1000 | 0.393292192934941 |
| 36.8764799999989 | 438.196582413692 | 1000 | 0.393291193283852 |
| 36.9021599999989 | 438.500778256497 | 1000 | 0.393290193627123 |
| 36.9278399999989 | 438.804972768492 | 1000 | 0.393289193969487 |
| 36.9535199999989 | 439.109165942082 | 1000 | 0.393288194303007 |
| 36.9791999999989 | 439.413357784791 | 1000 | 0.393287194635565 |
| 37.0048799999989 | 439.717548293589 | 1000 | 0.393286194964001 |
| 37.0305599999989 | 440.021737466920 | 1000 | 0.393285195286703 |
| 37.0562399999989 | 440.325925307783 | 1000 | 0.393284195606808 |
| 37.0819199999989 | 440.630111813121 | 1000 | 0.393283195921142 |
| 37.1075999999989 | 440.934296985919 | 1000 | 0.393282196232825 |
| 37.1332799999989 | 441.238480824658 | 1000 | 0.393281196540284 |
| 37.1589599999989 | 441.542663329297 | 1000 | 0.393280196843486 |
| 37.1846399999989 | 441.846844499805 | 1000 | 0.393279197142411 |
| 37.2103199999989 | 442.151024333126 | 1000 | 0.393278197433896 |
| 37.2359999999989 | 442.455202835271 | 1000 | 0.393277197724194 |
| 37.2616799999989 | 442.759380001693 | 1000 | 0.393276198008597 |
| 37.2873599999989 | 443.063555833863 | 1000 | 0.393275198288641 |
| 37.3130399999989 | 443.367730333265 | 1000 | 0.393274198565873 |
| 37.3387199999989 | 443.671903496840 | 1000 | 0.393273198837138 |
| 37.3643999999989 | 443.976075329085 | 1000 | 0.393272199107097 |
| 37.3900799999989 | 444.280245823941 | 1000 | 0.393271199369496 |
| 37.4157599999989 | 444.584414984391 | 1000 | 0.393270199627431 |
| 37.4414399999989 | 444.888582811917 | 1000 | 0.393269199882442 |
| 37.4671199999989 | 445.192749303460 | 1000 | 0.393268200131385 |
| 37.4927999999989 | 445.496914458977 | 1000 | 0.393267200374228 |
| 37.5184799999989 | 445.801078284514 | 1000 | 0.393266200617210 |
| 37.5441599999989 | 446.105240770947 | 1000 | 0.393265200850946 |
| 37.5698399999989 | 446.409401925810 | 1000 | 0.393264201083202 |
| 37.5955199999989 | 446.713561744531 | 1000 | 0.393263201309287 |
| 37.6211999999989 | 447.017720230093 | 1000 | 0.393262201532276 |
| 37.6468799999989 | 447.321877376434 | 1000 | 0.393261201745958 |
| 37.6725599999989 | 447.626033194103 | 1000 | 0.393260201961170 |
| 37.6982399999989 | 447.930187672472 | 1000 | 0.393259202167022 |
| 37.7239199999989 | 448.234340819078 | 1000 | 0.393258202371250 |
| 37.7495999999989 | 448.538492629333 | 1000 | 0.393257202569167 |
| 37.7752799999989 | 448.842643104742 | 1000 | 0.393256202762325 |
| 37.8009599999989 | 449.146792246775 | 1000 | 0.393255202952237 |
| 37.8266399999989 | 449.450940050856 | 1000 | 0.393254203134239 |
| 37.8523199999989 | 449.755086521492 | 1000 | 0.393253203312950 |
| 37.8779999999989 | 450.059231657124 | 1000 | 0.393252203486786 |
| 37.9036799999989 | 450.363375457742 | 1000 | 0.393251203655750 |
| 37.9293599999989 | 450.667517923301 | 1000 | 0.393250203819807 |
| 37.9550399999989 | 450.971659055279 | 1000 | 0.393249203980473 |
| 37.9807199999989 | 451.275798850615 | 1000 | 0.393248204134644 |
| 38.0063999999989 | 451.579937310776 | 1000 | 0.393247204283825 |
| 38.0320799999989 | 451.884074434237 | 1000 | 0.393246204426481 |
| 38.0577599999989 | 452.188210223979 | 1000 | 0.393245204565650 |
| 38.0834399999989 | 452.492344678454 | 1000 | 0.393244204699772 |
| 38.1091199999989 | 452.796477797626 | 1000 | 0.393243204828822 |
| 38.1347999999989 | 453.100609581448 | 1000 | 0.393242204952764 |
| 38.1604799999989 | 453.404740029909 | 1000 | 0.393241205071599 |
| 38.1861599999989 | 453.708869141446 | 1000 | 0.393240205183760 |
| 38.2118399999989 | 454.012996919052 | 1000 | 0.393239205292286 |
| 38.2375199999989 | 454.317123361177 | 1000 | 0.393238205395620 |
| 38.2631999999989 | 454.621248466259 | 1000 | 0.393237205492198 |
| 38.2888799999989 | 454.925372238827 | 1000 | 0.393236205586604 |
| 38.3145599999989 | 455.229494672778 | 1000 | 0.393235205672691 |
| 38.3402399999989 | 455.533615771103 | 1000 | 0.393234205753491 |
| 38.3659199999989 | 455.837735535278 | 1000 | 0.393233205830503 |
| 38.3915999999989 | 456.141853963741 | 1000 | 0.393232205902163 |
| 38.4172799999989 | 456.445971056478 | 1000 | 0.393231205968473 |
| 38.4429599999989 | 456.750086811928 | 1000 | 0.393230206027873 |
| 38.4686399999989 | 457.054201231565 | 1000 | 0.393229206081861 |
| 38.4943199999989 | 457.358314316867 | 1000 | 0.393228206131932 |
| 38.5199999999989 | 457.662426064755 | 1000 | 0.393227206175008 |
| 38.5456799999989 | 457.966536479758 | 1000 | 0.393226206215642 |
| 38.5713599999989 | 458.270645554257 | 1000 | 0.393225206246212 |
| 38.5970399999989 | 458.574753295781 | 1000 | 0.393224206274275 |
| 38.6227199999989 | 458.878859699752 | 1000 | 0.393223206295259 |
| 38.6483999999989 | 459.182964767631 | 1000 | 0.393222206310639 |
| 38.6740799999989 | 459.487068500919 | 1000 | 0.393221206321928 |
| 38.6997599999989 | 459.791170895023 | 1000 | 0.393220206324551 |
| 38.7254399999989 | 460.095271954446 | 1000 | 0.393219206323018 |
| 38.7511199999989 | 460.399371680661 | 1000 | 0.393218206318809 |
| 38.7767999999989 | 460.703470069076 | 1000 | 0.393217206307355 |
| 38.8024799999989 | 461.007567116648 | 1000 | 0.393216206285643 |
| 38.8281599999988 | 461.311662833921 | 1000 | 0.393215206264186 |
| 38.8538399999989 | 461.615757211773 | 1000 | 0.393214206233914 |
| 38.8795199999989 | 461.919850253190 | 1000 | 0.393213206197816 |
| 38.9051999999988 | 462.223941958121 | 1000 | 0.393212206155852 |
| 38.9308799999988 | 462.528032328064 | 1000 | 0.393211206109521 |
| 38.9565599999988 | 462.832121359936 | 1000 | 0.393210206055779 |
| 38.9822399999988 | 463.136209053699 | 1000 | 0.393209205994603 |
| 39.0079199999988 | 463.440295413851 | 1000 | 0.393208205930465 |
| 39.0335999999988 | 463.744380435798 | 1000 | 0.393207205858825 |
| 39.0592799999988 | 464.048464122552 | 1000 | 0.393206205782677 |
| 39.0849599999988 | 464.352546469515 | 1000 | 0.393205205697490 |
| 39.1106399999988 | 464.656627479674 | 1000 | 0.393204205606232 |
| 39.1363199999988 | 464.960707156015 | 1000 | 0.393203205511865 |
| 39.1619999999988 | 465.264785492428 | 1000 | 0.393202205408370 |
| 39.1876799999988 | 465.568862493437 | 1000 | 0.393201205300224 |
| 39.2133599999988 | 465.872938155958 | 1000 | 0.393200205184402 |
| 39.2390399999988 | 466.177012484492 | 1000 | 0.393199205065351 |
| 39.2647199999988 | 466.481085474453 | 1000 | 0.393198204938568 |
| 39.2903999999988 | 466.785157127300 | 1000 | 0.393197204805502 |
| 39.3160799999988 | 467.089227443013 | 1000 | 0.393196204666151 |
| 39.3417599999988 | 467.393296420024 | 1000 | 0.393195204518986 |
| 39.3674399999988 | 467.697364061316 | 1000 | 0.393194204366953 |
| 39.3931199999988 | 468.001430363818 | 1000 | 0.393193204207051 |
| 39.4187999999988 | 468.305495332015 | 1000 | 0.393192204043692 |
| 39.4444799999988 | 468.609558959831 | 1000 | 0.393191203870935 |
| 39.4701599999988 | 468.913621250237 | 1000 | 0.393190203691707 |
| 39.4958399999988 | 469.217682204704 | 1000 | 0.393189203507461 |
| 39.5215199999988 | 469.521741821671 | 1000 | 0.393188203316683 |
| 39.5471999999988 | 469.825800101082 | 1000 | 0.393187203119332 |
| 39.5728799999988 | 470.129857039887 | 1000 | 0.393186202912447 |
| 39.5985599999988 | 470.433912645599 | 1000 | 0.393185202703376 |
| 39.6242399999988 | 470.737966910603 | 1000 | 0.393184202484700 |
| 39.6499199999988 | 471.042019839394 | 1000 | 0.393183202260820 |
| 39.6755999999988 | 471.346071427371 | 1000 | 0.393182202027269 |
| 39.7012799999988 | 471.650121679056 | 1000 | 0.393181201788466 |
| 39.7269599999988 | 471.954170594389 | 1000 | 0.393180201544365 |
| 39.7526399999988 | 472.258218170295 | 1000 | 0.393179201291989 |
| 39.7783199999988 | 472.562264408242 | 1000 | 0.393178201032783 |
| 39.8039999999988 | 472.866309311199 | 1000 | 0.393177200769641 |
| 39.8296799999988 | 473.170352873086 | 1000 | 0.393176200496676 |
| 39.8553599999988 | 473.474395096870 | 1000 | 0.393175200216788 |
| 39.8810399999988 | 473.778435980992 | 1000 | 0.393174199928477 |
| 39.9067199999988 | 474.082475531459 | 1000 | 0.393173199637577 |
| 39.9323999999988 | 474.386513739124 | 1000 | 0.393172199335249 |
| 39.9580799999988 | 474.690550608509 | 1000 | 0.393171199025885 |
| 39.9837599999988 | 474.994586144093 | 1000 | 0.393170198713829 |
| 40.0094399999988 | 475.298620336743 | 1000 | 0.393169198390270 |
| 40.0351199999988 | 475.602653192467 | 1000 | 0.393168198061027 |
| 40.0607999999988 | 475.906684709688 | 1000 | 0.393167197724595 |
| 40.0864799999988 | 476.210714888382 | 1000 | 0.393166197380964 |
| 40.1121599999988 | 476.514743728487 | 1000 | 0.393165197030090 |
| 40.1378399999988 | 476.818771229953 | 1000 | 0.393164196671942 |
| 40.1635199999988 | 477.122797392730 | 1000 | 0.393163196306485 |
| 40.1891999999988 | 477.426822215240 | 1000 | 0.393162195932220 |
| 40.2148799999988 | 477.730845700486 | 1000 | 0.393161195552050 |
| 40.2405599999988 | 478.034867846891 | 1000 | 0.393160195164473 |
| 40.2662399999988 | 478.338888654403 | 1000 | 0.393159194769456 |
| 40.2919199999988 | 478.642908122972 | 1000 | 0.393158194366966 |
| 40.3175999999988 | 478.946926252533 | 1000 | 0.393157193956957 |
| 40.3432799999988 | 479.250943041545 | 1000 | 0.393156193537970 |
| 40.3689599999988 | 479.554958491458 | 1000 | 0.393155193111411 |
| 40.3946399999988 | 479.858972603734 | 1000 | 0.393154192678694 |
| 40.4203199999988 | 480.162985376807 | 1000 | 0.393153192238335 |
| 40.4459999999988 | 480.466996809096 | 1000 | 0.393152191788842 |
| 40.4716799999988 | 480.771006903603 | 1000 | 0.393151191333097 |
| 40.4973599999988 | 481.075015657233 | 1000 | 0.393150190868164 |
| 40.5230399999988 | 481.379023071446 | 1000 | 0.393149190395453 |
| 40.5487199999988 | 481.683029149219 | 1000 | 0.393148189917815 |
| 40.5743999999988 | 481.987033882913 | 1000 | 0.393147189427990 |
| 40.6000799999988 | 482.291037278556 | 1000 | 0.393146188931738 |
| 40.6257599999988 | 482.595039336081 | 1000 | 0.393145188429008 |
| 40.6514399999988 | 482.899040052406 | 1000 | 0.393144187916885 |
| 40.6771199999988 | 483.203039430503 | 1000 | 0.393143187398213 |
| 40.7027999999988 | 483.507037465763 | 1000 | 0.393142186868630 |
| 40.7284799999988 | 483.811034165727 | 1000 | 0.393141186335310 |
| 40.7541599999988 | 484.115029521239 | 1000 | 0.393140185789586 |
| 40.7798399999988 | 484.419023538303 | 1000 | 0.393139185237168 |
| 40.8055199999988 | 484.723016216862 | 1000 | 0.393138184678017 |
| 40.8311999999988 | 485.027007552304 | 1000 | 0.393137184107783 |
| 40.8568799999988 | 485.330997549141 | 1000 | 0.393136183530756 |
| 40.8825599999988 | 485.634986207303 | 1000 | 0.393135182946884 |
| 40.9082399999988 | 485.938973522190 | 1000 | 0.393134182351837 |
| 40.9339199999988 | 486.242959498287 | 1000 | 0.393133181749872 |
| 40.9595999999988 | 486.546944134012 | 1000 | 0.393132181139509 |
| 40.9852799999988 | 486.850927429330 | 1000 | 0.393131180520734 |
| 41.0109599999988 | 487.154909382656 | 1000 | 0.393130179892072 |
| 41.0366399999988 | 487.458889996961 | 1000 | 0.393129179256339 |
| 41.0623199999988 | 487.762869270672 | 1000 | 0.393128178612071 |
| 41.0879999999988 | 488.066847202204 | 1000 | 0.393127177957793 |
| 41.1136799999988 | 488.370823796065 | 1000 | 0.393126177297763 |
| 41.1393599999988 | 488.674799046125 | 1000 | 0.393125176626238 |
| 41.1650399999988 | 488.978772955354 | 1000 | 0.393124175946028 |
| 41.1907199999988 | 489.282745525207 | 1000 | 0.393123175258513 |
| 41.2163999999988 | 489.586716754096 | 1000 | 0.393122174562222 |
| 41.2420799999988 | 489.890686640471 | 1000 | 0.393121173855722 |
| 41.2677599999988 | 490.194655187289 | 1000 | 0.393120173141799 |
| 41.2934399999988 | 490.498622391458 | 1000 | 0.393119172417578 |
| 41.3191199999988 | 490.802588255947 | 1000 | 0.393118171685855 |
| 41.3447999999988 | 491.106552777652 | 1000 | 0.393117170943746 |
| 41.3704799999988 | 491.410515958052 | 1000 | 0.393116170192652 |
| 41.3961599999988 | 491.714477797072 | 1000 | 0.393115169432522 |
| 41.4218399999987 | 492.018438296164 | 1000 | 0.393114168664728 |
| 41.4475199999988 | 492.322397452236 | 1000 | 0.393113167886404 |
| 41.4731999999988 | 492.626355266727 | 1000 | 0.393112167098913 |
| 41.4988799999988 | 492.930311739600 | 1000 | 0.393111166302237 |
| 41.5245599999987 | 493.234266870777 | 1000 | 0.393110165496324 |
| 41.5502399999987 | 493.538220658681 | 1000 | 0.393109164679725 |
| 41.5759199999987 | 493.842173109307 | 1000 | 0.393108163858033 |
| 41.6015999999987 | 494.146124215004 | 1000 | 0.393107163024153 |
| 41.6272799999987 | 494.450073977246 | 1000 | 0.393106162179480 |
| 41.6529599999987 | 494.754022398987 | 1000 | 0.393105161326771 |
| 41.6786399999987 | 495.057969478646 | 1000 | 0.393104160464581 |
| 41.7043199999987 | 495.361915216158 | 1000 | 0.393103159592866 |
| 41.7299999999987 | 495.665859611445 | 1000 | 0.393102158711574 |
| 41.7556799999987 | 495.969802662950 | 1000 | 0.393101157819284 |
| 41.7813599999987 | 496.273744373624 | 1000 | 0.393100156918748 |
| 41.8070399999987 | 496.577684740372 | 1000 | 0.393099156007119 |
| 41.8327199999987 | 496.881623764640 | 1000 | 0.393098155085758 |
| 41.8583999999987 | 497.185561449380 | 1000 | 0.393097154157407 |
| 41.8840799999987 | 497.489497790003 | 1000 | 0.393096153217848 |
| 41.9097599999987 | 497.793432786429 | 1000 | 0.393095152267031 |
| 41.9354399999987 | 498.097366440106 | 1000 | 0.393094151306311 |
| 41.9611199999987 | 498.401298750964 | 1000 | 0.393093150335643 |
| 41.9867999999987 | 498.705229720438 | 1000 | 0.393092149356367 |
| 42.0124799999987 | 499.009159346968 | 1000 | 0.393091148367068 |
| 42.0381599999987 | 499.313087628958 | 1000 | 0.393090147366296 |
| 42.0638399999987 | 499.617014567852 | 1000 | 0.393089146355403 |
| 42.0895199999987 | 499.920940163581 | 1000 | 0.393088145334345 |
| 42.1151999999987 | 500.224864417578 | 1000 | 0.393087144304454 |
| 42.1408799999987 | 500.528787326766 | 1000 | 0.393086143262930 |
| 42.1665599999987 | 500.832708891062 | 1000 | 0.393085142209719 |
| 42.1922399999987 | 501.136629114940 | 1000 | 0.393084141148940 |
| 42.2179199999987 | 501.440547993782 | 1000 | 0.393083140076384 |
| 42.2435999999987 | 501.744465529022 | 1000 | 0.393082138993383 |
| 42.2692799999987 | 502.048381720609 | 1000 | 0.393081137899913 |
| 42.2949599999987 | 502.352296568461 | 1000 | 0.393080136795919 |
| 42.3206399999987 | 502.656210070987 | 1000 | 0.393079135679971 |
| 42.3463199999987 | 502.960122231147 | 1000 | 0.393078134554790 |
| 42.3719999999987 | 503.264033045824 | 1000 | 0.393077133417554 |
| 42.3976799999987 | 503.567942519514 | 1000 | 0.393076132272382 |
| 42.4233599999987 | 503.871850646069 | 1000 | 0.393075131113695 |
| 42.4490399999987 | 504.175757429962 | 1000 | 0.393074129945585 |
| 42.4747199999987 | 504.479662869602 | 1000 | 0.393073128766627 |
| 42.5003999999987 | 504.783566964902 | 1000 | 0.393072127576761 |
| 42.5260799999987 | 505.087469714295 | 1000 | 0.393071126374587 |
| 42.5517599999987 | 505.391371119209 | 1000 | 0.393070125161425 |
| 42.5774399999987 | 505.695271179568 | 1000 | 0.393069123937226 |
| 42.6031199999987 | 505.999169895294 | 1000 | 0.393068122701942 |
| 42.6287999999987 | 506.303067267816 | 1000 | 0.393067121456888 |
| 42.6544799999987 | 506.606963294048 | 1000 | 0.393066120199291 |
| 42.6801599999987 | 506.910857975417 | 1000 | 0.393065118930465 |
| 42.7058399999987 | 507.214751311845 | 1000 | 0.393064117650362 |
| 42.7315199999987 | 507.518643303241 | 1000 | 0.393063116358922 |
| 42.7571999999987 | 507.822533951068 | 1000 | 0.393062115057486 |
| 42.7828799999987 | 508.126423252202 | 1000 | 0.393061113743257 |
| 42.8085599999987 | 508.430311206566 | 1000 | 0.393060112416187 |
| 42.8342399999987 | 508.734197818624 | 1000 | 0.393059111080328 |
| 42.8599199999987 | 509.038083083740 | 1000 | 0.393058109731518 |
| 42.8855999999987 | 509.341967003373 | 1000 | 0.393057108371096 |
| 42.9112799999987 | 509.645849577429 | 1000 | 0.393056106999002 |
| 42.9369599999987 | 509.949730804312 | 1000 | 0.393055105613821 |
| 42.9626399999987 | 510.253610686973 | 1000 | 0.393054104218230 |
| 42.9883199999987 | 510.557489222286 | 1000 | 0.393053102809443 |
| 43.0139999999987 | 510.861366413224 | 1000 | 0.393052101390153 |
| 43.0396799999987 | 511.165242258178 | 1000 | 0.393051099958936 |
| 43.0653599999987 | 511.469116755548 | 1000 | 0.393050098514383 |
| 43.0910399999987 | 511.772989908284 | 1000 | 0.393049097059160 |
| 43.1167199999987 | 512.076861713257 | 1000 | 0.393048095590487 |
| 43.1423999999987 | 512.380732171925 | 1000 | 0.393047094109694 |
| 43.1680799999987 | 512.684601285706 | 1000 | 0.393046092618073 |
| 43.1937599999987 | 512.988469051484 | 1000 | 0.393045091112858 |
| 43.2194399999987 | 513.292335470691 | 1000 | 0.393044089595355 |
| 43.2451199999987 | 513.596200544744 | 1000 | 0.393043088066853 |
| 43.2707999999987 | 513.900064269034 | 1000 | 0.393042086523260 |
| 43.2964799999987 | 514.203926649527 | 1000 | 0.393041084969925 |
| 43.3221599999987 | 514.507787680072 | 1000 | 0.393040083401384 |
| 43.3478399999987 | 514.811647365130 | 1000 | 0.393039081821640 |
| 43.3735199999987 | 515.115505704602 | 1000 | 0.393038080230626 |
| 43.3991999999987 | 515.419362693877 | 1000 | 0.393037078624261 |
| 43.4248799999987 | 515.723218337402 | 1000 | 0.393036077006528 |
| 43.4505599999987 | 516.027072632057 | 1000 | 0.393035075374676 |
| 43.4762399999987 | 516.330925580785 | 1000 | 0.393034073731345 |
| 43.5019199999987 | 516.634777181968 | 1000 | 0.393033072075122 |
| 43.5275999999987 | 516.938627432510 | 1000 | 0.393032070403284 |
| 43.5532799999987 | 517.242476339887 | 1000 | 0.393031068722488 |
| 43.5789599999987 | 517.546323897946 | 1000 | 0.393030067027300 |
| 43.6046399999987 | 517.850170106596 | 1000 | 0.393029065317665 |
| 43.6303199999987 | 518.154014967249 | 1000 | 0.393028063594862 |
| 43.6559999999987 | 518.457858481354 | 1000 | 0.393027061860196 |
| 43.6816799999987 | 518.761700645776 | 1000 | 0.393026060110917 |
| 43.7073599999987 | 519.065541461936 | 1000 | 0.393025058348310 |
| 43.7330399999987 | 519.369380929743 | 1000 | 0.393024056572318 |
| 43.7587199999987 | 519.673219049091 | 1000 | 0.393023054782874 |
| 43.7843999999987 | 519.977055819911 | 1000 | 0.393022052979941 |
| 43.8100799999987 | 520.280891243612 | 1000 | 0.393021051164786 |
| 43.8357599999987 | 520.584725315552 | 1000 | 0.393020049333345 |
| 43.8614399999987 | 520.888558038668 | 1000 | 0.393019047488232 |
| 43.8871199999986 | 521.192389415885 | 1000 | 0.393018045632045 |
| 43.9127999999987 | 521.496219442582 | 1000 | 0.393017043760745 |
| 43.9384799999987 | 521.800048120168 | 1000 | 0.393016041875596 |
| 43.9641599999986 | 522.103875447031 | 1000 | 0.393015039975207 |
| 43.9898399999986 | 522.407701427620 | 1000 | 0.393014038063513 |
| 44.0155199999986 | 522.711526055762 | 1000 | 0.393013036135120 |
| 44.0411999999986 | 523.015349332901 | 1000 | 0.393012034191324 |
| 44.0668799999986 | 523.319171263472 | 1000 | 0.393011032236041 |
| 44.0925599999986 | 523.622991842830 | 1000 | 0.393010030265223 |
| 44.1182399999986 | 523.926811073907 | 1000 | 0.393009028281465 |
| 44.1439199999986 | 524.230628953560 | 1000 | 0.393008026282043 |
| 44.1695999999986 | 524.534445483228 | 1000 | 0.393007024268243 |
| 44.1952799999986 | 524.838260662799 | 1000 | 0.393006022239994 |
| 44.2209599999986 | 525.142074490656 | 1000 | 0.393005020195911 |
| 44.2466399999986 | 525.445886971233 | 1000 | 0.393004018139892 |
| 44.2723199999986 | 525.749698099905 | 1000 | 0.393003016067926 |
| 44.2979999999986 | 526.053507878073 | 1000 | 0.393002013981264 |
| 44.3236799999986 | 526.357316304119 | 1000 | 0.393001011878521 |
| 44.3493599999986 | 526.661123382488 | 1000 | 0.393000009763598 |
| 44.3750399999986 | 526.964929105482 | 1000 | 0.392999007629821 |
| 44.4007199999986 | 527.268733482119 | 1000 | 0.392998005485064 |
| 44.4263999999986 | 527.572536506218 | 1000 | 0.392997003323977 |
| 44.4520799999986 | 527.876338177674 | 1000 | 0.392996001146498 |
| 44.4777599999986 | 528.180138497896 | 1000 | 0.392994998953880 |
| 44.5034399999986 | 528.483937468284 | 1000 | 0.392993996747366 |
| 44.5291199999986 | 528.787735085723 | 1000 | 0.392992994524279 |
| 44.5547999999986 | 529.091531351609 | 1000 | 0.392991992285861 |
| 44.5804799999986 | 529.395326264320 | 1000 | 0.392990990030733 |
| 44.6061599999986 | 529.699119828295 | 1000 | 0.392989987762770 |
| 44.6318399999986 | 530.002912040382 | 1000 | 0.392988985479268 |
| 44.6575199999986 | 530.306702897465 | 1000 | 0.392987983177559 |
| 44.6831999999986 | 530.610492405485 | 1000 | 0.392986980862814 |
| 44.7088799999986 | 530.914280559785 | 1000 | 0.392985978531031 |
| 44.7345599999986 | 531.218067360256 | 1000 | 0.392984976182148 |
| 44.7602399999986 | 531.521852811321 | 1000 | 0.392983973820013 |
| 44.7859199999986 | 531.825636906830 | 1000 | 0.392982971439346 |
| 44.8115999999986 | 532.129419649690 | 1000 | 0.392981969042686 |
| 44.8372799999986 | 532.433201039791 | 1000 | 0.392980966629967 |
| 44.8629599999986 | 532.736981080051 | 1000 | 0.392979964203730 |
| 44.8886399999986 | 533.040759762764 | 1000 | 0.392978961757371 |
| 44.9143199999986 | 533.344537095421 | 1000 | 0.392977959297366 |
| 44.9399999999986 | 533.648313074864 | 1000 | 0.392976956821026 |
| 44.9656799999986 | 533.952087697945 | 1000 | 0.392975954325678 |
| 44.9913599999986 | 534.255860970615 | 1000 | 0.392974951816463 |
| 45.0170399999986 | 534.559632888196 | 1000 | 0.392973949289393 |
| 45.0427199999986 | 534.863403453629 | 1000 | 0.392972946747022 |
| 45.0683999999986 | 535.167172662237 | 1000 | 0.392971944185370 |
| 45.0940799999986 | 535.470940516933 | 1000 | 0.392970941606968 |
| 45.1197599999986 | 535.774707019118 | 1000 | 0.392969939013041 |
| 45.1454399999986 | 536.078472165628 | 1000 | 0.392968936400914 |
| 45.1711199999986 | 536.382235959400 | 1000 | 0.392967933773129 |
| 45.1967999999986 | 536.685998398788 | 1000 | 0.392966931128308 |
| 45.2224799999986 | 536.989759483670 | 1000 | 0.392965928466378 |
| 45.2481599999986 | 537.293519212399 | 1000 | 0.392964925785963 |
| 45.2738399999986 | 537.597277586393 | 1000 | 0.392963923088307 |
| 45.2995199999986 | 537.901034607036 | 1000 | 0.392962920374618 |
| 45.3251999999986 | 538.204790269657 | 1000 | 0.392961917640947 |
| 45.3508799999986 | 538.508544580197 | 1000 | 0.392960914892388 |
| 45.3765599999986 | 538.812297533973 | 1000 | 0.392959912124983 |
| 45.4022399999986 | 539.116049132402 | 1000 | 0.392958909339970 |
| 45.4279199999986 | 539.419799375348 | 1000 | 0.392957906537263 |
| 45.4535999999986 | 539.723548262685 | 1000 | 0.392956903716789 |
| 45.4792799999986 | 540.027295794288 | 1000 | 0.392955900878474 |
| 45.5049599999986 | 540.331041970019 | 1000 | 0.392954898022230 |
| 45.5306399999986 | 540.634786788261 | 1000 | 0.392953895146718 |
| 45.5563199999986 | 540.938530250389 | 1000 | 0.392952892253139 |
| 45.5819999999986 | 541.242272357793 | 1000 | 0.392951889342702 |
| 45.6076799999986 | 541.546013107312 | 1000 | 0.392950886412759 |
| 45.6333599999986 | 541.849752501837 | 1000 | 0.392949883465794 |
| 45.6590399999986 | 542.153490538232 | 1000 | 0.392948880499182 |
| 45.6847199999986 | 542.457227219387 | 1000 | 0.392947877515402 |
| 45.7103999999986 | 542.760962540622 | 1000 | 0.392946874510530 |
| 45.7360799999986 | 543.064696506356 | 1000 | 0.392945871488334 |
| 45.7617599999986 | 543.368429114928 | 1000 | 0.392944868447444 |
| 45.7874399999986 | 543.672160366230 | 1000 | 0.392943865387802 |
| 45.8131199999986 | 543.975890258602 | 1000 | 0.392942862308040 |
| 45.8387999999986 | 544.279618794943 | 1000 | 0.392941859210637 |
| 45.8644799999986 | 544.583345973603 | 1000 | 0.392940856094234 |
| 45.8901599999986 | 544.887071794434 | 1000 | 0.392939852958741 |
| 45.9158399999986 | 545.190796254294 | 1000 | 0.392938849801548 |
| 45.9415199999986 | 545.494519357583 | 1000 | 0.392937846626392 |
| 45.9671999999986 | 545.798241105683 | 1000 | 0.392936843434464 |
| 45.9928799999986 | 546.101961492390 | 1000 | 0.392935840220587 |
| 46.0185599999986 | 546.405680520587 | 1000 | 0.392934836987217 |
| 46.0442399999986 | 546.709398190161 | 1000 | 0.392933833734294 |
| 46.0699199999986 | 547.013114500961 | 1000 | 0.392932830461724 |
| 46.0955999999986 | 547.316829451332 | 1000 | 0.392931827168157 |
| 46.1212799999986 | 547.620543044153 | 1000 | 0.392930823856039 |
| 46.1469599999986 | 547.924255279310 | 1000 | 0.392929820525307 |
| 46.1726399999986 | 548.227966153615 | 1000 | 0.392928817173328 |
| 46.1983199999986 | 548.531675666927 | 1000 | 0.392927813800020 |
| 46.2239999999986 | 548.835383820620 | 1000 | 0.392926810406568 |
| 46.2496799999986 | 549.139090614539 | 1000 | 0.392925806992879 |
| 46.2753599999986 | 549.442796050081 | 1000 | 0.392924803560152 |
| 46.3010399999986 | 549.746500124056 | 1000 | 0.392923800105761 |
| 46.3267199999986 | 550.050202836320 | 1000 | 0.392922796629623 |
| 46.3523999999985 | 550.353904191276 | 1000 | 0.392921793135444 |
| 46.3780799999985 | 550.657604184217 | 1000 | 0.392920789619336 |
| 46.4037599999986 | 550.961302816537 | 1000 | 0.392919786082497 |
| 46.4294399999985 | 551.265000088076 | 1000 | 0.392918782524829 |
| 46.4551199999985 | 551.568695997169 | 1000 | 0.392917778944985 |
| 46.4807999999985 | 551.872390546700 | 1000 | 0.392916775345400 |
| 46.5064799999986 | 552.176083733476 | 1000 | 0.392915771723454 |
| 46.5321599999985 | 552.479775560403 | 1000 | 0.392914768081600 |
| 46.5578399999985 | 552.783466024284 | 1000 | 0.392913764417220 |
| 46.5835199999985 | 553.087155126487 | 1000 | 0.392912760731486 |
| 46.6091999999985 | 553.390842865340 | 1000 | 0.392911757023050 |
| 46.6348799999985 | 553.694529245231 | 1000 | 0.392910753295584 |
| 46.6605599999985 | 553.998214261478 | 1000 | 0.392909749545248 |
| 46.6862399999985 | 554.301897915434 | 1000 | 0.392908745773197 |
| 46.7119199999985 | 554.605580206942 | 1000 | 0.392907741979342 |
| 46.7375999999985 | 554.909261135849 | 1000 | 0.392906738163591 |
| 46.7632799999985 | 555.212940700467 | 1000 | 0.392905734324590 |
| 46.7889599999985 | 555.516618903699 | 1000 | 0.392904730464773 |
| 46.8146399999985 | 555.820295742342 | 1000 | 0.392903726581534 |
| 46.8403199999985 | 556.123971217753 | 1000 | 0.392902722676033 |
| 46.8659999999985 | 556.427645331278 | 1000 | 0.392901718749416 |
| 46.8916799999985 | 556.731318078233 | 1000 | 0.392900714797861 |
| 46.9173599999985 | 557.034989462994 | 1000 | 0.392899710825013 |
| 46.9430399999985 | 557.338659483883 | 1000 | 0.392898706829530 |
| 46.9687199999985 | 557.642328140740 | 1000 | 0.392897702811316 |
| 46.9943999999985 | 557.945995433389 | 1000 | 0.392896698770266 |
| 47.0200799999985 | 558.249661360175 | 1000 | 0.392895694705060 |
| 47.0457599999985 | 558.553325923955 | 1000 | 0.392894690618084 |
| 47.0714399999985 | 558.856989123048 | 1000 | 0.392893686507997 |
| 47.0971199999985 | 559.160650955772 | 1000 | 0.392892682373457 |
| 47.1227999999985 | 559.464311423465 | 1000 | 0.392891678215604 |
| 47.1484799999985 | 559.767970527501 | 1000 | 0.392890674035603 |
| 47.1741599999985 | 560.071628263151 | 1000 | 0.392889669829618 |
| 47.1998399999985 | 560.375284636313 | 1000 | 0.392888665602521 |
| 47.2255199999985 | 560.678939640752 | 1000 | 0.392887661349247 |
| 47.2511999999985 | 560.982593279318 | 1000 | 0.392886657072172 |
| 47.2768799999985 | 561.286245553383 | 1000 | 0.392885652772456 |
| 47.3025599999985 | 561.589896461246 | 1000 | 0.392884648448747 |
| 47.3282399999985 | 561.893546001218 | 1000 | 0.392883644099710 |
| 47.3539199999985 | 562.197194174631 | 1000 | 0.392882639726473 |
| 47.3795999999985 | 562.500840982853 | 1000 | 0.392881635330191 |
| 47.4052799999985 | 562.804486424181 | 1000 | 0.392880630909515 |
| 47.4309599999985 | 563.108130496923 | 1000 | 0.392879626463111 |
| 47.4566399999985 | 563.411773200902 | 1000 | 0.392878621990876 |
| 47.4823199999985 | 563.715414540481 | 1000 | 0.392877617496403 |
| 47.5079999999985 | 564.019054509439 | 1000 | 0.392876612974672 |
| 47.5336799999985 | 564.322693110620 | 1000 | 0.392875608428040 |
| 47.5593599999985 | 564.626330345361 | 1000 | 0.392874603857633 |
| 47.5850399999985 | 564.929966213482 | 1000 | 0.392873599263347 |
| 47.6107199999985 | 565.233600710241 | 1000 | 0.392872594641374 |
| 47.6363999999985 | 565.537233836997 | 1000 | 0.392871589992863 |
| 47.6620799999985 | 565.840865596590 | 1000 | 0.392870585320158 |
| 47.6877599999985 | 566.144495988836 | 1000 | 0.392869580623153 |
| 47.7134399999985 | 566.448125009002 | 1000 | 0.392868575898056 |
| 47.7391199999985 | 566.751752661441 | 1000 | 0.392867571148435 |
| 47.7647999999985 | 567.055378942959 | 1000 | 0.392866566371749 |
| 47.7904799999985 | 567.359003856390 | 1000 | 0.392865561570334 |
| 47.8161599999985 | 567.662627396998 | 1000 | 0.392864556740406 |
| 47.8418399999985 | 567.966249570662 | 1000 | 0.392863551886757 |
| 47.8675199999985 | 568.269870372631 | 1000 | 0.392862547005595 |
| 47.8931999999985 | 568.573489804256 | 1000 | 0.392861542098053 |
| 47.9188799999985 | 568.877107865334 | 1000 | 0.392860537164015 |
| 47.9445599999985 | 569.180724555676 | 1000 | 0.392859532203369 |
| 47.9702399999985 | 569.484339875076 | 1000 | 0.392858527215995 |
| 47.9959199999985 | 569.787953823366 | 1000 | 0.392857522201802 |
| 48.0215999999985 | 570.091566400340 | 1000 | 0.392856517160669 |
| 48.0472799999985 | 570.395177602770 | 1000 | 0.392855512090046 |
| 48.0729599999985 | 570.698787436529 | 1000 | 0.392854506994700 |
| 48.0986399999985 | 571.002395896856 | 1000 | 0.392853501870849 |
| 48.1243199999985 | 571.306002985097 | 1000 | 0.392852496719620 |
| 48.1499999999985 | 571.609608701041 | 1000 | 0.392851491540889 |
| 48.1756799999985 | 571.913213044489 | 1000 | 0.392850486334543 |
| 48.2013599999985 | 572.216816013724 | 1000 | 0.392849481099252 |
| 48.2270399999985 | 572.520417611568 | 1000 | 0.392848475837321 |
| 48.2527199999985 | 572.824017834810 | 1000 | 0.392847470546227 |
| 48.2783999999985 | 573.127616686269 | 1000 | 0.392846465228271 |
| 48.3040799999985 | 573.431214161191 | 1000 | 0.392845459879698 |
| 48.3297599999985 | 573.734810265439 | 1000 | 0.392844454505242 |
| 48.3554399999985 | 574.038404995763 | 1000 | 0.392843449102351 |
| 48.3811199999985 | 574.341998348947 | 1000 | 0.392842443668504 |
| 48.4067999999985 | 574.645590329323 | 1000 | 0.392841438207208 |
| 48.4324799999985 | 574.949180932131 | 1000 | 0.392840432714713 |
| 48.4581599999985 | 575.252770163221 | 1000 | 0.392839427195731 |
| 48.4838399999985 | 575.556358019371 | 1000 | 0.392838421647741 |
| 48.5095199999985 | 575.859944498843 | 1000 | 0.392837416069405 |
| 48.5351999999985 | 576.163529602941 | 1000 | 0.392836410461813 |
| 48.5608799999985 | 576.467113331454 | 1000 | 0.392835404824842 |
| 48.5865599999985 | 576.770695684156 | 1000 | 0.392834399158361 |
| 48.6122399999985 | 577.074276660858 | 1000 | 0.392833393462269 |
| 48.6379199999985 | 577.377856258297 | 1000 | 0.392832387734024 |
| 48.6635999999985 | 577.681434482326 | 1000 | 0.392831381978322 |
| 48.6892799999985 | 577.985011328177 | 1000 | 0.392830376191426 |
| 48.7149599999985 | 578.288586798653 | 1000 | 0.392829370375610 |
| 48.7406399999985 | 578.592160889009 | 1000 | 0.392828364527161 |
| 48.7663199999985 | 578.895733603564 | 1000 | 0.392827358649552 |
| 48.7919999999984 | 579.199304940580 | 1000 | 0.392826352741455 |
| 48.8176799999985 | 579.502874898305 | 1000 | 0.392825346801534 |
| 48.8433599999984 | 579.806443479576 | 1000 | 0.392824340832083 |
| 48.8690399999985 | 580.110010682639 | 1000 | 0.392823334831765 |
| 48.8947199999984 | 580.413576505752 | 1000 | 0.392822328799254 |
| 48.9203999999984 | 580.717140950207 | 1000 | 0.392821322735621 |
| 48.9460799999984 | 581.020704015765 | 1000 | 0.392820316640728 |
| 48.9717599999984 | 581.324265702222 | 1000 | 0.392819310514466 |
| 48.9974399999984 | 581.627826007820 | 1000 | 0.392818304355500 |
| 49.0231199999984 | 581.931384933847 | 1000 | 0.392817298164896 |
| 49.0487999999984 | 582.234942483106 | 1000 | 0.392816291944914 |
| 49.0744799999984 | 582.538498649284 | 1000 | 0.392815285690632 |
| 49.1001599999984 | 582.842053435207 | 1000 | 0.392814279404329 |
| 49.1258399999984 | 583.145606840629 | 1000 | 0.392813273085864 |
| 49.1515199999984 | 583.449158865316 | 1000 | 0.392812266735102 |
| 49.1771999999984 | 583.752709509019 | 1000 | 0.392811260351903 |
| 49.2028799999984 | 584.056258770008 | 1000 | 0.392810253934959 |
| 49.2285599999984 | 584.359806649551 | 1000 | 0.392809247485321 |
| 49.2542399999984 | 584.663353148925 | 1000 | 0.392808241004041 |
| 49.2799199999984 | 584.966898264856 | 1000 | 0.392807234488606 |
| 49.3055999999984 | 585.270441998608 | 1000 | 0.392806227940059 |
| 49.3312799999984 | 585.573984351481 | 1000 | 0.392805221359471 |
| 49.3569599999984 | 585.877525317149 | 1000 | 0.392804214741946 |
| 49.3826399999984 | 586.181064902955 | 1000 | 0.392803208093284 |
| 49.4083199999984 | 586.484603105619 | 1000 | 0.392802201410970 |
| 49.4339999999984 | 586.788139924883 | 1000 | 0.392801194694858 |
| 49.4596799999984 | 587.091675357488 | 1000 | 0.392800187942457 |
| 49.4853599999984 | 587.395209409242 | 1000 | 0.392799181158359 |
| 49.5110399999984 | 587.698742073828 | 1000 | 0.392798174337685 |
| 49.5367199999984 | 588.002273355533 | 1000 | 0.392797167483837 |
| 49.5623999999984 | 588.305803251095 | 1000 | 0.392796160594326 |
| 49.5880799999984 | 588.609331761768 | 1000 | 0.392795153670185 |
| 49.6137599999984 | 588.912858888813 | 1000 | 0.392794146712452 |
| 49.6394399999984 | 589.216384628940 | 1000 | 0.392793139718620 |
| 49.6651199999984 | 589.519908983400 | 1000 | 0.392792132689719 |
| 49.6907999999984 | 589.823431951958 | 1000 | 0.392791125625623 |
| 49.7164799999984 | 590.126953534343 | 1000 | 0.392790118526177 |
| 49.7421599999984 | 590.430473727262 | 1000 | 0.392789111388880 |
| 49.7678399999984 | 590.733992536522 | 1000 | 0.392788104218300 |
| 49.7935199999984 | 591.037509957297 | 1000 | 0.392787097010746 |
| 49.8191999999984 | 591.341025992384 | 1000 | 0.392786089768443 |
| 49.8448799999984 | 591.644540636953 | 1000 | 0.392785082487707 |
| 49.8705599999984 | 591.948053895291 | 1000 | 0.392784075171917 |
| 49.8962399999984 | 592.251565764084 | 1000 | 0.392783067818565 |
| 49.9219199999984 | 592.555076244607 | 1000 | 0.392782060428694 |
| 49.9475999999984 | 592.858585338095 | 1000 | 0.392781053003319 |
| 49.9732799999984 | 593.162093041244 | 1000 | 0.392780045539942 |
| 49.9989599999984 | 593.465599353781 | 1000 | 0.392779038038412 |
| 50.0246399999984 | 593.769104276940 | 1000 | 0.392778030499743 |
| 50.0503199999984 | 594.072607813506 | 1000 | 0.392777022926142 |
| 50.0759999999984 | 594.376109958640 | 1000 | 0.392776015313931 |
| 50.1016799999984 | 594.679610712065 | 1000 | 0.392775007662959 |
| 50.1273599999984 | 594.983110073504 | 1000 | 0.392773999973073 |
| 50.1530399999984 | 595.286608047218 | 1000 | 0.392772992247616 |
| 50.1787199999984 | 595.590104626883 | 1000 | 0.392771984481775 |
| 50.2043999999984 | 595.893599816757 | 1000 | 0.392770976678891 |
| 50.2300799999984 | 596.197093613522 | 1000 | 0.392769968836470 |
| 50.2557599999984 | 596.500586018401 | 1000 | 0.392768960955515 |
| 50.2814399999984 | 596.804077032651 | 1000 | 0.392767953037051 |
| 50.3071199999984 | 597.107566651421 | 1000 | 0.392766945077413 |
| 50.3327999999984 | 597.411054878977 | 1000 | 0.392765937079938 |
| 50.3584799999984 | 597.714541713512 | 1000 | 0.392764929043301 |
| 50.3841599999984 | 598.018027153206 | 1000 | 0.392763920966169 |
| 50.4098399999984 | 598.321511197792 | 1000 | 0.392762912848399 |
| 50.4355199999984 | 598.624993851521 | 1000 | 0.392761904693309 |
| 50.4611999999984 | 598.928475109543 | 1000 | 0.392760896497249 |
| 50.4868799999984 | 599.231954973083 | 1000 | 0.392759888261216 |
| 50.5125599999984 | 599.535433440314 | 1000 | 0.392758879983878 |
| 50.5382399999984 | 599.838910512481 | 1000 | 0.392757871666251 |
| 50.5639199999984 | 600.142386189272 | 1000 | 0.392756863308158 |
| 50.5895999999984 | 600.445860471906 | 1000 | 0.392755854910593 |
| 50.6152799999984 | 600.749333353997 | 1000 | 0.392754846468747 |
| 50.6409599999984 | 601.052804844373 | 1000 | 0.392753837989419 |
| 50.6666399999984 | 601.356274933613 | 1000 | 0.392752829465487 |
| 50.6923199999984 | 601.659743627481 | 1000 | 0.392751820901412 |
| 50.7179999999984 | 601.963210924153 | 1000 | 0.392750812295867 |
| 50.7436799999984 | 602.266676824826 | 1000 | 0.392749803649828 |
| 50.7693599999984 | 602.570141326180 | 1000 | 0.392748794960832 |
| 50.7950399999984 | 602.873604427891 | 1000 | 0.392747786228700 |
| 50.8207199999984 | 603.177066132684 | 1000 | 0.392746777455568 |
| 50.8463999999984 | 603.480526435678 | 1000 | 0.392745768637794 |
| 50.8720799999984 | 603.783985341137 | 1000 | 0.392744759778684 |
| 50.8977599999984 | 604.087442847214 | 1000 | 0.392743750876899 |
| 50.9234399999984 | 604.390898952074 | 1000 | 0.392742741931116 |
| 50.9491199999984 | 604.694353658433 | 1000 | 0.392741732943459 |
| 50.9747999999984 | 604.997806964441 | 1000 | 0.392740723912593 |
| 51.0004799999984 | 605.301258865247 | 1000 | 0.392739714834911 |
| 51.0261599999984 | 605.604709369622 | 1000 | 0.392738705717122 |
| 51.0518399999984 | 605.908158469653 | 1000 | 0.392737696553305 |
| 51.0775199999984 | 606.211606168036 | 1000 | 0.392736687345567 |
| 51.1031999999984 | 606.515052464469 | 1000 | 0.392735678093748 |
| 51.1288799999984 | 606.818497361646 | 1000 | 0.392734668799950 |
| 51.1545599999984 | 607.121940848611 | 1000 | 0.392733659455969 |
| 51.1802399999984 | 607.425382940211 | 1000 | 0.392732650073082 |
| 51.2059199999984 | 607.728823623958 | 1000 | 0.392731640641933 |
| 51.2315999999984 | 608.032262902577 | 1000 | 0.392730631164650 |
| 51.2572799999983 | 608.335700778755 | 1000 | 0.392729621643327 |
| 51.2829599999984 | 608.639137249119 | 1000 | 0.392728612075492 |
| 51.3086399999983 | 608.942572317883 | 1000 | 0.392727602464388 |
| 51.3343199999984 | 609.246005980141 | 1000 | 0.392726592806391 |
| 51.3599999999983 | 609.549438235574 | 1000 | 0.392725583101334 |
| 51.3856799999983 | 609.852869083827 | 1000 | 0.392724573349020 |
| 51.4113599999983 | 610.156298527591 | 1000 | 0.392723563551544 |
| 51.4370399999983 | 610.459726563470 | 1000 | 0.392722553706426 |
| 51.4627199999983 | 610.763153194176 | 1000 | 0.392721543815772 |
| 51.4883999999983 | 611.066578414795 | 1000 | 0.392720533875968 |
| 51.5140799999983 | 611.370002229527 | 1000 | 0.392719523890236 |
| 51.5397599999983 | 611.673424636503 | 1000 | 0.392718513857247 |
| 51.5654399999983 | 611.976845635356 | 1000 | 0.392717503776797 |
| 51.5911199999983 | 612.280265225755 | 1000 | 0.392716493648710 |
| 51.6167999999983 | 612.583683404292 | 1000 | 0.392715483470513 |
| 51.6424799999983 | 612.887100176683 | 1000 | 0.392714473246551 |
| 51.6681599999983 | 613.190515534963 | 1000 | 0.392713462970944 |
| 51.6938399999983 | 613.493929486385 | 1000 | 0.392712452649188 |
| 51.7195199999983 | 613.797342027537 | 1000 | 0.392711442278804 |
| 51.7451999999983 | 614.100753155017 | 1000 | 0.392710431857330 |
| 51.7708799999983 | 614.404162874531 | 1000 | 0.392709421389098 |
| 51.7965599999983 | 614.707571179625 | 1000 | 0.392708410869367 |
| 51.8222399999983 | 615.010978071472 | 1000 | 0.392707400299090 |
| 51.8479199999983 | 615.314383555759 | 1000 | 0.392706389682578 |
| 51.8735999999983 | 615.617787621488 | 1000 | 0.392705379011715 |
| 51.8992799999983 | 615.921190278897 | 1000 | 0.392704368294200 |
| 51.9249599999983 | 616.224591520043 | 1000 | 0.392703357524198 |
| 51.9506399999983 | 616.527991347573 | 1000 | 0.392702346703754 |
| 51.9763199999983 | 616.831389762623 | 1000 | 0.392701335833790 |
| 52.0019999999983 | 617.134786761777 | 1000 | 0.392700324911842 |
| 52.0276799999983 | 617.438182344637 | 1000 | 0.392699313937696 |
| 52.0533599999983 | 617.741576512363 | 1000 | 0.392698302912289 |
| 52.0790399999983 | 618.044969264555 | 1000 | 0.392697291835401 |
| 52.1047199999983 | 618.348360599306 | 1000 | 0.392696280705699 |
| 52.1303999999983 | 618.651750520769 | 1000 | 0.392695269526337 |
| 52.1560799999983 | 618.955139022503 | 1000 | 0.392694258292621 |
| 52.1817599999983 | 619.258526107139 | 1000 | 0.392693247006581 |
| 52.2074399999983 | 619.561911774282 | 1000 | 0.392692235668002 |
| 52.2331199999983 | 619.865296022016 | 1000 | 0.392691224275548 |
| 52.2587999999983 | 620.168678852967 | 1000 | 0.392690212831238 |
| 52.2844799999983 | 620.472060262204 | 1000 | 0.392689201331511 |
| 52.3101599999983 | 620.775440255386 | 1000 | 0.392688189780625 |
| 52.3358399999983 | 621.078818824517 | 1000 | 0.392687178172758 |
| 52.3615199999983 | 621.382195978289 | 1000 | 0.392686166514406 |
| 52.3871999999983 | 621.685571707209 | 1000 | 0.392685154798647 |
| 52.4128799999983 | 621.988946016929 | 1000 | 0.392684143029730 |
| 52.4385599999983 | 622.292318905519 | 1000 | 0.392683131206314 |
| 52.4642399999983 | 622.595690372554 | 1000 | 0.392682119328168 |
| 52.4899199999983 | 622.899060414608 | 1000 | 0.392681107392854 |
| 52.5155999999983 | 623.202429037324 | 1000 | 0.392680095404605 |
| 52.5412799999983 | 623.505796234211 | 1000 | 0.392679083358731 |
| 52.5669599999983 | 623.809162009404 | 1000 | 0.392678071258354 |
| 52.5926399999983 | 624.112526359432 | 1000 | 0.392677059101008 |
| 52.6183199999983 | 624.415889285414 | 1000 | 0.392676046887599 |
| 52.6439999999983 | 624.719250786911 | 1000 | 0.392675034617888 |
| 52.6696799999983 | 625.022610860461 | 1000 | 0.392674022289421 |
| 52.6953599999983 | 625.325969510174 | 1000 | 0.392673009905297 |
| 52.7210399999983 | 625.629326735646 | 1000 | 0.392671997465303 |
| 52.7467199999983 | 625.932682531876 | 1000 | 0.392670984965860 |
| 52.7723999999983 | 626.236036902982 | 1000 | 0.392669972410072 |
| 52.7980799999983 | 626.539389845493 | 1000 | 0.392668959795481 |
| 52.8237599999983 | 626.842741360473 | 1000 | 0.392667947122955 |
| 52.8494399999983 | 627.146091445991 | 1000 | 0.392666934391166 |
| 52.8751199999983 | 627.449440103107 | 1000 | 0.392665921600978 |
| 52.9007999999983 | 627.752787334415 | 1000 | 0.392664908754371 |
| 52.9264799999983 | 628.056133131863 | 1000 | 0.392663895845553 |
| 52.9521599999983 | 628.359477498065 | 1000 | 0.392662882876523 |
| 52.9778399999983 | 628.662820437112 | 1000 | 0.392661869850351 |
| 53.0035199999983 | 628.966161945515 | 1000 | 0.392660856764581 |
| 53.0291999999983 | 629.269502019783 | 1000 | 0.392659843616758 |
| 53.0548799999983 | 629.572840662484 | 1000 | 0.392658830408843 |
| 53.0805599999983 | 629.876177873184 | 1000 | 0.392657817140608 |
| 53.1062399999983 | 630.179513652928 | 1000 | 0.392656803812903 |
| 53.1319199999983 | 630.482847995181 | 1000 | 0.392655790421067 |
| 53.1575999999983 | 630.786180907057 | 1000 | 0.392654776970360 |
| 53.1832799999983 | 631.089512382041 | 1000 | 0.392653763456140 |
| 53.2089599999983 | 631.392842425725 | 1000 | 0.392652749882558 |
| 53.2346399999983 | 631.696171031566 | 1000 | 0.392651736244956 |
| 53.2603199999983 | 631.999498202115 | 1000 | 0.392650722545276 |
| 53.2859999999983 | 632.302823938440 | 1000 | 0.392649708784385 |
| 53.3116799999983 | 632.606148235497 | 1000 | 0.392648694958719 |
| 53.3373599999983 | 632.909471097362 | 1000 | 0.392647681071324 |
| 53.3630399999983 | 633.212792520517 | 1000 | 0.392646667119744 |
| 53.3887199999983 | 633.516112505985 | 1000 | 0.392645653104814 |
| 53.4143999999983 | 633.819431051788 | 1000 | 0.392644639025197 |
| 53.4400799999983 | 634.122748157424 | 1000 | 0.392643624880625 |
| 53.4657599999983 | 634.426063826962 | 1000 | 0.392642610674129 |
| 53.4914399999983 | 634.729378052302 | 1000 | 0.392641596399958 |
| 53.5171199999983 | 635.032690840571 | 1000 | 0.392640582063348 |
| 53.5427999999983 | 635.336002188221 | 1000 | 0.392639567661834 |
| 53.5684799999983 | 635.639312093236 | 1000 | 0.392638553194058 |
| 53.5941599999983 | 635.942620555104 | 1000 | 0.392637538659746 |
| 53.6198399999983 | 636.245927574865 | 1000 | 0.392636524059747 |
| 53.6455199999983 | 636.549233150484 | 1000 | 0.392635509392689 |
| 53.6711999999982 | 636.852537282973 | 1000 | 0.392634494659400 |
| 53.6968799999983 | 637.155839971822 | 1000 | 0.392633479859608 |
| 53.7225599999982 | 637.459141218026 | 1000 | 0.392632464994126 |
| 53.7482399999983 | 637.762441018059 | 1000 | 0.392631450060516 |
| 53.7739199999982 | 638.065739371393 | 1000 | 0.392630435058500 |
| 53.7995999999983 | 638.369036279029 | 1000 | 0.392629419988893 |
| 53.8252799999982 | 638.672331738915 | 1000 | 0.392628404850323 |
| 53.8509599999982 | 638.975625752072 | 1000 | 0.392627389643621 |
| 53.8766399999982 | 639.278918319482 | 1000 | 0.392626374369590 |
| 53.9023199999982 | 639.582209436061 | 1000 | 0.392625359024687 |
| 53.9279999999982 | 639.885499104304 | 1000 | 0.392624343610801 |
| 53.9536799999982 | 640.188787323706 | 1000 | 0.392623328127669 |
| 53.9793599999982 | 640.492074095237 | 1000 | 0.392622312576086 |
| 54.0050399999982 | 640.795359413806 | 1000 | 0.392621296952512 |
| 54.0307199999982 | 641.098643280392 | 1000 | 0.392620281257749 |
| 54.0563999999982 | 641.401925697480 | 1000 | 0.392619265493677 |
| 54.0820799999982 | 641.705206663031 | 1000 | 0.392618249658939 |
| 54.1077599999982 | 642.008486174969 | 1000 | 0.392617233752156 |
| 54.1334399999982 | 642.311764234265 | 1000 | 0.392616217774125 |
| 54.1591199999982 | 642.615040838836 | 1000 | 0.392615201723463 |
| 54.1847999999982 | 642.918315989673 | 1000 | 0.392614185600982 |
| 54.2104799999982 | 643.221589684690 | 1000 | 0.392613169405299 |
| 54.2361599999982 | 643.524861926365 | 1000 | 0.392612153138285 |
| 54.2618399999982 | 643.828132712608 | 1000 | 0.392611136798553 |
| 54.2875199999982 | 644.131402039843 | 1000 | 0.392610120383666 |
| 54.3131999999982 | 644.434669912049 | 1000 | 0.392609103896561 |
| 54.3388799999982 | 644.737936325620 | 1000 | 0.392608087334779 |
| 54.3645599999982 | 645.041201281493 | 1000 | 0.392607070699092 |
| 54.3902399999982 | 645.344464776083 | 1000 | 0.392606053987062 |
| 54.4159199999982 | 645.647726813357 | 1000 | 0.392605037201615 |
| 54.4415999999982 | 645.950987389702 | 1000 | 0.392604020340291 |
| 54.4672799999982 | 646.254246509091 | 1000 | 0.392603003406017 |
| 54.4929599999982 | 646.557504164857 | 1000 | 0.392601986394174 |
| 54.5186399999982 | 646.860760356438 | 1000 | 0.392600969304474 |
| 54.5443199999982 | 647.164015089310 | 1000 | 0.392599952140904 |
| 54.5699999999982 | 647.467268358324 | 1000 | 0.392598934899930 |
| 54.5956799999982 | 647.770520162877 | 1000 | 0.392597917581233 |
| 54.6213599999982 | 648.073770503916 | 1000 | 0.392596900185594 |
| 54.6470399999982 | 648.377019382349 | 1000 | 0.392595882713766 |
| 54.6727199999982 | 648.680266791500 | 1000 | 0.392594865161143 |
| 54.6983999999982 | 648.983512738348 | 1000 | 0.392593847532765 |
| 54.7240799999982 | 649.286757219274 | 1000 | 0.392592829826188 |
| 54.7497599999982 | 649.590000230618 | 1000 | 0.392591812038943 |
| 54.7754399999982 | 649.893241774808 | 1000 | 0.392590794172855 |
| 54.8011199999982 | 650.196481849699 | 1000 | 0.392589776226525 |
| 54.8267999999982 | 650.499720457735 | 1000 | 0.392588758201790 |
| 54.8524799999982 | 650.802957593729 | 1000 | 0.392587740095109 |
| 54.8781599999982 | 651.106193260096 | 1000 | 0.392586721908299 |
| 54.9038399999982 | 651.409427456212 | 1000 | 0.392585703641034 |
| 54.9295199999982 | 651.712660181437 | 1000 | 0.392584685292978 |
| 54.9551999999982 | 652.015891436684 | 1000 | 0.392583666864888 |
| 54.9808799999982 | 652.319121215232 | 1000 | 0.392582648352158 |
| 55.0065599999982 | 652.622349524039 | 1000 | 0.392581629759793 |
| 55.0322399999982 | 652.925576353343 | 1000 | 0.392580611081055 |
| 55.0579199999982 | 653.228801713158 | 1000 | 0.392579592323090 |
| 55.0835999999982 | 653.532025595233 | 1000 | 0.392578573480227 |
| 55.1092799999982 | 653.835248003478 | 1000 | 0.392577554555324 |
| 55.1349599999982 | 654.138468932675 | 1000 | 0.392576535544845 |
| 55.1606399999982 | 654.441688385233 | 1000 | 0.392575516450597 |
| 55.1863199999982 | 654.744906362004 | 1000 | 0.392574497273293 |
| 55.2119999999982 | 655.048122856254 | 1000 | 0.392573478008343 |
| 55.2376799999982 | 655.351337873384 | 1000 | 0.392572458659645 |
| 55.2633599999982 | 655.654551411236 | 1000 | 0.392571439225810 |
| 55.2890399999982 | 655.957763467613 | 1000 | 0.392570419705424 |
| 55.3147199999982 | 656.260974044883 | 1000 | 0.392569400100260 |
| 55.3403999999982 | 656.564183137803 | 1000 | 0.392568380406783 |
| 55.3660799999982 | 656.867390750279 | 1000 | 0.392567360627838 |
| 55.3917599999982 | 657.170596877064 | 1000 | 0.392566340759891 |
| 55.4174399999982 | 657.473801520513 | 1000 | 0.392565320804706 |
| 55.4431199999982 | 657.777004679931 | 1000 | 0.392564300761922 |
| 55.4687999999982 | 658.080206353134 | 1000 | 0.392563280630143 |
| 55.4944799999982 | 658.383406540940 | 1000 | 0.392562260410062 |
| 55.5201599999982 | 658.686605241135 | 1000 | 0.392561240100265 |
| 55.5458399999982 | 658.989802457568 | 1000 | 0.392560219703551 |
| 55.5715199999982 | 659.292998181969 | 1000 | 0.392559199214298 |
| 55.5971999999982 | 659.596192419699 | 1000 | 0.392558178636357 |
| 55.6228799999982 | 659.899385165495 | 1000 | 0.392557157966202 |
| 55.6485599999982 | 660.202576423205 | 1000 | 0.392556137206630 |
| 55.6742399999982 | 660.505766190586 | 1000 | 0.392555116356210 |
| 55.6999199999982 | 660.808954463910 | 1000 | 0.392554095412487 |
| 55.7255999999982 | 661.112141243965 | 1000 | 0.392553074376137 |
| 55.7512799999982 | 661.415326534588 | 1000 | 0.392552053249945 |
| 55.7769599999982 | 661.718510327445 | 1000 | 0.392551032028271 |
| 55.8026399999982 | 662.021692627911 | 1000 | 0.392550010714965 |
| 55.8283199999982 | 662.324873433724 | 1000 | 0.392548989308590 |
| 55.8539999999982 | 662.628052742630 | 1000 | 0.392547967807719 |
| 55.8796799999982 | 662.931230555399 | 1000 | 0.392546946213016 |
| 55.9053599999982 | 663.234406868278 | 1000 | 0.392545924522020 |
| 55.9310399999982 | 663.537581685067 | 1000 | 0.392544902737488 |
| 55.9567199999982 | 663.840755003501 | 1000 | 0.392543880857986 |
| 55.9823999999982 | 664.143926821298 | 1000 | 0.392542858882076 |
| 56.0080799999982 | 664.447097136212 | 1000 | 0.392541836808340 |
| 56.0337599999982 | 664.750265953548 | 1000 | 0.392540814640572 |
| 56.0594399999982 | 665.053433266472 | 1000 | 0.392539792374195 |
| 56.0851199999982 | 665.356599077244 | 1000 | 0.392538770010902 |
| 56.1107999999982 | 665.659763383606 | 1000 | 0.392537747549274 |
| 56.1364799999981 | 665.962926186293 | 1000 | 0.392536724989952 |
| 56.1621599999982 | 666.266087484533 | 1000 | 0.392535702332542 |
| 56.1878399999982 | 666.569247276017 | 1000 | 0.392534679575594 |
| 56.2135199999982 | 666.872405561509 | 1000 | 0.392533656719769 |
| 56.2391999999981 | 667.175562338694 | 1000 | 0.392532633763616 |
| 56.2648799999981 | 667.478717606787 | 1000 | 0.392531610706734 |
| 56.2905599999981 | 667.781871368021 | 1000 | 0.392530587550796 |
| 56.3162399999981 | 668.085023617071 | 1000 | 0.392529564292287 |
| 56.3419199999981 | 668.388174356167 | 1000 | 0.392528540932876 |
| 56.3675999999981 | 668.691323584506 | 1000 | 0.392527517472155 |
| 56.3932799999981 | 668.994471299754 | 1000 | 0.392526493908664 |
| 56.4189599999981 | 669.297617504167 | 1000 | 0.392525470244089 |
| 56.4446399999981 | 669.600762190843 | 1000 | 0.392524446473849 |
| 56.4703199999981 | 669.903905366565 | 1000 | 0.392523422602727 |
| 56.4959999999981 | 670.207047022906 | 1000 | 0.392522398625104 |
| 56.5216799999981 | 670.510187166664 | 1000 | 0.392521374545769 |
| 56.5473599999981 | 670.813325790927 | 1000 | 0.392520350360143 |
| 56.5730399999981 | 671.116462897902 | 1000 | 0.392519326069879 |
| 56.5987199999981 | 671.419598485226 | 1000 | 0.392518301673510 |
| 56.6243999999981 | 671.722732555126 | 1000 | 0.392517277172698 |
| 56.6500799999981 | 672.025865102192 | 1000 | 0.392516252563902 |
| 56.6757599999981 | 672.328996127100 | 1000 | 0.392515227847729 |
| 56.7014399999981 | 672.632125630510 | 1000 | 0.392514203024777 |
| 56.7271199999981 | 672.935253610076 | 1000 | 0.392513178093596 |
| 56.7527999999981 | 673.238380067971 | 1000 | 0.392512153055811 |
| 56.7784799999981 | 673.541504998778 | 1000 | 0.392511127907887 |
| 56.8041599999981 | 673.844628407699 | 1000 | 0.392510102653510 |
| 56.8298399999981 | 674.147750286298 | 1000 | 0.392509077287094 |
| 56.8555199999981 | 674.450870636731 | 1000 | 0.392508051810257 |
| 56.8811999999981 | 674.753989461164 | 1000 | 0.392507026224620 |
| 56.9068799999981 | 675.057106755669 | 1000 | 0.392506000527667 |
| 56.9325599999981 | 675.360222522427 | 1000 | 0.392504974721029 |
| 56.9582399999981 | 675.663336754466 | 1000 | 0.392503948800131 |
| 56.9839199999981 | 675.966449456972 | 1000 | 0.392502922768642 |
| 57.0095999999981 | 676.269560626004 | 1000 | 0.392501896624045 |
| 57.0352799999981 | 676.572670260690 | 1000 | 0.392500870365902 |
| 57.0609599999981 | 676.875778361635 | 1000 | 0.392499843994780 |
| 57.0866399999981 | 677.178884926418 | 1000 | 0.392498817509193 |
| 57.1123199999981 | 677.481989955636 | 1000 | 0.392497790909702 |
| 57.1379999999981 | 677.785093448402 | 1000 | 0.392496764195864 |
| 57.1636799999981 | 678.088195400750 | 1000 | 0.392495737365154 |
| 57.1893599999981 | 678.391295814799 | 1000 | 0.392494710419162 |
| 57.2150399999981 | 678.694394688091 | 1000 | 0.392493683356387 |
| 57.2407199999981 | 678.997492022763 | 1000 | 0.392492656178429 |
| 57.2663999999981 | 679.300587813313 | 1000 | 0.392491628881734 |
| 57.2920799999981 | 679.603682061843 | 1000 | 0.392490601467881 |
| 57.3177599999981 | 679.906774765880 | 1000 | 0.392489573935364 |
| 57.3434399999981 | 680.209865927545 | 1000 | 0.392488546285770 |
| 57.3691199999981 | 680.512955541319 | 1000 | 0.392487518515545 |
| 57.3947999999981 | 680.816043607755 | 1000 | 0.392486490625225 |
| 57.4204799999981 | 681.119130128962 | 1000 | 0.392485462616389 |
| 57.4461599999981 | 681.422215096369 | 1000 | 0.392484434483439 |
| 57.4718399999981 | 681.725298519645 | 1000 | 0.392483406233037 |
| 57.4975199999981 | 682.028380391734 | 1000 | 0.392482377860605 |
| 57.5231999999981 | 682.331460711684 | 1000 | 0.392481349365669 |
| 57.5488799999981 | 682.634539476989 | 1000 | 0.392480320746713 |
| 57.5745599999981 | 682.937616691223 | 1000 | 0.392479292006302 |
| 57.6002399999981 | 683.240692350350 | 1000 | 0.392478263141893 |
| 57.6259199999981 | 683.543766453403 | 1000 | 0.392477234153008 |
| 57.6515999999981 | 683.846838999376 | 1000 | 0.392476205039139 |
| 57.6772799999981 | 684.149909990308 | 1000 | 0.392475175801821 |
| 57.7029599999981 | 684.452979422144 | 1000 | 0.392474146438508 |
| 57.7286399999981 | 684.756047292384 | 1000 | 0.392473116947695 |
| 57.7543199999981 | 685.059113603039 | 1000 | 0.392472087330901 |
| 57.7799999999981 | 685.362178354614 | 1000 | 0.392471057588631 |
| 57.8056799999981 | 685.665241541517 | 1000 | 0.392470027717320 |
| 57.8313599999981 | 685.968303165785 | 1000 | 0.392468997718501 |
| 57.8570399999981 | 686.271363223339 | 1000 | 0.392467967589624 |
| 57.8827199999981 | 686.574421716181 | 1000 | 0.392466937332199 |
| 57.9083999999981 | 686.877478641742 | 1000 | 0.392465906944686 |
| 57.9340799999981 | 687.180534000523 | 1000 | 0.392464876427593 |
| 57.9597599999981 | 687.483587791463 | 1000 | 0.392463845780387 |
| 57.9854399999981 | 687.786639911275 | 1000 | 0.392462814934485 |
| 58.0111199999981 | 688.089690559914 | 1000 | 0.392461784023203 |
| 58.0367999999981 | 688.392739636564 | 1000 | 0.392460752979575 |
| 58.0624799999981 | 688.695787240486 | 1000 | 0.392459721869771 |
| 58.0881599999981 | 688.998833170252 | 1000 | 0.392458690560023 |
| 58.1138399999981 | 689.301877524286 | 1000 | 0.392457659115976 |
| 58.1395199999981 | 689.604920201800 | 1000 | 0.392456627470892 |
| 58.1651999999981 | 689.907961402356 | 1000 | 0.392455595757477 |
| 58.1908799999981 | 690.211001024430 | 1000 | 0.392454563908478 |
| 58.2165599999981 | 690.514039066924 | 1000 | 0.392453531923347 |
| 58.2422399999981 | 690.817075528733 | 1000 | 0.392452499801533 |
| 58.2679199999981 | 691.120110408749 | 1000 | 0.392451467542485 |
| 58.2935999999981 | 691.423143705860 | 1000 | 0.392450435145648 |
| 58.3192799999981 | 691.726175418946 | 1000 | 0.392449402610466 |
| 58.3449599999981 | 692.029205546886 | 1000 | 0.392448369936381 |
| 58.3706399999981 | 692.332234088551 | 1000 | 0.392447337122832 |
| 58.3963199999981 | 692.635261042810 | 1000 | 0.392446304169255 |
| 58.4219999999981 | 692.938286408523 | 1000 | 0.392445271075085 |
| 58.4476799999981 | 693.241310184549 | 1000 | 0.392444237839754 |
| 58.4733599999981 | 693.544332369740 | 1000 | 0.392443204462693 |
| 58.4990399999981 | 693.847352962944 | 1000 | 0.392442170943329 |
| 58.5247199999981 | 694.150371963004 | 1000 | 0.392441137281087 |
| 58.5503999999981 | 694.453389368755 | 1000 | 0.392440103475391 |
| 58.5760799999981 | 694.756405179033 | 1000 | 0.392439069525662 |
| 58.6017599999980 | 695.059419392662 | 1000 | 0.392438035431317 |
| 58.6274399999981 | 695.362432008467 | 1000 | 0.392437001191774 |
| 58.6531199999981 | 695.665443025263 | 1000 | 0.392435966806446 |
| 58.6787999999980 | 695.968452441863 | 1000 | 0.392434932274745 |
| 58.7044799999980 | 696.271460257074 | 1000 | 0.392433897596079 |
| 58.7301599999980 | 696.574466469697 | 1000 | 0.392432862769856 |
| 58.7558399999980 | 696.877471078530 | 1000 | 0.392431827795479 |
| 58.7815199999980 | 697.180474082362 | 1000 | 0.392430792672352 |
| 58.8071999999980 | 697.483475479980 | 1000 | 0.392429757399872 |
| 58.8328799999980 | 697.786475270165 | 1000 | 0.392428721977438 |
| 58.8585599999980 | 698.089473451692 | 1000 | 0.392427686404445 |
| 58.8842399999980 | 698.392470023331 | 1000 | 0.392426650680284 |
| 58.9099199999980 | 698.695464983847 | 1000 | 0.392425614804345 |
| 58.9355999999980 | 698.998458332000 | 1000 | 0.392424578776016 |
| 58.9612799999980 | 699.301450066542 | 1000 | 0.392423542594682 |
| 58.9869599999980 | 699.604440186224 | 1000 | 0.392422506259726 |
| 59.0126399999980 | 699.907428689787 | 1000 | 0.392421469770527 |
| 59.0383199999980 | 700.210415575969 | 1000 | 0.392420433126462 |
| 59.0639999999980 | 700.513400843504 | 1000 | 0.392419396326908 |
| 59.0896799999980 | 700.816384491116 | 1000 | 0.392418359371236 |
| 59.1153599999980 | 701.119366517529 | 1000 | 0.392417322258817 |
| 59.1410399999980 | 701.422346921456 | 1000 | 0.392416284989017 |
| 59.1667199999980 | 701.725325701609 | 1000 | 0.392415247561203 |
| 59.1923999999980 | 702.028302856690 | 1000 | 0.392414209974736 |
| 59.2180799999980 | 702.331278385401 | 1000 | 0.392413172228976 |
| 59.2437599999980 | 702.634252286432 | 1000 | 0.392412134323280 |
| 59.2694399999980 | 702.937224558473 | 1000 | 0.392411096257004 |
| 59.2951199999980 | 703.240195200203 | 1000 | 0.392410058029498 |
| 59.3207999999980 | 703.543164210301 | 1000 | 0.392409019640114 |
| 59.3464799999980 | 703.846131587435 | 1000 | 0.392407981088197 |
| 59.3721599999980 | 704.149097330271 | 1000 | 0.392406942373091 |
| 59.3978399999980 | 704.452061437467 | 1000 | 0.392405903494140 |
| 59.4235199999980 | 704.755023907675 | 1000 | 0.392404864450680 |
| 59.4491999999980 | 705.057984739544 | 1000 | 0.392403825242050 |
| 59.4748799999980 | 705.360943931713 | 1000 | 0.392402785867582 |
| 59.5005599999980 | 705.663901482819 | 1000 | 0.392401746326607 |
| 59.5262399999980 | 705.966857391491 | 1000 | 0.392400706618455 |
| 59.5519199999980 | 706.269811656351 | 1000 | 0.392399666742449 |
| 59.5775999999980 | 706.572764276018 | 1000 | 0.392398626697914 |
| 59.6032799999980 | 706.875715249102 | 1000 | 0.392397586484170 |
| 59.6289599999980 | 707.178664574210 | 1000 | 0.392396546100533 |
| 59.6546399999980 | 707.481612249940 | 1000 | 0.392395505546320 |
| 59.6803199999980 | 707.784558274886 | 1000 | 0.392394464820840 |
| 59.7059999999980 | 708.087502647634 | 1000 | 0.392393423923405 |
| 59.7316799999980 | 708.390445366766 | 1000 | 0.392392382853320 |
| 59.7573599999980 | 708.693386430857 | 1000 | 0.392391341609889 |
| 59.7830399999980 | 708.996325838475 | 1000 | 0.392390300192413 |
| 59.8087199999980 | 709.299263588182 | 1000 | 0.392389258600190 |
| 59.8343999999980 | 709.602199678535 | 1000 | 0.392388216832515 |
| 59.8600799999980 | 709.905134108084 | 1000 | 0.392387174888681 |
| 59.8857599999980 | 710.208066875372 | 1000 | 0.392386132767977 |
| 59.9114399999980 | 710.510997978937 | 1000 | 0.392385090469690 |
| 59.9371199999980 | 710.813927417309 | 1000 | 0.392384047993103 |
| 59.9627999999980 | 711.116855189013 | 1000 | 0.392383005337498 |
| 59.9884799999980 | 711.419781292568 | 1000 | 0.392381962502153 |
| 60.0141599999980 | 711.722705726484 | 1000 | 0.392380919486343 |
| 60.0398399999980 | 712.025628489268 | 1000 | 0.392379876289340 |
| 60.0655199999980 | 712.328549579418 | 1000 | 0.392378832910414 |
| 60.0911999999980 | 712.631468995426 | 1000 | 0.392377789348831 |
| 60.1168799999980 | 712.934386735778 | 1000 | 0.392376745603855 |
| 60.1425599999980 | 713.237302798954 | 1000 | 0.392375701674746 |
| 60.1682399999980 | 713.540217183425 | 1000 | 0.392374657560761 |
| 60.1939199999980 | 713.843129887657 | 1000 | 0.392373613261156 |
| 60.2195999999980 | 714.146040910111 | 1000 | 0.392372568775182 |
| 60.2452799999980 | 714.448950249238 | 1000 | 0.392371524102087 |
| 60.2709599999980 | 714.751857903485 | 1000 | 0.392370479241118 |
| 60.2966399999980 | 715.054763871290 | 1000 | 0.392369434191516 |
| 60.3223199999980 | 715.357668151087 | 1000 | 0.392368388952521 |
| 60.3479999999980 | 715.660570741299 | 1000 | 0.392367343523370 |
| 60.3736799999980 | 715.963471640347 | 1000 | 0.392366297903296 |
| 60.3993599999980 | 716.266370846642 | 1000 | 0.392365252091529 |
| 60.4250399999980 | 716.569268358589 | 1000 | 0.392364206087297 |
| 60.4507199999980 | 716.872164174586 | 1000 | 0.392363159889823 |
| 60.4763999999980 | 717.175058293024 | 1000 | 0.392362113498329 |
| 60.5020799999980 | 717.477950712288 | 1000 | 0.392361066912033 |
| 60.5277599999980 | 717.780841430754 | 1000 | 0.392360020130149 |
| 60.5534399999980 | 718.083730446793 | 1000 | 0.392358973151889 |
| 60.5791199999980 | 718.386617758767 | 1000 | 0.392357925976462 |
| 60.6047999999980 | 718.689503365032 | 1000 | 0.392356878603072 |
| 60.6304799999980 | 718.992387263938 | 1000 | 0.392355831030922 |
| 60.6561599999980 | 719.295269453825 | 1000 | 0.392354783259210 |
| 60.6818399999980 | 719.598149933029 | 1000 | 0.392353735287133 |
| 60.7075199999980 | 719.901028699876 | 1000 | 0.392352687113882 |
| 60.7331999999980 | 720.203905752687 | 1000 | 0.392351638738647 |
| 60.7588799999980 | 720.506781089773 | 1000 | 0.392350590160614 |
| 60.7845599999980 | 720.809654709441 | 1000 | 0.392349541378965 |
| 60.8102399999980 | 721.112526609988 | 1000 | 0.392348492392880 |
| 60.8359199999980 | 721.415396789705 | 1000 | 0.392347443201535 |
| 60.8615999999980 | 721.718265246876 | 1000 | 0.392346393804102 |
| 60.8872799999980 | 722.021131979775 | 1000 | 0.392345344199752 |
| 60.9129599999980 | 722.323996986671 | 1000 | 0.392344294387650 |
| 60.9386399999980 | 722.626860265825 | 1000 | 0.392343244366958 |
| 60.9643199999980 | 722.929721815490 | 1000 | 0.392342194136838 |
| 60.9899999999980 | 723.232581633912 | 1000 | 0.392341143696443 |
| 61.0156799999980 | 723.535439719329 | 1000 | 0.392340093044928 |
| 61.0413599999980 | 723.838296069971 | 1000 | 0.392339042181442 |
| 61.0670399999979 | 724.141150684061 | 1000 | 0.392337991105129 |
| 61.0927199999980 | 724.444003559814 | 1000 | 0.392336939815133 |
| 61.1183999999980 | 724.746854695437 | 1000 | 0.392335888310593 |
| 61.1440799999979 | 725.049704089130 | 1000 | 0.392334836590644 |
| 61.1697599999979 | 725.352551739084 | 1000 | 0.392333784654418 |
| 61.1954399999979 | 725.655397643483 | 1000 | 0.392332732501044 |
| 61.2211199999979 | 725.958241800504 | 1000 | 0.392331680129646 |
| 61.2467999999979 | 726.261084208314 | 1000 | 0.392330627539347 |
| 61.2724799999979 | 726.563924865073 | 1000 | 0.392329574729264 |
| 61.2981599999979 | 726.866763768934 | 1000 | 0.392328521698513 |
| 61.3238399999979 | 727.169600918041 | 1000 | 0.392327468446203 |
| 61.3495199999979 | 727.472436310530 | 1000 | 0.392326414971442 |
| 61.3751999999979 | 727.775269944529 | 1000 | 0.392325361273334 |
| 61.4008799999979 | 728.078101818158 | 1000 | 0.392324307350979 |
| 61.4265599999979 | 728.380931929528 | 1000 | 0.392323253203474 |
| 61.4522399999979 | 728.683760276743 | 1000 | 0.392322198829912 |
| 61.4779199999979 | 728.986586857899 | 1000 | 0.392321144229382 |
| 61.5035999999979 | 729.289411671083 | 1000 | 0.392320089400969 |
| 61.5292799999979 | 729.592234714373 | 1000 | 0.392319034343756 |
| 61.5549599999979 | 729.895055985840 | 1000 | 0.392317979056821 |
| 61.5806399999979 | 730.197875483546 | 1000 | 0.392316923539238 |
| 61.6063199999979 | 730.500693205545 | 1000 | 0.392315867790079 |
| 61.6319999999979 | 730.803509149881 | 1000 | 0.392314811808410 |
| 61.6576799999979 | 731.106323314594 | 1000 | 0.392313755593296 |
| 61.6833599999979 | 731.409135697709 | 1000 | 0.392312699143795 |
| 61.7090399999979 | 731.711946297247 | 1000 | 0.392311642458963 |
| 61.7347199999979 | 732.014755111219 | 1000 | 0.392310585537853 |
| 61.7603999999979 | 732.317562137629 | 1000 | 0.392309528379513 |
| 61.7860799999979 | 732.620367374469 | 1000 | 0.392308470982988 |
| 61.8117599999979 | 732.923170819725 | 1000 | 0.392307413347317 |
| 61.8374399999979 | 733.225972471373 | 1000 | 0.392306355471538 |
| 61.8631199999979 | 733.528772327382 | 1000 | 0.392305297354684 |
| 61.8887999999979 | 733.831570385709 | 1000 | 0.392304238995784 |
| 61.9144799999979 | 734.134366644306 | 1000 | 0.392303180393863 |
| 61.9401599999979 | 734.437161101113 | 1000 | 0.392302121547941 |
| 61.9658399999979 | 734.739953754063 | 1000 | 0.392301062457037 |
| 61.9915199999979 | 735.042744601079 | 1000 | 0.392300003120164 |
| 62.0171999999979 | 735.345533640075 | 1000 | 0.392298943536331 |
| 62.0428799999979 | 735.648320868957 | 1000 | 0.392297883704544 |
| 62.0685599999979 | 735.951106285621 | 1000 | 0.392296823623803 |
| 62.0942399999979 | 736.253889887953 | 1000 | 0.392295763293107 |
| 62.1199199999979 | 736.556671673833 | 1000 | 0.392294702711449 |
| 62.1455999999979 | 736.859451641128 | 1000 | 0.392293641877818 |
| 62.1712799999979 | 737.162229787699 | 1000 | 0.392292580791200 |
| 62.1969599999979 | 737.465006111395 | 1000 | 0.392291519450575 |
| 62.2226399999979 | 737.767780610057 | 1000 | 0.392290457854921 |
| 62.2483199999979 | 738.070553281517 | 1000 | 0.392289396003211 |
| 62.2739999999979 | 738.373324123598 | 1000 | 0.392288333894414 |
| 62.2996799999979 | 738.676093134111 | 1000 | 0.392287271527494 |
| 62.3253599999979 | 738.978860310861 | 1000 | 0.392286208901413 |
| 62.3510399999979 | 739.281625651641 | 1000 | 0.392285146015126 |
| 62.3767199999979 | 739.584389154235 | 1000 | 0.392284082867586 |
| 62.4023999999979 | 739.887150816418 | 1000 | 0.392283019457741 |
| 62.4280799999979 | 740.189910635955 | 1000 | 0.392281955784535 |
| 62.4537599999979 | 740.492668610601 | 1000 | 0.392280891846907 |
| 62.4794399999979 | 740.795424738103 | 1000 | 0.392279827643793 |
| 62.5051199999979 | 741.098179016195 | 1000 | 0.392278763174124 |
| 62.5307999999979 | 741.400931442603 | 1000 | 0.392277698436826 |
| 62.5564799999979 | 741.703682015045 | 1000 | 0.392276633430822 |
| 62.5821599999979 | 742.006430731226 | 1000 | 0.392275568155031 |
| 62.6078399999979 | 742.309177588843 | 1000 | 0.392274502608365 |
| 62.6335199999979 | 742.611922585581 | 1000 | 0.392273436789735 |
| 62.6591999999979 | 742.914665719118 | 1000 | 0.392272370698045 |
| 62.6848799999979 | 743.217406987120 | 1000 | 0.392271304332196 |
| 62.7105599999979 | 743.520146387242 | 1000 | 0.392270237691085 |
| 62.7362399999979 | 743.822883917132 | 1000 | 0.392269170773603 |
| 62.7619199999979 | 744.125619574425 | 1000 | 0.392268103578638 |
| 62.7875999999979 | 744.428353356746 | 1000 | 0.392267036105072 |
| 62.8132799999979 | 744.731085261711 | 1000 | 0.392265968351785 |
| 62.8389599999979 | 745.033815286925 | 1000 | 0.392264900317649 |
| 62.8646399999979 | 745.336543429982 | 1000 | 0.392263832001534 |
| 62.8903199999979 | 745.639269688468 | 1000 | 0.392262763402306 |
| 62.9159999999979 | 745.941994059955 | 1000 | 0.392261694518824 |
| 62.9416799999979 | 746.244716542007 | 1000 | 0.392260625349944 |
| 62.9673599999979 | 746.547437132177 | 1000 | 0.392259555894518 |
| 62.9930399999979 | 746.850155828007 | 1000 | 0.392258486151392 |
| 63.0187199999979 | 747.152872627028 | 1000 | 0.392257416119407 |
| 63.0443999999979 | 747.455587526761 | 1000 | 0.392256345797402 |
| 63.0700799999979 | 747.758300524717 | 1000 | 0.392255275184209 |
| 63.0957599999979 | 748.061011618395 | 1000 | 0.392254204278655 |
| 63.1214399999979 | 748.363720805283 | 1000 | 0.392253133079564 |
| 63.1471199999979 | 748.666428082859 | 1000 | 0.392252061585754 |
| 63.1727999999979 | 748.969133448590 | 1000 | 0.392250989796040 |
| 63.1984799999979 | 749.271836899931 | 1000 | 0.392249917709229 |
| 63.2241599999979 | 749.574538434327 | 1000 | 0.392248845324127 |
| 63.2498399999979 | 749.877238049212 | 1000 | 0.392247772639532 |
| 63.2755199999979 | 750.179935742008 | 1000 | 0.392246699654240 |
| 63.3011999999979 | 750.482631510128 | 1000 | 0.392245626367040 |
| 63.3268799999979 | 750.785325350970 | 1000 | 0.392244552776716 |
| 63.3525599999979 | 751.088017261925 | 1000 | 0.392243478882050 |
| 63.3782399999979 | 751.390707240370 | 1000 | 0.392242404681815 |
| 63.4039199999979 | 751.693395283671 | 1000 | 0.392241330174783 |
| 63.4295999999979 | 751.996081389183 | 1000 | 0.392240255359719 |
| 63.4552799999979 | 752.298765554250 | 1000 | 0.392239180235382 |
| 63.4809599999979 | 752.601447776203 | 1000 | 0.392238104800528 |
| 63.5066399999979 | 752.904128052364 | 1000 | 0.392237029053908 |
| 63.5323199999978 | 753.206806380040 | 1000 | 0.392235952994267 |
| 63.5579999999979 | 753.509482756530 | 1000 | 0.392234876620345 |
| 63.5836799999979 | 753.812157179119 | 1000 | 0.392233799930877 |
| 63.6093599999978 | 754.114829645081 | 1000 | 0.392232722924593 |
| 63.6350399999978 | 754.417500151677 | 1000 | 0.392231645600220 |
| 63.6607199999978 | 754.720168696158 | 1000 | 0.392230567956476 |
| 63.6863999999978 | 755.022835275762 | 1000 | 0.392229489992076 |
| 63.7120799999978 | 755.325499887716 | 1000 | 0.392228411705730 |
| 63.7377599999978 | 755.628162529233 | 1000 | 0.392227333096142 |
| 63.7634399999978 | 755.930823197517 | 1000 | 0.392226254162013 |
| 63.7891199999978 | 756.233481889756 | 1000 | 0.392225174902035 |
| 63.8147999999978 | 756.536138603130 | 1000 | 0.392224095314898 |
| 63.8404799999978 | 756.838793334804 | 1000 | 0.392223015399284 |
| 63.8661599999978 | 757.141446081932 | 1000 | 0.392221935153874 |
| 63.8918399999978 | 757.444096841655 | 1000 | 0.392220854577338 |
| 63.9175199999978 | 757.746745611102 | 1000 | 0.392219773668346 |
| 63.9431999999978 | 758.049392387389 | 1000 | 0.392218692425559 |
| 63.9688799999978 | 758.352037167620 | 1000 | 0.392217610847634 |
| 63.9945599999978 | 758.654679948887 | 1000 | 0.392216528933223 |
| 64.0202399999978 | 758.957320728268 | 1000 | 0.392215446680972 |
| 64.0459199999978 | 759.259959502831 | 1000 | 0.392214364089522 |
| 64.0715999999978 | 759.562596269628 | 1000 | 0.392213281157507 |
| 64.0972799999978 | 759.865231025701 | 1000 | 0.392212197883557 |
| 64.1229599999978 | 760.167863768077 | 1000 | 0.392211114266298 |
| 64.1486399999978 | 760.470494493772 | 1000 | 0.392210030304347 |
| 64.1743199999978 | 760.773123199787 | 1000 | 0.392208945996318 |
| 64.1999999999978 | 761.075749883113 | 1000 | 0.392207861340818 |
| 64.2256799999978 | 761.378374540726 | 1000 | 0.392206776336449 |
| 64.2513599999978 | 761.680997169588 | 1000 | 0.392205690981808 |
| 64.2770399999978 | 761.983617766651 | 1000 | 0.392204605275486 |
| 64.3027199999978 | 762.286236328850 | 1000 | 0.392203519216068 |
| 64.3283999999978 | 762.588852853110 | 1000 | 0.392202432802133 |
| 64.3540799999978 | 762.891467336340 | 1000 | 0.392201346032254 |
| 64.3797599999978 | 763.194079775439 | 1000 | 0.392200258905001 |
| 64.4054399999978 | 763.496690167289 | 1000 | 0.392199171418935 |
| 64.4311199999978 | 763.799298508761 | 1000 | 0.392198083572613 |
| 64.4567999999978 | 764.101904796712 | 1000 | 0.392196995364585 |
| 64.4824799999978 | 764.404509027984 | 1000 | 0.392195906793396 |
| 64.5081599999978 | 764.707111199406 | 1000 | 0.392194817857586 |
| 64.5338399999978 | 765.009711307795 | 1000 | 0.392193728555688 |
| 64.5595199999978 | 765.312309349953 | 1000 | 0.392192638886229 |
| 64.5851999999978 | 765.614905322667 | 1000 | 0.392191548847730 |
| 64.6108799999978 | 765.917499222711 | 1000 | 0.392190458438707 |
| 64.6365599999978 | 766.220091046846 | 1000 | 0.392189367657669 |
| 64.6622399999978 | 766.522680791819 | 1000 | 0.392188276503120 |
| 64.6879199999978 | 766.825268454361 | 1000 | 0.392187184973558 |
| 64.7135999999978 | 767.127854031191 | 1000 | 0.392186093067473 |
| 64.7392799999978 | 767.430437519011 | 1000 | 0.392185000783352 |
| 64.7649599999978 | 767.733018914513 | 1000 | 0.392183908119674 |
| 64.7906399999978 | 768.035598214371 | 1000 | 0.392182815074912 |
| 64.8163199999978 | 768.338175415246 | 1000 | 0.392181721647533 |
| 64.8419999999978 | 768.640750513786 | 1000 | 0.392180627835998 |
| 64.8676799999978 | 768.943323506621 | 1000 | 0.392179533638762 |
| 64.8933599999978 | 769.245894390369 | 1000 | 0.392178439054273 |
| 64.9190399999978 | 769.548463161634 | 1000 | 0.392177344080974 |
| 64.9447199999978 | 769.851029817003 | 1000 | 0.392176248717302 |
| 64.9703999999978 | 770.153594353051 | 1000 | 0.392175152961685 |
| 64.9960799999978 | 770.456156766334 | 1000 | 0.392174056812548 |
| 65.0217599999978 | 770.758717053399 | 1000 | 0.392172960268307 |
| 65.0474399999978 | 771.061275210772 | 1000 | 0.392171863327374 |
| 65.0731199999978 | 771.363831234969 | 1000 | 0.392170765988153 |
| 65.0987999999978 | 771.666385122487 | 1000 | 0.392169668249043 |
| 65.1244799999978 | 771.968936869811 | 1000 | 0.392168570108434 |
| 65.1501599999978 | 772.271486473409 | 1000 | 0.392167471564712 |
| 65.1758399999978 | 772.574033929735 | 1000 | 0.392166372616255 |
| 65.2015199999978 | 772.876579235226 | 1000 | 0.392165273261437 |
| 65.2271999999978 | 773.179122386304 | 1000 | 0.392164173498622 |
| 65.2528799999978 | 773.481663379377 | 1000 | 0.392163073326170 |
| 65.2785599999978 | 773.784202210837 | 1000 | 0.392161972742434 |
| 65.3042399999978 | 774.086738877059 | 1000 | 0.392160871745758 |
| 65.3299199999978 | 774.389273374403 | 1000 | 0.392159770334484 |
| 65.3555999999978 | 774.691805699214 | 1000 | 0.392158668506942 |
| 65.3812799999978 | 774.994335847822 | 1000 | 0.392157566261459 |
| 65.4069599999978 | 775.296863816538 | 1000 | 0.392156463596355 |
| 65.4326399999978 | 775.599389601660 | 1000 | 0.392155360509942 |
| 65.4583199999978 | 775.901913199469 | 1000 | 0.392154257000525 |
| 65.4839999999978 | 776.204434606229 | 1000 | 0.392153153066403 |
| 65.5096799999978 | 776.506953818190 | 1000 | 0.392152048705869 |
| 65.5353599999978 | 776.809470831585 | 1000 | 0.392150943917208 |
| 65.5610399999978 | 777.111985642629 | 1000 | 0.392149838698698 |
| 65.5867199999978 | 777.414498247523 | 1000 | 0.392148733048610 |
| 65.6123999999978 | 777.717008642449 | 1000 | 0.392147626965210 |
| 65.6380799999978 | 778.019516823577 | 1000 | 0.392146520446753 |
| 65.6637599999978 | 778.322022787055 | 1000 | 0.392145413491492 |
| 65.6894399999978 | 778.624526529017 | 1000 | 0.392144306097670 |
| 65.7151199999978 | 778.927028045582 | 1000 | 0.392143198263522 |
| 65.7407999999978 | 779.229527332849 | 1000 | 0.392142089987279 |
| 65.7664799999978 | 779.532024386903 | 1000 | 0.392140981267162 |
| 65.7921599999978 | 779.834519203809 | 1000 | 0.392139872101387 |
| 65.8178399999978 | 780.137011779618 | 1000 | 0.392138762488163 |
| 65.8435199999978 | 780.439502110362 | 1000 | 0.392137652425688 |
| 65.8691999999978 | 780.741990192057 | 1000 | 0.392136541912158 |
| 65.8948799999978 | 781.044476020701 | 1000 | 0.392135430945759 |
| 65.9205599999978 | 781.346959592276 | 1000 | 0.392134319524669 |
| 65.9462399999977 | 781.649440902745 | 1000 | 0.392133207647060 |
| 65.9719199999977 | 781.951919948054 | 1000 | 0.392132095311097 |
| 65.9975999999978 | 782.254396724132 | 1000 | 0.392130982514937 |
| 66.0232799999978 | 782.556871226890 | 1000 | 0.392129869256729 |
| 66.0489599999978 | 782.859343452223 | 1000 | 0.392128755534616 |
| 66.0746399999977 | 783.161813396005 | 1000 | 0.392127641346732 |
| 66.1003199999977 | 783.464281054095 | 1000 | 0.392126526691204 |
| 66.1259999999977 | 783.766746422333 | 1000 | 0.392125411566153 |
| 66.1516799999977 | 784.069209496541 | 1000 | 0.392124295969691 |
| 66.1773599999977 | 784.371670272524 | 1000 | 0.392123179899922 |
| 66.2030399999977 | 784.674128746067 | 1000 | 0.392122063354943 |
| 66.2287199999977 | 784.976584912939 | 1000 | 0.392120946332843 |
| 66.2543999999977 | 785.279038768888 | 1000 | 0.392119828831705 |
| 66.2800799999977 | 785.581490309647 | 1000 | 0.392118710849603 |
| 66.3057599999977 | 785.883939530927 | 1000 | 0.392117592384602 |
| 66.3314399999977 | 786.186386428424 | 1000 | 0.392116473434761 |
| 66.3571199999977 | 786.488830997813 | 1000 | 0.392115353998131 |
| 66.3827999999977 | 786.791273234752 | 1000 | 0.392114234072755 |
| 66.4084799999977 | 787.093713134878 | 1000 | 0.392113113656668 |
| 66.4341599999977 | 787.396150693811 | 1000 | 0.392111992747897 |
| 66.4598399999977 | 787.698585907152 | 1000 | 0.392110871344462 |
| 66.4855199999977 | 788.001018770482 | 1000 | 0.392109749444373 |
| 66.5111999999977 | 788.303449279364 | 1000 | 0.392108627045634 |
| 66.5368799999977 | 788.605877429341 | 1000 | 0.392107504146241 |
| 66.5625599999977 | 788.908303215938 | 1000 | 0.392106380744180 |
| 66.5882399999977 | 789.210726634659 | 1000 | 0.392105256837431 |
| 66.6139199999977 | 789.513147680991 | 1000 | 0.392104132423965 |
| 66.6395999999977 | 789.815566350398 | 1000 | 0.392103007501745 |
| 66.6652799999977 | 790.117982638328 | 1000 | 0.392101882068725 |
| 66.6909599999977 | 790.420396540206 | 1000 | 0.392100756122853 |
| 66.7166399999977 | 790.722808051441 | 1000 | 0.392099629662065 |
| 66.7423199999977 | 791.025217167419 | 1000 | 0.392098502684294 |
| 66.7679999999977 | 791.327623883508 | 1000 | 0.392097375187460 |
| 66.7936799999977 | 791.630028195055 | 1000 | 0.392096247169477 |
| 66.8193599999977 | 791.932430097388 | 1000 | 0.392095118628249 |
| 66.8450399999977 | 792.234829585813 | 1000 | 0.392093989561674 |
| 66.8707199999977 | 792.537226655617 | 1000 | 0.392092859967640 |
| 66.8963999999977 | 792.839621302067 | 1000 | 0.392091729844025 |
| 66.9220799999977 | 793.142013520409 | 1000 | 0.392090599188702 |
| 66.9477599999977 | 793.444403305869 | 1000 | 0.392089467999534 |
| 66.9734399999977 | 793.746790653652 | 1000 | 0.392088336274373 |
| 66.9991199999977 | 794.049175558942 | 1000 | 0.392087204011066 |
| 67.0247999999977 | 794.351558016902 | 1000 | 0.392086071207450 |
| 67.0504799999977 | 794.653938022676 | 1000 | 0.392084937861352 |
| 67.0761599999977 | 794.956315571384 | 1000 | 0.392083803970592 |
| 67.1018399999977 | 795.258690658129 | 1000 | 0.392082669532981 |
| 67.1275199999977 | 795.561063277988 | 1000 | 0.392081534546320 |
| 67.1531999999977 | 795.863433426022 | 1000 | 0.392080399008403 |
| 67.1788799999977 | 796.165801097266 | 1000 | 0.392079262917014 |
| 67.2045599999977 | 796.468166286736 | 1000 | 0.392078126269928 |
| 67.2302399999977 | 796.770528989428 | 1000 | 0.392076989064912 |
| 67.2559199999977 | 797.072889200312 | 1000 | 0.392075851299723 |
| 67.2815999999977 | 797.375246914340 | 1000 | 0.392074712972109 |
| 67.3072799999977 | 797.677602126441 | 1000 | 0.392073574079810 |
| 67.3329599999977 | 797.979954831522 | 1000 | 0.392072434620556 |
| 67.3586399999977 | 798.282305024469 | 1000 | 0.392071294592068 |
| 67.3843199999977 | 798.584652700144 | 1000 | 0.392070153992059 |
| 67.4099999999977 | 798.886997853390 | 1000 | 0.392069012818231 |
| 67.4356799999977 | 799.189340479024 | 1000 | 0.392067871068278 |
| 67.4613599999977 | 799.491680571843 | 1000 | 0.392066728739884 |
| 67.4870399999977 | 799.794018126621 | 1000 | 0.392065585830725 |
| 67.5127199999977 | 800.096353138110 | 1000 | 0.392064442338466 |
| 67.5383999999977 | 800.398685601038 | 1000 | 0.392063298260764 |
| 67.5640799999977 | 800.701015510112 | 1000 | 0.392062153595265 |
| 67.5897599999977 | 801.003342860015 | 1000 | 0.392061008339608 |
| 67.6154399999977 | 801.305667645407 | 1000 | 0.392059862491420 |
| 67.6411199999977 | 801.607989860926 | 1000 | 0.392058716048320 |
| 67.6667999999977 | 801.910309501186 | 1000 | 0.392057569007916 |
| 67.6924799999977 | 802.212626560778 | 1000 | 0.392056421367809 |
| 67.7181599999977 | 802.514941034269 | 1000 | 0.392055273125589 |
| 67.7438399999977 | 802.817252916205 | 1000 | 0.392054124278834 |
| 67.7695199999977 | 803.119562201105 | 1000 | 0.392052974825116 |
| 67.7951999999977 | 803.421868883468 | 1000 | 0.392051824761996 |
| 67.8208799999977 | 803.724172957765 | 1000 | 0.392050674087024 |
| 67.8465599999977 | 804.026474418448 | 1000 | 0.392049522797741 |
| 67.8722399999977 | 804.328773259942 | 1000 | 0.392048370891678 |
| 67.8979199999977 | 804.631069476647 | 1000 | 0.392047218366357 |
| 67.9235999999977 | 804.933363062943 | 1000 | 0.392046065219289 |
| 67.9492799999977 | 805.235654013182 | 1000 | 0.392044911447975 |
| 67.9749599999977 | 805.537942321693 | 1000 | 0.392043757049907 |
| 68.0006399999977 | 805.840227982781 | 1000 | 0.392042602022566 |
| 68.0263199999977 | 806.142510990725 | 1000 | 0.392041446363422 |
| 68.0519999999977 | 806.444791339781 | 1000 | 0.392040290069938 |
| 68.0776799999977 | 806.747069024180 | 1000 | 0.392039133139563 |
| 68.1033599999977 | 807.049344038127 | 1000 | 0.392037975569739 |
| 68.1290399999977 | 807.351616375802 | 1000 | 0.392036817357895 |
| 68.1547199999977 | 807.653886031362 | 1000 | 0.392035658501451 |
| 68.1803999999977 | 807.956152998937 | 1000 | 0.392034498997818 |
| 68.2060799999977 | 808.258417272632 | 1000 | 0.392033338844394 |
| 68.2317599999977 | 808.560678846527 | 1000 | 0.392032178038569 |
| 68.2574399999977 | 808.862937714676 | 1000 | 0.392031016577719 |
| 68.2831199999977 | 809.165193871108 | 1000 | 0.392029854459214 |
| 68.3087999999977 | 809.467447309825 | 1000 | 0.392028691680411 |
| 68.3344799999977 | 809.769698024805 | 1000 | 0.392027528238656 |
| 68.3601599999977 | 810.071946009999 | 1000 | 0.392026364131285 |
| 68.3858399999976 | 810.374191259331 | 1000 | 0.392025199355623 |
| 68.4115199999976 | 810.676433766701 | 1000 | 0.392024033908985 |
| 68.4371999999977 | 810.978673525982 | 1000 | 0.392022867788675 |
| 68.4628799999977 | 811.280910531018 | 1000 | 0.392021700991985 |
| 68.4885599999977 | 811.583144775631 | 1000 | 0.392020533516198 |
| 68.5142399999976 | 811.885376253612 | 1000 | 0.392019365358586 |
| 68.5399199999977 | 812.187604958729 | 1000 | 0.392018196516407 |
| 68.5655999999976 | 812.489830884720 | 1000 | 0.392017026986912 |
| 68.5912799999976 | 812.792054025299 | 1000 | 0.392015856767338 |
| 68.6169599999976 | 813.094274374150 | 1000 | 0.392014685854914 |
| 68.6426399999976 | 813.396491924931 | 1000 | 0.392013514246853 |
| 68.6683199999977 | 813.698706671275 | 1000 | 0.392012341940363 |
| 68.6939999999976 | 814.000918606783 | 1000 | 0.392011168932635 |
| 68.7196799999976 | 814.303127725032 | 1000 | 0.392009995220853 |
| 68.7453599999976 | 814.605334019570 | 1000 | 0.392008820802188 |
| 68.7710399999976 | 814.907537483917 | 1000 | 0.392007645673799 |
| 68.7967199999976 | 815.209738111566 | 1000 | 0.392006469832834 |
| 68.8223999999976 | 815.511935895981 | 1000 | 0.392005293276431 |
| 68.8480799999976 | 815.814130830599 | 1000 | 0.392004116001714 |
| 68.8737599999976 | 816.116322908827 | 1000 | 0.392002938005798 |
| 68.8994399999976 | 816.418512124045 | 1000 | 0.392001759285784 |
| 68.9251199999976 | 816.720698469605 | 1000 | 0.392000579838764 |
| 68.9507999999976 | 817.022881938829 | 1000 | 0.391999399661816 |
| 68.9764799999976 | 817.325062525010 | 1000 | 0.391998218752008 |
| 69.0021599999976 | 817.627240221413 | 1000 | 0.391997037106394 |
| 69.0278399999976 | 817.929415021274 | 1000 | 0.391995854722019 |
| 69.0535199999976 | 818.231586917801 | 1000 | 0.391994671595915 |
| 69.0791999999976 | 818.533755904169 | 1000 | 0.391993487725100 |
| 69.1048799999976 | 818.835921973528 | 1000 | 0.391992303106584 |
| 69.1305599999976 | 819.138085118996 | 1000 | 0.391991117737361 |
| 69.1562399999976 | 819.440245333662 | 1000 | 0.391989931614416 |
| 69.1819199999976 | 819.742402610585 | 1000 | 0.391988744734721 |
| 69.2075999999976 | 820.044556942793 | 1000 | 0.391987557095234 |
| 69.2332799999976 | 820.346708323288 | 1000 | 0.391986368692904 |
| 69.2589599999976 | 820.648856745037 | 1000 | 0.391985179524666 |
| 69.2846399999976 | 820.951002200980 | 1000 | 0.391983989587442 |
| 69.3103199999976 | 821.253144684024 | 1000 | 0.391982798878143 |
| 69.3359999999976 | 821.555284187049 | 1000 | 0.391981607393667 |
| 69.3616799999976 | 821.857420702900 | 1000 | 0.391980415130899 |
| 69.3873599999976 | 822.159554224395 | 1000 | 0.391979222086713 |
| 69.4130399999976 | 822.461684744319 | 1000 | 0.391978028257969 |
| 69.4387199999976 | 822.763812255427 | 1000 | 0.391976833641515 |
| 69.4643999999976 | 823.065936750441 | 1000 | 0.391975638234187 |
| 69.4900799999976 | 823.368058222054 | 1000 | 0.391974442032807 |
| 69.5157599999976 | 823.670176662926 | 1000 | 0.391973245034186 |
| 69.5414399999976 | 823.972292065686 | 1000 | 0.391972047235119 |
| 69.5671199999976 | 824.274404422931 | 1000 | 0.391970848632392 |
| 69.5927999999976 | 824.576513727226 | 1000 | 0.391969649222776 |
| 69.6184799999976 | 824.878619971105 | 1000 | 0.391968449003029 |
| 69.6441599999976 | 825.180723147069 | 1000 | 0.391967247969897 |
| 69.6698399999976 | 825.482823247587 | 1000 | 0.391966046120111 |
| 69.6955199999976 | 825.784920265095 | 1000 | 0.391964843450391 |
| 69.7211999999976 | 826.087014191996 | 1000 | 0.391963639957444 |
| 69.7468799999976 | 826.389105020663 | 1000 | 0.391962435637961 |
| 69.7725599999976 | 826.691192743434 | 1000 | 0.391961230488623 |
| 69.7982399999976 | 826.993277352613 | 1000 | 0.391960024506095 |
| 69.8239199999976 | 827.295358840473 | 1000 | 0.391958817687031 |
| 69.8495999999976 | 827.597437199253 | 1000 | 0.391957610028069 |
| 69.8752799999976 | 827.899512421159 | 1000 | 0.391956401525837 |
| 69.9009599999976 | 828.201584498363 | 1000 | 0.391955192176946 |
| 69.9266399999976 | 828.503653423003 | 1000 | 0.391953981977994 |
| 69.9523199999976 | 828.805719187183 | 1000 | 0.391952770925568 |
| 69.9779999999976 | 829.107781782974 | 1000 | 0.391951559016238 |
| 70.0036799999976 | 829.409841202413 | 1000 | 0.391950346246563 |
| 70.0293599999976 | 829.711897437502 | 1000 | 0.391949132613086 |
| 70.0550399999976 | 830.013950480208 | 1000 | 0.391947918112337 |
| 70.0807199999976 | 830.316000322464 | 1000 | 0.391946702740832 |
| 70.1063999999976 | 830.618046956170 | 1000 | 0.391945486495074 |
| 70.1320799999976 | 830.920090373188 | 1000 | 0.391944269371549 |
| 70.1577599999976 | 831.222130565347 | 1000 | 0.391943051366733 |
| 70.1834399999976 | 831.524167524441 | 1000 | 0.391941832477086 |
| 70.2091199999976 | 831.826201242227 | 1000 | 0.391940612699052 |
| 70.2347999999976 | 832.128231710429 | 1000 | 0.391939392029063 |
| 70.2604799999976 | 832.430258920733 | 1000 | 0.391938170463536 |
| 70.2861599999976 | 832.732282864790 | 1000 | 0.391936947998875 |
| 70.3118399999976 | 833.034303534217 | 1000 | 0.391935724631466 |
| 70.3375199999976 | 833.336320920591 | 1000 | 0.391934500357684 |
| 70.3631999999976 | 833.638335015457 | 1000 | 0.391933275173888 |
| 70.3888799999976 | 833.940345810321 | 1000 | 0.391932049076422 |
| 70.4145599999976 | 834.242353296653 | 1000 | 0.391930822061617 |
| 70.4402399999976 | 834.544357465886 | 1000 | 0.391929594125787 |
| 70.4659199999976 | 834.846358309418 | 1000 | 0.391928365265234 |
| 70.4915999999976 | 835.148355818606 | 1000 | 0.391927135476242 |
| 70.5172799999976 | 835.450349984775 | 1000 | 0.391925904755082 |
| 70.5429599999976 | 835.752340799209 | 1000 | 0.391924673098010 |
| 70.5686399999976 | 836.054328253155 | 1000 | 0.391923440501266 |
| 70.5943199999976 | 836.356312337824 | 1000 | 0.391922206961076 |
| 70.6199999999976 | 836.658293044387 | 1000 | 0.391920972473651 |
| 70.6456799999976 | 836.960270363979 | 1000 | 0.391919737035186 |
| 70.6713599999976 | 837.262244287696 | 1000 | 0.391918500641860 |
| 70.6970399999975 | 837.564214806595 | 1000 | 0.391917263289838 |
| 70.7227199999976 | 837.866181911697 | 1000 | 0.391916024975270 |
| 70.7483999999976 | 838.168145593980 | 1000 | 0.391914785694289 |
| 70.7740799999976 | 838.470105844387 | 1000 | 0.391913545443014 |
| 70.7997599999976 | 838.772062653821 | 1000 | 0.391912304217546 |
| 70.8254399999975 | 839.074016013146 | 1000 | 0.391911062013974 |
| 70.8511199999976 | 839.375965913186 | 1000 | 0.391909818828369 |
| 70.8767999999975 | 839.677912344727 | 1000 | 0.391908574656785 |
| 70.9024799999975 | 839.979855298513 | 1000 | 0.391907329495264 |
| 70.9281599999976 | 840.281794765250 | 1000 | 0.391906083339828 |
| 70.9538399999976 | 840.583730735604 | 1000 | 0.391904836186487 |
| 70.9795199999976 | 840.885663200199 | 1000 | 0.391903588031230 |
| 71.0051999999975 | 841.187592149622 | 1000 | 0.391902338870035 |
| 71.0308799999975 | 841.489517574417 | 1000 | 0.391901088698861 |
| 71.0565599999975 | 841.791439465088 | 1000 | 0.391899837513652 |
| 71.0822399999975 | 842.093357812098 | 1000 | 0.391898585310334 |
| 71.1079199999975 | 842.395272605869 | 1000 | 0.391897332084819 |
| 71.1335999999975 | 842.697183836783 | 1000 | 0.391896077833001 |
| 71.1592799999975 | 842.999091495178 | 1000 | 0.391894822550757 |
| 71.1849599999975 | 843.300995571353 | 1000 | 0.391893566233950 |
| 71.2106399999975 | 843.602896055564 | 1000 | 0.391892308878424 |
| 71.2363199999975 | 843.904792938026 | 1000 | 0.391891050480007 |
| 71.2619999999975 | 844.206686208911 | 1000 | 0.391889791034511 |
| 71.2876799999975 | 844.508575858350 | 1000 | 0.391888530537730 |
| 71.3133599999975 | 844.810461876429 | 1000 | 0.391887268985442 |
| 71.3390399999975 | 845.112344253195 | 1000 | 0.391886006373408 |
| 71.3647199999975 | 845.414222978649 | 1000 | 0.391884742697371 |
| 71.3903999999975 | 845.716098042750 | 1000 | 0.391883477953058 |
| 71.4160799999975 | 846.017969435415 | 1000 | 0.391882212136179 |
| 71.4417599999975 | 846.319837146517 | 1000 | 0.391880945242426 |
| 71.4674399999975 | 846.621701165886 | 1000 | 0.391879677267474 |
| 71.4931199999975 | 846.923561483305 | 1000 | 0.391878408206981 |
| 71.5187999999975 | 847.225418088518 | 1000 | 0.391877138056586 |
| 71.5444799999975 | 847.527270971221 | 1000 | 0.391875866811913 |
| 71.5701599999975 | 847.829120121069 | 1000 | 0.391874594468567 |
| 71.5958399999975 | 848.130965527669 | 1000 | 0.391873321022136 |
| 71.6215199999975 | 848.432807180586 | 1000 | 0.391872046468189 |
| 71.6471999999975 | 848.734645069339 | 1000 | 0.391870770802279 |
| 71.6728799999975 | 849.036479183402 | 1000 | 0.391869494019939 |
| 71.6985599999975 | 849.338309512206 | 1000 | 0.391868216116686 |
| 71.7242399999975 | 849.640136045131 | 1000 | 0.391866937088018 |
| 71.7499199999975 | 849.941958771518 | 1000 | 0.391865656929417 |
| 71.7755999999975 | 850.243777680658 | 1000 | 0.391864375636342 |
| 71.8012799999975 | 850.545592761797 | 1000 | 0.391863093204240 |
| 71.8269599999975 | 850.847404004135 | 1000 | 0.391861809628535 |
| 71.8526399999975 | 851.149211396825 | 1000 | 0.391860524904634 |
| 71.8783199999975 | 851.451014928975 | 1000 | 0.391859239027927 |
| 71.9039999999975 | 851.752814589643 | 1000 | 0.391857951993784 |
| 71.9296799999975 | 852.054610367844 | 1000 | 0.391856663797556 |
| 71.9553599999975 | 852.356402252544 | 1000 | 0.391855374434577 |
| 71.9810399999975 | 852.658190232659 | 1000 | 0.391854083900161 |
| 72.0067199999975 | 852.959974297062 | 1000 | 0.391852792189605 |
| 72.0323999999975 | 853.261754434575 | 1000 | 0.391851499298183 |
| 72.0580799999975 | 853.563530633974 | 1000 | 0.391850205221155 |
| 72.0837599999975 | 853.865302883984 | 1000 | 0.391848909953759 |
| 72.1094399999975 | 854.167071173285 | 1000 | 0.391847613491215 |
| 72.1351199999975 | 854.468835490505 | 1000 | 0.391846315828723 |
| 72.1607999999975 | 854.770595824225 | 1000 | 0.391845016961464 |
| 72.1864799999975 | 855.072352162978 | 1000 | 0.391843716884601 |
| 72.2121599999975 | 855.374104495246 | 1000 | 0.391842415593275 |
| 72.2378399999975 | 855.675852809461 | 1000 | 0.391841113082610 |
| 72.2635199999975 | 855.977597094006 | 1000 | 0.391839809347709 |
| 72.2891999999975 | 856.279337337216 | 1000 | 0.391838504383657 |
| 72.3148799999975 | 856.581073527373 | 1000 | 0.391837198185516 |
| 72.3405599999975 | 856.882805652711 | 1000 | 0.391835890748331 |
| 72.3662399999975 | 857.184533701411 | 1000 | 0.391834582067127 |
| 72.3919199999975 | 857.486257661606 | 1000 | 0.391833272136908 |
| 72.4175999999975 | 857.787977521374 | 1000 | 0.391831960952658 |
| 72.4432799999975 | 858.089693268748 | 1000 | 0.391830648509342 |
| 72.4689599999975 | 858.391404891703 | 1000 | 0.391829334801903 |
| 72.4946399999975 | 858.693112378166 | 1000 | 0.391828019825265 |
| 72.5203199999975 | 858.994815716013 | 1000 | 0.391826703574332 |
| 72.5459999999975 | 859.296514893065 | 1000 | 0.391825386043987 |
| 72.5716799999975 | 859.598209897092 | 1000 | 0.391824067229091 |
| 72.5973599999975 | 859.899900715812 | 1000 | 0.391822747124488 |
| 72.6230399999975 | 860.201587336889 | 1000 | 0.391821425724997 |
| 72.6487199999975 | 860.503269747936 | 1000 | 0.391820103025419 |
| 72.6743999999975 | 860.804947936511 | 1000 | 0.391818779020533 |
| 72.7000799999975 | 861.106621890120 | 1000 | 0.391817453705099 |
| 72.7257599999975 | 861.408291596213 | 1000 | 0.391816127073853 |
| 72.7514399999975 | 861.709957042188 | 1000 | 0.391814799121512 |
| 72.7771199999975 | 862.011618215390 | 1000 | 0.391813469842770 |
| 72.8027999999975 | 862.313275103106 | 1000 | 0.391812139232303 |
| 72.8284799999975 | 862.614927692571 | 1000 | 0.391810807284762 |
| 72.8541599999975 | 862.916575970966 | 1000 | 0.391809473994778 |
| 72.8798399999975 | 863.218219925415 | 1000 | 0.391808139356961 |
| 72.9055199999975 | 863.519859542988 | 1000 | 0.391806803365898 |
| 72.9311999999975 | 863.821494810697 | 1000 | 0.391805466016157 |
| 72.9568799999975 | 864.123125715502 | 1000 | 0.391804127302281 |
| 72.9825599999975 | 864.424752244304 | 1000 | 0.391802787218792 |
| 73.0082399999975 | 864.726374383949 | 1000 | 0.391801445760191 |
| 73.0339199999975 | 865.027992121227 | 1000 | 0.391800102920958 |
| 73.0595999999975 | 865.329605442870 | 1000 | 0.391798758695546 |
| 73.0852799999975 | 865.631214335554 | 1000 | 0.391797413078392 |
| 73.1109599999975 | 865.932818785898 | 1000 | 0.391796066063907 |
| 73.1366399999975 | 866.234418780462 | 1000 | 0.391794717646479 |
| 73.1623199999975 | 866.536014305750 | 1000 | 0.391793367820476 |
| 73.1879999999975 | 866.837605348207 | 1000 | 0.391792016580241 |
| 73.2136799999975 | 867.139191894220 | 1000 | 0.391790663920097 |
| 73.2393599999975 | 867.440773930119 | 1000 | 0.391789309834342 |
| 73.2650399999975 | 867.742351442172 | 1000 | 0.391787954317252 |
| 73.2907199999975 | 868.043924416592 | 1000 | 0.391786597363079 |
| 73.3163999999974 | 868.345492839530 | 1000 | 0.391785238966053 |
| 73.3420799999974 | 868.647056697079 | 1000 | 0.391783879120382 |
| 73.3677599999975 | 868.948615975271 | 1000 | 0.391782517820248 |
| 73.3934399999975 | 869.250170660079 | 1000 | 0.391781155059812 |
| 73.4191199999975 | 869.551720737416 | 1000 | 0.391779790833209 |
| 73.4447999999974 | 869.853266193134 | 1000 | 0.391778425134554 |
| 73.4704799999974 | 870.154807013024 | 1000 | 0.391777057957936 |
| 73.4961599999975 | 870.456343182817 | 1000 | 0.391775689297420 |
| 73.5218399999974 | 870.757874688182 | 1000 | 0.391774319147049 |
| 73.5475199999974 | 871.059401514726 | 1000 | 0.391772947500841 |
| 73.5731999999974 | 871.360923647996 | 1000 | 0.391771574352789 |
| 73.5988799999974 | 871.662441073475 | 1000 | 0.391770199696863 |
| 73.6245599999974 | 871.963953776585 | 1000 | 0.391768823527010 |
| 73.6502399999974 | 872.265461742684 | 1000 | 0.391767445837151 |
| 73.6759199999974 | 872.566964957069 | 1000 | 0.391766066621183 |
| 73.7015999999974 | 872.868463404974 | 1000 | 0.391764685872979 |
| 73.7272799999974 | 873.169957071566 | 1000 | 0.391763303586386 |
| 73.7529599999974 | 873.471445941953 | 1000 | 0.391761919755228 |
| 73.7786399999974 | 873.772930001177 | 1000 | 0.391760534373304 |
| 73.8043199999974 | 874.074409234215 | 1000 | 0.391759147434386 |
| 73.8299999999974 | 874.375883625981 | 1000 | 0.391757758932226 |
| 73.8556799999974 | 874.677353161323 | 1000 | 0.391756368860544 |
| 73.8813599999974 | 874.978817825026 | 1000 | 0.391754977213041 |
| 73.9070399999974 | 875.280277601807 | 1000 | 0.391753583983389 |
| 73.9327199999974 | 875.581732476319 | 1000 | 0.391752189165236 |
| 73.9583999999974 | 875.883182433150 | 1000 | 0.391750792752205 |
| 73.9840799999974 | 876.184627456820 | 1000 | 0.391749394737891 |
| 74.0097599999974 | 876.486067531784 | 1000 | 0.391747995115867 |
| 74.0354399999974 | 876.787502642428 | 1000 | 0.391746593879677 |
| 74.0611199999974 | 877.088932773074 | 1000 | 0.391745191022841 |
| 74.0867999999974 | 877.390357907976 | 1000 | 0.391743786538852 |
| 74.1124799999974 | 877.691778031319 | 1000 | 0.391742380421177 |
| 74.1381599999974 | 877.993193127221 | 1000 | 0.391740972663258 |
| 74.1638399999974 | 878.294603179731 | 1000 | 0.391739563258508 |
| 74.1895199999974 | 878.596008172833 | 1000 | 0.391738152200318 |
| 74.2151999999974 | 878.897408090438 | 1000 | 0.391736739482047 |
| 74.2408799999974 | 879.198802916389 | 1000 | 0.391735325097031 |
| 74.2665599999974 | 879.500192634462 | 1000 | 0.391733909038580 |
| 74.2922399999974 | 879.801577228360 | 1000 | 0.391732491299973 |
| 74.3179199999974 | 880.102956681720 | 1000 | 0.391731071874467 |
| 74.3435999999974 | 880.404330978105 | 1000 | 0.391729650755288 |
| 74.3692799999974 | 880.705700101010 | 1000 | 0.391728227935637 |
| 74.3949599999974 | 881.007064033858 | 1000 | 0.391726803408687 |
| 74.4206399999974 | 881.308422760002 | 1000 | 0.391725377167584 |
| 74.4463199999974 | 881.609776262721 | 1000 | 0.391723949205445 |
| 74.4719999999974 | 881.911124525227 | 1000 | 0.391722519515363 |
| 74.4976799999974 | 882.212467530654 | 1000 | 0.391721088090398 |
| 74.5233599999974 | 882.513805262069 | 1000 | 0.391719654923587 |
| 74.5490399999974 | 882.815137702463 | 1000 | 0.391718220007937 |
| 74.5747199999974 | 883.116464834756 | 1000 | 0.391716783336426 |
| 74.6003999999974 | 883.417786641793 | 1000 | 0.391715344902006 |
| 74.6260799999974 | 883.719103106348 | 1000 | 0.391713904697599 |
| 74.6517599999974 | 884.020414211117 | 1000 | 0.391712462716100 |
| 74.6774399999974 | 884.321719938727 | 1000 | 0.391711018950373 |
| 74.7031199999974 | 884.623020271725 | 1000 | 0.391709573393258 |
| 74.7287999999974 | 884.924315192589 | 1000 | 0.391708126037560 |
| 74.7544799999974 | 885.225604683716 | 1000 | 0.391706676876061 |
| 74.7801599999974 | 885.526888727431 | 1000 | 0.391705225901511 |
| 74.8058399999974 | 885.828167305983 | 1000 | 0.391703773106631 |
| 74.8315199999974 | 886.129440401545 | 1000 | 0.391702318484114 |
| 74.8571999999974 | 886.430707996212 | 1000 | 0.391700862026621 |
| 74.8828799999974 | 886.731970072003 | 1000 | 0.391699403726788 |
| 74.9085599999974 | 887.033226610860 | 1000 | 0.391697943577218 |
| 74.9342399999974 | 887.334477594648 | 1000 | 0.391696481570486 |
| 74.9599199999974 | 887.635723005153 | 1000 | 0.391695017699135 |
| 74.9855999999974 | 887.936962824085 | 1000 | 0.391693551955680 |
| 75.0112799999974 | 888.238197033074 | 1000 | 0.391692084332607 |
| 75.0369599999974 | 888.539425613671 | 1000 | 0.391690614822369 |
| 75.0626399999974 | 888.840648547349 | 1000 | 0.391689143417391 |
| 75.0883199999974 | 889.141865815500 | 1000 | 0.391687670110066 |
| 75.1139999999974 | 889.443077399438 | 1000 | 0.391686194892757 |
| 75.1396799999974 | 889.744283280396 | 1000 | 0.391684717757799 |
| 75.1653599999974 | 890.045483439527 | 1000 | 0.391683238697491 |
| 75.1910399999974 | 890.346677857902 | 1000 | 0.391681757704105 |
| 75.2167199999974 | 890.647866516513 | 1000 | 0.391680274769882 |
| 75.2423999999974 | 890.949049396267 | 1000 | 0.391678789887029 |
| 75.2680799999974 | 891.250226477994 | 1000 | 0.391677303047725 |
| 75.2937599999974 | 891.551397742437 | 1000 | 0.391675814244116 |
| 75.3194399999974 | 891.852563170260 | 1000 | 0.391674323468317 |
| 75.3451199999974 | 892.153722742042 | 1000 | 0.391672830712410 |
| 75.3707999999974 | 892.454876438280 | 1000 | 0.391671335968448 |
| 75.3964799999974 | 892.756024239386 | 1000 | 0.391669839228449 |
| 75.4221599999974 | 893.057166125690 | 1000 | 0.391668340484400 |
| 75.4478399999974 | 893.358302077436 | 1000 | 0.391666839728258 |
| 75.4735199999974 | 893.659432074784 | 1000 | 0.391665336951945 |
| 75.4991999999974 | 893.960556097808 | 1000 | 0.391663832147352 |
| 75.5248799999974 | 894.261674126499 | 1000 | 0.391662325306337 |
| 75.5505599999974 | 894.562786140760 | 1000 | 0.391660816420725 |
| 75.5762399999974 | 894.863892120408 | 1000 | 0.391659305482309 |
| 75.6019199999974 | 895.164992045175 | 1000 | 0.391657792482849 |
| 75.6275999999973 | 895.466085894704 | 1000 | 0.391656277414071 |
| 75.6532799999974 | 895.767173648554 | 1000 | 0.391654760267669 |
| 75.6789599999974 | 896.068255286192 | 1000 | 0.391653241035303 |
| 75.7046399999974 | 896.369330787001 | 1000 | 0.391651719708600 |
| 75.7303199999974 | 896.670400130273 | 1000 | 0.391650196279153 |
| 75.7559999999973 | 896.971463295212 | 1000 | 0.391648670738521 |
| 75.7816799999974 | 897.272520260934 | 1000 | 0.391647143078229 |
| 75.8073599999974 | 897.573571006465 | 1000 | 0.391645613289770 |
| 75.8330399999973 | 897.874615510739 | 1000 | 0.391644081364601 |
| 75.8587199999974 | 898.175653752603 | 1000 | 0.391642547294145 |
| 75.8843999999973 | 898.476685710811 | 1000 | 0.391641011069790 |
| 75.9100799999974 | 898.777711364027 | 1000 | 0.391639472682892 |
| 75.9357599999973 | 899.078730690823 | 1000 | 0.391637932124768 |
| 75.9614399999973 | 899.379743669680 | 1000 | 0.391636389386705 |
| 75.9871199999973 | 899.680750278986 | 1000 | 0.391634844459951 |
| 76.0127999999973 | 899.981750497037 | 1000 | 0.391633297335722 |
| 76.0384799999973 | 900.282744302036 | 1000 | 0.391631748005197 |
| 76.0641599999973 | 900.583731672091 | 1000 | 0.391630196459520 |
| 76.0898399999973 | 900.884712585219 | 1000 | 0.391628642689799 |
| 76.1155199999973 | 901.185687019341 | 1000 | 0.391627086687107 |
| 76.1411999999973 | 901.486654952283 | 1000 | 0.391625528442482 |
| 76.1668799999973 | 901.787616361778 | 1000 | 0.391623967946924 |
| 76.1925599999973 | 902.088571225461 | 1000 | 0.391622405191399 |
| 76.2182399999973 | 902.389519520872 | 1000 | 0.391620840166835 |
| 76.2439199999973 | 902.690461225458 | 1000 | 0.391619272864124 |
| 76.2695999999973 | 902.991396316564 | 1000 | 0.391617703274122 |
| 76.2952799999973 | 903.292324771443 | 1000 | 0.391616131387649 |
| 76.3209599999973 | 903.593246567245 | 1000 | 0.391614557195487 |
| 76.3466399999973 | 903.894161681028 | 1000 | 0.391612980688381 |
| 76.3723199999973 | 904.195070089749 | 1000 | 0.391611401857039 |
| 76.3979999999973 | 904.495971770264 | 1000 | 0.391609820692133 |
| 76.4236799999973 | 904.796866699335 | 1000 | 0.391608237184295 |
| 76.4493599999973 | 905.097754853619 | 1000 | 0.391606651324123 |
| 76.4750399999973 | 905.398636209678 | 1000 | 0.391605063102173 |
| 76.5007199999973 | 905.699510743970 | 1000 | 0.391603472508967 |
| 76.5263999999973 | 906.000378432853 | 1000 | 0.391601879534987 |
| 76.5520799999973 | 906.301239252584 | 1000 | 0.391600284170677 |
| 76.5777599999973 | 906.602093179320 | 1000 | 0.391598686406443 |
| 76.6034399999973 | 906.902940189114 | 1000 | 0.391597086232653 |
| 76.6291199999973 | 907.203780257915 | 1000 | 0.391595483639634 |
| 76.6547999999973 | 907.504613361572 | 1000 | 0.391593878617678 |
| 76.6804799999973 | 907.805439475830 | 1000 | 0.391592271157034 |
| 76.7061599999973 | 908.106258576328 | 1000 | 0.391590661247916 |
| 76.7318399999973 | 908.407070638603 | 1000 | 0.391589048880495 |
| 76.7575199999973 | 908.707875638086 | 1000 | 0.391587434044905 |
| 76.7831999999973 | 909.008673550104 | 1000 | 0.391585816731239 |
| 76.8088799999973 | 909.309464349876 | 1000 | 0.391584196929552 |
| 76.8345599999973 | 909.610248012516 | 1000 | 0.391582574629857 |
| 76.8602399999973 | 909.911024513034 | 1000 | 0.391580949822129 |
| 76.8859199999973 | 910.211793826329 | 1000 | 0.391579322496301 |
| 76.9115999999973 | 910.512555927195 | 1000 | 0.391577692642267 |
| 76.9372799999973 | 910.813310790317 | 1000 | 0.391576060249879 |
| 76.9629599999973 | 911.114058390272 | 1000 | 0.391574425308950 |
| 76.9886399999973 | 911.414798701528 | 1000 | 0.391572787809251 |
| 77.0143199999973 | 911.715531698443 | 1000 | 0.391571147740512 |
| 77.0399999999973 | 912.016257355268 | 1000 | 0.391569505092423 |
| 77.0656799999973 | 912.316975646139 | 1000 | 0.391567859854630 |
| 77.0913599999973 | 912.617686545087 | 1000 | 0.391566212016741 |
| 77.1170399999973 | 912.918390026026 | 1000 | 0.391564561568320 |
| 77.1427199999973 | 913.219086062762 | 1000 | 0.391562908498889 |
| 77.1683999999973 | 913.519774628988 | 1000 | 0.391561252797929 |
| 77.1940799999973 | 913.820455698285 | 1000 | 0.391559594454879 |
| 77.2197599999973 | 914.121129244119 | 1000 | 0.391557933459135 |
| 77.2454399999973 | 914.421795239845 | 1000 | 0.391556269800050 |
| 77.2711199999973 | 914.722453658700 | 1000 | 0.391554603466935 |
| 77.2967999999973 | 915.023104473812 | 1000 | 0.391552934449058 |
| 77.3224799999973 | 915.323747658189 | 1000 | 0.391551262735645 |
| 77.3481599999973 | 915.624383184726 | 1000 | 0.391549588315876 |
| 77.3738399999973 | 915.925011026201 | 1000 | 0.391547911178891 |
| 77.3995199999973 | 916.225631155276 | 1000 | 0.391546231313784 |
| 77.4251999999973 | 916.526243544495 | 1000 | 0.391544548709606 |
| 77.4508799999973 | 916.826848166287 | 1000 | 0.391542863355365 |
| 77.4765599999973 | 917.127444992959 | 1000 | 0.391541175240023 |
| 77.5022399999973 | 917.428033996704 | 1000 | 0.391539484352499 |
| 77.5279199999973 | 917.728615149591 | 1000 | 0.391537790681668 |
| 77.5535999999973 | 918.029188423574 | 1000 | 0.391536094216359 |
| 77.5792799999973 | 918.329753790485 | 1000 | 0.391534394945358 |
| 77.6049599999973 | 918.630311222035 | 1000 | 0.391532692857404 |
| 77.6306399999973 | 918.930860689814 | 1000 | 0.391530987941192 |
| 77.6563199999973 | 919.231402165291 | 1000 | 0.391529280185371 |
| 77.6819999999973 | 919.531935619813 | 1000 | 0.391527569578547 |
| 77.7076799999973 | 919.832461024605 | 1000 | 0.391525856109276 |
| 77.7333599999973 | 920.132978350765 | 1000 | 0.391524139766071 |
| 77.7590399999973 | 920.433487569273 | 1000 | 0.391522420537398 |
| 77.7847199999973 | 920.733988650981 | 1000 | 0.391520698411679 |
| 77.8103999999973 | 921.034481566618 | 1000 | 0.391518973377285 |
| 77.8360799999973 | 921.334966286786 | 1000 | 0.391517245422545 |
| 77.8617599999973 | 921.635442781962 | 1000 | 0.391515514535738 |
| 77.8874399999973 | 921.935911022497 | 1000 | 0.391513780705097 |
| 77.9131199999973 | 922.236370978616 | 1000 | 0.391512043918809 |
| 77.9387999999973 | 922.536822620415 | 1000 | 0.391510304165011 |
| 77.9644799999973 | 922.837265917863 | 1000 | 0.391508561431796 |
| 77.9901599999973 | 923.137700840799 | 1000 | 0.391506815707207 |
| 78.0158399999973 | 923.438127358935 | 1000 | 0.391505066979238 |
| 78.0415199999973 | 923.738545441852 | 1000 | 0.391503315235837 |
| 78.0671999999973 | 924.038955059001 | 1000 | 0.391501560464903 |
| 78.0928799999973 | 924.339356179703 | 1000 | 0.391499802654285 |
| 78.1185599999973 | 924.639748773148 | 1000 | 0.391498041791787 |
| 78.1442399999973 | 924.940132808392 | 1000 | 0.391496277865160 |
| 78.1699199999973 | 925.240508254361 | 1000 | 0.391494510862108 |
| 78.1955999999973 | 925.540875079847 | 1000 | 0.391492740770285 |
| 78.2212799999973 | 925.841233253508 | 1000 | 0.391490967577296 |
| 78.2469599999972 | 926.141582743869 | 1000 | 0.391489191270696 |
| 78.2726399999972 | 926.441923519320 | 1000 | 0.391487411837990 |
| 78.2983199999972 | 926.742255548116 | 1000 | 0.391485629266634 |
| 78.3239999999973 | 927.042578798375 | 1000 | 0.391483843544031 |
| 78.3496799999973 | 927.342893238080 | 1000 | 0.391482054657537 |
| 78.3753599999972 | 927.643198835075 | 1000 | 0.391480262594454 |
| 78.4010399999972 | 927.943495557070 | 1000 | 0.391478467342035 |
| 78.4267199999973 | 928.243783371634 | 1000 | 0.391476668887483 |
| 78.4523999999972 | 928.544062246197 | 1000 | 0.391474867217946 |
| 78.4780799999972 | 928.844332148052 | 1000 | 0.391473062320524 |
| 78.5037599999972 | 929.144593044350 | 1000 | 0.391471254182263 |
| 78.5294399999972 | 929.444844902102 | 1000 | 0.391469442790158 |
| 78.5551199999973 | 929.745087688180 | 1000 | 0.391467628131153 |
| 78.5807999999972 | 930.045321369310 | 1000 | 0.391465810192138 |
| 78.6064799999972 | 930.345545912080 | 1000 | 0.391463988959950 |
| 78.6321599999972 | 930.645761282932 | 1000 | 0.391462164421375 |
| 78.6578399999972 | 930.945967448167 | 1000 | 0.391460336563145 |
| 78.6835199999972 | 931.246164373939 | 1000 | 0.391458505371938 |
| 78.7091999999972 | 931.546352026260 | 1000 | 0.391456670834380 |
| 78.7348799999972 | 931.846530370995 | 1000 | 0.391454832937044 |
| 78.7605599999972 | 932.146699373863 | 1000 | 0.391452991666447 |
| 78.7862399999972 | 932.446859000437 | 1000 | 0.391451147009053 |
| 78.8119199999972 | 932.747009216144 | 1000 | 0.391449298951271 |
| 78.8375999999972 | 933.047149986259 | 1000 | 0.391447447479458 |
| 78.8632799999972 | 933.347281275914 | 1000 | 0.391445592579913 |
| 78.8889599999972 | 933.647403050087 | 1000 | 0.391443734238882 |
| 78.9146399999972 | 933.947515273610 | 1000 | 0.391441872442555 |
| 78.9403199999972 | 934.247617911162 | 1000 | 0.391440007177068 |
| 78.9659999999972 | 934.547710927272 | 1000 | 0.391438138428500 |
| 78.9916799999972 | 934.847794286317 | 1000 | 0.391436266182874 |
| 79.0173599999972 | 935.147867952523 | 1000 | 0.391434390426158 |
| 79.0430399999972 | 935.447931889960 | 1000 | 0.391432511144263 |
| 79.0687199999972 | 935.747986062548 | 1000 | 0.391430628323045 |
| 79.0943999999972 | 936.048030434050 | 1000 | 0.391428741948301 |
| 79.1200799999972 | 936.348064968076 | 1000 | 0.391426852005773 |
| 79.1457599999972 | 936.648089628079 | 1000 | 0.391424958481143 |
| 79.1714399999972 | 936.948104377356 | 1000 | 0.391423061360040 |
| 79.1971199999972 | 937.248109179046 | 1000 | 0.391421160628032 |
| 79.2227999999972 | 937.548103996135 | 1000 | 0.391419256270630 |
| 79.2484799999972 | 937.848088791444 | 1000 | 0.391417348273287 |
| 79.2741599999972 | 938.148063527641 | 1000 | 0.391415436621398 |
| 79.2998399999972 | 938.448028167230 | 1000 | 0.391413521300299 |
| 79.3255199999972 | 938.747982672557 | 1000 | 0.391411602295267 |
| 79.3511999999972 | 939.047927005807 | 1000 | 0.391409679591520 |
| 79.3768799999972 | 939.347861129003 | 1000 | 0.391407753174218 |
| 79.4025599999972 | 939.647785004005 | 1000 | 0.391405823028460 |
| 79.4282399999972 | 939.947698592511 | 1000 | 0.391403889139286 |
| 79.4539199999972 | 940.247601856054 | 1000 | 0.391401951491676 |
| 79.4795999999972 | 940.547494756005 | 1000 | 0.391400010070549 |
| 79.5052799999972 | 940.847377253566 | 1000 | 0.391398064860764 |
| 79.5309599999972 | 941.147249309776 | 1000 | 0.391396115847120 |
| 79.5566399999972 | 941.447110885508 | 1000 | 0.391394163014354 |
| 79.5823199999972 | 941.746961941465 | 1000 | 0.391392206347142 |
| 79.6079999999972 | 942.046802438184 | 1000 | 0.391390245830100 |
| 79.6336799999972 | 942.346632336033 | 1000 | 0.391388281447781 |
| 79.6593599999972 | 942.646451595211 | 1000 | 0.391386313184675 |
| 79.6850399999972 | 942.946260175745 | 1000 | 0.391384341025212 |
| 79.7107199999972 | 943.246058037494 | 1000 | 0.391382364953759 |
| 79.7363999999972 | 943.545845140143 | 1000 | 0.391380384954618 |
| 79.7620799999972 | 943.845621443207 | 1000 | 0.391378401012033 |
| 79.7877599999972 | 944.145386906024 | 1000 | 0.391376413110179 |
| 79.8134399999972 | 944.445141487763 | 1000 | 0.391374421233172 |
| 79.8391199999972 | 944.744885147415 | 1000 | 0.391372425365062 |
| 79.8647999999972 | 945.044617843798 | 1000 | 0.391370425489837 |
| 79.8904799999972 | 945.344339535553 | 1000 | 0.391368421591418 |
| 79.9161599999972 | 945.644050181143 | 1000 | 0.391366413653664 |
| 79.9418399999972 | 945.943749738856 | 1000 | 0.391364401660368 |
| 79.9675199999972 | 946.243438166800 | 1000 | 0.391362385595259 |
| 79.9931999999972 | 946.543115422905 | 1000 | 0.391360365441999 |
| 80.0188799999972 | 946.842781464921 | 1000 | 0.391358341184188 |
| 80.0445599999972 | 947.142436250415 | 1000 | 0.391356312805356 |
| 80.0702399999972 | 947.442079736777 | 1000 | 0.391354280288969 |
| 80.0959199999972 | 947.741711881213 | 1000 | 0.391352243618427 |
| 80.1215999999972 | 948.041332640744 | 1000 | 0.391350202777064 |
| 80.1472799999972 | 948.340941972210 | 1000 | 0.391348157748145 |
| 80.1729599999972 | 948.640539832267 | 1000 | 0.391346108514870 |
| 80.1986399999972 | 948.940126177383 | 1000 | 0.391344055060371 |
| 80.2243199999972 | 949.239700963842 | 1000 | 0.391341997367711 |
| 80.2499999999972 | 949.539264147742 | 1000 | 0.391339935419888 |
| 80.2756799999972 | 949.838815684991 | 1000 | 0.391337869199828 |
| 80.3013599999972 | 950.138355531310 | 1000 | 0.391335798690393 |
| 80.3270399999972 | 950.437883642232 | 1000 | 0.391333723874373 |
| 80.3527199999972 | 950.737399973096 | 1000 | 0.391331644734491 |
| 80.3783999999972 | 951.036904479056 | 1000 | 0.391329561253398 |
| 80.4040799999972 | 951.336397115069 | 1000 | 0.391327473413678 |
| 80.4297599999972 | 951.635877835904 | 1000 | 0.391325381197845 |
| 80.4554399999972 | 951.935346596132 | 1000 | 0.391323284588342 |
| 80.4811199999973 | 952.234803350135 | 1000 | 0.391321183567542 |
| 80.5067999999972 | 952.534248052096 | 1000 | 0.391319078117747 |
| 80.5324799999973 | 952.833680656004 | 1000 | 0.391316968221189 |
| 80.5581599999973 | 953.133101115653 | 1000 | 0.391314853860027 |
| 80.5838399999973 | 953.432509384637 | 1000 | 0.391312735016351 |
| 80.6095199999973 | 953.731905416352 | 1000 | 0.391310611672176 |
| 80.6351999999973 | 954.031289163997 | 1000 | 0.391308483809449 |
| 80.6608799999973 | 954.330660580569 | 1000 | 0.391306351410041 |
| 80.6865599999973 | 954.630019618866 | 1000 | 0.391304214455751 |
| 80.7122399999973 | 954.929366231482 | 1000 | 0.391302072928308 |
| 80.7379199999973 | 955.228700370811 | 1000 | 0.391299926809363 |
| 80.7635999999973 | 955.528021989043 | 1000 | 0.391297776080497 |
| 80.7892799999973 | 955.827331038161 | 1000 | 0.391295620723217 |
| 80.8149599999973 | 956.126627469948 | 1000 | 0.391293460718954 |
| 80.8406399999974 | 956.425911235977 | 1000 | 0.391291296049065 |
| 80.8663199999974 | 956.725182287616 | 1000 | 0.391289126694834 |
| 80.8919999999974 | 957.024440576025 | 1000 | 0.391286952637469 |
| 80.9176799999974 | 957.323686052154 | 1000 | 0.391284773858101 |
| 80.9433599999974 | 957.622918666746 | 1000 | 0.391282590337788 |
| 80.9690399999974 | 957.922138370333 | 1000 | 0.391280402057511 |
| 80.9947199999974 | 958.221345113233 | 1000 | 0.391278208998174 |
| 81.0203999999974 | 958.520538845555 | 1000 | 0.391276011140605 |
| 81.0460799999974 | 958.819719517194 | 1000 | 0.391273808465557 |
| 81.0717599999974 | 959.118887077831 | 1000 | 0.391271600953702 |
| 81.0974399999974 | 959.418041476932 | 1000 | 0.391269388585638 |
| 81.1231199999974 | 959.717182663747 | 1000 | 0.391267171341884 |
| 81.1487999999974 | 960.016310587310 | 1000 | 0.391264949202881 |
| 81.1744799999974 | 960.315425196437 | 1000 | 0.391262722148990 |
| 81.2001599999975 | 960.614526439725 | 1000 | 0.391260490160496 |
| 81.2258399999975 | 960.913614265551 | 1000 | 0.391258253217604 |
| 81.2515199999975 | 961.212688622074 | 1000 | 0.391256011300439 |
| 81.2771999999975 | 961.511749457230 | 1000 | 0.391253764389046 |
| 81.3028799999975 | 961.810796718731 | 1000 | 0.391251512463392 |
| 81.3285599999975 | 962.109830354069 | 1000 | 0.391249255503362 |
| 81.3542399999975 | 962.408850310510 | 1000 | 0.391246993488760 |
| 81.3799199999975 | 962.707856535096 | 1000 | 0.391244726399312 |
| 81.4055999999975 | 963.006848974640 | 1000 | 0.391242454214658 |
| 81.4312799999975 | 963.305827575732 | 1000 | 0.391240176914361 |
| 81.4569599999975 | 963.604792284732 | 1000 | 0.391237894477900 |
| 81.4826399999976 | 963.903743047769 | 1000 | 0.391235606884671 |
| 81.5083199999976 | 964.202679810746 | 1000 | 0.391233314113989 |
| 81.5339999999976 | 964.501602519332 | 1000 | 0.391231016145085 |
| 81.5596799999976 | 964.800511118965 | 1000 | 0.391228712957108 |
| 81.5853599999976 | 965.099405554851 | 1000 | 0.391226404529122 |
| 81.6110399999976 | 965.398285771961 | 1000 | 0.391224090840107 |
| 81.6367199999976 | 965.697151715031 | 1000 | 0.391221771868961 |
| 81.6623999999976 | 965.996003328561 | 1000 | 0.391219447594495 |
| 81.6880799999976 | 966.294840556816 | 1000 | 0.391217117995437 |
| 81.7137599999976 | 966.593663343821 | 1000 | 0.391214783050427 |
| 81.7394399999976 | 966.892471633361 | 1000 | 0.391212442738023 |
| 81.7651199999976 | 967.191265368985 | 1000 | 0.391210097036695 |
| 81.7907999999977 | 967.490044493999 | 1000 | 0.391207745924825 |
| 81.8164799999977 | 967.788808951464 | 1000 | 0.391205389380713 |
| 81.8421599999977 | 968.087558684203 | 1000 | 0.391203027382567 |
| 81.8678399999977 | 968.386293634792 | 1000 | 0.391200659908512 |
| 81.8935199999977 | 968.685013745563 | 1000 | 0.391198286936582 |
| 81.9191999999977 | 968.983718958602 | 1000 | 0.391195908444725 |
| 81.9448799999977 | 969.282409215745 | 1000 | 0.391193524410800 |
| 81.9705599999977 | 969.581084458585 | 1000 | 0.391191134812577 |
| 81.9962399999977 | 969.879744628461 | 1000 | 0.391188739627738 |
| 82.0219199999977 | 970.178389666463 | 1000 | 0.391186338833873 |
| 82.0475999999977 | 970.477019513432 | 1000 | 0.391183932408485 |
| 82.0732799999977 | 970.775634109953 | 1000 | 0.391181520328986 |
| 82.0989599999977 | 971.074233396359 | 1000 | 0.391179102572697 |
| 82.1246399999977 | 971.372817312729 | 1000 | 0.391176679116848 |
| 82.1503199999978 | 971.671385798886 | 1000 | 0.391174249938579 |
| 82.1759999999978 | 971.969938794395 | 1000 | 0.391171815014937 |
| 82.2016799999978 | 972.268476238564 | 1000 | 0.391169374322878 |
| 82.2273599999978 | 972.566998070443 | 1000 | 0.391166927839265 |
| 82.2530399999978 | 972.865504228820 | 1000 | 0.391164475540868 |
| 82.2787199999978 | 973.163994652223 | 1000 | 0.391162017404365 |
| 82.3043999999978 | 973.462469278917 | 1000 | 0.391159553406340 |
| 82.3300799999978 | 973.760928046904 | 1000 | 0.391157083523283 |
| 82.3557599999978 | 974.059370893922 | 1000 | 0.391154607731591 |
| 82.3814399999978 | 974.357797757443 | 1000 | 0.391152126007563 |
| 82.4071199999978 | 974.656208574671 | 1000 | 0.391149638327408 |
| 82.4327999999978 | 974.954603282544 | 1000 | 0.391147144667236 |
| 82.4584799999978 | 975.252981817729 | 1000 | 0.391144645003062 |
| 82.4841599999978 | 975.551344116625 | 1000 | 0.391142139310806 |
| 82.5098399999979 | 975.849690115358 | 1000 | 0.391139627566290 |
| 82.5355199999979 | 976.148019749781 | 1000 | 0.391137109745241 |
| 82.5611999999979 | 976.446332955476 | 1000 | 0.391134585823287 |
| 82.5868799999979 | 976.744629667747 | 1000 | 0.391132055775959 |
| 82.6125599999979 | 977.042909821624 | 1000 | 0.391129519578691 |
| 82.6382399999979 | 977.341173351860 | 1000 | 0.391126977206816 |
| 82.6639199999979 | 977.639420192927 | 1000 | 0.391124428635570 |
| 82.6895999999979 | 977.937650279022 | 1000 | 0.391121873840091 |
| 82.7152799999979 | 978.235863544056 | 1000 | 0.391119312795414 |
| 82.7409599999979 | 978.534059921663 | 1000 | 0.391116745476477 |
| 82.7666399999979 | 978.832239345190 | 1000 | 0.391114171858117 |
| 82.7923199999979 | 979.130401747703 | 1000 | 0.391111591915068 |
| 82.8179999999980 | 979.428547061979 | 1000 | 0.391109005621966 |
| 82.8436799999980 | 979.726675220512 | 1000 | 0.391106412953342 |
| 82.8693599999980 | 980.024786155504 | 1000 | 0.391103813883629 |
| 82.8950399999980 | 980.322879798872 | 1000 | 0.391101208387154 |
| 82.9207199999980 | 980.620956082239 | 1000 | 0.391098596438143 |
| 82.9463999999980 | 980.919014936940 | 1000 | 0.391095978010719 |
| 82.9720799999980 | 981.217056294012 | 1000 | 0.391093353078898 |
| 82.9977599999980 | 981.515080084204 | 1000 | 0.391090721616597 |
| 83.0234399999980 | 981.813086237965 | 1000 | 0.391088083597626 |
| 83.0491199999980 | 982.111074685448 | 1000 | 0.391085438995688 |
| 83.0747999999980 | 982.409045356512 | 1000 | 0.391082787784384 |
| 83.1004799999980 | 982.706998180711 | 1000 | 0.391080129937209 |
| 83.1261599999981 | 983.004933087302 | 1000 | 0.391077465427549 |
| 83.1518399999981 | 983.302850005242 | 1000 | 0.391074794228686 |
| 83.1775199999981 | 983.600748863180 | 1000 | 0.391072116313793 |
| 83.2031999999981 | 983.898629589465 | 1000 | 0.391069431655939 |
| 83.2288799999981 | 984.196492112139 | 1000 | 0.391066740228081 |
| 83.2545599999981 | 984.494336358936 | 1000 | 0.391064042003071 |
| 83.2802399999981 | 984.792162257285 | 1000 | 0.391061336953649 |
| 83.3059199999981 | 985.089969734301 | 1000 | 0.391058625052448 |
| 83.3315999999981 | 985.387758716793 | 1000 | 0.391055906271991 |
| 83.3572799999981 | 985.685529131255 | 1000 | 0.391053180584692 |
| 83.3829599999981 | 985.983280903867 | 1000 | 0.391050447962851 |
| 83.4086399999981 | 986.281013960496 | 1000 | 0.391047708378662 |
| 83.4343199999981 | 986.578728226693 | 1000 | 0.391044961804202 |
| 83.4599999999982 | 986.876423627691 | 1000 | 0.391042208211444 |
| 83.4856799999982 | 987.174100088403 | 1000 | 0.391039447572237 |
| 83.5113599999982 | 987.471757533423 | 1000 | 0.391036679858329 |
| 83.5370399999982 | 987.769395887025 | 1000 | 0.391033905041349 |
| 83.5627199999982 | 988.067015073156 | 1000 | 0.391031123092813 |
| 83.5883999999982 | 988.364615015444 | 1000 | 0.391028333984123 |
| 83.6140799999982 | 988.662195637188 | 1000 | 0.391025537686567 |
| 83.6397599999982 | 988.959756861360 | 1000 | 0.391022734171318 |
| 83.6654399999982 | 989.257298610604 | 1000 | 0.391019923409433 |
| 83.6911199999982 | 989.554820807236 | 1000 | 0.391017105371853 |
| 83.7167999999982 | 989.852323373238 | 1000 | 0.391014280029403 |
| 83.7424799999982 | 990.149806230262 | 1000 | 0.391011447352791 |
| 83.7681599999983 | 990.447269299624 | 1000 | 0.391008607312608 |
| 83.7938399999982 | 990.744712502305 | 1000 | 0.391005759879327 |
| 83.8195199999983 | 991.042135758951 | 1000 | 0.391002905023302 |
| 83.8451999999983 | 991.339538989868 | 1000 | 0.391000042714770 |
| 83.8708799999983 | 991.636922115021 | 1000 | 0.390997172923846 |
| 83.8965599999983 | 991.934285054036 | 1000 | 0.390994295620529 |
| 83.9222399999983 | 992.231627726197 | 1000 | 0.390991410774695 |
| 83.9479199999983 | 992.528950050441 | 1000 | 0.390988518356100 |
| 83.9735999999983 | 992.826251945363 | 1000 | 0.390985618334380 |
| 83.9992799999983 | 993.123533329208 | 1000 | 0.390982710679048 |
| 84.0249599999983 | 993.420794119874 | 1000 | 0.390979795359495 |
| 84.0506399999983 | 993.718034234910 | 1000 | 0.390976872344991 |
| 84.0763199999984 | 994.015253591511 | 1000 | 0.390973941604680 |
| 84.1019999999983 | 994.312452106521 | 1000 | 0.390971003107586 |
| 84.1276799999984 | 994.609629696428 | 1000 | 0.390968056822606 |
| 84.1533599999984 | 994.906786277368 | 1000 | 0.390965102718514 |
| 84.1790399999984 | 995.203921765114 | 1000 | 0.390962140763959 |
| 84.2047199999984 | 995.501036075084 | 1000 | 0.390959170927463 |
| 84.2303999999984 | 995.798129122335 | 1000 | 0.390956193177424 |
| 84.2560799999984 | 996.095200821560 | 1000 | 0.390953207482112 |
| 84.2817599999984 | 996.392251087091 | 1000 | 0.390950213809670 |
| 84.3074399999984 | 996.689279832893 | 1000 | 0.390947212128114 |
| 84.3331199999984 | 996.986286972565 | 1000 | 0.390944202405332 |
| 84.3587999999984 | 997.283272419338 | 1000 | 0.390941184609083 |
| 84.3844799999984 | 997.580236086074 | 1000 | 0.390938158706996 |
| 84.4101599999984 | 997.877177885262 | 1000 | 0.390935124666572 |
| 84.4358399999985 | 998.174097729020 | 1000 | 0.390932082455182 |
| 84.4615199999985 | 998.470995529090 | 1000 | 0.390929032040065 |
| 84.4871999999985 | 998.767871196837 | 1000 | 0.390925973388330 |
| 84.5128799999985 | 999.064724643252 | 1000 | 0.390922906466952 |
| 84.5385599999985 | 999.361555778944 | 1000 | 0.390919831242777 |
| 84.5642399999985 | 999.658364514140 | 1000 | 0.390916747682516 |
| 84.5899199999985 | 999.955150758689 | 1000 | 0.390913655752748 |
| 84.6155999999985 | 1000.25191442205 | 1000 | 0.390910555419917 |
| 84.6412799999985 | 1000.54865541330 | 1000 | 0.390907446650334 |
| 84.6669599999985 | 1000.84537364113 | 1000 | 0.390904329410174 |
| 84.6926399999985 | 1001.14206901384 | 1000 | 0.390901203665476 |
| 84.7183199999985 | 1001.43874143934 | 1000 | 0.390898069382146 |
| 84.7439999999985 | 1001.73539082514 | 1000 | 0.390894926525949 |
| 84.7696799999985 | 1002.03201707837 | 1000 | 0.390891775062517 |
| 84.7953599999986 | 1002.32862010575 | 1000 | 0.390888614957343 |
| 84.8210399999986 | 1002.62519981362 | 1000 | 0.390885446175780 |
| 84.8467199999986 | 1002.92175610791 | 1000 | 0.390882268683046 |
| 84.8723999999986 | 1003.21828889414 | 1000 | 0.390879082444216 |
| 84.8980799999986 | 1003.51479807744 | 1000 | 0.390875887424228 |
| 84.9237599999986 | 1003.81128356254 | 1000 | 0.390872683587877 |
| 84.9494399999986 | 1004.10774525376 | 1000 | 0.390869470899820 |
| 84.9751199999986 | 1004.40418305500 | 1000 | 0.390866249324571 |
| 85.0007999999986 | 1004.70059686976 | 1000 | 0.390863018826500 |
| 85.0264799999986 | 1004.99698660114 | 1000 | 0.390859779369839 |
| 85.0521599999986 | 1005.29335215182 | 1000 | 0.390856530918672 |
| 85.0778399999986 | 1005.58969342405 | 1000 | 0.390853273436943 |
| 85.1035199999987 | 1005.88601031968 | 1000 | 0.390850006888449 |
| 85.1291999999987 | 1006.18230274014 | 1000 | 0.390846731236843 |
| 85.1548799999987 | 1006.47857058644 | 1000 | 0.390843446445633 |
| 85.1805599999987 | 1006.77481375916 | 1000 | 0.390840152478180 |
| 85.2062399999987 | 1007.07103215847 | 1000 | 0.390836849297700 |
| 85.2319199999987 | 1007.36722568411 | 1000 | 0.390833536867259 |
| 85.2575999999987 | 1007.66339423538 | 1000 | 0.390830215149778 |
| 85.2832799999987 | 1007.95953771117 | 1000 | 0.390826884108028 |
| 85.3089599999987 | 1008.25565600994 | 1000 | 0.390823543704631 |
| 85.3346399999987 | 1008.55174902969 | 1000 | 0.390820193902060 |
| 85.3603199999987 | 1008.84781666802 | 1000 | 0.390816834662637 |
| 85.3859999999988 | 1009.14385882207 | 1000 | 0.390813465948534 |
| 85.4116799999988 | 1009.43987538856 | 1000 | 0.390810087721771 |
| 85.4373599999988 | 1009.73586626375 | 1000 | 0.390806699944217 |
| 85.4630399999988 | 1010.03183134348 | 1000 | 0.390803302577586 |
| 85.4887199999988 | 1010.32777052312 | 1000 | 0.390799895583442 |
| 85.5143999999988 | 1010.62368369763 | 1000 | 0.390796478923192 |
| 85.5400799999988 | 1010.91957076149 | 1000 | 0.390793052558091 |
| 85.5657599999988 | 1011.21543160875 | 1000 | 0.390789616449238 |
| 85.5914399999988 | 1011.51126613300 | 1000 | 0.390786170557576 |
| 85.6171199999988 | 1011.80707422738 | 1000 | 0.390782714843892 |
| 85.6427999999988 | 1012.10285578457 | 1000 | 0.390779249268816 |
| 85.6684799999988 | 1012.39861069680 | 1000 | 0.390775773792820 |
| 85.6941599999988 | 1012.69433885584 | 1000 | 0.390772288376218 |
| 85.7198399999988 | 1012.99004015300 | 1000 | 0.390768792979167 |
| 85.7455199999989 | 1013.28571447912 | 1000 | 0.390765287561662 |
| 85.7711999999989 | 1013.58136172458 | 1000 | 0.390761772083538 |
| 85.7968799999989 | 1013.87698177930 | 1000 | 0.390758246504471 |
| 85.8225599999989 | 1014.17257453272 | 1000 | 0.390754710783975 |
| 85.8482399999989 | 1014.46813987382 | 1000 | 0.390751164881401 |
| 85.8739199999989 | 1014.76367769109 | 1000 | 0.390747608755938 |
| 85.8995999999989 | 1015.05918787257 | 1000 | 0.390744042366612 |
| 85.9252799999989 | 1015.35467030580 | 1000 | 0.390740465672285 |
| 85.9509599999989 | 1015.65012487786 | 1000 | 0.390736878631653 |
| 85.9766399999989 | 1015.94555147534 | 1000 | 0.390733281203250 |
| 86.0023199999989 | 1016.24094998434 | 1000 | 0.390729673345439 |
| 86.0279999999989 | 1016.53632029049 | 1000 | 0.390726055016422 |
| 86.0536799999989 | 1016.83166227892 | 1000 | 0.390722426174229 |
| 86.0793599999989 | 1017.12697583429 | 1000 | 0.390718786776725 |
| 86.1050399999990 | 1017.42226084075 | 1000 | 0.390715136781605 |
| 86.1307199999990 | 1017.71751718196 | 1000 | 0.390711476146396 |
| 86.1563999999990 | 1018.01274474109 | 1000 | 0.390707804828454 |
| 86.1820799999990 | 1018.30794340081 | 1000 | 0.390704122784964 |
| 86.2077599999990 | 1018.60311304330 | 1000 | 0.390700429972941 |
| 86.2334399999990 | 1018.89825355022 | 1000 | 0.390696726349226 |
| 86.2591199999990 | 1019.19336480275 | 1000 | 0.390693011870491 |
| 86.2847999999990 | 1019.48844668153 | 1000 | 0.390689286493229 |
| 86.3104799999990 | 1019.78349906673 | 1000 | 0.390685550173766 |
| 86.3361599999990 | 1020.07852183799 | 1000 | 0.390681802868246 |
| 86.3618399999990 | 1020.37351487443 | 1000 | 0.390678044532643 |
| 86.3875199999990 | 1020.66847805468 | 1000 | 0.390674275122752 |
| 86.4131999999991 | 1020.96341125684 | 1000 | 0.390670494594192 |
| 86.4388799999991 | 1021.25831435847 | 1000 | 0.390666702902405 |
| 86.4645599999991 | 1021.55318723666 | 1000 | 0.390662900002654 |
| 86.4902399999991 | 1021.84802976793 | 1000 | 0.390659085850023 |
| 86.5159199999991 | 1022.14284182829 | 1000 | 0.390655260399416 |
| 86.5415999999991 | 1022.43762329322 | 1000 | 0.390651423605557 |
| 86.5672799999991 | 1022.73237403769 | 1000 | 0.390647575422990 |
| 86.5929599999991 | 1023.02709393612 | 1000 | 0.390643715806076 |
| 86.6186399999991 | 1023.32178286238 | 1000 | 0.390639844708992 |
| 86.6443199999991 | 1023.61644068984 | 1000 | 0.390635962085735 |
| 86.6699999999991 | 1023.91106729130 | 1000 | 0.390632067890115 |
| 86.6956799999991 | 1024.20566253903 | 1000 | 0.390628162075758 |
| 86.7213599999992 | 1024.50022630477 | 1000 | 0.390624244596107 |
| 86.7470399999992 | 1024.79475845969 | 1000 | 0.390620315404414 |
| 86.7727199999992 | 1025.08925887443 | 1000 | 0.390616374453749 |
| 86.7983999999992 | 1025.38372741907 | 1000 | 0.390612421696991 |
| 86.8240799999992 | 1025.67816396315 | 1000 | 0.390608457086831 |
| 86.8497599999992 | 1025.97256837563 | 1000 | 0.390604480575771 |
| 86.8754399999992 | 1026.26694052493 | 1000 | 0.390600492116125 |
| 86.9011199999992 | 1026.56128027892 | 1000 | 0.390596491660012 |
| 86.9267999999992 | 1026.85558750489 | 1000 | 0.390592479159364 |
| 86.9524799999992 | 1027.14986206958 | 1000 | 0.390588454565918 |
| 86.9781599999992 | 1027.44410383913 | 1000 | 0.390584417831219 |
| 87.0038399999992 | 1027.73831267916 | 1000 | 0.390580368906617 |
| 87.0295199999992 | 1028.03248845469 | 1000 | 0.390576307743269 |
| 87.0551999999993 | 1028.32663103015 | 1000 | 0.390572234292136 |
| 87.0808799999993 | 1028.62074026944 | 1000 | 0.390568148503982 |
| 87.1065599999993 | 1028.91481603584 | 1000 | 0.390564050329376 |
| 87.1322399999993 | 1029.20885819205 | 1000 | 0.390559939718688 |
| 87.1579199999993 | 1029.50286660023 | 1000 | 0.390555816622089 |
| 87.1835999999993 | 1029.79684112189 | 1000 | 0.390551680989552 |
| 87.2092799999993 | 1030.09078161800 | 1000 | 0.390547532770850 |
| 87.2349599999993 | 1030.38468794892 | 1000 | 0.390543371915553 |
| 87.2606399999993 | 1030.67855997442 | 1000 | 0.390539198373032 |
| 87.2863199999993 | 1030.97239755366 | 1000 | 0.390535012092455 |
| 87.3119999999993 | 1031.26620054522 | 1000 | 0.390530813022784 |
| 87.3376799999993 | 1031.55996880707 | 1000 | 0.390526601112781 |
| 87.3633599999994 | 1031.85370219657 | 1000 | 0.390522376311000 |
| 87.3890399999993 | 1032.14740057050 | 1000 | 0.390518138565791 |
| 87.4147199999994 | 1032.44106378499 | 1000 | 0.390513887825297 |
| 87.4403999999994 | 1032.73469169559 | 1000 | 0.390509624037453 |
| 87.4660799999994 | 1033.02828415722 | 1000 | 0.390505347149986 |
| 87.4917599999994 | 1033.32184102419 | 1000 | 0.390501057110415 |
| 87.5174399999994 | 1033.61536215018 | 1000 | 0.390496753866049 |
| 87.5431199999994 | 1033.90884738827 | 1000 | 0.390492437363985 |
| 87.5687999999994 | 1034.20229659090 | 1000 | 0.390488107551109 |
| 87.5944799999994 | 1034.49570960988 | 1000 | 0.390483764374095 |
| 87.6201599999994 | 1034.78908629639 | 1000 | 0.390479407779405 |
| 87.6458399999994 | 1035.08242650098 | 1000 | 0.390475037713284 |
| 87.6715199999995 | 1035.37573007357 | 1000 | 0.390470654121765 |
| 87.6971999999994 | 1035.66899686344 | 1000 | 0.390466256950662 |
| 87.7228799999995 | 1035.96222671922 | 1000 | 0.390461846145577 |
| 87.7485599999995 | 1036.25541948891 | 1000 | 0.390457421651891 |
| 87.7742399999995 | 1036.54857501985 | 1000 | 0.390452983414766 |
| 87.7999199999995 | 1036.84169315875 | 1000 | 0.390448531379148 |
| 87.8255999999995 | 1037.13477375164 | 1000 | 0.390444065489760 |
| 87.8512799999995 | 1037.42781664391 | 1000 | 0.390439585691106 |
| 87.8769599999995 | 1037.72082168031 | 1000 | 0.390435091927467 |
| 87.9026399999995 | 1038.01378870490 | 1000 | 0.390430584142902 |
| 87.9283199999995 | 1038.30671756110 | 1000 | 0.390426062281246 |
| 87.9539999999995 | 1038.59960809165 | 1000 | 0.390421526286109 |
| 87.9796799999995 | 1038.89246013863 | 1000 | 0.390416976100876 |
| 88.0053599999995 | 1039.18527354345 | 1000 | 0.390412411668707 |
| 88.0310399999996 | 1039.47804814682 | 1000 | 0.390407832932532 |
| 88.0567199999996 | 1039.77078378882 | 1000 | 0.390403239835055 |
| 88.0823999999996 | 1040.06348030881 | 1000 | 0.390398632318751 |
| 88.1080799999996 | 1040.35613754548 | 1000 | 0.390394010325863 |
| 88.1337599999996 | 1040.64875533685 | 1000 | 0.390389373798407 |
| 88.1594399999996 | 1040.94133352022 | 1000 | 0.390384722678163 |
| 88.1851199999996 | 1041.23387193222 | 1000 | 0.390380056906681 |
| 88.2107999999996 | 1041.52637040879 | 1000 | 0.390375376425277 |
| 88.2364799999996 | 1041.81882878516 | 1000 | 0.390370681175031 |
| 88.2621599999996 | 1042.11124689586 | 1000 | 0.390365971096790 |
| 88.2878399999996 | 1042.40362457472 | 1000 | 0.390361246131162 |
| 88.3135199999996 | 1042.69596165486 | 1000 | 0.390356506218519 |
| 88.3391999999996 | 1042.98825796871 | 1000 | 0.390351751298995 |
| 88.3648799999997 | 1043.28051334796 | 1000 | 0.390346981312484 |
| 88.3905599999997 | 1043.57272762360 | 1000 | 0.390342196198640 |
| 88.4162399999997 | 1043.86490062590 | 1000 | 0.390337395896876 |
| 88.4419199999997 | 1044.15703218441 | 1000 | 0.390332580346363 |
| 88.4675999999997 | 1044.44912212795 | 1000 | 0.390327749486029 |
| 88.4932799999997 | 1044.74117028461 | 1000 | 0.390322903254557 |
| 88.5189599999997 | 1045.03317648176 | 1000 | 0.390318041590386 |
| 88.5446399999997 | 1045.32514054604 | 1000 | 0.390313164431710 |
| 88.5703199999997 | 1045.61706230334 | 1000 | 0.390308271716473 |
| 88.5959999999997 | 1045.90894157880 | 1000 | 0.390303363382373 |
| 88.6216799999997 | 1046.20077819686 | 1000 | 0.390298439366860 |
| 88.6473599999997 | 1046.49257198116 | 1000 | 0.390293499607132 |
| 88.6730399999997 | 1046.78432275463 | 1000 | 0.390288544040137 |
| 88.6987199999998 | 1047.07603033943 | 1000 | 0.390283572602572 |
| 88.7243999999998 | 1047.36769455698 | 1000 | 0.390278585230879 |
| 88.7500799999998 | 1047.65931522791 | 1000 | 0.390273581861248 |
| 88.7757599999998 | 1047.95089217212 | 1000 | 0.390268562429613 |
| 88.8014399999998 | 1048.24242520873 | 1000 | 0.390263526871653 |
| 88.8271199999998 | 1048.53391415610 | 1000 | 0.390258475122789 |
| 88.8527999999998 | 1048.82535883180 | 1000 | 0.390253407118185 |
| 88.8784799999998 | 1049.11675905265 | 1000 | 0.390248322792744 |
| 88.9041599999998 | 1049.40811463467 | 1000 | 0.390243222081112 |
| 88.9298399999998 | 1049.69942539311 | 1000 | 0.390238104917672 |
| 88.9555199999998 | 1049.99069114243 | 1000 | 0.390232971236544 |
| 88.9811999999998 | 1050.28191169631 | 1000 | 0.390227820971587 |
| 89.0068799999999 | 1050.57308686763 | 1000 | 0.390222654056395 |
| 89.0325599999999 | 1050.86421646848 | 1000 | 0.390217470424296 |
| 89.0582399999999 | 1051.15530031014 | 1000 | 0.390212270008353 |
| 89.0839199999999 | 1051.44633820310 | 1000 | 0.390207052741360 |
| 89.1095999999999 | 1051.73732995706 | 1000 | 0.390201818555845 |
| 89.1352799999999 | 1052.02827538088 | 1000 | 0.390196567384063 |
| 89.1609599999999 | 1052.31917428262 | 1000 | 0.390191299158001 |
| 89.1866399999999 | 1052.61002646953 | 1000 | 0.390186013809375 |
| 89.2123199999999 | 1052.90083174804 | 1000 | 0.390180711269626 |
| 89.2379999999999 | 1053.19158992376 | 1000 | 0.390175391469923 |
| 89.2636799999999 | 1053.48230080147 | 1000 | 0.390170054341159 |
| 89.2893599999999 | 1053.77296418511 | 1000 | 0.390164699813951 |
| 89.3150399999999 | 1054.06357987781 | 1000 | 0.390159327818639 |
| 89.3407200000000 | 1054.35414768186 | 1000 | 0.390153938285286 |
| 89.3664000000000 | 1054.64466739868 | 1000 | 0.390148531143675 |
| 89.3920800000000 | 1054.93513882889 | 1000 | 0.390143106323308 |
| 89.4177600000000 | 1055.22556177223 | 1000 | 0.390137663753405 |
| 89.4434400000000 | 1055.51593602761 | 1000 | 0.390132203362906 |
| 89.4691200000000 | 1055.80626139308 | 1000 | 0.390126725080465 |
| 89.4948000000000 | 1056.09653766582 | 1000 | 0.390121228834450 |
| 89.5204800000000 | 1056.38676464217 | 1000 | 0.390115714552947 |
| 89.5461600000000 | 1056.67694211759 | 1000 | 0.390110182163752 |
| 89.5718400000000 | 1056.96706988667 | 1000 | 0.390104631594373 |
| 89.5975200000000 | 1057.25714774315 | 1000 | 0.390099062772028 |
| 89.6232000000000 | 1057.54717547986 | 1000 | 0.390093475623646 |
| 89.6488800000000 | 1057.83715288878 | 1000 | 0.390087870075863 |
| 89.6745600000001 | 1058.12707976099 | 1000 | 0.390082246055023 |
| 89.7002400000001 | 1058.41695588668 | 1000 | 0.390076603487175 |
| 89.7259200000001 | 1058.70678105518 | 1000 | 0.390070942298074 |
| 89.7516000000001 | 1058.99655505487 | 1000 | 0.390065262413178 |
| 89.7772800000001 | 1059.28627767329 | 1000 | 0.390059563757646 |
| 89.8029600000001 | 1059.57594869705 | 1000 | 0.390053846256341 |
| 89.8286400000001 | 1059.86556791184 | 1000 | 0.390048109833825 |
| 89.8543200000001 | 1060.15513510246 | 1000 | 0.390042354414356 |
| 89.8800000000001 | 1060.44465005280 | 1000 | 0.390036579921895 |
| 89.9056800000001 | 1060.73411254582 | 1000 | 0.390030786280095 |
| 89.9313600000001 | 1061.02352236357 | 1000 | 0.390024973412306 |
| 89.9570400000001 | 1061.31287928715 | 1000 | 0.390019141241573 |
| 89.9827200000001 | 1061.60218309677 | 1000 | 0.390013289690632 |
| 90.0084000000002 | 1061.89143357168 | 1000 | 0.390007418681912 |
| 90.0340800000002 | 1062.18063049018 | 1000 | 0.390001528137530 |
| 90.0597600000002 | 1062.46977362967 | 1000 | 0.389995617979297 |
| 90.0854400000002 | 1062.75886276658 | 1000 | 0.389989688128706 |
| 90.1111200000002 | 1063.04789767638 | 1000 | 0.389983738506942 |
| 90.1368000000002 | 1063.33687813361 | 1000 | 0.389977769034872 |
| 90.1624800000002 | 1063.62580391184 | 1000 | 0.389971779633049 |
| 90.1881600000002 | 1063.91467478369 | 1000 | 0.389965770221707 |
| 90.2138400000002 | 1064.20349052079 | 1000 | 0.389959740720764 |
| 90.2395200000002 | 1064.49225089384 | 1000 | 0.389953691049817 |
| 90.2652000000002 | 1064.78095567252 | 1000 | 0.389947621128143 |
| 90.2908800000002 | 1065.06960462557 | 1000 | 0.389941530874696 |
| 90.3165600000003 | 1065.35819752074 | 1000 | 0.389935420208108 |
| 90.3422400000003 | 1065.64673412479 | 1000 | 0.389929289046684 |
| 90.3679200000003 | 1065.93521420347 | 1000 | 0.389923137308406 |
| 90.3936000000003 | 1066.22363752158 | 1000 | 0.389916964910926 |
| 90.4192800000003 | 1066.51200384288 | 1000 | 0.389910771771570 |
| 90.4449600000003 | 1066.80031293016 | 1000 | 0.389904557807331 |
| 90.4706400000003 | 1067.08856454517 | 1000 | 0.389898322934874 |
| 90.4963200000003 | 1067.37675844868 | 1000 | 0.389892067070531 |
| 90.5220000000003 | 1067.66489440043 | 1000 | 0.389885790130297 |
| 90.5476800000003 | 1067.95297215914 | 1000 | 0.389879492029837 |
| 90.5733600000003 | 1068.24099148250 | 1000 | 0.389873172684475 |
| 90.5990400000003 | 1068.52895212720 | 1000 | 0.389866832009200 |
| 90.6247200000003 | 1068.81685384887 | 1000 | 0.389860469918661 |
| 90.6504000000004 | 1069.10469640210 | 1000 | 0.389854086327167 |
| 90.6760800000004 | 1069.39247954046 | 1000 | 0.389847681148683 |
| 90.7017600000004 | 1069.68020301646 | 1000 | 0.389841254296835 |
| 90.7274400000004 | 1069.96786658156 | 1000 | 0.389834805684900 |
| 90.7531200000004 | 1070.25546998618 | 1000 | 0.389828335225813 |
| 90.7788000000004 | 1070.54301297966 | 1000 | 0.389821842832158 |
| 90.8044800000004 | 1070.83049531030 | 1000 | 0.389815328416174 |
| 90.8301600000004 | 1071.11791672530 | 1000 | 0.389808791889748 |
| 90.8558400000004 | 1071.40527697082 | 1000 | 0.389802233164416 |
| 90.8815200000004 | 1071.69257579193 | 1000 | 0.389795652151360 |
| 90.9072000000004 | 1071.97981293261 | 1000 | 0.389789048761410 |
| 90.9328800000004 | 1072.26698813576 | 1000 | 0.389782422905039 |
| 90.9585600000005 | 1072.55410114320 | 1000 | 0.389775774492363 |
| 90.9842400000004 | 1072.84115169565 | 1000 | 0.389769103433141 |
| 91.0099200000005 | 1073.12813953271 | 1000 | 0.389762409636771 |
| 91.0356000000005 | 1073.41506439290 | 1000 | 0.389755693012288 |
| 91.0612800000005 | 1073.70192601362 | 1000 | 0.389748953468368 |
| 91.0869600000005 | 1073.98872413116 | 1000 | 0.389742190913321 |
| 91.1126400000005 | 1074.27545848069 | 1000 | 0.389735405255090 |
| 91.1383200000005 | 1074.56212879626 | 1000 | 0.389728596401254 |
| 91.1640000000005 | 1074.84873481077 | 1000 | 0.389721764259021 |
| 91.1896800000005 | 1075.13527625603 | 1000 | 0.389714908735231 |
| 91.2153600000005 | 1075.42175286268 | 1000 | 0.389708029736350 |
| 91.2410400000005 | 1075.70816436021 | 1000 | 0.389701127168475 |
| 91.2667200000006 | 1075.99451047700 | 1000 | 0.389694200937324 |
| 91.2924000000005 | 1076.28079094024 | 1000 | 0.389687250948244 |
| 91.3180800000006 | 1076.56700547598 | 1000 | 0.389680277106200 |
| 91.3437600000006 | 1076.85315380912 | 1000 | 0.389673279315783 |
| 91.3694400000006 | 1077.13923566337 | 1000 | 0.389666257481199 |
| 91.3951200000006 | 1077.42525076127 | 1000 | 0.389659211506275 |
| 91.4208000000006 | 1077.71119882421 | 1000 | 0.389652141294455 |
| 91.4464800000006 | 1077.99707957237 | 1000 | 0.389645046748796 |
| 91.4721600000006 | 1078.28289272476 | 1000 | 0.389637927771971 |
| 91.4978400000006 | 1078.56863799919 | 1000 | 0.389630784266263 |
| 91.5235200000006 | 1078.85431511227 | 1000 | 0.389623616133567 |
| 91.5492000000006 | 1079.13992377942 | 1000 | 0.389616423275387 |
| 91.5748800000006 | 1079.42546371484 | 1000 | 0.389609205592834 |
| 91.6005600000006 | 1079.71093463153 | 1000 | 0.389601962986624 |
| 91.6262400000007 | 1079.99633624127 | 1000 | 0.389594695357080 |
| 91.6519200000007 | 1080.28166825462 | 1000 | 0.389587402604124 |
| 91.6776000000007 | 1080.56693038090 | 1000 | 0.389580084627284 |
| 91.7032800000007 | 1080.85212232821 | 1000 | 0.389572741325684 |
| 91.7289600000007 | 1081.13724380341 | 1000 | 0.389565372598047 |
| 91.7546400000007 | 1081.42229451212 | 1000 | 0.389557978342692 |
| 91.7803200000007 | 1081.70727415870 | 1000 | 0.389550558457536 |
| 91.8060000000007 | 1081.99218244627 | 1000 | 0.389543112840085 |
| 91.8316800000007 | 1082.27701907668 | 1000 | 0.389535641387438 |
| 91.8573600000007 | 1082.56178375053 | 1000 | 0.389528143996287 |
| 91.8830400000007 | 1082.84647616713 | 1000 | 0.389520620562908 |
| 91.9087200000007 | 1083.13109602455 | 1000 | 0.389513070983166 |
| 91.9344000000007 | 1083.41564301954 | 1000 | 0.389505495152513 |
| 91.9600800000008 | 1083.70011684759 | 1000 | 0.389497892965981 |
| 91.9857600000008 | 1083.98451720288 | 1000 | 0.389490264318186 |
| 92.0114400000008 | 1084.26884377832 | 1000 | 0.389482609103324 |
| 92.0371200000008 | 1084.55309626550 | 1000 | 0.389474927215170 |
| 92.0628000000008 | 1084.83727435471 | 1000 | 0.389467218547074 |
| 92.0884800000008 | 1085.12137773491 | 1000 | 0.389459482991963 |
| 92.1141600000008 | 1085.40540609377 | 1000 | 0.389451720442338 |
| 92.1398400000008 | 1085.68935911762 | 1000 | 0.389443930790271 |
| 92.1655200000008 | 1085.97323649145 | 1000 | 0.389436113927403 |
| 92.1912000000008 | 1086.25703789893 | 1000 | 0.389428269744947 |
| 92.2168800000008 | 1086.54076302240 | 1000 | 0.389420398133678 |
| 92.2425600000008 | 1086.82441154283 | 1000 | 0.389412498983941 |
| 92.2682400000008 | 1087.10798313984 | 1000 | 0.389404572185640 |
| 92.2939200000009 | 1087.39147749172 | 1000 | 0.389396617628244 |
| 92.3196000000009 | 1087.67489427536 | 1000 | 0.389388635200781 |
| 92.3452800000009 | 1087.95823316630 | 1000 | 0.389380624791836 |
| 92.3709600000009 | 1088.24149383870 | 1000 | 0.389372586289551 |
| 92.3966400000009 | 1088.52467596535 | 1000 | 0.389364519581624 |
| 92.4223200000009 | 1088.80777921765 | 1000 | 0.389356424555303 |
| 92.4480000000009 | 1089.09080326558 | 1000 | 0.389348301097391 |
| 92.4736800000009 | 1089.37374777777 | 1000 | 0.389340149094238 |
| 92.4993600000009 | 1089.65661242140 | 1000 | 0.389331968431741 |
| 92.5250400000009 | 1089.93939686227 | 1000 | 0.389323758995344 |
| 92.5507200000009 | 1090.22210076475 | 1000 | 0.389315520670036 |
| 92.5764000000009 | 1090.50472379180 | 1000 | 0.389307253340346 |
| 92.6020800000010 | 1090.78726560493 | 1000 | 0.389298956890345 |
| 92.6277600000010 | 1091.06972586424 | 1000 | 0.389290631203642 |
| 92.6534400000010 | 1091.35210422837 | 1000 | 0.389282276163383 |
| 92.6791200000010 | 1091.63440035453 | 1000 | 0.389273891652248 |
| 92.7048000000010 | 1091.91661389847 | 1000 | 0.389265477552452 |
| 92.7304800000010 | 1092.19874451449 | 1000 | 0.389257033745740 |
| 92.7561600000010 | 1092.48079185540 | 1000 | 0.389248560113387 |
| 92.7818400000010 | 1092.76275557257 | 1000 | 0.389240056536194 |
| 92.8075200000010 | 1093.04463531587 | 1000 | 0.389231522894491 |
| 92.8332000000010 | 1093.32643073371 | 1000 | 0.389222959068129 |
| 92.8588800000010 | 1093.60814147299 | 1000 | 0.389214364936481 |
| 92.8845600000010 | 1093.88976717912 | 1000 | 0.389205740378442 |
| 92.9102400000011 | 1094.17130749601 | 1000 | 0.389197085272423 |
| 92.9359200000011 | 1094.45276206607 | 1000 | 0.389188399496354 |
| 92.9616000000011 | 1094.73413053018 | 1000 | 0.389179682927676 |
| 92.9872800000011 | 1095.01541252771 | 1000 | 0.389170935443346 |
| 93.0129600000011 | 1095.29660769648 | 1000 | 0.389162156919829 |
| 93.0386400000011 | 1095.57771567282 | 1000 | 0.389153347233098 |
| 93.0643200000011 | 1095.85873609148 | 1000 | 0.389144506258637 |
| 93.0900000000011 | 1096.13966858568 | 1000 | 0.389135633871430 |
| 93.1156800000011 | 1096.42051278708 | 1000 | 0.389126729945966 |
| 93.1413600000011 | 1096.70126832579 | 1000 | 0.389117794356234 |
| 93.1670400000011 | 1096.98193483034 | 1000 | 0.389108826975722 |
| 93.1927200000011 | 1097.26251192770 | 1000 | 0.389099827677417 |
| 93.2184000000011 | 1097.54299924325 | 1000 | 0.389090796333798 |
| 93.2440800000011 | 1097.82339640078 | 1000 | 0.389081732816838 |
| 93.2697600000012 | 1098.10370302251 | 1000 | 0.389072636998001 |
| 93.2954400000012 | 1098.38391872904 | 1000 | 0.389063508748240 |
| 93.3211200000012 | 1098.66404313936 | 1000 | 0.389054347937994 |
| 93.3468000000012 | 1098.94407587087 | 1000 | 0.389045154437189 |
| 93.3724800000012 | 1099.22401653932 | 1000 | 0.389035928115231 |
| 93.3981600000012 | 1099.50386475885 | 1000 | 0.389026668841010 |
| 93.4238400000012 | 1099.78362014197 | 1000 | 0.389017376482891 |
| 93.4495200000012 | 1100.06328229954 | 1000 | 0.389008050908720 |
| 93.4752000000012 | 1100.34285084078 | 1000 | 0.388998691985814 |
| 93.5008800000012 | 1100.62232537324 | 1000 | 0.388989299580964 |
| 93.5265600000012 | 1100.90170550284 | 1000 | 0.388979873560432 |
| 93.5522400000012 | 1101.18099083378 | 1000 | 0.388970413789949 |
| 93.5779200000012 | 1101.46018096865 | 1000 | 0.388960920134709 |
| 93.6036000000013 | 1101.73927550829 | 1000 | 0.388951392459374 |
| 93.6292800000013 | 1102.01827405191 | 1000 | 0.388941830628067 |
| 93.6549600000013 | 1102.29717619699 | 1000 | 0.388932234504370 |
| 93.6806400000013 | 1102.57598153930 | 1000 | 0.388922603951324 |
| 93.7063200000013 | 1102.85468967292 | 1000 | 0.388912938831425 |
| 93.7320000000013 | 1103.13330019021 | 1000 | 0.388903239006623 |
| 93.7576800000013 | 1103.41181268179 | 1000 | 0.388893504338320 |
| 93.7833600000013 | 1103.69022673655 | 1000 | 0.388883734687366 |
| 93.8090400000013 | 1103.96854194165 | 1000 | 0.388873929914059 |
| 93.8347200000013 | 1104.24675788250 | 1000 | 0.388864089878143 |
| 93.8604000000013 | 1104.52487414275 | 1000 | 0.388854214438802 |
| 93.8860800000013 | 1104.80289030429 | 1000 | 0.388844303454663 |
| 93.9117600000014 | 1105.08080594724 | 1000 | 0.388834356783791 |
| 93.9374400000014 | 1105.35862064994 | 1000 | 0.388824374283687 |
| 93.9631200000014 | 1105.63633398895 | 1000 | 0.388814355811285 |
| 93.9888000000014 | 1105.91394553905 | 1000 | 0.388804301222952 |
| 94.0144800000014 | 1106.19145487319 | 1000 | 0.388794210374483 |
| 94.0401600000014 | 1106.46886156253 | 1000 | 0.388784083121104 |
| 94.0658400000014 | 1106.74616517643 | 1000 | 0.388773919317461 |
| 94.0915200000014 | 1107.02336528240 | 1000 | 0.388763718817626 |
| 94.1172000000014 | 1107.30046144613 | 1000 | 0.388753481475091 |
| 94.1428800000014 | 1107.57745323149 | 1000 | 0.388743207142765 |
| 94.1685600000014 | 1107.85434020048 | 1000 | 0.388732895672975 |
| 94.1942400000014 | 1108.13112191325 | 1000 | 0.388722546917460 |
| 94.2199200000015 | 1108.40779792811 | 1000 | 0.388712160727370 |
| 94.2456000000015 | 1108.68436780147 | 1000 | 0.388701736953266 |
| 94.2712800000015 | 1108.96083108789 | 1000 | 0.388691275445114 |
| 94.2969600000015 | 1109.23718734003 | 1000 | 0.388680776052285 |
| 94.3226400000015 | 1109.51343610867 | 1000 | 0.388670238623553 |
| 94.3483200000015 | 1109.78957694266 | 1000 | 0.388659663007088 |
| 94.3740000000015 | 1110.06560938898 | 1000 | 0.388649049050463 |
| 94.3996800000015 | 1110.34153299268 | 1000 | 0.388638396600642 |
| 94.4253600000015 | 1110.61734729686 | 1000 | 0.388627705503981 |
| 94.4510400000015 | 1110.89305184273 | 1000 | 0.388616975606230 |
| 94.4767200000015 | 1111.16864616953 | 1000 | 0.388606206752524 |
| 94.5024000000015 | 1111.44412981455 | 1000 | 0.388595398787384 |
| 94.5280800000015 | 1111.71950231314 | 1000 | 0.388584551554714 |
| 94.5537600000016 | 1111.99476319867 | 1000 | 0.388573664897798 |
| 94.5794400000016 | 1112.26991200255 | 1000 | 0.388562738659300 |
| 94.6051200000016 | 1112.54494825419 | 1000 | 0.388551772681258 |
| 94.6308000000016 | 1112.81987148102 | 1000 | 0.388540766805083 |
| 94.6564800000016 | 1113.09468120848 | 1000 | 0.388529720871560 |
| 94.6821600000016 | 1113.36937695998 | 1000 | 0.388518634720838 |
| 94.7078400000016 | 1113.64395825694 | 1000 | 0.388507508192434 |
| 94.7335200000016 | 1113.91842461873 | 1000 | 0.388496341125229 |
| 94.7592000000016 | 1114.19277556271 | 1000 | 0.388485133357463 |
| 94.7848800000016 | 1114.46701060419 | 1000 | 0.388473884726738 |
| 94.8105600000016 | 1114.74112925642 | 1000 | 0.388462595070007 |
| 94.8362400000016 | 1115.01513103060 | 1000 | 0.388451264223579 |
| 94.8619200000017 | 1115.28901543588 | 1000 | 0.388439892023115 |
| 94.8876000000016 | 1115.56278197931 | 1000 | 0.388428478303622 |
| 94.9132800000017 | 1115.83643016586 | 1000 | 0.388417022899454 |
| 94.9389600000017 | 1116.10995949841 | 1000 | 0.388405525644308 |
| 94.9646400000017 | 1116.38336947774 | 1000 | 0.388393986371220 |
| 94.9903200000017 | 1116.65665960252 | 1000 | 0.388382404912566 |
| 95.0160000000017 | 1116.92982936929 | 1000 | 0.388370781100058 |
| 95.0416800000017 | 1117.20287827248 | 1000 | 0.388359114764738 |
| 95.0673600000017 | 1117.47580580436 | 1000 | 0.388347405736980 |
| 95.0930400000017 | 1117.74861145507 | 1000 | 0.388335653846487 |
| 95.1187200000017 | 1118.02129471260 | 1000 | 0.388323858922283 |
| 95.1444000000017 | 1118.29385506274 | 1000 | 0.388312020792718 |
| 95.1700800000017 | 1118.56629198915 | 1000 | 0.388300139285460 |
| 95.1957600000017 | 1118.83860497328 | 1000 | 0.388288214227494 |
| 95.2214400000018 | 1119.11079349439 | 1000 | 0.388276245445120 |
| 95.2471200000018 | 1119.38285702955 | 1000 | 0.388264232763949 |
| 95.2728000000018 | 1119.65479505362 | 1000 | 0.388252176008901 |
| 95.2984800000018 | 1119.92660703922 | 1000 | 0.388240075004203 |
| 95.3241600000018 | 1120.19829245676 | 1000 | 0.388227929573384 |
| 95.3498400000018 | 1120.46985077441 | 1000 | 0.388215739539276 |
| 95.3755200000018 | 1120.74128145807 | 1000 | 0.388203504724007 |
| 95.4012000000018 | 1121.01258397143 | 1000 | 0.388191224949000 |
| 95.4268800000018 | 1121.28375777585 | 1000 | 0.388178900034974 |
| 95.4525600000018 | 1121.55480233047 | 1000 | 0.388166529801934 |
| 95.4782400000018 | 1121.82571709211 | 1000 | 0.388154114069174 |
| 95.5039200000018 | 1122.09650151531 | 1000 | 0.388141652655271 |
| 95.5296000000019 | 1122.36715505229 | 1000 | 0.388129145378086 |
| 95.5552800000019 | 1122.63767715298 | 1000 | 0.388116592054755 |
| 95.5809600000019 | 1122.90806726495 | 1000 | 0.388103992501693 |
| 95.6066400000019 | 1123.17832483348 | 1000 | 0.388091346534586 |
| 95.6323200000019 | 1123.44844930146 | 1000 | 0.388078653968391 |
| 95.6580000000019 | 1123.71844010945 | 1000 | 0.388065914617332 |
| 95.6836800000019 | 1123.98829669565 | 1000 | 0.388053128294898 |
| 95.7093600000019 | 1124.25801849588 | 1000 | 0.388040294813839 |
| 95.7350400000019 | 1124.52760494357 | 1000 | 0.388027413986164 |
| 95.7607200000019 | 1124.79705546976 | 1000 | 0.388014485623137 |
| 95.7864000000019 | 1125.06636950309 | 1000 | 0.388001509535276 |
| 95.8120800000019 | 1125.33554646978 | 1000 | 0.387988485532348 |
| 95.8377600000019 | 1125.60458579363 | 1000 | 0.387975413423369 |
| 95.8634400000019 | 1125.87348689601 | 1000 | 0.387962293016596 |
| 95.8891200000020 | 1126.14224919583 | 1000 | 0.387949124119529 |
| 95.9148000000020 | 1126.41087210956 | 1000 | 0.387935906538908 |
| 95.9404800000020 | 1126.67935505120 | 1000 | 0.387922640080704 |
| 95.9661600000020 | 1126.94769743227 | 1000 | 0.387909324550123 |
| 95.9918400000020 | 1127.21589866181 | 1000 | 0.387895959751600 |
| 96.0175200000020 | 1127.48395814637 | 1000 | 0.387882545488797 |
| 96.0432000000020 | 1127.75187528997 | 1000 | 0.387869081564597 |
| 96.0688800000020 | 1128.01964949413 | 1000 | 0.387855567781104 |
| 96.0945600000020 | 1128.28728015785 | 1000 | 0.387842003939641 |
| 96.1202400000020 | 1128.55476667758 | 1000 | 0.387828389840743 |
| 96.1459200000020 | 1128.82210844720 | 1000 | 0.387814725284157 |
| 96.1716000000020 | 1129.08930485808 | 1000 | 0.387801010068838 |
| 96.1972800000021 | 1129.35635529897 | 1000 | 0.387787243992945 |
| 96.2229600000021 | 1129.62325915607 | 1000 | 0.387773426853840 |
| 96.2486400000021 | 1129.89001581298 | 1000 | 0.387759558448083 |
| 96.2743200000021 | 1130.15662465068 | 1000 | 0.387745638571429 |
| 96.3000000000021 | 1130.42308504756 | 1000 | 0.387731667018826 |
| 96.3256800000021 | 1130.68939637937 | 1000 | 0.387717643584414 |
| 96.3513600000021 | 1130.95555801923 | 1000 | 0.387703568061514 |
| 96.3770400000021 | 1131.22156933761 | 1000 | 0.387689440242633 |
| 96.4027200000021 | 1131.48742970232 | 1000 | 0.387675259919458 |
| 96.4284000000021 | 1131.75313847851 | 1000 | 0.387661026882851 |
| 96.4540800000021 | 1132.01869502864 | 1000 | 0.387646740922848 |
| 96.4797600000021 | 1132.28409871249 | 1000 | 0.387632401828657 |
| 96.5054400000022 | 1132.54934888711 | 1000 | 0.387618009388651 |
| 96.5311200000022 | 1132.81444490686 | 1000 | 0.387603563390366 |
| 96.5568000000022 | 1133.07938612337 | 1000 | 0.387589063620501 |
| 96.5824800000022 | 1133.34417188552 | 1000 | 0.387574509864910 |
| 96.6081600000022 | 1133.60880153947 | 1000 | 0.387559901908601 |
| 96.6338400000022 | 1133.87327442859 | 1000 | 0.387545239535735 |
| 96.6595200000022 | 1134.13758989348 | 1000 | 0.387530522529617 |
| 96.6852000000022 | 1134.40174727197 | 1000 | 0.387515750672698 |
| 96.7108800000022 | 1134.66574589910 | 1000 | 0.387500923746571 |
| 96.7365600000022 | 1134.92958510708 | 1000 | 0.387486041531962 |
| 96.7622400000022 | 1135.19326422532 | 1000 | 0.387471103808736 |
| 96.7879200000022 | 1135.45678258040 | 1000 | 0.387456110355884 |
| 96.8136000000022 | 1135.72013949603 | 1000 | 0.387441060951529 |
| 96.8392800000022 | 1135.98333429310 | 1000 | 0.387425955372914 |
| 96.8649600000023 | 1136.24636628963 | 1000 | 0.387410793396403 |
| 96.8906400000023 | 1136.50923480073 | 1000 | 0.387395574797480 |
| 96.9163200000023 | 1136.77193913865 | 1000 | 0.387380299350740 |
| 96.9420000000023 | 1137.03447861274 | 1000 | 0.387364966829887 |
| 96.9676800000023 | 1137.29685252941 | 1000 | 0.387349577007735 |
| 96.9933600000023 | 1137.55906019216 | 1000 | 0.387334129656200 |
| 97.0190400000023 | 1137.82110090155 | 1000 | 0.387318624546296 |
| 97.0447200000023 | 1138.08297395518 | 1000 | 0.387303061448136 |
| 97.0704000000023 | 1138.34467864770 | 1000 | 0.387287440130924 |
| 97.0960800000023 | 1138.60621427077 | 1000 | 0.387271760362954 |
| 97.1217600000023 | 1138.86758011307 | 1000 | 0.387256021911605 |
| 97.1474400000023 | 1139.12877546028 | 1000 | 0.387240224543340 |
| 97.1731200000023 | 1139.38979959505 | 1000 | 0.387224368023698 |
| 97.1988000000024 | 1139.65065179703 | 1000 | 0.387208452117296 |
| 97.2244800000024 | 1139.91133134280 | 1000 | 0.387192476587820 |
| 97.2501600000024 | 1140.17183750592 | 1000 | 0.387176441198024 |
| 97.2758400000024 | 1140.43216955685 | 1000 | 0.387160345709728 |
| 97.3015200000024 | 1140.69232676301 | 1000 | 0.387144189883812 |
| 97.3272000000024 | 1140.95230838870 | 1000 | 0.387127973480212 |
| 97.3528800000024 | 1141.21211369513 | 1000 | 0.387111696257919 |
| 97.3785600000024 | 1141.47174194039 | 1000 | 0.387095357974971 |
| 97.4042400000024 | 1141.73119237944 | 1000 | 0.387078958388456 |
| 97.4299200000024 | 1141.99046426410 | 1000 | 0.387062497254501 |
| 97.4556000000024 | 1142.24955684304 | 1000 | 0.387045974328272 |
| 97.4812800000024 | 1142.50846936174 | 1000 | 0.387029389363971 |
| 97.5069600000025 | 1142.76720106252 | 1000 | 0.387012742114832 |
| 97.5326400000025 | 1143.02575118450 | 1000 | 0.386996032333114 |
| 97.5583200000025 | 1143.28411896358 | 1000 | 0.386979259770101 |
| 97.5840000000025 | 1143.54230363244 | 1000 | 0.386962424176097 |
| 97.6096800000025 | 1143.80030442054 | 1000 | 0.386945525300421 |
| 97.6353600000025 | 1144.05812055407 | 1000 | 0.386928562891406 |
| 97.6610400000025 | 1144.31575125597 | 1000 | 0.386911536696392 |
| 97.6867200000025 | 1144.57319574590 | 1000 | 0.386894446461724 |
| 97.7124000000025 | 1144.83045324022 | 1000 | 0.386877291932748 |
| 97.7380800000025 | 1145.08752295200 | 1000 | 0.386860072853807 |
| 97.7637600000025 | 1145.34440409099 | 1000 | 0.386842788968237 |
| 97.7894400000025 | 1145.60109586359 | 1000 | 0.386825440018363 |
| 97.8151200000026 | 1145.85759747289 | 1000 | 0.386808025745495 |
| 97.8408000000026 | 1146.11390811857 | 1000 | 0.386790545889924 |
| 97.8664800000026 | 1146.37002699699 | 1000 | 0.386773000190921 |
| 97.8921600000026 | 1146.62595330108 | 1000 | 0.386755388386727 |
| 97.9178400000026 | 1146.88168622038 | 1000 | 0.386737710214554 |
| 97.9435200000026 | 1147.13722494103 | 1000 | 0.386719965410580 |
| 97.9692000000026 | 1147.39256864572 | 1000 | 0.386702153709945 |
| 97.9948800000026 | 1147.64771651370 | 1000 | 0.386684274846743 |
| 98.0205600000026 | 1147.90266772076 | 1000 | 0.386666328554026 |
| 98.0462400000026 | 1148.15742143921 | 1000 | 0.386648314563793 |
| 98.0719200000026 | 1148.41197683789 | 1000 | 0.386630232606989 |
| 98.0976000000026 | 1148.66633308212 | 1000 | 0.386612082413500 |
| 98.1232800000026 | 1148.92048933370 | 1000 | 0.386593863712149 |
| 98.1489600000027 | 1149.17444475091 | 1000 | 0.386575576230695 |
| 98.1746400000027 | 1149.42819848847 | 1000 | 0.386557219695821 |
| 98.2003200000027 | 1149.68174969756 | 1000 | 0.386538793833140 |
| 98.2260000000027 | 1149.93509752574 | 1000 | 0.386520298367181 |
| 98.2516800000027 | 1150.18824111703 | 1000 | 0.386501733021395 |
| 98.2773600000027 | 1150.44117961181 | 1000 | 0.386483097518141 |
| 98.3030400000027 | 1150.69391214683 | 1000 | 0.386464391578688 |
| 98.3287200000027 | 1150.94643785524 | 1000 | 0.386445614923209 |
| 98.3544000000027 | 1151.19875586649 | 1000 | 0.386426767270777 |
| 98.3800800000027 | 1151.45086530641 | 1000 | 0.386407848339361 |
| 98.4057600000027 | 1151.70276529711 | 1000 | 0.386388857845820 |
| 98.4314400000027 | 1151.95445495702 | 1000 | 0.386369795505901 |
| 98.4571200000028 | 1152.20593340085 | 1000 | 0.386350661034234 |
| 98.4828000000027 | 1152.45719973958 | 1000 | 0.386331454144328 |
| 98.5084800000028 | 1152.70825308044 | 1000 | 0.386312174548565 |
| 98.5341600000028 | 1152.95909252692 | 1000 | 0.386292821958197 |
| 98.5598400000028 | 1153.20971717870 | 1000 | 0.386273396083343 |
| 98.5855200000028 | 1153.46012613169 | 1000 | 0.386253896632981 |
| 98.6112000000028 | 1153.71031847799 | 1000 | 0.386234323314948 |
| 98.6368800000028 | 1153.96029330588 | 1000 | 0.386214675835933 |
| 98.6625600000028 | 1154.21004969978 | 1000 | 0.386194953901471 |
| 98.6882400000028 | 1154.45958674027 | 1000 | 0.386175157215943 |
| 98.7139200000028 | 1154.70890350405 | 1000 | 0.386155285482568 |
| 98.7396000000028 | 1154.95799906395 | 1000 | 0.386135338403401 |
| 98.7652800000029 | 1155.20687248886 | 1000 | 0.386115315679325 |
| 98.7909600000028 | 1155.45552284378 | 1000 | 0.386095217010050 |
| 98.8166400000029 | 1155.70394918977 | 1000 | 0.386075042094108 |
| 98.8423200000029 | 1155.95215058391 | 1000 | 0.386054790628846 |
| 98.8680000000029 | 1156.20012607934 | 1000 | 0.386034462310425 |
| 98.8936800000029 | 1156.44787472519 | 1000 | 0.386014056833812 |
| 98.9193600000029 | 1156.69539556660 | 1000 | 0.385993573892779 |
| 98.9450400000029 | 1156.94268764469 | 1000 | 0.385973013179894 |
| 98.9707200000029 | 1157.18974999653 | 1000 | 0.385952374386520 |
| 98.9964000000029 | 1157.43658165514 | 1000 | 0.385931657202811 |
| 99.0220800000029 | 1157.68318164949 | 1000 | 0.385910861317703 |
| 99.0477600000029 | 1157.92954900444 | 1000 | 0.385889986418913 |
| 99.0734400000029 | 1158.17568274074 | 1000 | 0.385869032192933 |
| 99.0991200000029 | 1158.42158187503 | 1000 | 0.385847998325026 |
| 99.1248000000030 | 1158.66724541980 | 1000 | 0.385826884499221 |
| 99.1504800000030 | 1158.91267238341 | 1000 | 0.385805690398308 |
| 99.1761600000030 | 1159.15786177002 | 1000 | 0.385784415703833 |
| 99.2018400000030 | 1159.40281257959 | 1000 | 0.385763060096094 |
| 99.2275200000030 | 1159.64752380790 | 1000 | 0.385741623254136 |
| 99.2532000000030 | 1159.89199444649 | 1000 | 0.385720104855747 |
| 99.2788800000030 | 1160.13622348265 | 1000 | 0.385698504577449 |
| 99.3045600000030 | 1160.38020989940 | 1000 | 0.385676822094501 |
| 99.3302400000030 | 1160.62395267552 | 1000 | 0.385655057080886 |
| 99.3559200000030 | 1160.86745078544 | 1000 | 0.385633209209313 |
| 99.3816000000030 | 1161.11070319931 | 1000 | 0.385611278151204 |
| 99.4072800000030 | 1161.35370888293 | 1000 | 0.385589263576700 |
| 99.4329600000030 | 1161.59646679776 | 1000 | 0.385567165154646 |
| 99.4586400000030 | 1161.83897590087 | 1000 | 0.385544982552591 |
| 99.4843200000031 | 1162.08123514498 | 1000 | 0.385522715436783 |
| 99.5100000000031 | 1162.32324347836 | 1000 | 0.385500363472163 |
| 99.5356800000031 | 1162.56499984487 | 1000 | 0.385477926322359 |
| 99.5613600000031 | 1162.80650318395 | 1000 | 0.385455403649683 |
| 99.5870400000031 | 1163.04775243054 | 1000 | 0.385432795115127 |
| 99.6127200000031 | 1163.28874651512 | 1000 | 0.385410100378353 |
| 99.6384000000031 | 1163.52948436369 | 1000 | 0.385387319097693 |
| 99.6640800000031 | 1163.76996489768 | 1000 | 0.385364450930143 |
| 99.6897600000031 | 1164.01018703404 | 1000 | 0.385341495531355 |
| 99.7154400000031 | 1164.25014968512 | 1000 | 0.385318452555634 |
| 99.7411200000031 | 1164.48985175872 | 1000 | 0.385295321655933 |
| 99.7668000000031 | 1164.72929215804 | 1000 | 0.385272102483850 |
| 99.7924800000032 | 1164.96846978167 | 1000 | 0.385248794689615 |
| 99.8181600000032 | 1165.20738352356 | 1000 | 0.385225397922096 |
| 99.8438400000032 | 1165.44603227301 | 1000 | 0.385201911828785 |
| 99.8695200000032 | 1165.68441491467 | 1000 | 0.385178336055795 |
| 99.8952000000032 | 1165.92253032845 | 1000 | 0.385154670247857 |
| 99.9208800000032 | 1166.16037738961 | 1000 | 0.385130914048314 |
| 99.9465600000032 | 1166.39795496864 | 1000 | 0.385107067099114 |
| 99.9722400000032 | 1166.63526193129 | 1000 | 0.385083129040805 |
| 99.9979200000032 | 1166.87229713855 | 1000 | 0.385059099512531 |
| 100.023600000003 | 1167.10905944661 | 1000 | 0.385034978152026 |
| 100.049280000003 | 1167.34554770686 | 1000 | 0.385010764595609 |
| 100.074960000003 | 1167.58176076585 | 1000 | 0.384986458478179 |
| 100.100640000003 | 1167.81769746529 | 1000 | 0.384962059433207 |
| 100.126320000003 | 1168.05335664202 | 1000 | 0.384937567092734 |
| 100.152000000003 | 1168.28873712799 | 1000 | 0.384912981087363 |
| 100.177680000003 | 1168.52383775023 | 1000 | 0.384888301046256 |
| 100.203360000003 | 1168.75865733086 | 1000 | 0.384863526597126 |
| 100.229040000003 | 1168.99319468702 | 1000 | 0.384838657366233 |
| 100.254720000003 | 1169.22744863091 | 1000 | 0.384813692978379 |
| 100.280400000003 | 1169.46141796972 | 1000 | 0.384788633056899 |
| 100.306080000003 | 1169.69510150563 | 1000 | 0.384763477223661 |
| 100.331760000003 | 1169.92849803577 | 1000 | 0.384738225099057 |
| 100.357440000003 | 1170.16160635226 | 1000 | 0.384712876301996 |
| 100.383120000003 | 1170.39442524209 | 1000 | 0.384687430449903 |
| 100.408800000003 | 1170.62695348719 | 1000 | 0.384661887158708 |
| 100.434480000003 | 1170.85918986435 | 1000 | 0.384636246042844 |
| 100.460160000003 | 1171.09113314524 | 1000 | 0.384610506715242 |
| 100.485840000003 | 1171.32278209635 | 1000 | 0.384584668787322 |
| 100.511520000003 | 1171.55413547899 | 1000 | 0.384558731868988 |
| 100.537200000003 | 1171.78519204929 | 1000 | 0.384532695568626 |
| 100.562880000003 | 1172.01595055812 | 1000 | 0.384506559493093 |
| 100.588560000003 | 1172.24640975113 | 1000 | 0.384480323247715 |
| 100.614240000003 | 1172.47656836867 | 1000 | 0.384453986436280 |
| 100.639920000003 | 1172.70642514584 | 1000 | 0.384427548661031 |
| 100.665600000003 | 1172.93597881239 | 1000 | 0.384401009522662 |
| 100.691280000003 | 1173.16522809274 | 1000 | 0.384374368620312 |
| 100.716960000003 | 1173.39417170598 | 1000 | 0.384347625551557 |
| 100.742640000003 | 1173.62280836579 | 1000 | 0.384320779912408 |
| 100.768320000003 | 1173.85113678046 | 1000 | 0.384293831297301 |
| 100.794000000003 | 1174.07915565285 | 1000 | 0.384266779299092 |
| 100.819680000003 | 1174.30686368038 | 1000 | 0.384239623509054 |
| 100.845360000003 | 1174.53425955500 | 1000 | 0.384212363516868 |
| 100.871040000003 | 1174.76134196315 | 1000 | 0.384184998910618 |
| 100.896720000003 | 1174.98810958578 | 1000 | 0.384157529276784 |
| 100.922400000004 | 1175.21456109827 | 1000 | 0.384129954200238 |
| 100.948080000004 | 1175.44069517048 | 1000 | 0.384102273264236 |
| 100.973760000004 | 1175.66651046665 | 1000 | 0.384074486050413 |
| 100.999440000004 | 1175.89200564543 | 1000 | 0.384046592138777 |
| 101.025120000004 | 1176.11717935982 | 1000 | 0.384018591107702 |
| 101.050800000004 | 1176.34203025719 | 1000 | 0.383990482533922 |
| 101.076480000004 | 1176.56655697921 | 1000 | 0.383962265992525 |
| 101.102160000004 | 1176.79075816187 | 1000 | 0.383933941056948 |
| 101.127840000004 | 1177.01463243541 | 1000 | 0.383905507298969 |
| 101.153520000004 | 1177.23817842434 | 1000 | 0.383876964288703 |
| 101.179200000004 | 1177.46139474739 | 1000 | 0.383848311594591 |
| 101.204880000004 | 1177.68428001749 | 1000 | 0.383819548783400 |
| 101.230560000004 | 1177.90683284175 | 1000 | 0.383790675420214 |
| 101.256240000004 | 1178.12905182144 | 1000 | 0.383761691068423 |
| 101.281920000004 | 1178.35093555195 | 1000 | 0.383732595289726 |
| 101.307600000004 | 1178.57248262278 | 1000 | 0.383703387644118 |
| 101.333280000004 | 1178.79369161752 | 1000 | 0.383674067689884 |
| 101.358960000004 | 1179.01456111381 | 1000 | 0.383644634983596 |
| 101.384640000004 | 1179.23508968331 | 1000 | 0.383615089080103 |
| 101.410320000004 | 1179.45527589171 | 1000 | 0.383585429532526 |
| 101.436000000004 | 1179.67511829867 | 1000 | 0.383555655892251 |
| 101.461680000004 | 1179.89461545780 | 1000 | 0.383525767708926 |
| 101.487360000004 | 1180.11376591665 | 1000 | 0.383495764530449 |
| 101.513040000004 | 1180.33256821669 | 1000 | 0.383465645902963 |
| 101.538720000004 | 1180.55102089326 | 1000 | 0.383435411370853 |
| 101.564400000004 | 1180.76912247554 | 1000 | 0.383405060476735 |
| 101.590080000004 | 1180.98687148657 | 1000 | 0.383374592761452 |
| 101.615760000004 | 1181.20426644317 | 1000 | 0.383344007764067 |
| 101.641440000004 | 1181.42130585596 | 1000 | 0.383313305021855 |
| 101.667120000004 | 1181.63798822931 | 1000 | 0.383282484070297 |
| 101.692800000004 | 1181.85431206131 | 1000 | 0.383251544443075 |
| 101.718480000004 | 1182.07027584375 | 1000 | 0.383220485672063 |
| 101.744160000004 | 1182.28587806210 | 1000 | 0.383189307287319 |
| 101.769840000004 | 1182.50111719548 | 1000 | 0.383158008817085 |
| 101.795520000004 | 1182.71599171664 | 1000 | 0.383126589787772 |
| 101.821200000004 | 1182.93050009191 | 1000 | 0.383095049723956 |
| 101.846880000004 | 1183.14464078121 | 1000 | 0.383063388148376 |
| 101.872560000004 | 1183.35841223798 | 1000 | 0.383031604581918 |
| 101.898240000004 | 1183.57181290920 | 1000 | 0.382999698543617 |
| 101.923920000004 | 1183.78484123532 | 1000 | 0.382967669550645 |
| 101.949600000004 | 1183.99749565028 | 1000 | 0.382935517118303 |
| 101.975280000004 | 1184.20977458142 | 1000 | 0.382903240760019 |
| 102.000960000004 | 1184.42167644950 | 1000 | 0.382870839987339 |
| 102.026640000004 | 1184.63319966869 | 1000 | 0.382838314309916 |
| 102.052320000004 | 1184.84434264647 | 1000 | 0.382805663235510 |
| 102.078000000004 | 1185.05510378368 | 1000 | 0.382772886269974 |
| 102.103680000004 | 1185.26548147444 | 1000 | 0.382739982917251 |
| 102.129360000004 | 1185.47547410614 | 1000 | 0.382706952679369 |
| 102.155040000004 | 1185.68508005942 | 1000 | 0.382673795056427 |
| 102.180720000004 | 1185.89429770814 | 1000 | 0.382640509546593 |
| 102.206400000004 | 1186.10312541933 | 1000 | 0.382607095646097 |
| 102.232080000004 | 1186.31156155320 | 1000 | 0.382573552849221 |
| 102.257760000004 | 1186.51960446306 | 1000 | 0.382539880648293 |
| 102.283440000004 | 1186.72725249535 | 1000 | 0.382506078533682 |
| 102.309120000004 | 1186.93450398958 | 1000 | 0.382472145993786 |
| 102.334800000004 | 1187.14135727828 | 1000 | 0.382438082515029 |
| 102.360480000004 | 1187.34781068703 | 1000 | 0.382403887581851 |
| 102.386160000004 | 1187.55386253436 | 1000 | 0.382369560676704 |
| 102.411840000004 | 1187.75951113179 | 1000 | 0.382335101280039 |
| 102.437520000004 | 1187.96475478374 | 1000 | 0.382300508870305 |
| 102.463200000004 | 1188.16959178757 | 1000 | 0.382265782923936 |
| 102.488880000004 | 1188.37402043345 | 1000 | 0.382230922915349 |
| 102.514560000004 | 1188.57803900445 | 1000 | 0.382195928316931 |
| 102.540240000004 | 1188.78164577640 | 1000 | 0.382160798599035 |
| 102.565920000004 | 1188.98483901796 | 1000 | 0.382125533229972 |
| 102.591600000004 | 1189.18761699050 | 1000 | 0.382090131676002 |
| 102.617280000004 | 1189.38997794813 | 1000 | 0.382054593401330 |
| 102.642960000004 | 1189.59192013765 | 1000 | 0.382018917868092 |
| 102.668640000004 | 1189.79344179851 | 1000 | 0.381983104536356 |
| 102.694320000004 | 1189.99454116280 | 1000 | 0.381947152864105 |
| 102.720000000004 | 1190.19521645521 | 1000 | 0.381911062307238 |
| 102.745680000004 | 1190.39546589300 | 1000 | 0.381874832319554 |
| 102.771360000004 | 1190.59528768596 | 1000 | 0.381838462352753 |
| 102.797040000004 | 1190.79468003639 | 1000 | 0.381801951856421 |
| 102.822720000004 | 1190.99364113907 | 1000 | 0.381765300278027 |
| 102.848400000004 | 1191.19216918123 | 1000 | 0.381728507062909 |
| 102.874080000004 | 1191.39026234251 | 1000 | 0.381691571654277 |
| 102.899760000004 | 1191.58791879491 | 1000 | 0.381654493493192 |
| 102.925440000004 | 1191.78513670283 | 1000 | 0.381617272018570 |
| 102.951120000004 | 1191.98191422294 | 1000 | 0.381579906667166 |
| 102.976800000004 | 1192.17824950424 | 1000 | 0.381542396873568 |
| 103.002480000004 | 1192.37414068795 | 1000 | 0.381504742070192 |
| 103.028160000004 | 1192.56958590753 | 1000 | 0.381466941687272 |
| 103.053840000004 | 1192.76458328864 | 1000 | 0.381428995152850 |
| 103.079520000004 | 1192.95913094909 | 1000 | 0.381390901892771 |
| 103.105200000004 | 1193.15322699881 | 1000 | 0.381352661330674 |
| 103.130880000004 | 1193.34686953984 | 1000 | 0.381314272887984 |
| 103.156560000004 | 1193.54005666628 | 1000 | 0.381275735983902 |
| 103.182240000004 | 1193.73278646425 | 1000 | 0.381237050035401 |
| 103.207920000004 | 1193.92505701187 | 1000 | 0.381198214457213 |
| 103.233600000004 | 1194.11686637923 | 1000 | 0.381159228661823 |
| 103.259280000004 | 1194.30821262833 | 1000 | 0.381120092059464 |
| 103.284960000004 | 1194.49909381310 | 1000 | 0.381080804058102 |
| 103.310640000004 | 1194.68950797930 | 1000 | 0.381041364063434 |
| 103.336320000004 | 1194.87945316455 | 1000 | 0.381001771478875 |
| 103.362000000004 | 1195.06892739823 | 1000 | 0.380962025705553 |
| 103.387680000004 | 1195.25792870152 | 1000 | 0.380922126142300 |
| 103.413360000004 | 1195.44645508731 | 1000 | 0.380882072185641 |
| 103.439040000004 | 1195.63450456017 | 1000 | 0.380841863229789 |
| 103.464720000004 | 1195.82207511636 | 1000 | 0.380801498666635 |
| 103.490400000004 | 1196.00916474375 | 1000 | 0.380760977885741 |
| 103.516080000004 | 1196.19577142179 | 1000 | 0.380720300274326 |
| 103.541760000004 | 1196.38189312151 | 1000 | 0.380679465217267 |
| 103.567440000004 | 1196.56752780544 | 1000 | 0.380638472097082 |
| 103.593120000004 | 1196.75267342760 | 1000 | 0.380597320293925 |
| 103.618800000004 | 1196.93732793348 | 1000 | 0.380556009185577 |
| 103.644480000004 | 1197.12148925996 | 1000 | 0.380514538147438 |
| 103.670160000004 | 1197.30515533533 | 1000 | 0.380472906552516 |
| 103.695840000004 | 1197.48832407918 | 1000 | 0.380431113771423 |
| 103.721520000004 | 1197.67099340247 | 1000 | 0.380389159172361 |
| 103.747200000004 | 1197.85316120737 | 1000 | 0.380347042121116 |
| 103.772880000004 | 1198.03482538734 | 1000 | 0.380304761981048 |
| 103.798560000004 | 1198.21598382701 | 1000 | 0.380262318113085 |
| 103.824240000004 | 1198.39663440219 | 1000 | 0.380219709875710 |
| 103.849920000004 | 1198.57677497982 | 1000 | 0.380176936624955 |
| 103.875600000004 | 1198.75640341791 | 1000 | 0.380133997714394 |
| 103.901280000004 | 1198.93551756555 | 1000 | 0.380090892495126 |
| 103.926960000004 | 1199.11411526285 | 1000 | 0.380047620315777 |
| 103.952640000004 | 1199.29219434088 | 1000 | 0.380004180522483 |
| 103.978320000004 | 1199.46975262167 | 1000 | 0.379960572458883 |
| 104.004000000004 | 1199.64678791816 | 1000 | 0.379916795466113 |
| 104.029680000004 | 1199.82329803415 | 1000 | 0.379872848882792 |
| 104.055360000004 | 1199.99928076428 | 1000 | 0.379828732045017 |
| 104.081040000004 | 1200.17473389398 | 1000 | 0.379784444286352 |
| 104.106720000004 | 1200.34965519945 | 1000 | 0.379739984937819 |
| 104.132400000004 | 1200.52404244758 | 1000 | 0.379695353327890 |
| 104.158080000004 | 1200.69789339596 | 1000 | 0.379650548782474 |
| 104.183760000005 | 1200.87120579283 | 1000 | 0.379605570624914 |
| 104.209440000005 | 1201.04397737702 | 1000 | 0.379560418175973 |
| 104.235120000005 | 1201.21620587792 | 1000 | 0.379515090753826 |
| 104.260800000005 | 1201.38788901547 | 1000 | 0.379469587674051 |
| 104.286480000005 | 1201.55902450007 | 1000 | 0.379423908249619 |
| 104.312160000005 | 1201.72961003259 | 1000 | 0.379378051790885 |
| 104.337840000005 | 1201.89964330429 | 1000 | 0.379332017605580 |
| 104.363520000005 | 1202.06912199682 | 1000 | 0.379285804998798 |
| 104.389200000005 | 1202.23804378214 | 1000 | 0.379239413272991 |
| 104.414880000005 | 1202.40640632250 | 1000 | 0.379192841727956 |
| 104.440560000005 | 1202.57420727043 | 1000 | 0.379146089660827 |
| 104.466240000005 | 1202.74144426864 | 1000 | 0.379099156366065 |
| 104.491920000005 | 1202.90811495001 | 1000 | 0.379052041135450 |
| 104.517600000005 | 1203.07421693757 | 1000 | 0.379004743258068 |
| 104.543280000005 | 1203.23974784442 | 1000 | 0.378957262020305 |
| 104.568960000005 | 1203.40470527372 | 1000 | 0.378909596705834 |
| 104.594640000005 | 1203.56908681864 | 1000 | 0.378861746595609 |
| 104.620320000005 | 1203.73289006229 | 1000 | 0.378813710967853 |
| 104.646000000005 | 1203.89611257775 | 1000 | 0.378765489098047 |
| 104.671680000005 | 1204.05875192793 | 1000 | 0.378717080258924 |
| 104.697360000005 | 1204.22080566564 | 1000 | 0.378668483720454 |
| 104.723040000005 | 1204.38227133344 | 1000 | 0.378619698749841 |
| 104.748720000005 | 1204.54314646367 | 1000 | 0.378570724611506 |
| 104.774400000005 | 1204.70342857840 | 1000 | 0.378521560567081 |
| 104.800080000005 | 1204.86311518936 | 1000 | 0.378472205875400 |
| 104.825760000005 | 1205.02220379790 | 1000 | 0.378422659792486 |
| 104.851440000005 | 1205.18069189498 | 1000 | 0.378372921571542 |
| 104.877120000005 | 1205.33857696111 | 1000 | 0.378322990462943 |
| 104.902800000005 | 1205.49585646628 | 1000 | 0.378272865714223 |
| 104.928480000005 | 1205.65252786995 | 1000 | 0.378222546570068 |
| 104.954160000005 | 1205.80858862102 | 1000 | 0.378172032272301 |
| 104.979840000005 | 1205.96403615774 | 1000 | 0.378121322059878 |
| 105.005520000005 | 1206.11886790769 | 1000 | 0.378070415168874 |
| 105.031200000005 | 1206.27308128775 | 1000 | 0.378019310832473 |
| 105.056880000005 | 1206.42667370405 | 1000 | 0.377968008280958 |
| 105.082560000005 | 1206.57964255188 | 1000 | 0.377916506741703 |
| 105.108240000005 | 1206.73198521574 | 1000 | 0.377864805439159 |
| 105.133920000005 | 1206.88369906919 | 1000 | 0.377812903594847 |
| 105.159600000005 | 1207.03478147489 | 1000 | 0.377760800427344 |
| 105.185280000005 | 1207.18522978451 | 1000 | 0.377708495152276 |
| 105.210960000005 | 1207.33504133869 | 1000 | 0.377655986982306 |
| 105.236640000005 | 1207.48421346701 | 1000 | 0.377603275127124 |
| 105.262320000005 | 1207.63274348792 | 1000 | 0.377550358793437 |
| 105.288000000005 | 1207.78062870874 | 1000 | 0.377497237184957 |
| 105.313680000005 | 1207.92786642555 | 1000 | 0.377443909502391 |
| 105.339360000005 | 1208.07445392320 | 1000 | 0.377390374943430 |
| 105.365040000005 | 1208.22038847524 | 1000 | 0.377336632702741 |
| 105.390720000005 | 1208.36566734388 | 1000 | 0.377282681971954 |
| 105.416400000005 | 1208.51028777991 | 1000 | 0.377228521939651 |
| 105.442080000005 | 1208.65424702272 | 1000 | 0.377174151791355 |
| 105.467760000005 | 1208.79754230021 | 1000 | 0.377119570709523 |
| 105.493440000005 | 1208.94017082873 | 1000 | 0.377064777873530 |
| 105.519120000005 | 1209.08212981308 | 1000 | 0.377009772459662 |
| 105.544800000005 | 1209.22341644640 | 1000 | 0.376954553641105 |
| 105.570480000005 | 1209.36402791018 | 1000 | 0.376899120587929 |
| 105.596160000005 | 1209.50396137421 | 1000 | 0.376843472467086 |
| 105.621840000005 | 1209.64321399646 | 1000 | 0.376787608442391 |
| 105.647520000005 | 1209.78178292312 | 1000 | 0.376731527674515 |
| 105.673200000005 | 1209.91966528852 | 1000 | 0.376675229320974 |
| 105.698880000005 | 1210.05685821506 | 1000 | 0.376618712536116 |
| 105.724560000005 | 1210.19335881317 | 1000 | 0.376561976471113 |
| 105.750240000005 | 1210.32916418130 | 1000 | 0.376505020273946 |
| 105.775920000005 | 1210.46427140581 | 1000 | 0.376447843089396 |
| 105.801600000005 | 1210.59867756098 | 1000 | 0.376390444059035 |
| 105.827280000005 | 1210.73237970890 | 1000 | 0.376332822321211 |
| 105.852960000005 | 1210.86537489948 | 1000 | 0.376274977011037 |
| 105.878640000005 | 1210.99766017036 | 1000 | 0.376216907260385 |
| 105.904320000005 | 1211.12923254686 | 1000 | 0.376158612197868 |
| 105.930000000005 | 1211.26008904197 | 1000 | 0.376100090948830 |
| 105.955680000005 | 1211.39022665625 | 1000 | 0.376041342635342 |
| 105.981360000005 | 1211.51964237781 | 1000 | 0.375982366376178 |
| 106.007040000005 | 1211.64833318224 | 1000 | 0.375923161286815 |
| 106.032720000005 | 1211.77629603259 | 1000 | 0.375863726479416 |
| 106.058400000005 | 1211.90352787928 | 1000 | 0.375804061062818 |
| 106.084080000005 | 1212.03002566007 | 1000 | 0.375744164142524 |
| 106.109760000005 | 1212.15578630001 | 1000 | 0.375684034820689 |
| 106.135440000005 | 1212.28080671137 | 1000 | 0.375623672196106 |
| 106.161120000005 | 1212.40508379362 | 1000 | 0.375563075364202 |
| 106.186800000005 | 1212.52861443335 | 1000 | 0.375502243417020 |
| 106.212480000005 | 1212.65139550422 | 1000 | 0.375441175443206 |
| 106.238160000005 | 1212.77342386692 | 1000 | 0.375379870528003 |
| 106.263840000005 | 1212.89469636912 | 1000 | 0.375318327753238 |
| 106.289520000005 | 1213.01520984539 | 1000 | 0.375256546197305 |
| 106.315200000005 | 1213.13496111717 | 1000 | 0.375194524935160 |
| 106.340880000005 | 1213.25394699272 | 1000 | 0.375132263038305 |
| 106.366560000005 | 1213.37216426705 | 1000 | 0.375069759574777 |
| 106.392240000005 | 1213.48960972188 | 1000 | 0.375007013609137 |
| 106.417920000005 | 1213.60628012557 | 1000 | 0.374944024202458 |
| 106.443600000005 | 1213.72217223308 | 1000 | 0.374880790412309 |
| 106.469280000005 | 1213.83728278590 | 1000 | 0.374817311292752 |
| 106.494960000005 | 1213.95160851203 | 1000 | 0.374753585894320 |
| 106.520640000005 | 1214.06514612588 | 1000 | 0.374689613264012 |
| 106.546320000005 | 1214.17789232825 | 1000 | 0.374625392445277 |
| 106.572000000005 | 1214.28984380624 | 1000 | 0.374560922478003 |
| 106.597680000005 | 1214.40099723323 | 1000 | 0.374496202398507 |
| 106.623360000005 | 1214.51134926882 | 1000 | 0.374431231239518 |
| 106.649040000005 | 1214.62089655873 | 1000 | 0.374366008030169 |
| 106.674720000005 | 1214.72963573480 | 1000 | 0.374300531795984 |
| 106.700400000005 | 1214.83756341491 | 1000 | 0.374234801558865 |
| 106.726080000005 | 1214.94467620292 | 1000 | 0.374168816337078 |
| 106.751760000005 | 1215.05097068861 | 1000 | 0.374102575145244 |
| 106.777440000005 | 1215.15644344764 | 1000 | 0.374036076994325 |
| 106.803120000005 | 1215.26109104147 | 1000 | 0.373969320891610 |
| 106.828800000005 | 1215.36491001732 | 1000 | 0.373902305840706 |
| 106.854480000005 | 1215.46789690811 | 1000 | 0.373835030841523 |
| 106.880160000005 | 1215.57004823239 | 1000 | 0.373767494890261 |
| 106.905840000005 | 1215.67136049430 | 1000 | 0.373699696979400 |
| 106.931520000005 | 1215.77183018350 | 1000 | 0.373631636097686 |
| 106.957200000005 | 1215.87145377510 | 1000 | 0.373563311230117 |
| 106.982880000005 | 1215.97022772963 | 1000 | 0.373494721357932 |
| 107.008560000005 | 1216.06814849295 | 1000 | 0.373425865458599 |
| 107.034240000005 | 1216.16521249623 | 1000 | 0.373356742505801 |
| 107.059920000005 | 1216.26141615584 | 1000 | 0.373287351469422 |
| 107.085600000005 | 1216.35675587332 | 1000 | 0.373217691315538 |
| 107.111280000005 | 1216.45122803533 | 1000 | 0.373147761006400 |
| 107.136960000005 | 1216.54482901356 | 1000 | 0.373077559500425 |
| 107.162640000005 | 1216.63755516470 | 1000 | 0.373007085752179 |
| 107.188320000005 | 1216.72940283034 | 1000 | 0.372936338712368 |
| 107.214000000005 | 1216.82036833696 | 1000 | 0.372865317327821 |
| 107.239680000005 | 1216.91044799582 | 1000 | 0.372794020541483 |
| 107.265360000005 | 1216.99963810293 | 1000 | 0.372722447292395 |
| 107.291040000005 | 1217.08793493899 | 1000 | 0.372650596515685 |
| 107.316720000005 | 1217.17533476930 | 1000 | 0.372578467142555 |
| 107.342400000005 | 1217.26183384370 | 1000 | 0.372506058100267 |
| 107.368080000005 | 1217.34742839657 | 1000 | 0.372433368312129 |
| 107.393760000005 | 1217.43211464666 | 1000 | 0.372360396697483 |
| 107.419440000005 | 1217.51588879713 | 1000 | 0.372287142171692 |
| 107.445120000005 | 1217.59874703542 | 1000 | 0.372213603646126 |
| 107.470800000006 | 1217.68068553323 | 1000 | 0.372139780028148 |
| 107.496480000006 | 1217.76170044640 | 1000 | 0.372065670221103 |
| 107.522160000006 | 1217.84178791492 | 1000 | 0.371991273124302 |
| 107.547840000006 | 1217.92094406280 | 1000 | 0.371916587633012 |
| 107.573520000006 | 1217.99916499805 | 1000 | 0.371841612638437 |
| 107.599200000006 | 1218.07644681258 | 1000 | 0.371766347027712 |
| 107.624880000006 | 1218.15278558219 | 1000 | 0.371690789683883 |
| 107.650560000006 | 1218.22817736642 | 1000 | 0.371614939485896 |
| 107.676240000006 | 1218.30261820856 | 1000 | 0.371538795308585 |
| 107.701920000006 | 1218.37610413555 | 1000 | 0.371462356022655 |
| 107.727600000006 | 1218.44863115794 | 1000 | 0.371385620494673 |
| 107.753280000006 | 1218.52019526977 | 1000 | 0.371308587587048 |
| 107.778960000006 | 1218.59079244856 | 1000 | 0.371231256158024 |
| 107.804640000006 | 1218.66041865522 | 1000 | 0.371153625061662 |
| 107.830320000006 | 1218.72906983398 | 1000 | 0.371075693147828 |
| 107.856000000006 | 1218.79674191234 | 1000 | 0.370997459262178 |
| 107.881680000006 | 1218.86343080097 | 1000 | 0.370918922246145 |
| 107.907360000006 | 1218.92913239367 | 1000 | 0.370840080936925 |
| 107.933040000006 | 1218.99384256729 | 1000 | 0.370760934167464 |
| 107.958720000006 | 1219.05755718169 | 1000 | 0.370681480766443 |
| 107.984400000006 | 1219.12027207962 | 1000 | 0.370601719558264 |
| 108.010080000006 | 1219.18198308668 | 1000 | 0.370521649363037 |
| 108.035760000006 | 1219.24268601127 | 1000 | 0.370441268996564 |
| 108.061440000006 | 1219.30237664448 | 1000 | 0.370360577270326 |
| 108.087120000006 | 1219.36105076005 | 1000 | 0.370279572991473 |
| 108.112800000006 | 1219.41870411428 | 1000 | 0.370198254962801 |
| 108.138480000006 | 1219.47533244598 | 1000 | 0.370116621982747 |
| 108.164160000006 | 1219.53093147640 | 1000 | 0.370034672845369 |
| 108.189840000006 | 1219.58549690913 | 1000 | 0.369952406340334 |
| 108.215520000006 | 1219.63902443005 | 1000 | 0.369869821252902 |
| 108.241200000006 | 1219.69150970728 | 1000 | 0.369786916363916 |
| 108.266880000006 | 1219.74294839106 | 1000 | 0.369703690449782 |
| 108.292560000006 | 1219.79333611371 | 1000 | 0.369620142282459 |
| 108.318240000006 | 1219.84266848956 | 1000 | 0.369536270629442 |
| 108.343920000006 | 1219.89094111487 | 1000 | 0.369452074253751 |
| 108.369600000006 | 1219.93814956775 | 1000 | 0.369367551913912 |
| 108.395280000006 | 1219.98428940809 | 1000 | 0.369282702363945 |
| 108.420960000006 | 1220.02935617751 | 1000 | 0.369197524353351 |
| 108.446640000006 | 1220.07334539925 | 1000 | 0.369112016627093 |
| 108.472320000006 | 1220.11625257811 | 1000 | 0.369026177925587 |
| 108.498000000006 | 1220.15807320041 | 1000 | 0.368940006984683 |
| 108.523680000006 | 1220.19880273386 | 1000 | 0.368853502535653 |
| 108.549360000006 | 1220.23843662751 | 1000 | 0.368766663305175 |
| 108.575040000006 | 1220.27697031169 | 1000 | 0.368679488015317 |
| 108.600720000006 | 1220.31439919792 | 1000 | 0.368591975383528 |
| 108.626400000006 | 1220.35071867882 | 1000 | 0.368504124122615 |
| 108.652080000006 | 1220.38592412807 | 1000 | 0.368415932940735 |
| 108.677760000006 | 1220.42001090030 | 1000 | 0.368327400541377 |
| 108.703440000006 | 1220.45297433103 | 1000 | 0.368238525623346 |
| 108.729120000006 | 1220.48480973662 | 1000 | 0.368149306880753 |
| 108.754800000006 | 1220.51551241411 | 1000 | 0.368059743002995 |
| 108.780480000006 | 1220.54507764125 | 1000 | 0.367969832674743 |
| 108.806160000006 | 1220.57350067634 | 1000 | 0.367879574575925 |
| 108.831840000006 | 1220.60077675819 | 1000 | 0.367788967381713 |
| 108.857520000006 | 1220.62690110604 | 1000 | 0.367698009762507 |
| 108.883200000006 | 1220.65186891947 | 1000 | 0.367606700383919 |
| 108.908880000006 | 1220.67567537833 | 1000 | 0.367515037906760 |
| 108.934560000006 | 1220.69831564265 | 1000 | 0.367423020987024 |
| 108.960240000006 | 1220.71978485259 | 1000 | 0.367330648275872 |
| 108.985920000006 | 1220.74007812832 | 1000 | 0.367237918419616 |
| 109.011600000006 | 1220.75919056998 | 1000 | 0.367144830059709 |
| 109.037280000006 | 1220.77711725755 | 1000 | 0.367051381832721 |
| 109.062960000006 | 1220.79385325083 | 1000 | 0.366957572370334 |
| 109.088640000006 | 1220.80939358931 | 1000 | 0.366863400299317 |
| 109.114320000006 | 1220.82373329213 | 1000 | 0.366768864241518 |
| 109.140000000006 | 1220.83686735794 | 1000 | 0.366673962813843 |
| 109.165680000006 | 1220.84879076490 | 1000 | 0.366578694628246 |
| 109.191360000006 | 1220.85949847051 | 1000 | 0.366483058291709 |
| 109.217040000006 | 1220.86898541161 | 1000 | 0.366387052406230 |
| 109.242720000006 | 1220.87724650423 | 1000 | 0.366290675568804 |
| 109.268400000006 | 1220.88427664355 | 1000 | 0.366193926371410 |
| 109.294080000006 | 1220.89007070380 | 1000 | 0.366096803400995 |
| 109.319760000006 | 1220.89462353817 | 1000 | 0.365999305239460 |
| 109.345440000006 | 1220.89792997875 | 1000 | 0.365901430463639 |
| 109.371120000006 | 1220.89998483642 | 1000 | 0.365803177645291 |
| 109.396800000006 | 1220.90078290078 | 1000 | 0.365704545351077 |
| 109.422480000006 | 1220.90031894007 | 1000 | 0.365605532142549 |
| 109.448160000006 | 1220.89858770107 | 1000 | 0.365506136576135 |
| 109.473840000006 | 1220.89558390902 | 1000 | 0.365406357203117 |
| 109.499520000006 | 1220.89130226753 | 1000 | 0.365306192569623 |
| 109.525200000006 | 1220.88573745852 | 1000 | 0.365205641216605 |
| 109.550880000006 | 1220.87888414209 | 1000 | 0.365104701679828 |
| 109.576560000006 | 1220.87073695649 | 1000 | 0.365003372489851 |
| 109.602240000006 | 1220.86129051795 | 1000 | 0.364901652172012 |
| 109.627920000006 | 1220.85053942069 | 1000 | 0.364799539246410 |
| 109.653600000006 | 1220.83847823677 | 1000 | 0.364697032227895 |
| 109.679280000006 | 1220.82510151600 | 1000 | 0.364594129626046 |
| 109.704960000006 | 1220.81040378590 | 1000 | 0.364490829945156 |
| 109.730640000006 | 1220.79437955155 | 1000 | 0.364387131684221 |
| 109.756320000006 | 1220.77702329555 | 1000 | 0.364283033336915 |
| 109.782000000006 | 1220.75832947790 | 1000 | 0.364178533391583 |
| 109.807680000006 | 1220.73829253595 | 1000 | 0.364073630331218 |
| 109.833360000006 | 1220.71690688425 | 1000 | 0.363968322633450 |
| 109.859040000006 | 1220.69416691450 | 1000 | 0.363862608770526 |
| 109.884720000006 | 1220.67006699546 | 1000 | 0.363756487209296 |
| 109.910400000006 | 1220.64460147286 | 1000 | 0.363649956411196 |
| 109.936080000006 | 1220.61776466928 | 1000 | 0.363543014832232 |
| 109.961760000006 | 1220.58955088408 | 1000 | 0.363435660922962 |
| 109.987440000006 | 1220.55995439332 | 1000 | 0.363327893128484 |
| 110.013120000006 | 1220.52896944964 | 1000 | 0.363219709888415 |
| 110.038800000006 | 1220.49659028219 | 1000 | 0.363111109636877 |
| 110.064480000006 | 1220.46281109653 | 1000 | 0.363002090802481 |
| 110.090160000006 | 1220.42762607453 | 1000 | 0.362892651808308 |
| 110.115840000006 | 1220.39102937428 | 1000 | 0.362782791071896 |
| 110.141520000006 | 1220.35301512999 | 1000 | 0.362672507005221 |
| 110.167200000006 | 1220.31357745193 | 1000 | 0.362561798014682 |
| 110.192880000006 | 1220.27271042628 | 1000 | 0.362450662501083 |
| 110.218560000006 | 1220.23040811507 | 1000 | 0.362339098859619 |
| 110.244240000006 | 1220.18666455610 | 1000 | 0.362227105479857 |
| 110.269920000006 | 1220.14147376278 | 1000 | 0.362114680745719 |
| 110.295600000006 | 1220.09482972412 | 1000 | 0.362001823035468 |
| 110.321280000006 | 1220.04672640457 | 1000 | 0.361888530721689 |
| 110.346960000006 | 1219.99715774392 | 1000 | 0.361774802171275 |
| 110.372640000006 | 1219.94611765728 | 1000 | 0.361660635745407 |
| 110.398320000006 | 1219.89360003487 | 1000 | 0.361546029799540 |
| 110.424000000006 | 1219.83959874202 | 1000 | 0.361430982683385 |
| 110.449680000006 | 1219.78410761902 | 1000 | 0.361315492740891 |
| 110.475360000006 | 1219.72712048102 | 1000 | 0.361199558310232 |
| 110.501040000006 | 1219.66863111798 | 1000 | 0.361083177723787 |
| 110.526720000006 | 1219.60863329450 | 1000 | 0.360966349308123 |
| 110.552400000006 | 1219.54712074976 | 1000 | 0.360849071383981 |
| 110.578080000006 | 1219.48408719743 | 1000 | 0.360731342266256 |
| 110.603760000006 | 1219.41952632556 | 1000 | 0.360613160263982 |
| 110.629440000006 | 1219.35343179645 | 1000 | 0.360494523680316 |
| 110.655120000006 | 1219.28579724659 | 1000 | 0.360375430812516 |
| 110.680800000006 | 1219.21661628654 | 1000 | 0.360255879951931 |
| 110.706480000007 | 1219.14588250083 | 1000 | 0.360135869383980 |
| 110.732160000007 | 1219.07358944786 | 1000 | 0.360015397388135 |
| 110.757840000007 | 1218.99973065978 | 1000 | 0.359894462237906 |
| 110.783520000007 | 1218.92429964242 | 1000 | 0.359773062200821 |
| 110.809200000007 | 1218.84728987516 | 1000 | 0.359651195538411 |
| 110.834880000007 | 1218.76869481084 | 1000 | 0.359528860506194 |
| 110.860560000007 | 1218.68850787564 | 1000 | 0.359406055353655 |
| 110.886240000007 | 1218.60672246901 | 1000 | 0.359282778324231 |
| 110.911920000007 | 1218.52333196352 | 1000 | 0.359159027655293 |
| 110.937600000007 | 1218.43832970477 | 1000 | 0.359034801578130 |
| 110.963280000007 | 1218.35170901132 | 1000 | 0.358910098317928 |
| 110.988960000007 | 1218.26346317453 | 1000 | 0.358784916093759 |
| 111.014640000007 | 1218.17358545849 | 1000 | 0.358659253118558 |
| 111.040320000007 | 1218.08206909990 | 1000 | 0.358533107599111 |
| 111.066000000007 | 1217.98890730795 | 1000 | 0.358406477736033 |
| 111.091680000007 | 1217.89409326426 | 1000 | 0.358279361723752 |
| 111.117360000007 | 1217.79762012270 | 1000 | 0.358151757750494 |
| 111.143040000007 | 1217.69948100934 | 1000 | 0.358023663998264 |
| 111.168720000007 | 1217.59966902233 | 1000 | 0.357895078642829 |
| 111.194400000007 | 1217.49817723176 | 1000 | 0.357765999853699 |
| 111.220080000007 | 1217.39499867961 | 1000 | 0.357636425794113 |
| 111.245760000007 | 1217.29012637956 | 1000 | 0.357506354621019 |
| 111.271440000007 | 1217.18355331695 | 1000 | 0.357375784485057 |
| 111.297120000007 | 1217.07527244864 | 1000 | 0.357244713530542 |
| 111.322800000007 | 1216.96527670289 | 1000 | 0.357113139895447 |
| 111.348480000007 | 1216.85355897927 | 1000 | 0.356981061711386 |
| 111.374160000007 | 1216.74011214854 | 1000 | 0.356848477103595 |
| 111.399840000007 | 1216.62492905254 | 1000 | 0.356715384190913 |
| 111.425520000007 | 1216.50800250404 | 1000 | 0.356581781085771 |
| 111.451200000007 | 1216.38932528671 | 1000 | 0.356447665894168 |
| 111.476880000007 | 1216.26889015492 | 1000 | 0.356313036715654 |
| 111.502560000007 | 1216.14668983367 | 1000 | 0.356177891643318 |
| 111.528240000007 | 1216.02271701849 | 1000 | 0.356042228763765 |
| 111.553920000007 | 1215.89696437527 | 1000 | 0.355906046157099 |
| 111.579600000007 | 1215.76942454021 | 1000 | 0.355769341896908 |
| 111.605280000007 | 1215.64009011965 | 1000 | 0.355632114050245 |
| 111.630960000007 | 1215.50895368999 | 1000 | 0.355494360677611 |
| 111.656640000007 | 1215.37600779756 | 1000 | 0.355356079832935 |
| 111.682320000007 | 1215.24124495850 | 1000 | 0.355217269563561 |
| 111.708000000007 | 1215.10465765865 | 1000 | 0.355077927910225 |
| 111.733680000007 | 1214.96623835343 | 1000 | 0.354938052907043 |
| 111.759360000007 | 1214.82597946771 | 1000 | 0.354797642581486 |
| 111.785040000007 | 1214.68387339574 | 1000 | 0.354656694954372 |
| 111.810720000007 | 1214.53991250094 | 1000 | 0.354515208039840 |
| 111.836400000007 | 1214.39408911588 | 1000 | 0.354373179845336 |
| 111.862080000007 | 1214.24639554210 | 1000 | 0.354230608371595 |
| 111.887760000007 | 1214.09682405001 | 1000 | 0.354087491612623 |
| 111.913440000007 | 1213.94536687874 | 1000 | 0.353943827555679 |
| 111.939120000007 | 1213.79201623608 | 1000 | 0.353799614181260 |
| 111.964800000007 | 1213.63676429831 | 1000 | 0.353654849463077 |
| 111.990480000007 | 1213.47960321008 | 1000 | 0.353509531368045 |
| 112.016160000007 | 1213.32052508431 | 1000 | 0.353363657856259 |
| 112.041840000007 | 1213.15952200205 | 1000 | 0.353217226880981 |
| 112.067520000007 | 1212.99658601238 | 1000 | 0.353070236388619 |
| 112.093200000007 | 1212.83170913224 | 1000 | 0.352922684318711 |
| 112.118880000007 | 1212.66488334637 | 1000 | 0.352774568603905 |
| 112.144560000007 | 1212.49610060712 | 1000 | 0.352625887169944 |
| 112.170240000007 | 1212.32535283438 | 1000 | 0.352476637935648 |
| 112.195920000007 | 1212.15263191542 | 1000 | 0.352326818812895 |
| 112.221600000007 | 1211.97792970478 | 1000 | 0.352176427706601 |
| 112.247280000007 | 1211.80123802413 | 1000 | 0.352025462514709 |
| 112.272960000007 | 1211.62254866217 | 1000 | 0.351873921128164 |
| 112.298640000007 | 1211.44185337448 | 1000 | 0.351721801430900 |
| 112.324320000007 | 1211.25914388339 | 1000 | 0.351569101299819 |
| 112.350000000007 | 1211.07441187787 | 1000 | 0.351415818604777 |
| 112.375680000007 | 1210.88764901339 | 1000 | 0.351261951208561 |
| 112.401360000007 | 1210.69884691180 | 1000 | 0.351107496966876 |
| 112.427040000007 | 1210.50799716120 | 1000 | 0.350952453728325 |
| 112.452720000007 | 1210.31509131577 | 1000 | 0.350796819334392 |
| 112.478400000007 | 1210.12012089572 | 1000 | 0.350640591619424 |
| 112.504080000007 | 1209.92307738709 | 1000 | 0.350483768410612 |
| 112.529760000007 | 1209.72395224166 | 1000 | 0.350326347527974 |
| 112.555440000007 | 1209.52273687677 | 1000 | 0.350168326784339 |
| 112.581120000007 | 1209.31942267526 | 1000 | 0.350009703985327 |
| 112.606800000007 | 1209.11400098528 | 1000 | 0.349850476929331 |
| 112.632480000007 | 1208.90646312017 | 1000 | 0.349690643407501 |
| 112.658160000007 | 1208.69680035834 | 1000 | 0.349530201203726 |
| 112.683840000007 | 1208.48500394313 | 1000 | 0.349369148094614 |
| 112.709520000007 | 1208.27106508267 | 1000 | 0.349207481849477 |
| 112.735200000007 | 1208.05497494975 | 1000 | 0.349045200230311 |
| 112.760880000007 | 1207.83672468169 | 1000 | 0.348882300991780 |
| 112.786560000007 | 1207.61630538019 | 1000 | 0.348718781881199 |
| 112.812240000007 | 1207.39370811122 | 1000 | 0.348554640638511 |
| 112.837920000007 | 1207.16892390485 | 1000 | 0.348389874996276 |
| 112.863600000007 | 1206.94194375513 | 1000 | 0.348224482679650 |
| 112.889280000007 | 1206.71275861997 | 1000 | 0.348058461406367 |
| 112.914960000007 | 1206.48135942096 | 1000 | 0.347891808886721 |
| 112.940640000007 | 1206.24773704328 | 1000 | 0.347724522823551 |
| 112.966320000007 | 1206.01188233553 | 1000 | 0.347556600912220 |
| 112.992000000007 | 1205.77378610958 | 1000 | 0.347388040840599 |
| 113.017680000007 | 1205.53343914048 | 1000 | 0.347218840289049 |
| 113.043360000007 | 1205.29083216627 | 1000 | 0.347048996930404 |
| 113.069040000007 | 1205.04595588787 | 1000 | 0.346878508429953 |
| 113.094720000007 | 1204.79880096889 | 1000 | 0.346707372445419 |
| 113.120400000007 | 1204.54935803558 | 1000 | 0.346535586626949 |
| 113.146080000007 | 1204.29761767660 | 1000 | 0.346363148617087 |
| 113.171760000007 | 1204.04357044291 | 1000 | 0.346190056050765 |
| 113.197440000007 | 1203.78720684765 | 1000 | 0.346016306555280 |
| 113.223120000007 | 1203.52851736594 | 1000 | 0.345841897750277 |
| 113.248800000007 | 1203.26749243479 | 1000 | 0.345666827247733 |
| 113.274480000007 | 1203.00412245295 | 1000 | 0.345491092651940 |
| 113.300160000007 | 1202.73839778073 | 1000 | 0.345314691559484 |
| 113.325840000007 | 1202.47030873987 | 1000 | 0.345137621559231 |
| 113.351520000007 | 1202.19984561341 | 1000 | 0.344959880232309 |
| 113.377200000007 | 1201.92699864554 | 1000 | 0.344781465152087 |
| 113.402880000007 | 1201.65175804144 | 1000 | 0.344602373884162 |
| 113.428560000007 | 1201.37411396713 | 1000 | 0.344422603986339 |
| 113.454240000007 | 1201.09405654933 | 1000 | 0.344242153008616 |
| 113.479920000007 | 1200.81157587534 | 1000 | 0.344061018493161 |
| 113.505600000007 | 1200.52666199282 | 1000 | 0.343879197974302 |
| 113.531280000007 | 1200.23930490972 | 1000 | 0.343696688978505 |
| 113.556960000007 | 1199.94949459408 | 1000 | 0.343513489024356 |
| 113.582640000007 | 1199.65722097389 | 1000 | 0.343329595622546 |
| 113.608320000007 | 1199.36247393695 | 1000 | 0.343145006275855 |
| 113.634000000007 | 1199.06524333072 | 1000 | 0.342959718479129 |
| 113.659680000007 | 1198.76551896213 | 1000 | 0.342773729719268 |
| 113.685360000007 | 1198.46329059750 | 1000 | 0.342587037475209 |
| 113.711040000007 | 1198.15854796230 | 1000 | 0.342399639217903 |
| 113.736720000007 | 1197.85128074108 | 1000 | 0.342211532410304 |
| 113.762400000007 | 1197.54147857725 | 1000 | 0.342022714507349 |
| 113.788080000007 | 1197.22913107297 | 1000 | 0.341833182955940 |
| 113.813760000007 | 1196.91422778897 | 1000 | 0.341642935194930 |
| 113.839440000007 | 1196.59675824441 | 1000 | 0.341451968655102 |
| 113.865120000007 | 1196.27671191671 | 1000 | 0.341260280759155 |
| 113.890800000007 | 1195.95407824141 | 1000 | 0.341067868921686 |
| 113.916480000007 | 1195.62884661199 | 1000 | 0.340874730549172 |
| 113.942160000007 | 1195.30100637974 | 1000 | 0.340680863039954 |
| 113.967840000008 | 1194.97054685358 | 1000 | 0.340486263784219 |
| 113.993520000007 | 1194.63745729991 | 1000 | 0.340290930163985 |
| 114.019200000008 | 1194.30172694247 | 1000 | 0.340094859553082 |
| 114.044880000008 | 1193.96334496213 | 1000 | 0.339898049317138 |
| 114.070560000008 | 1193.62230049679 | 1000 | 0.339700496813557 |
| 114.096240000008 | 1193.27858264119 | 1000 | 0.339502199391508 |
| 114.121920000008 | 1192.93218044672 | 1000 | 0.339303154391904 |
| 114.147600000008 | 1192.58308292133 | 1000 | 0.339103359147389 |
| 114.173280000008 | 1192.23127902931 | 1000 | 0.338902810982316 |
| 114.198960000008 | 1191.87675769112 | 1000 | 0.338701507212735 |
| 114.224640000008 | 1191.51950778331 | 1000 | 0.338499445146377 |
| 114.250320000008 | 1191.15951813823 | 1000 | 0.338296622082630 |
| 114.276000000008 | 1190.79677754400 | 1000 | 0.338093035312534 |
| 114.301680000008 | 1190.43127474422 | 1000 | 0.337888682118752 |
| 114.327360000008 | 1190.06299843790 | 1000 | 0.337683559775565 |
| 114.353040000008 | 1189.69193727926 | 1000 | 0.337477665548847 |
| 114.378720000008 | 1189.31807987755 | 1000 | 0.337270996696052 |
| 114.404400000008 | 1188.94141479689 | 1000 | 0.337063550466198 |
| 114.430080000008 | 1188.56193055613 | 1000 | 0.336855324099851 |
| 114.455760000008 | 1188.17961562864 | 1000 | 0.336646314829106 |
| 114.481440000008 | 1187.79445844217 | 1000 | 0.336436519877574 |
| 114.507120000008 | 1187.40644737869 | 1000 | 0.336225936460364 |
| 114.532800000008 | 1187.01557077417 | 1000 | 0.336014561784067 |
| 114.558480000008 | 1186.62181691848 | 1000 | 0.335802393046740 |
| 114.584160000008 | 1186.22517405516 | 1000 | 0.335589427437891 |
| 114.609840000008 | 1185.82563038129 | 1000 | 0.335375662138462 |
| 114.635520000008 | 1185.42317404729 | 1000 | 0.335161094320810 |
| 114.661200000008 | 1185.01779315676 | 1000 | 0.334945721148700 |
| 114.686880000008 | 1184.60947576632 | 1000 | 0.334729539777279 |
| 114.712560000008 | 1184.19820988540 | 1000 | 0.334512547353065 |
| 114.738240000008 | 1183.78398347613 | 1000 | 0.334294741013932 |
| 114.763920000008 | 1183.36678445308 | 1000 | 0.334076117889093 |
| 114.789600000008 | 1182.94660068316 | 1000 | 0.333856675099083 |
| 114.815280000008 | 1182.52341998542 | 1000 | 0.333636409755747 |
| 114.840960000008 | 1182.09723013086 | 1000 | 0.333415318962220 |
| 114.866640000008 | 1181.66801884225 | 1000 | 0.333193399812915 |
| 114.892320000008 | 1181.23577379400 | 1000 | 0.332970649393505 |
| 114.918000000008 | 1180.80048261193 | 1000 | 0.332747064780912 |
| 114.943680000008 | 1180.36213287312 | 1000 | 0.332522643043285 |
| 114.969360000008 | 1179.92071210571 | 1000 | 0.332297381239991 |
| 114.995040000008 | 1179.47620778874 | 1000 | 0.332071276421594 |
| 115.020720000008 | 1179.02860735199 | 1000 | 0.331844325629847 |
| 115.046400000008 | 1178.57789817573 | 1000 | 0.331616525897670 |
| 115.072080000008 | 1178.12406759063 | 1000 | 0.331387874249139 |
| 115.097760000008 | 1177.66710287750 | 1000 | 0.331158367699469 |
| 115.123440000008 | 1177.20699126716 | 1000 | 0.330928003255000 |
| 115.149120000008 | 1176.74371994022 | 1000 | 0.330696777913183 |
| 115.174800000008 | 1176.27727602693 | 1000 | 0.330464688662564 |
| 115.200480000008 | 1175.80764660700 | 1000 | 0.330231732482769 |
| 115.226160000008 | 1175.33481870935 | 1000 | 0.329997906344491 |
| 115.251840000008 | 1174.85877931203 | 1000 | 0.329763207209474 |
| 115.277520000008 | 1174.37951534193 | 1000 | 0.329527632030498 |
| 115.303200000008 | 1173.89701367468 | 1000 | 0.329291177751366 |
| 115.328880000008 | 1173.41126113440 | 1000 | 0.329053841306890 |
| 115.354560000008 | 1172.92224449357 | 1000 | 0.328815619622875 |
| 115.380240000008 | 1172.42995047278 | 1000 | 0.328576509616104 |
| 115.405920000008 | 1171.93436574060 | 1000 | 0.328336508194329 |
| 115.431600000008 | 1171.43547691335 | 1000 | 0.328095612256248 |
| 115.457280000008 | 1170.93327055495 | 1000 | 0.327853818691502 |
| 115.482960000008 | 1170.42773317668 | 1000 | 0.327611124380652 |
| 115.508640000008 | 1169.91885123703 | 1000 | 0.327367526195168 |
| 115.534320000008 | 1169.40661114151 | 1000 | 0.327123020997417 |
| 115.560000000008 | 1168.89099924243 | 1000 | 0.326877605640648 |
| 115.585680000008 | 1168.37200183873 | 1000 | 0.326631276968978 |
| 115.611360000008 | 1167.84960517578 | 1000 | 0.326384031817378 |
| 115.637040000008 | 1167.32379544519 | 1000 | 0.326135867011661 |
| 115.662720000008 | 1166.79455878462 | 1000 | 0.325886779368468 |
| 115.688400000008 | 1166.26188127758 | 1000 | 0.325636765695255 |
| 115.714080000008 | 1165.72574895322 | 1000 | 0.325385822790277 |
| 115.739760000008 | 1165.18614778618 | 1000 | 0.325133947442580 |
| 115.765440000008 | 1164.64306369635 | 1000 | 0.324881136431984 |
| 115.791120000008 | 1164.09648254869 | 1000 | 0.324627386529071 |
| 115.816800000008 | 1163.54639015304 | 1000 | 0.324372694495173 |
| 115.842480000008 | 1162.99277226390 | 1000 | 0.324117057082356 |
| 115.868160000008 | 1162.43561458026 | 1000 | 0.323860471033414 |
| 115.893840000008 | 1161.87490274539 | 1000 | 0.323602933081848 |
| 115.919520000008 | 1161.31062234663 | 1000 | 0.323344439951860 |
| 115.945200000008 | 1160.74275891521 | 1000 | 0.323084988358337 |
| 115.970880000008 | 1160.17129792604 | 1000 | 0.322824575006841 |
| 115.996560000008 | 1159.59622479749 | 1000 | 0.322563196593595 |
| 116.022240000008 | 1159.01752489123 | 1000 | 0.322300849805470 |
| 116.047920000008 | 1158.43518351199 | 1000 | 0.322037531319977 |
| 116.073600000008 | 1157.84918590739 | 1000 | 0.321773237805250 |
| 116.099280000008 | 1157.25951726770 | 1000 | 0.321507965920038 |
| 116.124960000008 | 1156.66616272566 | 1000 | 0.321241712313689 |
| 116.150640000008 | 1156.06910735628 | 1000 | 0.320974473626146 |
| 116.176320000008 | 1155.46833617662 | 1000 | 0.320706246487926 |
| 116.202000000008 | 1154.86383414560 | 1000 | 0.320437027520114 |
| 116.227680000008 | 1154.25558616377 | 1000 | 0.320166813334352 |
| 116.253360000008 | 1153.64357707313 | 1000 | 0.319895600532825 |
| 116.279040000008 | 1153.02779165691 | 1000 | 0.319623385708252 |
| 116.304720000008 | 1152.40821463936 | 1000 | 0.319350165443873 |
| 116.330400000008 | 1151.78483068555 | 1000 | 0.319075936313439 |
| 116.356080000008 | 1151.15762440115 | 1000 | 0.318800694881204 |
| 116.381760000008 | 1150.52658033223 | 1000 | 0.318524437701908 |
| 116.407440000008 | 1149.89168296506 | 1000 | 0.318247161320773 |
| 116.433120000008 | 1149.25291672586 | 1000 | 0.317968862273487 |
| 116.458800000008 | 1148.61026598063 | 1000 | 0.317689537086196 |
| 116.484480000008 | 1147.96371503494 | 1000 | 0.317409182275497 |
| 116.510160000008 | 1147.31324813366 | 1000 | 0.317127794348420 |
| 116.535840000008 | 1146.65884946083 | 1000 | 0.316845369802425 |
| 116.561520000008 | 1146.00050313938 | 1000 | 0.316561905125390 |
| 116.587200000008 | 1145.33819323095 | 1000 | 0.316277396795599 |
| 116.612880000008 | 1144.67190373566 | 1000 | 0.315991841281735 |
| 116.638560000008 | 1144.00161859190 | 1000 | 0.315705235042869 |
| 116.664240000008 | 1143.32732167613 | 1000 | 0.315417574528450 |
| 116.689920000008 | 1142.64899680263 | 1000 | 0.315128856178298 |
| 116.715600000008 | 1141.96662772332 | 1000 | 0.314839076422594 |
| 116.741280000008 | 1141.28019812750 | 1000 | 0.314548231681866 |
| 116.766960000008 | 1140.58969164167 | 1000 | 0.314256318366989 |
| 116.792640000008 | 1139.89509182930 | 1000 | 0.313963332879169 |
| 116.818320000008 | 1139.19638219061 | 1000 | 0.313669271609935 |
| 116.844000000008 | 1138.49354616233 | 1000 | 0.313374130941136 |
| 116.869680000008 | 1137.78656711752 | 1000 | 0.313077907244925 |
| 116.895360000008 | 1137.07542836532 | 1000 | 0.312780596883755 |
| 116.921040000008 | 1136.36011315072 | 1000 | 0.312482196210371 |
| 116.946720000008 | 1135.64060465438 | 1000 | 0.312182701567799 |
| 116.972400000008 | 1134.91688599235 | 1000 | 0.311882109289342 |
| 116.998080000008 | 1134.18894021591 | 1000 | 0.311580415698568 |
| 117.023760000008 | 1133.45675031127 | 1000 | 0.311277617109305 |
| 117.049440000008 | 1132.72029919944 | 1000 | 0.310973709825633 |
| 117.075120000008 | 1131.97956973592 | 1000 | 0.310668690141876 |
| 117.100800000008 | 1131.23454471051 | 1000 | 0.310362554342596 |
| 117.126480000008 | 1130.48520684708 | 1000 | 0.310055298702584 |
| 117.152160000008 | 1129.73153880338 | 1000 | 0.309746919486853 |
| 117.177840000008 | 1128.97352317072 | 1000 | 0.309437412950635 |
| 117.203520000008 | 1128.21114247385 | 1000 | 0.309126775339369 |
| 117.229200000008 | 1127.44437917065 | 1000 | 0.308815002888697 |
| 117.254880000009 | 1126.67321565196 | 1000 | 0.308502091824459 |
| 117.280560000009 | 1125.89763424128 | 1000 | 0.308188038362683 |
| 117.306240000009 | 1125.11761719463 | 1000 | 0.307872838709582 |
| 117.331920000009 | 1124.33314670024 | 1000 | 0.307556489061547 |
| 117.357600000009 | 1123.54420487837 | 1000 | 0.307238985605142 |
| 117.383280000009 | 1122.75077378104 | 1000 | 0.306920324517096 |
| 117.408960000009 | 1121.95283539182 | 1000 | 0.306600501964300 |
| 117.434640000009 | 1121.15037162560 | 1000 | 0.306279514103800 |
| 117.460320000009 | 1120.34336432836 | 1000 | 0.305957357082793 |
| 117.486000000009 | 1119.53179527689 | 1000 | 0.305634027038620 |
| 117.511680000009 | 1118.71564617863 | 1000 | 0.305309520098765 |
| 117.537360000009 | 1117.89489867136 | 1000 | 0.304983832380845 |
| 117.563040000009 | 1117.06953432302 | 1000 | 0.304656959992610 |
| 117.588720000009 | 1116.23953463145 | 1000 | 0.304328899031936 |
| 117.614400000009 | 1115.40488102415 | 1000 | 0.303999645586821 |
| 117.640080000009 | 1114.56555485804 | 1000 | 0.303669195735381 |
| 117.665760000009 | 1113.72153741926 | 1000 | 0.303337545545849 |
| 117.691440000009 | 1112.87280992286 | 1000 | 0.303004691076564 |
| 117.717120000009 | 1112.01935351262 | 1000 | 0.302670628375977 |
| 117.742800000009 | 1111.16114926080 | 1000 | 0.302335353482640 |
| 117.768480000009 | 1110.29817816788 | 1000 | 0.301998862425204 |
| 117.794160000009 | 1109.43042116234 | 1000 | 0.301661151222420 |
| 117.819840000009 | 1108.55785910038 | 1000 | 0.301322215883132 |
| 117.845520000009 | 1107.68047276574 | 1000 | 0.300982052406275 |
| 117.871200000009 | 1106.79824286941 | 1000 | 0.300640656780874 |
| 117.896880000009 | 1105.91115004940 | 1000 | 0.300298024986040 |
| 117.922560000009 | 1105.01917487049 | 1000 | 0.299954152990969 |
| 117.948240000009 | 1104.12229782400 | 1000 | 0.299609036754938 |
| 117.973920000009 | 1103.22049932754 | 1000 | 0.299262672227304 |
| 117.999600000009 | 1102.31375972474 | 1000 | 0.298915055347506 |
| 118.025280000009 | 1101.40205928506 | 1000 | 0.298566182045057 |
| 118.050960000009 | 1100.48537820348 | 1000 | 0.298216048239546 |
| 118.076640000009 | 1099.56369660028 | 1000 | 0.297864649840639 |
| 118.102320000009 | 1098.63699452082 | 1000 | 0.297511982748074 |
| 118.128000000009 | 1097.70525193525 | 1000 | 0.297158042851661 |
| 118.153680000009 | 1096.76844873826 | 1000 | 0.296802826031286 |
| 118.179360000009 | 1095.82656474888 | 1000 | 0.296446328156903 |
| 118.205040000009 | 1094.87957971017 | 1000 | 0.296088545088540 |
| 118.230720000009 | 1093.92747328901 | 1000 | 0.295729472676297 |
| 118.256400000009 | 1092.97022507583 | 1000 | 0.295369106760344 |
| 118.282080000009 | 1092.00781458438 | 1000 | 0.295007443170926 |
| 118.307760000009 | 1091.04022125142 | 1000 | 0.294644477728359 |
| 118.333440000009 | 1090.06742443657 | 1000 | 0.294280206243034 |
| 118.359120000009 | 1089.08940342193 | 1000 | 0.293914624515414 |
| 118.384800000009 | 1088.10613741196 | 1000 | 0.293547728336041 |
| 118.410480000009 | 1087.11760553309 | 1000 | 0.293179513485532 |
| 118.436160000009 | 1086.12378683359 | 1000 | 0.292809975734585 |
| 118.461840000009 | 1085.12466028322 | 1000 | 0.292439110843975 |
| 118.487520000009 | 1084.12020477304 | 1000 | 0.292066914564561 |
| 118.513200000009 | 1083.11039911510 | 1000 | 0.291693382637289 |
| 118.538880000009 | 1082.09522204222 | 1000 | 0.291318510793188 |
| 118.564560000009 | 1081.07465220772 | 1000 | 0.290942294753378 |
| 118.590240000009 | 1080.04866818516 | 1000 | 0.290564730229073 |
| 118.615920000009 | 1079.01724846810 | 1000 | 0.290185812921581 |
| 118.641600000009 | 1077.98037146979 | 1000 | 0.289805538522307 |
| 118.667280000009 | 1076.93801552299 | 1000 | 0.289423902712760 |
| 118.692960000009 | 1075.89015887963 | 1000 | 0.289040901164555 |
| 118.718640000009 | 1074.83677971060 | 1000 | 0.288656529539414 |
| 118.744320000009 | 1073.77785610546 | 1000 | 0.288270783489175 |
| 118.770000000009 | 1072.71336607223 | 1000 | 0.287883658655792 |
| 118.795680000009 | 1071.64328753703 | 1000 | 0.287495150671342 |
| 118.821360000009 | 1070.56759834392 | 1000 | 0.287105255158029 |
| 118.847040000009 | 1069.48627625459 | 1000 | 0.286713967728189 |
| 118.872720000009 | 1068.39929894808 | 1000 | 0.286321283984295 |
| 118.898400000009 | 1067.30664402056 | 1000 | 0.285927199518963 |
| 118.924080000009 | 1066.20828898501 | 1000 | 0.285531709914957 |
| 118.949760000009 | 1065.10421127101 | 1000 | 0.285134810745193 |
| 118.975440000009 | 1063.99438822445 | 1000 | 0.284736497572750 |
| 119.001120000009 | 1062.87879710725 | 1000 | 0.284336765950872 |
| 119.026800000009 | 1061.75741509710 | 1000 | 0.283935611422975 |
| 119.052480000009 | 1060.63021928723 | 1000 | 0.283533029522657 |
| 119.078160000009 | 1059.49718668607 | 1000 | 0.283129015773699 |
| 119.103840000009 | 1058.35829421707 | 1000 | 0.282723565690079 |
| 119.129520000009 | 1057.21351871834 | 1000 | 0.282316674775976 |
| 119.155200000009 | 1056.06283694245 | 1000 | 0.281908338525775 |
| 119.180880000009 | 1054.90622555613 | 1000 | 0.281498552424082 |
| 119.206560000009 | 1053.74366114001 | 1000 | 0.281087311945725 |
| 119.232240000009 | 1052.57512018832 | 1000 | 0.280674612555767 |
| 119.257920000009 | 1051.40057910868 | 1000 | 0.280260449709513 |
| 119.283600000009 | 1050.22001422177 | 1000 | 0.279844818852518 |
| 119.309280000009 | 1049.03340176106 | 1000 | 0.279427715420599 |
| 119.334960000009 | 1047.84071787261 | 1000 | 0.279009134839840 |
| 119.360640000009 | 1046.64193861469 | 1000 | 0.278589072526608 |
| 119.386320000009 | 1045.43703995758 | 1000 | 0.278167523887554 |
| 119.412000000009 | 1044.22599778329 | 1000 | 0.277744484319632 |
| 119.437680000009 | 1043.00878788525 | 1000 | 0.277319949210105 |
| 119.463360000009 | 1041.78538596807 | 1000 | 0.276893913936556 |
| 119.489040000009 | 1040.55576764723 | 1000 | 0.276466373866898 |
| 119.514720000009 | 1039.31990844886 | 1000 | 0.276037324359386 |
| 119.540400000009 | 1038.07778380939 | 1000 | 0.275606760762631 |
| 119.566080000009 | 1036.82936907534 | 1000 | 0.275174678415607 |
| 119.591760000009 | 1035.57463950301 | 1000 | 0.274741072647666 |
| 119.617440000009 | 1034.31357025818 | 1000 | 0.274305938778549 |
| 119.643120000009 | 1033.04613641591 | 1000 | 0.273869272118400 |
| 119.668800000009 | 1031.77231296016 | 1000 | 0.273431067967774 |
| 119.694480000009 | 1030.49207478358 | 1000 | 0.272991321617656 |
| 119.720160000009 | 1029.20539668723 | 1000 | 0.272550028349473 |
| 119.745840000009 | 1027.91225338025 | 1000 | 0.272107183435102 |
| 119.771520000009 | 1026.61261947963 | 1000 | 0.271662782136891 |
| 119.797200000009 | 1025.30646950991 | 1000 | 0.271216819707667 |
| 119.822880000009 | 1023.99377790290 | 1000 | 0.270769291390756 |
| 119.848560000009 | 1022.67451899739 | 1000 | 0.270320192419993 |
| 119.874240000009 | 1021.34866703888 | 1000 | 0.269869518019737 |
| 119.899920000009 | 1020.01619617931 | 1000 | 0.269417263404891 |
| 119.925600000009 | 1018.67708047672 | 1000 | 0.268963423780909 |
| 119.951280000009 | 1017.33129389506 | 1000 | 0.268507994343821 |
| 119.976960000009 | 1015.97881030381 | 1000 | 0.268050970280241 |
| 120.002640000009 | 1014.61960347777 | 1000 | 0.267592346767390 |
| 120.028320000009 | 1013.25364709674 | 1000 | 0.267132118973104 |
| 120.054000000009 | 1011.88091474521 | 1000 | 0.266670282055860 |
| 120.079680000009 | 1010.50137991216 | 1000 | 0.266206831164788 |
| 120.105360000009 | 1009.11501599067 | 1000 | 0.265741761439687 |
| 120.131040000009 | 1007.72179627772 | 1000 | 0.265275068011047 |
| 120.156720000009 | 1006.32169397385 | 1000 | 0.264806746000062 |
| 120.182400000009 | 1004.91468218289 | 1000 | 0.264336790518653 |
| 120.208080000009 | 1003.50073391169 | 1000 | 0.263865196669483 |
| 120.233760000009 | 1002.07982206980 | 1000 | 0.263391959545977 |
| 120.259440000009 | 1000.65191946921 | 1000 | 0.262917074232340 |
| 120.285120000009 | 999.216998824040 | 1000 | 0.262440535803577 |
| 120.310800000009 | 997.775032750276 | 1000 | 0.261962339325515 |
| 120.336480000009 | 996.325993765458 | 1000 | 0.261482479854818 |
| 120.362160000009 | 994.869854288404 | 1000 | 0.261000952439012 |
| 120.387840000009 | 993.406586638915 | 1000 | 0.260517752116500 |
| 120.413520000009 | 991.936163037493 | 1000 | 0.260032873916590 |
| 120.439200000009 | 990.458555605036 | 1000 | 0.259546312859508 |
| 120.464880000009 | 988.973736362563 | 1000 | 0.259058063956427 |
| 120.490560000009 | 987.481677230912 | 1000 | 0.258568122209482 |
| 120.516240000010 | 985.982350030456 | 1000 | 0.258076482611798 |
| 120.541920000010 | 984.475726480809 | 1000 | 0.257583140147507 |
| 120.567600000010 | 982.961778200533 | 1000 | 0.257088089791774 |
| 120.593280000010 | 981.440476706847 | 1000 | 0.256591326510817 |
| 120.618960000010 | 979.911793415332 | 1000 | 0.256092845261935 |
| 120.644640000010 | 978.375699639644 | 1000 | 0.255592640993524 |
| 120.670320000010 | 976.832166591219 | 1000 | 0.255090708645110 |
| 120.696000000010 | 975.281165378974 | 1000 | 0.254587043147365 |
| 120.721680000010 | 973.722667009019 | 1000 | 0.254081639422136 |
| 120.747360000010 | 972.156642384362 | 1000 | 0.253574492382465 |
| 120.773040000010 | 970.583062304618 | 1000 | 0.253065596932623 |
| 120.798720000010 | 969.001897465708 | 1000 | 0.252554947968126 |
| 120.824400000010 | 967.413118459566 | 1000 | 0.252042540375763 |
| 120.850080000010 | 965.816695773848 | 1000 | 0.251528369033627 |
| 120.875760000010 | 964.212599791638 | 1000 | 0.251012428811134 |
| 120.901440000010 | 962.600800791141 | 1000 | 0.250494714569056 |
| 120.927120000010 | 960.981268945400 | 1000 | 0.249975221159542 |
| 120.952800000010 | 959.353974321998 | 1000 | 0.249453943426150 |
| 120.978480000010 | 957.718886882754 | 1000 | 0.248930876203873 |
| 121.004160000010 | 956.075976483431 | 1000 | 0.248406014319164 |
| 121.029840000010 | 954.425212873447 | 1000 | 0.247879352589970 |
| 121.055520000010 | 952.766565695565 | 1000 | 0.247350885825754 |
| 121.081200000010 | 951.100004485604 | 1000 | 0.246820608827531 |
| 121.106880000010 | 949.425498672138 | 1000 | 0.246288516387888 |
| 121.132560000010 | 947.743017576207 | 1000 | 0.245754603291023 |
| 121.158240000010 | 946.052530411006 | 1000 | 0.245218864312769 |
| 121.183920000010 | 944.354006281594 | 1000 | 0.244681294220623 |
| 121.209600000010 | 942.647414184597 | 1000 | 0.244141887773782 |
| 121.235280000010 | 940.932723007910 | 1000 | 0.243600639723169 |
| 121.260960000010 | 939.209901530395 | 1000 | 0.243057544811467 |
| 121.286640000010 | 937.478918421588 | 1000 | 0.242512597773149 |
| 121.312320000010 | 935.739742241391 | 1000 | 0.241965793334508 |
| 121.338000000010 | 933.992341439780 | 1000 | 0.241417126213693 |
| 121.363680000010 | 932.236684356509 | 1000 | 0.240866591120740 |
| 121.389360000010 | 930.472739220804 | 1000 | 0.240314182757601 |
| 121.415040000010 | 928.700474151061 | 1000 | 0.239759895818183 |
| 121.440720000010 | 926.919857154561 | 1000 | 0.239203724988379 |
| 121.466400000010 | 925.130856127153 | 1000 | 0.238645664946099 |
| 121.492080000010 | 923.333438852964 | 1000 | 0.238085710361308 |
| 121.517760000010 | 921.527573004099 | 1000 | 0.237523855896058 |
| 121.543440000010 | 919.713226140340 | 1000 | 0.236960096204526 |
| 121.569120000010 | 917.890365708841 | 1000 | 0.236394425933044 |
| 121.594800000010 | 916.058959043836 | 1000 | 0.235826839720140 |
| 121.620480000010 | 914.218973366332 | 1000 | 0.235257332196567 |
| 121.646160000010 | 912.370375783813 | 1000 | 0.234685897985347 |
| 121.671840000010 | 910.513133289937 | 1000 | 0.234112531701802 |
| 121.697520000010 | 908.647212764237 | 1000 | 0.233537227953592 |
| 121.723200000010 | 906.772580971819 | 1000 | 0.232959981340754 |
| 121.748880000010 | 904.889204563063 | 1000 | 0.232380786455736 |
| 121.774560000010 | 902.997050073323 | 1000 | 0.231799637883439 |
| 121.800240000010 | 901.096083922618 | 1000 | 0.231216530201252 |
| 121.825920000010 | 899.186272415347 | 1000 | 0.230631457979091 |
| 121.851600000010 | 897.267581739975 | 1000 | 0.230044415779440 |
| 121.877280000010 | 895.339977968734 | 1000 | 0.229455398157386 |
| 121.902960000010 | 893.403427057329 | 1000 | 0.228864399660666 |
| 121.928640000010 | 891.457894844629 | 1000 | 0.228271414829696 |
| 121.954320000010 | 889.503347052373 | 1000 | 0.227676438197624 |
| 121.980000000010 | 887.539749284864 | 1000 | 0.227079464290359 |
| 122.005680000010 | 885.567067028671 | 1000 | 0.226480487626619 |
| 122.031360000010 | 883.585265652326 | 1000 | 0.225879502717970 |
| 122.057040000010 | 881.594310406028 | 1000 | 0.225276504068870 |
| 122.082720000010 | 879.594166421335 | 1000 | 0.224671486176705 |
| 122.108400000010 | 877.584798710869 | 1000 | 0.224064443531840 |
| 122.134080000010 | 875.566172168013 | 1000 | 0.223455370617654 |
| 122.159760000010 | 873.538251566613 | 1000 | 0.222844261910587 |
| 122.185440000010 | 871.501001560671 | 1000 | 0.222231111880184 |
| 122.211120000010 | 869.454386684055 | 1000 | 0.221615914989137 |
| 122.236800000010 | 867.398371350187 | 1000 | 0.220998665693328 |
| 122.262480000010 | 865.332919851752 | 1000 | 0.220379358441877 |
| 122.288160000010 | 863.257996360391 | 1000 | 0.219757987677185 |
| 122.313840000010 | 861.173564926409 | 1000 | 0.219134547834979 |
| 122.339520000010 | 859.079589478465 | 1000 | 0.218509033344356 |
| 122.365200000010 | 856.976033823278 | 1000 | 0.217881438627834 |
| 122.390880000010 | 854.862861645332 | 1000 | 0.217251758101394 |
| 122.416560000010 | 852.740036506566 | 1000 | 0.216619986174527 |
| 122.442240000010 | 850.607521846078 | 1000 | 0.215986117250282 |
| 122.467920000010 | 848.465280979833 | 1000 | 0.215350145725314 |
| 122.493600000010 | 846.313277100356 | 1000 | 0.214712065989929 |
| 122.519280000010 | 844.151473276435 | 1000 | 0.214071872428137 |
| 122.544960000010 | 841.979832452817 | 1000 | 0.213429559417694 |
| 122.570640000010 | 839.798317449927 | 1000 | 0.212785121330158 |
| 122.596320000010 | 837.606890963551 | 1000 | 0.212138552530931 |
| 122.622000000010 | 835.405515564542 | 1000 | 0.211489847379315 |
| 122.647680000010 | 833.194153698531 | 1000 | 0.210839000228556 |
| 122.673360000010 | 830.972767685613 | 1000 | 0.210186005425899 |
| 122.699040000010 | 828.741319720075 | 1000 | 0.209530857312637 |
| 122.724720000010 | 826.499771870075 | 1000 | 0.208873550224163 |
| 122.750400000010 | 824.248086077347 | 1000 | 0.208214078490016 |
| 122.776080000010 | 821.986224156917 | 1000 | 0.207552436433941 |
| 122.801760000010 | 819.714147796803 | 1000 | 0.206888618373934 |
| 122.827440000010 | 817.431818557716 | 1000 | 0.206222618622302 |
| 122.853120000010 | 815.139197872756 | 1000 | 0.205554431485705 |
| 122.878800000010 | 812.836247047133 | 1000 | 0.204884051265220 |
| 122.904480000010 | 810.522927257860 | 1000 | 0.204211472256390 |
| 122.930160000010 | 808.199199553466 | 1000 | 0.203536688749275 |
| 122.955840000010 | 805.865024853690 | 1000 | 0.202859695028513 |
| 122.981520000010 | 803.520363949196 | 1000 | 0.202180485373366 |
| 123.007200000010 | 801.165177501281 | 1000 | 0.201499054057784 |
| 123.032880000010 | 798.799426041575 | 1000 | 0.200815395350455 |
| 123.058560000010 | 796.423069971748 | 1000 | 0.200129503514862 |
| 123.084240000010 | 794.036069563222 | 1000 | 0.199441372809339 |
| 123.109920000010 | 791.638384956874 | 1000 | 0.198750997487127 |
| 123.135600000010 | 789.229976162749 | 1000 | 0.198058371796432 |
| 123.161280000010 | 786.810803059761 | 1000 | 0.197363489980480 |
| 123.186960000010 | 784.380825395413 | 1000 | 0.196666346277581 |
| 123.212640000010 | 781.940002785488 | 1000 | 0.195966934921175 |
| 123.238320000010 | 779.488294713789 | 1000 | 0.195265250139904 |
| 123.264000000010 | 777.025660531804 | 1000 | 0.194561286157657 |
| 123.289680000010 | 774.552059458469 | 1000 | 0.193855037193645 |
| 123.315360000010 | 772.067450579840 | 1000 | 0.193146497462444 |
| 123.341040000010 | 769.571792848822 | 1000 | 0.192435661174066 |
| 123.366720000010 | 767.065045084867 | 1000 | 0.191722522534013 |
| 123.392400000010 | 764.547165973708 | 1000 | 0.191007075743342 |
| 123.418080000010 | 762.018114067057 | 1000 | 0.190289314998722 |
| 123.443760000010 | 759.477847782324 | 1000 | 0.189569234492499 |
| 123.469440000010 | 756.926325402320 | 1000 | 0.188846828412754 |
| 123.495120000010 | 754.363505074992 | 1000 | 0.188122090943366 |
| 123.520800000010 | 751.789344813123 | 1000 | 0.187395016264076 |
| 123.546480000010 | 749.203802494053 | 1000 | 0.186665598550548 |
| 123.572160000010 | 746.606835859389 | 1000 | 0.185933831974433 |
| 123.597840000010 | 743.998402514734 | 1000 | 0.185199710703431 |
| 123.623520000010 | 741.378459929397 | 1000 | 0.184463228901356 |
| 123.649200000010 | 738.746965436112 | 1000 | 0.183724380728201 |
| 123.674880000010 | 736.103876230755 | 1000 | 0.182983160340199 |
| 123.700560000010 | 733.449149372073 | 1000 | 0.182239561889894 |
| 123.726240000010 | 730.782741781385 | 1000 | 0.181493579526197 |
| 123.751920000010 | 728.104610242326 | 1000 | 0.180745207394461 |
| 123.777600000011 | 725.414711400554 | 1000 | 0.179994439636543 |
| 123.803280000011 | 722.713001763473 | 1000 | 0.179241270390869 |
| 123.828960000011 | 719.999437699963 | 1000 | 0.178485693792502 |
| 123.854640000011 | 717.273975440095 | 1000 | 0.177727703973208 |
| 123.880320000011 | 714.536571074859 | 1000 | 0.176967295061526 |
| 123.906000000011 | 711.787180555897 | 1000 | 0.176204461182835 |
| 123.931680000011 | 709.025759695215 | 1000 | 0.175439196459418 |
| 123.957360000011 | 706.252264164916 | 1000 | 0.174671495010536 |
| 123.983040000011 | 703.466649496931 | 1000 | 0.173901350952494 |
| 124.008720000011 | 700.668871082743 | 1000 | 0.173128758398711 |
| 124.034400000011 | 697.858884173114 | 1000 | 0.172353711459788 |
| 124.060080000011 | 695.036643877822 | 1000 | 0.171576204243582 |
| 124.085760000011 | 692.202105165379 | 1000 | 0.170796230855268 |
| 124.111440000011 | 689.355222862784 | 1000 | 0.170013785397422 |
| 124.137120000011 | 686.495951655226 | 1000 | 0.169228861970077 |
| 124.162800000011 | 683.624246085851 | 1000 | 0.168441454670812 |
| 124.188480000011 | 680.740060555458 | 1000 | 0.167651557594803 |
| 124.214160000011 | 677.843349322267 | 1000 | 0.166859164834914 |
| 124.239840000011 | 674.934066501644 | 1000 | 0.166064270481758 |
| 124.265520000011 | 672.012166065831 | 1000 | 0.165266868623776 |
| 124.291200000011 | 669.077601843685 | 1000 | 0.164466953347301 |
| 124.316880000011 | 666.130327520426 | 1000 | 0.163664518736645 |
| 124.342560000011 | 663.170296637364 | 1000 | 0.162859558874158 |
| 124.368240000011 | 660.197462591657 | 1000 | 0.162052067840316 |
| 124.393920000011 | 657.211778636037 | 1000 | 0.161242039713787 |
| 124.419600000011 | 654.213197878547 | 1000 | 0.160429468571503 |
| 124.445280000011 | 651.201673282314 | 1000 | 0.159614348488747 |
| 124.470960000011 | 648.177157665262 | 1000 | 0.158796673539217 |
| 124.496640000011 | 645.139603699871 | 1000 | 0.157976437795106 |
| 124.522320000011 | 642.088963912929 | 1000 | 0.157153635327183 |
| 124.548000000011 | 639.025190685265 | 1000 | 0.156328260204858 |
| 124.573680000011 | 635.948236251519 | 1000 | 0.155500306496274 |
| 124.599360000011 | 632.858052699870 | 1000 | 0.154669768268369 |
| 124.625040000011 | 629.754591971796 | 1000 | 0.153836639586963 |
| 124.650720000011 | 626.637805861834 | 1000 | 0.153000914516836 |
| 124.676400000011 | 623.507646017323 | 1000 | 0.152162587121802 |
| 124.702080000011 | 620.364063938158 | 1000 | 0.151321651464787 |
| 124.727760000011 | 617.207010976554 | 1000 | 0.150478101607914 |
| 124.753440000011 | 614.036438336800 | 1000 | 0.149631931612578 |
| 124.779120000011 | 610.852297075013 | 1000 | 0.148783135539525 |
| 124.804800000011 | 607.654538098894 | 1000 | 0.147931707448931 |
| 124.830480000011 | 604.443112167499 | 1000 | 0.147077641400488 |
| 124.856160000011 | 601.217969891002 | 1000 | 0.146220931453481 |
| 124.881840000011 | 597.979061730444 | 1000 | 0.145361571666865 |
| 124.907520000011 | 594.726337997507 | 1000 | 0.144499556099353 |
| 124.933200000011 | 591.459748854272 | 1000 | 0.143634878809493 |
| 124.958880000011 | 588.179244313002 | 1000 | 0.142767533855752 |
| 124.984560000011 | 584.884774235898 | 1000 | 0.141897515296599 |
| 125.010240000011 | 581.576288334864 | 1000 | 0.141024817190584 |
| 125.035920000011 | 578.253736171288 | 1000 | 0.140149433596425 |
| 125.061600000011 | 574.917067155802 | 1000 | 0.139271358573085 |
| 125.087280000011 | 571.566230548085 | 1000 | 0.138390586179867 |
| 125.112960000011 | 568.201175456596 | 1000 | 0.137507110476484 |
| 125.138640000011 | 564.821850838380 | 1000 | 0.136620925523152 |
| 125.164320000011 | 561.428205498835 | 1000 | 0.135732025380672 |
| 125.190000000011 | 558.020188091498 | 1000 | 0.134840404110516 |
| 125.215680000011 | 554.597747117815 | 1000 | 0.133946055774908 |
| 125.241360000011 | 551.160830926931 | 1000 | 0.133048974436916 |
| 125.267040000011 | 547.709387715478 | 1000 | 0.132149154160530 |
| 125.292720000011 | 544.243365527349 | 1000 | 0.131246589010756 |
| 125.318400000011 | 540.762712253486 | 1000 | 0.130341273053694 |
| 125.344080000011 | 537.267375631682 | 1000 | 0.129433200356632 |
| 125.369760000011 | 533.757303246361 | 1000 | 0.128522364988131 |
| 125.395440000011 | 530.232442528358 | 1000 | 0.127608761018107 |
| 125.421120000011 | 526.692740754734 | 1000 | 0.126692382517924 |
| 125.446800000011 | 523.138145048560 | 1000 | 0.125773223560483 |
| 125.472480000011 | 519.568602378712 | 1000 | 0.124851278220304 |
| 125.498160000011 | 515.984059559667 | 1000 | 0.123926540573621 |
| 125.523840000011 | 512.384463251304 | 1000 | 0.122999004698463 |
| 125.549520000011 | 508.769759958721 | 1000 | 0.122068664674753 |
| 125.575200000011 | 505.139896032007 | 1000 | 0.121135514584389 |
| 125.600880000011 | 501.494817666080 | 1000 | 0.120199548511338 |
| 125.626560000011 | 497.834470900462 | 1000 | 0.119260760541722 |
| 125.652240000011 | 494.158801619104 | 1000 | 0.118319144763911 |
| 125.677920000011 | 490.467755550203 | 1000 | 0.117374695268616 |
| 125.703600000011 | 486.761278266002 | 1000 | 0.116427406148974 |
| 125.729280000011 | 483.039315182597 | 1000 | 0.115477271500641 |
| 125.754960000011 | 479.301811559768 | 1000 | 0.114524285421887 |
| 125.780640000011 | 475.548712500783 | 1000 | 0.113568442013681 |
| 125.806320000011 | 471.779962952237 | 1000 | 0.112609735379791 |
| 125.832000000011 | 467.995507703836 | 1000 | 0.111648159626865 |
| 125.857680000011 | 464.195291388260 | 1000 | 0.110683708864537 |
| 125.883360000011 | 460.379258480957 | 1000 | 0.109716377205506 |
| 125.909040000011 | 456.547353299996 | 1000 | 0.108746158765641 |
| 125.934720000011 | 452.699520005868 | 1000 | 0.107773047664065 |
| 125.960400000011 | 448.835702601325 | 1000 | 0.106797038023253 |
| 125.986080000011 | 444.955844931235 | 1000 | 0.105818123969125 |
| 126.011760000011 | 441.059890682380 | 1000 | 0.104836299631141 |
| 126.037440000011 | 437.147783383320 | 1000 | 0.103851559142393 |
| 126.063120000011 | 433.219466404213 | 1000 | 0.102863896639699 |
| 126.088800000011 | 429.274882956667 | 1000 | 0.101873306263701 |
| 126.114480000011 | 425.313976093582 | 1000 | 0.100879782158959 |
| 126.140160000011 | 421.336688708979 | 1000 | 0.0998833184740435 |
| 126.165840000011 | 417.342963537870 | 1000 | 0.0988839093616343 |
| 126.191520000011 | 413.332743156089 | 1000 | 0.0978815489786147 |
| 126.217200000011 | 409.305969980146 | 1000 | 0.0968762314861674 |
| 126.242880000011 | 405.262586267085 | 1000 | 0.0958679510498713 |
| 126.268560000011 | 401.202534114337 | 1000 | 0.0948567018397986 |
| 126.294240000011 | 397.125755459568 | 1000 | 0.0938424780306081 |
| 126.319920000011 | 393.032192080557 | 1000 | 0.0928252738016482 |
| 126.345600000011 | 388.921785595041 | 1000 | 0.0918050833370485 |
| 126.371280000011 | 384.794477460575 | 1000 | 0.0907819008258171 |
| 126.396960000011 | 380.650208974429 | 1000 | 0.0897557204619462 |
| 126.422640000011 | 376.488921273419 | 1000 | 0.0887265364444997 |
| 126.448320000011 | 372.310555333789 | 1000 | 0.0876943429777155 |
| 126.474000000011 | 368.115051971106 | 1000 | 0.0866591342711077 |
| 126.499680000011 | 363.902351840097 | 1000 | 0.0856209045395570 |
| 126.525360000011 | 359.672395434565 | 1000 | 0.0845796480034185 |
| 126.551040000011 | 355.425123087234 | 1000 | 0.0835353588886119 |
| 126.576720000011 | 351.160474969656 | 1000 | 0.0824880314267272 |
| 126.602400000011 | 346.878391092092 | 1000 | 0.0814376598551223 |
| 126.628080000011 | 342.578811303387 | 1000 | 0.0803842384170210 |
| 126.653760000011 | 338.261675290867 | 1000 | 0.0793277613616132 |
| 126.679440000011 | 333.926922580235 | 1000 | 0.0782682229441571 |
| 126.705120000011 | 329.574492535459 | 1000 | 0.0772056174260770 |
| 126.730800000011 | 325.204324358670 | 1000 | 0.0761399390750648 |
| 126.756480000011 | 320.816357090062 | 1000 | 0.0750711821651791 |
| 126.782160000011 | 316.410529607801 | 1000 | 0.0739993409769501 |
| 126.807840000011 | 311.986780627919 | 1000 | 0.0729244097974761 |
| 126.833520000011 | 307.545048704219 | 1000 | 0.0718463829205247 |
| 126.859200000011 | 303.085272228201 | 1000 | 0.0707652546466389 |
| 126.884880000011 | 298.607389428958 | 1000 | 0.0696810192832334 |
| 126.910560000011 | 294.111338373098 | 1000 | 0.0685936711446993 |
| 126.936240000011 | 289.597056964663 | 1000 | 0.0675032045525050 |
| 126.961920000011 | 285.064482945038 | 1000 | 0.0664096138352974 |
| 126.987600000011 | 280.513553892895 | 1000 | 0.0653128933290073 |
| 127.013280000011 | 275.944207224097 | 1000 | 0.0642130373769477 |
| 127.038960000011 | 271.356380191636 | 1000 | 0.0631100403299188 |
| 127.064640000012 | 266.750009885566 | 1000 | 0.0620038965463110 |
| 127.090320000012 | 262.125033232931 | 1000 | 0.0608946003922064 |
| 127.116000000012 | 257.481386997704 | 1000 | 0.0597821462414822 |
| 127.141680000012 | 252.819007780737 | 1000 | 0.0586665284759170 |
| 127.167360000012 | 248.137832019679 | 1000 | 0.0575477414852879 |
| 127.193040000012 | 243.437795988933 | 1000 | 0.0564257796674779 |
| 127.218720000012 | 238.718835799630 | 1000 | 0.0553006374285841 |
| 127.244400000012 | 233.980887399526 | 1000 | 0.0541723091830105 |
| 127.270080000012 | 229.223886573002 | 1000 | 0.0530407893535821 |
| 127.295760000012 | 224.447768940995 | 1000 | 0.0519060723716428 |
| 127.321440000012 | 219.652469960966 | 1000 | 0.0507681526771626 |
| 127.347120000012 | 214.837924926886 | 1000 | 0.0496270247188462 |
| 127.372800000012 | 210.004068969134 | 1000 | 0.0484826829542230 |
| 127.398480000012 | 205.150837054547 | 1000 | 0.0473351218497704 |
| 127.424160000012 | 200.278163986330 | 1000 | 0.0461843358810034 |
| 127.449840000012 | 195.385984404068 | 1000 | 0.0450303195325901 |
| 127.475520000012 | 190.474232783684 | 1000 | 0.0438730672984504 |
| 127.501200000012 | 185.542843437417 | 1000 | 0.0427125736818615 |
| 127.526880000012 | 180.591750513825 | 1000 | 0.0415488331955678 |
| 127.552560000012 | 175.620887997751 | 1000 | 0.0403818403618802 |
| 127.578240000012 | 170.630189710327 | 1000 | 0.0392115897127868 |
| 127.603920000012 | 165.619589308958 | 1000 | 0.0380380757900551 |
| 127.629600000012 | 160.589020287317 | 1000 | 0.0368612931453375 |
| 127.655280000012 | 155.538415975367 | 1000 | 0.0356812363402827 |
| 127.680960000012 | 150.467709539317 | 1000 | 0.0344978999466308 |
| 127.706640000012 | 145.376833981674 | 1000 | 0.0333112785463294 |
| 127.732320000012 | 140.265722141238 | 1000 | 0.0321213667316376 |
| 127.758000000012 | 135.134306693095 | 1000 | 0.0309281591052270 |
| 127.783680000012 | 129.982520148654 | 1000 | 0.0297316502802919 |
| 127.809360000012 | 124.810294855654 | 1000 | 0.0285318348806554 |
| 127.835040000012 | 119.617562998205 | 1000 | 0.0273287075408775 |
| 127.860720000012 | 114.404256596787 | 1000 | 0.0261222629063576 |
| 127.886400000012 | 109.170307508290 | 1000 | 0.0249124956334416 |
| 127.912080000012 | 103.915647426052 | 1000 | 0.0236994003895322 |
| 127.937760000012 | 98.6402078798924 | 1000 | 0.0224829718531929 |
| 127.963440000012 | 93.3439202361388 | 1000 | 0.0212632047142533 |
| 127.989120000012 | 88.0267156976897 | 1000 | 0.0200400936739202 |
| 128.014800000012 | 82.6885253040380 | 1000 | 0.0188136334448790 |
| 128.040480000012 | 77.3292799313405 | 1000 | 0.0175838187514059 |
| 128.066160000012 | 71.9489102924523 | 1000 | 0.0163506443294700 |
| 128.091840000012 | 66.5473469370027 | 1000 | 0.0151141049268452 |
| 128.117520000012 | 61.1245202514346 | 1000 | 0.0138741953032123 |
| 128.143200000012 | 55.6803604590916 | 1000 | 0.0126309102302720 |
| 128.168880000012 | 50.2147976202543 | 1000 | 0.0113842444918443 |
| 128.194560000012 | 44.7277616322327 | 1000 | 0.0101341928839815 |
| 128.220240000012 | 39.2191822294440 | 1000 | 0.00888075021507525 |
| 128.245920000012 | 33.6889889834649 | 1000 | 0.00762391130595906 |
| 128.271600000012 | 28.1371113031561 | 1000 | 0.00636367099002448 |
| 128.297280000012 | 22.5634784346817 | 1000 | 0.00510002411331394 |
| 128.322960000012 | 16.9680194616695 | 1000 | 0.00383296553464407 |
| 128.348640000012 | 11.3506633052471 | 1000 | 0.00256249012570035 |
| 128.374320000012 | 5.71133872418843 | 1000 | 0.00128859277115573 |
| 0.0256800000000000 | 0.229577459724757 | 750 | 0.284628890952022 |
| 0.0513600000000000 | 0.459153591954617 | 750 | 0.284627976930387 |
| 0.0770400000000000 | 0.688728396689549 | 750 | 0.284627062909387 |
| 0.102720000000000 | 0.918301873929522 | 750 | 0.284626148889022 |
| 0.128400000000000 | 1.14787402368999 | 750 | 0.284625234873559 |
| 0.154080000000000 | 1.37744484592446 | 750 | 0.284624320850201 |
| 0.179760000000000 | 1.60701434067937 | 750 | 0.284623406831744 |
| 0.205440000000000 | 1.83658250790928 | 750 | 0.284622492808774 |
| 0.231120000000000 | 2.06614934770389 | 750 | 0.284621578796735 |
| 0.256800000000000 | 2.29571485997345 | 750 | 0.284620664780184 |
| 0.282480000000000 | 2.52527904474782 | 750 | 0.284619750764268 |
| 0.308160000000000 | 2.75484190202698 | 750 | 0.284618836748988 |
| 0.333840000000000 | 2.98440343181090 | 750 | 0.284617922734342 |
| 0.359520000000000 | 3.21396363409953 | 750 | 0.284617008720332 |
| 0.385200000000000 | 3.44352250889285 | 750 | 0.284616094706957 |
| 0.410880000000000 | 3.67308005624261 | 750 | 0.284615180698674 |
| 0.436560000000000 | 3.90263627599342 | 750 | 0.284614266682112 |
| 0.462240000000000 | 4.13219116830061 | 750 | 0.284613352670641 |
| 0.487920000000000 | 4.36174473308997 | 750 | 0.284612438658183 |
| 0.513600000000000 | 4.59129697042861 | 750 | 0.284611524649605 |
| 0.539280000000000 | 4.82084788024936 | 750 | 0.284610610640039 |
| 0.564960000000000 | 5.05039746246855 | 750 | 0.284609696624472 |
| 0.590640000000000 | 5.27994571740417 | 750 | 0.284608782622810 |
| 0.616320000000000 | 5.50949264473817 | 750 | 0.284607868615148 |
| 0.642000000000000 | 5.73903824457652 | 750 | 0.284606954608120 |
| 0.667680000000000 | 5.96858251691918 | 750 | 0.284606040601726 |
| 0.693360000000000 | 6.19812546176613 | 750 | 0.284605126595967 |
| 0.719040000000000 | 6.42766707911731 | 750 | 0.284604212590842 |
| 0.744720000000000 | 6.65720736897270 | 750 | 0.284603298586351 |
| 0.770400000000000 | 6.88674633133560 | 750 | 0.284602384582647 |
| 0.796080000000001 | 7.11628396619597 | 750 | 0.284601470579271 |
| 0.821760000000001 | 7.34582027356377 | 750 | 0.284600556576682 |
| 0.847440000000001 | 7.57535525334616 | 750 | 0.284599642570994 |
| 0.873120000000001 | 7.80488890581153 | 750 | 0.284598728573407 |
| 0.898800000000001 | 8.03442123069141 | 750 | 0.284597814572719 |
| 0.924480000000001 | 8.26395222788912 | 750 | 0.284596900565546 |
| 0.950160000000001 | 8.49348189796301 | 750 | 0.284595986573246 |
| 0.975840000000001 | 8.72301024048787 | 750 | 0.284595072579288 |
| 1.00152000000000 | 8.95253725525013 | 750 | 0.284594158576307 |
| 1.02720000000000 | 9.18206294264941 | 750 | 0.284593244578788 |
| 1.05288000000000 | 9.41158730259050 | 750 | 0.284592330583180 |
| 1.07856000000000 | 9.64111033495924 | 750 | 0.284591416585650 |
| 1.10424000000000 | 9.87063203986971 | 750 | 0.284590502590030 |
| 1.12992000000000 | 10.1001524172235 | 750 | 0.284589588593156 |
| 1.15560000000000 | 10.3296714672016 | 750 | 0.284588674600691 |
| 1.18128000000000 | 10.5591891898987 | 750 | 0.284587760615229 |
| 1.20696000000000 | 10.7887055843864 | 750 | 0.284586846609158 |
| 1.23264000000000 | 11.0182206519761 | 750 | 0.284585932621431 |
| 1.25832000000000 | 11.2477343920864 | 750 | 0.284585018634627 |
| 1.28400000000000 | 11.4772468040788 | 750 | 0.284584104631143 |
| 1.30968000000000 | 11.7067578892817 | 750 | 0.284583190647865 |
| 1.33536000000000 | 11.9362676467236 | 750 | 0.284582276657942 |
| 1.36104000000000 | 12.1657760766689 | 750 | 0.284581362668652 |
| 1.38672000000000 | 12.3952831791174 | 750 | 0.284580448679994 |
| 1.41240000000000 | 12.6247889540459 | 750 | 0.284579534691389 |
| 1.43808000000000 | 12.8542934015240 | 750 | 0.284578620704575 |
| 1.46376000000000 | 13.0837965218102 | 750 | 0.284577706725743 |
| 1.48944000000000 | 13.3132983138163 | 750 | 0.284576792728674 |
| 1.51512000000000 | 13.5427987789072 | 750 | 0.284575878746188 |
| 1.54080000000000 | 13.7722979166016 | 750 | 0.284574964766538 |
| 1.56648000000000 | 14.0017957263446 | 750 | 0.284574050777091 |
| 1.59216000000000 | 14.2312922088176 | 750 | 0.284573136793491 |
| 1.61784000000000 | 14.4607873639181 | 750 | 0.284572222813242 |
| 1.64352000000000 | 14.6902811912724 | 750 | 0.284571308828185 |
| 1.66920000000000 | 14.9197736912540 | 750 | 0.284570394846479 |
| 1.69488000000000 | 15.1492648637586 | 750 | 0.284569480865829 |
| 1.72056000000000 | 15.3787547082793 | 750 | 0.284568566875796 |
| 1.74624000000000 | 15.6082432265978 | 750 | 0.284567652912903 |
| 1.77192000000000 | 15.8377304161226 | 750 | 0.284566738924280 |
| 1.79760000000000 | 16.0672162787023 | 750 | 0.284565824947425 |
| 1.82328000000000 | 16.2967008139788 | 750 | 0.284564910974920 |
| 1.84896000000000 | 16.5261840211998 | 750 | 0.284563996992222 |
| 1.87464000000000 | 16.7556659012022 | 750 | 0.284563083015568 |
| 1.90032000000000 | 16.9851464538817 | 750 | 0.284562169042796 |
| 1.92600000000000 | 17.2146256784152 | 750 | 0.284561255058664 |
| 1.95168000000000 | 17.4441035762236 | 750 | 0.284560341089390 |
| 1.97736000000000 | 17.6735801463048 | 750 | 0.284559427116501 |
| 2.00304000000000 | 17.9030553883417 | 750 | 0.284558513134563 |
| 2.02872000000000 | 18.1325293037654 | 750 | 0.284557599168889 |
| 2.05440000000000 | 18.3620018912836 | 750 | 0.284556685196650 |
| 2.08008000000000 | 18.5914731513040 | 750 | 0.284555771225041 |
| 2.10576000000000 | 18.8209430838263 | 750 | 0.284554857254063 |
| 2.13144000000000 | 19.0504116887062 | 750 | 0.284553943281318 |
| 2.15712000000000 | 19.2798789663770 | 750 | 0.284553029313997 |
| 2.18280000000000 | 19.5093449164051 | 750 | 0.284552115344910 |
| 2.20848000000000 | 19.7388095389352 | 750 | 0.284551201376452 |
| 2.23416000000000 | 19.9682728339670 | 750 | 0.284550287408625 |
| 2.25984000000000 | 20.1977348015006 | 750 | 0.284549373441427 |
| 2.28552000000000 | 20.4271954415358 | 750 | 0.284548459474859 |
| 2.31120000000000 | 20.6566547545737 | 750 | 0.284547545516586 |
| 2.33688000000000 | 20.8861127391274 | 750 | 0.284546631543857 |
| 2.36256000000000 | 21.1155693961807 | 750 | 0.284545717571890 |
| 2.38824000000000 | 21.3450247270877 | 750 | 0.284544803620731 |
| 2.41392000000000 | 21.5744787292359 | 750 | 0.284543889651465 |
| 2.43960000000000 | 21.8039314042803 | 750 | 0.284542975688674 |
| 2.46528000000001 | 22.0333827521149 | 750 | 0.284542061730655 |
| 2.49096000000000 | 22.2628327718732 | 750 | 0.284541147764981 |
| 2.51664000000001 | 22.4922814644215 | 750 | 0.284540233804077 |
| 2.54232000000000 | 22.7217288296525 | 750 | 0.284539319846328 |
| 2.56800000000001 | 22.9511748670217 | 750 | 0.284538405884158 |
| 2.59368000000001 | 23.1806195776387 | 750 | 0.284537491932848 |
| 2.61936000000001 | 23.4100629597007 | 750 | 0.284536577967759 |
| 2.64504000000001 | 23.6395050142626 | 750 | 0.284535664003415 |
| 2.67072000000001 | 23.8689457426941 | 750 | 0.284534750057947 |
| 2.69640000000001 | 24.0983851422574 | 750 | 0.284533836094930 |
| 2.72208000000001 | 24.3278232148468 | 750 | 0.284532922139489 |
| 2.74776000000001 | 24.5572599599040 | 750 | 0.284532008184244 |
| 2.77344000000001 | 24.7866953773205 | 750 | 0.284531094227823 |
| 2.79912000000001 | 25.0161294682606 | 750 | 0.284530180284986 |
| 2.82480000000001 | 25.2455622300799 | 750 | 0.284529266322277 |
| 2.85048000000001 | 25.4749936648902 | 750 | 0.284528352366449 |
| 2.87616000000001 | 25.7044237733344 | 750 | 0.284527438425238 |
| 2.90184000000001 | 25.9338525527607 | 750 | 0.284526524465962 |
| 2.92752000000001 | 26.1632800053219 | 750 | 0.284525610515112 |
| 2.95320000000001 | 26.3927061309111 | 750 | 0.284524696571209 |
| 2.97888000000001 | 26.6221309279760 | 750 | 0.284523782615661 |
| 3.00456000000001 | 26.8515543980072 | 750 | 0.284522868666328 |
| 3.03024000000001 | 27.0809765409909 | 750 | 0.284521954722907 |
| 3.05592000000001 | 27.3103973555551 | 750 | 0.284521040769389 |
| 3.08160000000001 | 27.5398168426179 | 750 | 0.284520126816579 |
| 3.10728000000001 | 27.7692350032565 | 750 | 0.284519212876732 |
| 3.13296000000001 | 27.9986518356339 | 750 | 0.284518298928814 |
| 3.15864000000001 | 28.2280673415883 | 750 | 0.284517384993614 |
| 3.18432000000001 | 28.4574815184430 | 750 | 0.284516471041118 |
| 3.21000000000001 | 28.6868943682122 | 750 | 0.284515557093944 |
| 3.23568000000001 | 28.9163058902727 | 750 | 0.284514643145170 |
| 3.26136000000001 | 29.1457160862277 | 750 | 0.284513729212231 |
| 3.28704000000001 | 29.3751249536882 | 750 | 0.284512815269073 |
| 3.31272000000001 | 29.6045324928455 | 750 | 0.284511901317988 |
| 3.33840000000001 | 29.8339387063016 | 750 | 0.284510987386737 |
| 3.36408000000001 | 30.0633435922584 | 750 | 0.284510073455980 |
| 3.38976000000001 | 30.2927471493150 | 750 | 0.284509159511105 |
| 3.41544000000001 | 30.5221493789838 | 750 | 0.284508245568107 |
| 3.44112000000001 | 30.7515502823259 | 750 | 0.284507331637859 |
| 3.46680000000001 | 30.9809498565970 | 750 | 0.284506417692101 |
| 3.49248000000001 | 31.2103481039311 | 750 | 0.284505503752797 |
| 3.51816000000001 | 31.4397450248866 | 750 | 0.284504589825432 |
| 3.54384000000001 | 31.6691406167600 | 750 | 0.284503675882786 |
| 3.56952000000001 | 31.8985348818123 | 750 | 0.284502761947598 |
| 3.59520000000001 | 32.1279278185303 | 750 | 0.284501848004842 |
| 3.62088000000001 | 32.3573194294826 | 750 | 0.284500934079774 |
| 3.64656000000001 | 32.5867097115261 | 750 | 0.284500020141559 |
| 3.67224000000001 | 32.8160986660677 | 750 | 0.284499106204032 |
| 3.69792000000001 | 33.0454862945190 | 750 | 0.284498192280689 |
| 3.72360000000001 | 33.2748725940568 | 750 | 0.284497278344443 |
| 3.74928000000001 | 33.5042575675049 | 750 | 0.284496364422202 |
| 3.77496000000001 | 33.7336412121837 | 750 | 0.284495450488595 |
| 3.80064000000001 | 33.9630235290731 | 750 | 0.284494536552981 |
| 3.82632000000001 | 34.1924045200191 | 750 | 0.284493622632474 |
| 3.85200000000001 | 34.4217841820477 | 750 | 0.284492708699483 |
| 3.87768000000001 | 34.6511625165754 | 750 | 0.284491794767187 |
| 3.90336000000001 | 34.8805395250159 | 750 | 0.284490880848391 |
| 3.92904000000001 | 35.1099152045380 | 750 | 0.284489966917365 |
| 3.95472000000001 | 35.3392895579770 | 750 | 0.284489052999709 |
| 3.98040000000001 | 35.5686625824485 | 750 | 0.284488139069553 |
| 4.00608000000001 | 35.7980342809305 | 750 | 0.284487225153437 |
| 4.03176000000001 | 36.0274046504435 | 750 | 0.284486311224973 |
| 4.05744000000001 | 36.2567736925133 | 750 | 0.284485397297705 |
| 4.08312000000001 | 36.4861414083839 | 750 | 0.284484483382395 |
| 4.10880000000001 | 36.7155077939672 | 750 | 0.284483569443651 |
| 4.13448000000001 | 36.9448728550563 | 750 | 0.284482655531465 |
| 4.16016000000001 | 37.1742365868920 | 750 | 0.284481741604906 |
| 4.18584000000001 | 37.4035989928133 | 750 | 0.284480827692458 |
| 4.21152000000001 | 37.6329600712312 | 750 | 0.284479913780533 |
| 4.23720000000001 | 37.8623198207236 | 750 | 0.284478999857265 |
| 4.26288000000001 | 38.0916782427113 | 750 | 0.284478085934656 |
| 4.28856000000001 | 38.3210353386218 | 750 | 0.284477172024476 |
| 4.31424000000001 | 38.5503911041780 | 750 | 0.284476258091461 |
| 4.33992000000001 | 38.7797455450506 | 750 | 0.284475344182228 |
| 4.36560000000001 | 39.0090986570585 | 750 | 0.284474430262504 |
| 4.39128000000001 | 39.2384504415293 | 750 | 0.284473516343175 |
| 4.41696000000001 | 39.4678008999976 | 750 | 0.284472602436530 |
| 4.44264000000001 | 39.6971500308203 | 750 | 0.284471688529283 |
| 4.46832000000001 | 39.9264978313508 | 750 | 0.284470774600513 |
| 4.49400000000001 | 40.1558443072390 | 750 | 0.284469860695053 |
| 4.51968000000001 | 40.3851894556229 | 750 | 0.284468946790126 |
| 4.54536000000001 | 40.6145332736418 | 750 | 0.284468032863481 |
| 4.57104000000001 | 40.8438757684507 | 750 | 0.284467118970842 |
| 4.59672000000001 | 41.0732169328935 | 750 | 0.284466205056603 |
| 4.62240000000001 | 41.3025567683974 | 750 | 0.284465291132053 |
| 4.64808000000001 | 41.5318952792633 | 750 | 0.284464377230091 |
| 4.67376000000001 | 41.7612324626273 | 750 | 0.284463463328689 |
| 4.69944000000001 | 41.9905683170517 | 750 | 0.284462549417030 |
| 4.72512000000001 | 42.2199028454080 | 750 | 0.284461635516782 |
| 4.75080000000001 | 42.4492360443852 | 750 | 0.284460721603125 |
| 4.77648000000001 | 42.6785679181721 | 750 | 0.284459807707297 |
| 4.80216000000001 | 42.9078984611420 | 750 | 0.284458893787590 |
| 4.82784000000001 | 43.1372276791437 | 750 | 0.284457979887233 |
| 4.85352000000001 | 43.3665555688785 | 750 | 0.284457065981897 |
| 4.87920000000001 | 43.5958821307701 | 750 | 0.284456152074743 |
| 4.90488000000001 | 43.8252073665966 | 750 | 0.284455238178625 |
| 4.93056000000001 | 44.0545312734785 | 750 | 0.284454324272732 |
| 4.95624000000001 | 44.2838538528565 | 750 | 0.284453410367505 |
| 4.98192000000001 | 44.5131751047277 | 750 | 0.284452496462923 |
| 5.00760000000001 | 44.7424950305337 | 750 | 0.284451582569165 |
| 5.03328000000001 | 44.9718136273959 | 750 | 0.284450668665853 |
| 5.05896000000001 | 45.2011308967510 | 750 | 0.284449754763184 |
| 5.08464000000001 | 45.4304468400435 | 750 | 0.284448840871202 |
| 5.11032000000001 | 45.6597614543876 | 750 | 0.284447926969791 |
| 5.13600000000001 | 45.8890747426698 | 750 | 0.284447013078972 |
| 5.16168000000001 | 46.1183867034454 | 750 | 0.284446099188702 |
| 5.18736000000001 | 46.3476973338294 | 750 | 0.284445185279317 |
| 5.21304000000001 | 46.5770066395952 | 750 | 0.284444271390267 |
| 5.23872000000001 | 46.8063146164101 | 750 | 0.284443357492021 |
| 5.26440000000001 | 47.0356212669141 | 750 | 0.284442443602453 |
| 5.29008000000001 | 47.2649265889674 | 750 | 0.284441529707145 |
| 5.31576000000001 | 47.4942305847104 | 750 | 0.284440615820426 |
| 5.34144000000001 | 47.7235332529462 | 750 | 0.284439701934259 |
| 5.36712000000001 | 47.9528345907842 | 750 | 0.284438788029603 |
| 5.39280000000001 | 48.1821346040089 | 750 | 0.284437874144656 |
| 5.41848000000001 | 48.4114332868326 | 750 | 0.284436960241382 |
| 5.44416000000001 | 48.6407306450445 | 750 | 0.284436046357646 |
| 5.46984000000001 | 48.8700266757486 | 750 | 0.284435132474463 |
| 5.49552000000001 | 49.0993213775001 | 750 | 0.284434218582537 |
| 5.52120000000001 | 49.3286147517429 | 750 | 0.284433304691249 |
| 5.54688000000001 | 49.5579067999271 | 750 | 0.284432390809840 |
| 5.57256000000001 | 49.7871975206062 | 750 | 0.284431476929005 |
| 5.59824000000001 | 50.0164869123279 | 750 | 0.284430563039573 |
| 5.62392000000001 | 50.2457749750938 | 750 | 0.284429649141681 |
| 5.64960000000001 | 50.4750617132500 | 750 | 0.284428735262651 |
| 5.67528000000001 | 50.7043471239006 | 750 | 0.284427821384197 |
| 5.70096000000001 | 50.9336312055920 | 750 | 0.284426907497302 |
| 5.72664000000001 | 51.1629139597756 | 750 | 0.284425993611051 |
| 5.75232000000001 | 51.3921953864526 | 750 | 0.284425079725451 |
| 5.77800000000001 | 51.6214754870718 | 750 | 0.284424165849368 |
| 5.80368000000001 | 51.8507542587330 | 750 | 0.284423251965015 |
| 5.82936000000001 | 52.0800317043368 | 750 | 0.284422338090104 |
| 5.85504000000001 | 52.3093078209816 | 750 | 0.284421424206997 |
| 5.88072000000001 | 52.5385826101166 | 750 | 0.284420510324522 |
| 5.90640000000001 | 52.7678560731964 | 750 | 0.284419596451388 |
| 5.93208000000001 | 52.9971282073159 | 750 | 0.284418682570166 |
| 5.95776000000001 | 53.2263990139253 | 750 | 0.284417768689576 |
| 5.98344000000001 | 53.4556684944815 | 750 | 0.284416854818228 |
| 6.00912000000001 | 53.6849366460734 | 750 | 0.284415940938881 |
| 6.03480000000001 | 53.9142034701577 | 750 | 0.284415027060182 |
| 6.06048000000001 | 54.1434689681866 | 750 | 0.284414113190602 |
| 6.08616000000001 | 54.3727331372514 | 750 | 0.284413199313136 |
| 6.11184000000001 | 54.6019959802641 | 750 | 0.284412285444738 |
| 6.13752000000001 | 54.8312574943104 | 750 | 0.284411371568514 |
| 6.16320000000001 | 55.0605176808486 | 750 | 0.284410457692935 |
| 6.18888000000001 | 55.2897765398756 | 750 | 0.284409543817985 |
| 6.21456000000002 | 55.5190340728514 | 750 | 0.284408629951968 |
| 6.24024000000001 | 55.7482902768591 | 750 | 0.284407716078258 |
| 6.26592000000001 | 55.9775451548145 | 750 | 0.284406802213407 |
| 6.29160000000001 | 56.2067987023461 | 750 | 0.284405888332751 |
| 6.31728000000001 | 56.4360509252818 | 750 | 0.284404974469107 |
| 6.34296000000002 | 56.6653018207092 | 750 | 0.284404060606043 |
| 6.36864000000001 | 56.8945513871666 | 750 | 0.284403146735444 |
| 6.39432000000001 | 57.1237996261149 | 750 | 0.284402232865487 |
| 6.42000000000002 | 57.3530465390105 | 750 | 0.284401319004192 |
| 6.44568000000001 | 57.5822921229363 | 750 | 0.284400405135464 |
| 6.47136000000002 | 57.8115363808126 | 750 | 0.284399491275353 |
| 6.49704000000001 | 58.0407793097170 | 750 | 0.284398577407860 |
| 6.52272000000002 | 58.2700209125720 | 750 | 0.284397663548924 |
| 6.54840000000002 | 58.4992611864544 | 750 | 0.284396749682666 |
| 6.57408000000002 | 58.7285001313662 | 750 | 0.284395835809192 |
| 6.59976000000002 | 58.9577377516868 | 750 | 0.284394921952055 |
| 6.62544000000002 | 59.1869740444964 | 750 | 0.284394008095490 |
| 6.65112000000002 | 59.4162090083348 | 750 | 0.284393094231737 |
| 6.67680000000002 | 59.6454426446600 | 750 | 0.284392180368607 |
| 6.70248000000002 | 59.8746749534748 | 750 | 0.284391266506115 |
| 6.72816000000002 | 60.1039059347762 | 750 | 0.284390352644245 |
| 6.75384000000002 | 60.3331355885669 | 750 | 0.284389438783012 |
| 6.77952000000002 | 60.5623639177697 | 750 | 0.284388524937659 |
| 6.80520000000002 | 60.7915909165350 | 750 | 0.284387611077621 |
| 6.83088000000002 | 61.0208165892527 | 750 | 0.284386697225794 |
| 6.85656000000002 | 61.2500409329933 | 750 | 0.284385783366986 |
| 6.88224000000002 | 61.4792639492227 | 750 | 0.284384869508814 |
| 6.90792000000002 | 61.7084856394018 | 750 | 0.284383955658756 |
| 6.93360000000002 | 61.9377060006058 | 750 | 0.284383041801812 |
| 6.95928000000002 | 62.1669250342952 | 750 | 0.284382127945489 |
| 6.98496000000002 | 62.3961427404715 | 750 | 0.284381214089792 |
| 7.01064000000002 | 62.6253591220659 | 750 | 0.284380300249506 |
| 7.03632000000002 | 62.8545741717509 | 750 | 0.284379386387648 |
| 7.06200000000002 | 63.0837878953887 | 750 | 0.284378472533811 |
| 7.08768000000002 | 63.3130002944427 | 750 | 0.284377558695215 |
| 7.11336000000002 | 63.5422113615877 | 750 | 0.284376644835293 |
| 7.13904000000002 | 63.7714211041491 | 750 | 0.284375730990561 |
| 7.16472000000002 | 64.0006295191984 | 750 | 0.284374817146411 |
| 7.19040000000002 | 64.2298366052660 | 750 | 0.284373903295617 |
| 7.21608000000002 | 64.4590423638193 | 750 | 0.284372989445448 |
| 7.24176000000002 | 64.6882467933927 | 750 | 0.284372075588748 |
| 7.26744000000002 | 64.9174498998513 | 750 | 0.284371161754127 |
| 7.29312000000002 | 65.1466516758627 | 750 | 0.284370247905811 |
| 7.31880000000002 | 65.3758521243579 | 750 | 0.284369334058112 |
| 7.34448000000002 | 65.6050512468074 | 750 | 0.284368420218109 |
| 7.37016000000002 | 65.8342490388048 | 750 | 0.284367506364589 |
| 7.39584000000002 | 66.0634455076913 | 750 | 0.284366592532795 |
| 7.42152000000002 | 66.2926406446594 | 750 | 0.284365678680549 |
| 7.44720000000002 | 66.5218344585162 | 750 | 0.284364764849882 |
| 7.47288000000002 | 66.7510269404532 | 750 | 0.284363850998903 |
| 7.49856000000002 | 66.9802180963416 | 750 | 0.284362937155510 |
| 7.52424000000002 | 67.2094079261848 | 750 | 0.284362023319648 |
| 7.54992000000002 | 67.4385964285111 | 750 | 0.284361109484355 |
| 7.57560000000002 | 67.6677836003822 | 750 | 0.284360195635919 |
| 7.60128000000002 | 67.8969694476780 | 750 | 0.284359281801828 |
| 7.62696000000002 | 68.1261539659861 | 750 | 0.284358367961491 |
| 7.65264000000002 | 68.3553371567793 | 750 | 0.284357454121780 |
| 7.67832000000002 | 68.5845190215247 | 750 | 0.284356540289452 |
| 7.70400000000002 | 68.8136995572845 | 750 | 0.284355626450956 |
| 7.72968000000002 | 69.0428787655259 | 750 | 0.284354712613072 |
| 7.75536000000002 | 69.2720566462502 | 750 | 0.284353798775806 |
| 7.78104000000002 | 69.5012332038709 | 750 | 0.284352884959213 |
| 7.80672000000002 | 69.7304084280927 | 750 | 0.284351971116468 |
| 7.83240000000002 | 69.9595823262652 | 750 | 0.284351057281012 |
| 7.85808000000002 | 70.1887548969200 | 750 | 0.284350143446174 |
| 7.88376000000002 | 70.4179261415290 | 750 | 0.284349229618552 |
| 7.90944000000002 | 70.6470960571483 | 750 | 0.284348315784926 |
| 7.93512000000002 | 70.8762646467246 | 750 | 0.284347401958489 |
| 7.96080000000002 | 71.1054319073077 | 750 | 0.284346488126074 |
| 7.98648000000002 | 71.3345978403724 | 750 | 0.284345574294274 |
| 8.01216000000002 | 71.5637624473913 | 750 | 0.284344660469589 |
| 8.03784000000002 | 71.7929257254218 | 750 | 0.284343746639013 |
| 8.06352000000002 | 72.0220876759306 | 750 | 0.284342832809038 |
| 8.08920000000002 | 72.2512483003936 | 750 | 0.284341918986118 |
| 8.11488000000002 | 72.4804075958644 | 750 | 0.284341005157352 |
| 8.14056000000002 | 72.7095655652895 | 750 | 0.284340091335600 |
| 8.16624000000002 | 72.9387222042507 | 750 | 0.284339177501674 |
| 8.19192000000002 | 73.1678775186370 | 750 | 0.284338263681111 |
| 8.21760000000002 | 73.3970315055040 | 750 | 0.284337349861122 |
| 8.24328000000002 | 73.6261841633770 | 750 | 0.284336436035384 |
| 8.26896000000002 | 73.8553354937298 | 750 | 0.284335522210259 |
| 8.29464000000002 | 74.0844854980402 | 750 | 0.284334608392044 |
| 8.32032000000002 | 74.3136341733526 | 750 | 0.284333694568123 |
| 8.34600000000002 | 74.5427815196693 | 750 | 0.284332780738566 |
| 8.37168000000002 | 74.7719275414155 | 750 | 0.284331866922117 |
| 8.39736000000002 | 75.0010722356441 | 750 | 0.284330953106254 |
| 8.42304000000002 | 75.2302156008733 | 750 | 0.284330039284760 |
| 8.44872000000002 | 75.4593576385815 | 750 | 0.284329125463876 |
| 8.47440000000002 | 75.6884983487683 | 750 | 0.284328211643602 |
| 8.50008000000002 | 75.9176377314334 | 750 | 0.284327297823938 |
| 8.52576000000002 | 76.1467757895324 | 750 | 0.284326384017138 |
| 8.55144000000002 | 76.3759125171544 | 750 | 0.284325470198657 |
| 8.57712000000002 | 76.6050479172543 | 750 | 0.284324556380785 |
| 8.60280000000002 | 76.8341819913087 | 750 | 0.284323642569590 |
| 8.62848000000002 | 77.0633147363642 | 750 | 0.284322728752917 |
| 8.65416000000002 | 77.2924461524232 | 750 | 0.284321814930831 |
| 8.67984000000002 | 77.5215762439105 | 750 | 0.284320901121407 |
| 8.70552000000002 | 77.7507050093525 | 750 | 0.284319987318557 |
| 8.73120000000002 | 77.9798324413623 | 750 | 0.284319073492316 |
| 8.75688000000002 | 78.2089585502840 | 750 | 0.284318159684657 |
| 8.78256000000002 | 78.4380833316797 | 750 | 0.284317245877560 |
| 8.80824000000002 | 78.6672067840742 | 750 | 0.284316332065104 |
| 8.83392000000002 | 78.8963289089449 | 750 | 0.284315418253253 |
| 8.85960000000002 | 79.1254497077699 | 750 | 0.284314504447907 |
| 8.88528000000002 | 79.3545691790741 | 750 | 0.284313590643146 |
| 8.91096000000002 | 79.5836873198944 | 750 | 0.284312676827213 |
| 8.93664000000002 | 79.8128041361472 | 750 | 0.284311763023616 |
| 8.96232000000002 | 80.0419196248757 | 750 | 0.284310849220592 |
| 8.98800000000002 | 80.2710337846007 | 750 | 0.284309935412322 |
| 9.01368000000002 | 80.5001466168037 | 750 | 0.284309021604669 |
| 9.03936000000002 | 80.7292581229578 | 750 | 0.284308107803392 |
| 9.06504000000002 | 80.9583683001074 | 750 | 0.284307193996918 |
| 9.09072000000002 | 81.1874771497315 | 750 | 0.284306280191047 |
| 9.11640000000002 | 81.4165846733125 | 750 | 0.284305366391528 |
| 9.14208000000002 | 81.6456908693648 | 750 | 0.284304452592569 |
| 9.16776000000002 | 81.8747957349316 | 750 | 0.284303538782768 |
| 9.19344000000002 | 82.1038992744518 | 750 | 0.284302624979290 |
| 9.21912000000002 | 82.3330014879258 | 750 | 0.284301711182091 |
| 9.24480000000002 | 82.5621023738763 | 750 | 0.284300797385474 |
| 9.27048000000002 | 82.7912019293364 | 750 | 0.284299883578124 |
| 9.29616000000002 | 83.0203001587497 | 750 | 0.284298969777036 |
| 9.32184000000002 | 83.2493970621165 | 750 | 0.284298055982164 |
| 9.34752000000002 | 83.4784926379562 | 750 | 0.284297142187863 |
| 9.37320000000002 | 83.7075868862715 | 750 | 0.284296228394143 |
| 9.39888000000002 | 83.9366798070562 | 750 | 0.284295314600981 |
| 9.42456000000002 | 84.1657713958696 | 750 | 0.284294400791720 |
| 9.45024000000002 | 84.3948616630796 | 750 | 0.284293487005284 |
| 9.47592000000002 | 84.6239505983202 | 750 | 0.284292573202818 |
| 9.50160000000002 | 84.8530382104739 | 750 | 0.284291659417506 |
| 9.52728000000002 | 85.0821244906540 | 750 | 0.284290745616239 |
| 9.55296000000002 | 85.3112094447867 | 750 | 0.284289831821083 |
| 9.57864000000002 | 85.5402930728722 | 750 | 0.284288918031994 |
| 9.60432000000002 | 85.7693753719494 | 750 | 0.284288004238029 |
| 9.63000000000002 | 85.9984563449763 | 750 | 0.284287090450092 |
| 9.65568000000002 | 86.2275359889912 | 750 | 0.284286176657296 |
| 9.68136000000002 | 86.4566143054761 | 750 | 0.284285262865095 |
| 9.70704000000002 | 86.6856912944338 | 750 | 0.284284349073502 |
| 9.73272000000002 | 86.9147669573407 | 750 | 0.284283435287879 |
| 9.75840000000002 | 87.1438412912344 | 750 | 0.284282521497452 |
| 9.78408000000002 | 87.3729142975973 | 750 | 0.284281607707619 |
| 9.80976000000002 | 87.6019859779122 | 750 | 0.284280693923727 |
| 9.83544000000002 | 87.8310563292158 | 750 | 0.284279780135081 |
| 9.86112000000002 | 88.0601253544682 | 750 | 0.284278866352336 |
| 9.88680000000002 | 88.2891930507057 | 750 | 0.284277952564853 |
| 9.91248000000002 | 88.5182594208949 | 750 | 0.284277038783255 |
| 9.93816000000002 | 88.7473244620685 | 750 | 0.284276124996946 |
| 9.96384000000002 | 88.9763881757127 | 750 | 0.284275211211240 |
| 9.98952000000003 | 89.2054505618213 | 750 | 0.284274297426116 |
| 10.0152000000000 | 89.4345116203972 | 750 | 0.284273383641585 |
| 10.0408800000000 | 89.6635713529241 | 750 | 0.284272469862870 |
| 10.0665600000000 | 89.8926297579211 | 750 | 0.284271556084732 |
| 10.0922400000000 | 90.1216868338974 | 750 | 0.284270642301949 |
| 10.1179200000000 | 90.3507425823401 | 750 | 0.284269728519757 |
| 10.1436000000000 | 90.5797970032488 | 750 | 0.284268814738156 |
| 10.1692800000000 | 90.8088500981082 | 750 | 0.284267900962306 |
| 10.1949600000000 | 91.0379018639515 | 750 | 0.284266987181882 |
| 10.2206400000000 | 91.2669523022573 | 750 | 0.284266073402038 |
| 10.2463200000000 | 91.4960014145133 | 750 | 0.284265159627907 |
| 10.2720000000000 | 91.7250491977493 | 750 | 0.284264245849228 |
| 10.2976800000000 | 91.9540956549353 | 750 | 0.284263332076238 |
| 10.3233600000000 | 92.1831407831036 | 750 | 0.284262418298734 |
| 10.3490400000000 | 92.4121845852187 | 750 | 0.284261504526883 |
| 10.3747200000000 | 92.6412270583124 | 750 | 0.284260590750533 |
| 10.4004000000000 | 92.8702682038699 | 750 | 0.284259676974770 |
| 10.4260800000000 | 93.0993080218939 | 750 | 0.284258763199604 |
| 10.4517600000000 | 93.3283465138641 | 750 | 0.284257849430041 |
| 10.4774400000000 | 93.5573836768116 | 750 | 0.284256935656026 |
| 10.5031200000000 | 93.7864195107358 | 750 | 0.284256021877595 |
| 10.5288000000000 | 94.0154540215811 | 750 | 0.284255108114745 |
| 10.5544800000000 | 94.2444872019194 | 750 | 0.284254194342486 |
| 10.5801600000000 | 94.4735190547168 | 750 | 0.284253280570801 |
| 10.6058400000000 | 94.7025495814626 | 750 | 0.284252366804657 |
| 10.6315200000000 | 94.9315787791836 | 750 | 0.284251453034130 |
| 10.6572000000000 | 95.1606066493659 | 750 | 0.284250539264187 |
| 10.6828800000000 | 95.3896331920123 | 750 | 0.284249625494838 |
| 10.7085600000000 | 95.6186584100906 | 750 | 0.284248711735880 |
| 10.7342400000000 | 95.8476822961686 | 750 | 0.284247797962767 |
| 10.7599200000000 | 96.0767048591683 | 750 | 0.284246884204918 |
| 10.7856000000000 | 96.3057260901696 | 750 | 0.284245970432991 |
| 10.8112800000000 | 96.5347459951150 | 750 | 0.284245056666523 |
| 10.8369600000000 | 96.7637645725199 | 750 | 0.284244142900636 |
| 10.8626400000000 | 96.9927818238718 | 750 | 0.284243229140172 |
| 10.8883200000000 | 97.2217977461952 | 750 | 0.284242315375437 |
| 10.9140000000000 | 97.4508123409804 | 750 | 0.284241401611291 |
| 10.9396800000000 | 97.6798256097091 | 750 | 0.284240487852525 |
| 10.9653600000000 | 97.9088375494083 | 750 | 0.284239574089519 |
| 10.9910400000000 | 98.1378481615654 | 750 | 0.284238660327093 |
| 11.0167200000000 | 98.3668574476685 | 750 | 0.284237746570022 |
| 11.0424000000000 | 98.5958654047440 | 750 | 0.284236832808753 |
| 11.0680800000000 | 98.8248720357621 | 750 | 0.284235919052806 |
| 11.0937600000000 | 99.0538773362604 | 750 | 0.284235005287930 |
| 11.1194400000000 | 99.2828813136808 | 750 | 0.284234091537851 |
| 11.1451200000000 | 99.5118839605837 | 750 | 0.284233177778873 |
| 11.1708000000000 | 99.7408852799398 | 750 | 0.284232264020462 |
| 11.1964800000000 | 99.9698852717517 | 750 | 0.284231350262628 |
| 11.2221600000000 | 100.198883937508 | 750 | 0.284230436510061 |
| 11.2478400000000 | 100.427881275720 | 750 | 0.284229522758049 |
| 11.2735200000000 | 100.656877284901 | 750 | 0.284228609001932 |
| 11.2992000000000 | 100.885871966534 | 750 | 0.284227695246381 |
| 11.3248800000000 | 101.114865319132 | 750 | 0.284226781486756 |
| 11.3505600000000 | 101.343857345673 | 750 | 0.284225867732365 |
| 11.3762400000000 | 101.572848046158 | 750 | 0.284224953983176 |
| 11.4019200000000 | 101.801837419099 | 750 | 0.284224040234551 |
| 11.4276000000000 | 102.030825463001 | 750 | 0.284223126481861 |
| 11.4532800000000 | 102.259812179357 | 750 | 0.284222212729744 |
| 11.4789600000000 | 102.488797568165 | 750 | 0.284221298978200 |
| 11.5046400000000 | 102.717781632408 | 750 | 0.284220385236395 |
| 11.5303200000000 | 102.946764366121 | 750 | 0.284219471485975 |
| 11.5560000000000 | 103.175745772285 | 750 | 0.284218557736128 |
| 11.5816800000000 | 103.404725852392 | 750 | 0.284217643991400 |
| 11.6073600000000 | 103.633704603460 | 750 | 0.284216730242685 |
| 11.6330400000000 | 103.862682025491 | 750 | 0.284215816490020 |
| 11.6587200000000 | 104.091658124441 | 750 | 0.284214902751494 |
| 11.6844000000000 | 104.320632892861 | 750 | 0.284213989004480 |
| 11.7100800000000 | 104.549606333730 | 750 | 0.284213075258036 |
| 11.7357600000000 | 104.778578447050 | 750 | 0.284212161512160 |
| 11.7614400000000 | 105.007549234313 | 750 | 0.284211247771343 |
| 11.7871200000000 | 105.236518692531 | 750 | 0.284210334026595 |
| 11.8128000000000 | 105.465486823198 | 750 | 0.284209420282415 |
| 11.8384799999999 | 105.694453627805 | 750 | 0.284208506543254 |
| 11.8641599999999 | 105.923419103372 | 750 | 0.284207592800208 |
| 11.8898399999999 | 106.152383251384 | 750 | 0.284206679057720 |
| 11.9155199999999 | 106.381346073334 | 750 | 0.284205765320223 |
| 11.9411999999999 | 106.610307567732 | 750 | 0.284204851583273 |
| 11.9668799999999 | 106.839267731595 | 750 | 0.284203937838059 |
| 11.9925599999999 | 107.068226570890 | 750 | 0.284203024102230 |
| 12.0182399999999 | 107.297184081137 | 750 | 0.284202110362551 |
| 12.0439199999999 | 107.526140265321 | 750 | 0.284201196627816 |
| 12.0695999999999 | 107.755095120460 | 750 | 0.284200282889257 |
| 12.0952799999999 | 107.984048648043 | 750 | 0.284199369151261 |
| 12.1209599999999 | 108.213000849567 | 750 | 0.284198455418189 |
| 12.1466399999999 | 108.441951722041 | 750 | 0.284197541681310 |
| 12.1723199999999 | 108.670901266959 | 750 | 0.284196627944994 |
| 12.1979999999999 | 108.899849484321 | 750 | 0.284195714209241 |
| 12.2236799999999 | 109.128796377114 | 750 | 0.284194800482688 |
| 12.2493599999999 | 109.357741937871 | 750 | 0.284193886743733 |
| 12.2750399999999 | 109.586686172564 | 750 | 0.284192973009654 |
| 12.3007199999999 | 109.815629079698 | 750 | 0.284192059276135 |
| 12.3263999999999 | 110.044570660768 | 750 | 0.284191145547457 |
| 12.3520799999999 | 110.273510912791 | 750 | 0.284190231815058 |
| 12.3777599999999 | 110.502449837252 | 750 | 0.284189318083209 |
| 12.4034399999999 | 110.731387435647 | 750 | 0.284188404356174 |
| 12.4291199999999 | 110.960323704990 | 750 | 0.284187490625434 |
| 12.4547999999999 | 111.189258648267 | 750 | 0.284186576899489 |
| 12.4804799999999 | 111.418192261001 | 750 | 0.284185663165636 |
| 12.5061599999999 | 111.647124549158 | 750 | 0.284184749440788 |
| 12.5318399999999 | 111.876055508262 | 750 | 0.284183835712269 |
| 12.5575199999999 | 112.104985142792 | 750 | 0.284182921992713 |
| 12.5831999999999 | 112.333913445283 | 750 | 0.284182008261102 |
| 12.6088799999999 | 112.562840421704 | 750 | 0.284181094534242 |
| 12.6345599999999 | 112.791766072056 | 750 | 0.284180180812115 |
| 12.6602399999999 | 113.020690393352 | 750 | 0.284179267086356 |
| 12.6859199999999 | 113.249613388580 | 750 | 0.284178353365314 |
| 12.7115999999999 | 113.478535054753 | 750 | 0.284177439640663 |
| 12.7372799999999 | 113.707455393361 | 750 | 0.284176525916557 |
| 12.7629599999999 | 113.936374405899 | 750 | 0.284175612197141 |
| 12.7886399999999 | 114.165292089379 | 750 | 0.284174698474132 |
| 12.8143199999999 | 114.394208445294 | 750 | 0.284173784751675 |
| 12.8399999999999 | 114.623123473648 | 750 | 0.284172871029778 |
| 12.8656799999999 | 114.852037177421 | 750 | 0.284171957316634 |
| 12.8913599999999 | 115.080949550641 | 750 | 0.284171043595814 |
| 12.9170399999999 | 115.309860596296 | 750 | 0.284170129875545 |
| 12.9427199999999 | 115.538770312892 | 750 | 0.284169216151752 |
| 12.9683999999999 | 115.767678703413 | 750 | 0.284168302432590 |
| 12.9940799999999 | 115.996585767861 | 750 | 0.284167388718042 |
| 13.0197599999999 | 116.225491503248 | 750 | 0.284166474999968 |
| 13.0454399999999 | 116.454395911066 | 750 | 0.284165561282443 |
| 13.0711199999999 | 116.683298994309 | 750 | 0.284164647573558 |
| 13.0967999999999 | 116.912200746991 | 750 | 0.284163733857112 |
| 13.1224799999999 | 117.141101173598 | 750 | 0.284162820145240 |
| 13.1481599999999 | 117.370000269647 | 750 | 0.284161906425860 |
| 13.1738399999999 | 117.598898041115 | 750 | 0.284160992715064 |
| 13.1995199999999 | 117.827794483521 | 750 | 0.284160079000802 |
| 13.2251999999999 | 118.056689598354 | 750 | 0.284159165287076 |
| 13.2508799999999 | 118.285583385615 | 750 | 0.284158251573896 |
| 13.2765599999999 | 118.514475846801 | 750 | 0.284157337865240 |
| 13.3022399999999 | 118.743366978922 | 750 | 0.284156424153146 |
| 13.3279199999999 | 118.972256784963 | 750 | 0.284155510445554 |
| 13.3535999999999 | 119.201145263432 | 750 | 0.284154596738490 |
| 13.3792799999999 | 119.430032412832 | 750 | 0.284153683028001 |
| 13.4049599999999 | 119.658918234659 | 750 | 0.284152769318054 |
| 13.4306399999999 | 119.887802728915 | 750 | 0.284151855608655 |
| 13.4563199999999 | 120.116685894098 | 750 | 0.284150941895861 |
| 13.4819999999999 | 120.345567733201 | 750 | 0.284150028187543 |
| 13.5076799999999 | 120.574448246226 | 750 | 0.284149114483678 |
| 13.5333599999999 | 120.803327433170 | 750 | 0.284148200784246 |
| 13.5590399999999 | 121.032205289550 | 750 | 0.284147287077531 |
| 13.5847199999999 | 121.261081818351 | 750 | 0.284146373371346 |
| 13.6103999999999 | 121.489957019574 | 750 | 0.284145459665700 |
| 13.6360799999999 | 121.718830894716 | 750 | 0.284144545964468 |
| 13.6617599999999 | 121.947703440788 | 750 | 0.284143632259896 |
| 13.6874399999999 | 122.176574660774 | 750 | 0.284142718559716 |
| 13.7131199999999 | 122.405444550189 | 750 | 0.284141804852344 |
| 13.7387999999999 | 122.634313115018 | 750 | 0.284140891153220 |
| 13.7644799999999 | 122.863180350771 | 750 | 0.284139977450775 |
| 13.7901599999999 | 123.092046261940 | 750 | 0.284139063756544 |
| 13.8158399999999 | 123.320910841036 | 750 | 0.284138150051323 |
| 13.8415199999999 | 123.549774094047 | 750 | 0.284137236350472 |
| 13.8671999999999 | 123.778636019477 | 750 | 0.284136322650153 |
| 13.8928799999999 | 124.007496617325 | 750 | 0.284135408950366 |
| 13.9185599999999 | 124.236355889091 | 750 | 0.284134495254921 |
| 13.9442399999999 | 124.465213833271 | 750 | 0.284133581559985 |
| 13.9699199999999 | 124.694070448372 | 750 | 0.284132667861778 |
| 13.9955999999999 | 124.922925735888 | 750 | 0.284131754164101 |
| 14.0212799999999 | 125.151779697321 | 750 | 0.284130840470736 |
| 14.0469599999999 | 125.380632328171 | 750 | 0.284129926770344 |
| 14.0726399999999 | 125.609483635928 | 750 | 0.284129013081776 |
| 14.0983199999999 | 125.838333611608 | 750 | 0.284128099382448 |
| 14.1239999999999 | 126.067182262697 | 750 | 0.284127185691155 |
| 14.1496799999999 | 126.296029586202 | 750 | 0.284126272000383 |
| 14.1753599999999 | 126.524875579122 | 750 | 0.284125358302649 |
| 14.2010399999999 | 126.753720247450 | 750 | 0.284124444612909 |
| 14.2267199999999 | 126.982563585195 | 750 | 0.284123530916239 |
| 14.2523999999999 | 127.211405599844 | 750 | 0.284122617231250 |
| 14.2780799999999 | 127.440246283912 | 750 | 0.284121703539352 |
| 14.3037599999999 | 127.669085638890 | 750 | 0.284120789844268 |
| 14.3294399999998 | 127.897923667776 | 750 | 0.284119876153416 |
| 14.3551199999998 | 128.126760372066 | 750 | 0.284118962470462 |
| 14.3807999999998 | 128.355595745774 | 750 | 0.284118048780650 |
| 14.4064799999998 | 128.584429791887 | 750 | 0.284117135091350 |
| 14.4321599999998 | 128.813262510408 | 750 | 0.284116221402573 |
| 14.4578399999998 | 129.042093902835 | 750 | 0.284115307717978 |
| 14.4835199999998 | 129.270923966170 | 750 | 0.284114394030235 |
| 14.5091999999998 | 129.499752703414 | 750 | 0.284113480346668 |
| 14.5348799999998 | 129.728580110064 | 750 | 0.284112566656313 |
| 14.5605599999998 | 129.957406192116 | 750 | 0.284111652973763 |
| 14.5862399999998 | 130.186230945075 | 750 | 0.284110739288088 |
| 14.6119199999998 | 130.415054370439 | 750 | 0.284109825602929 |
| 14.6375999999998 | 130.643876468209 | 750 | 0.284108911918295 |
| 14.6632799999998 | 130.872697238380 | 750 | 0.284107998234170 |
| 14.6889599999998 | 131.101516682452 | 750 | 0.284107084554166 |
| 14.7146399999998 | 131.330334800425 | 750 | 0.284106170878265 |
| 14.7403199999998 | 131.559151586308 | 750 | 0.284105257192079 |
| 14.7659999999998 | 131.787967046087 | 750 | 0.284104343510000 |
| 14.7916799999998 | 132.016781178267 | 750 | 0.284103429828433 |
| 14.8173599999998 | 132.245593985845 | 750 | 0.284102516154531 |
| 14.8430399999998 | 132.474405461326 | 750 | 0.284101602470408 |
| 14.8687199999998 | 132.703215610708 | 750 | 0.284100688790379 |
| 14.8943999999998 | 132.932024433985 | 750 | 0.284099775114411 |
| 14.9200799999998 | 133.160831928161 | 750 | 0.284098861435391 |
| 14.9457599999998 | 133.389638096233 | 750 | 0.284097947760425 |
| 14.9714399999998 | 133.618442933704 | 750 | 0.284097034078877 |
| 14.9971199999998 | 133.847246445074 | 750 | 0.284096120401391 |
| 15.0227999999998 | 134.076048630336 | 750 | 0.284095206727933 |
| 15.0484799999998 | 134.304849486493 | 750 | 0.284094293051449 |
| 15.0741599999998 | 134.533649015046 | 750 | 0.284093379375471 |
| 15.0998399999998 | 134.762447218994 | 750 | 0.284092465707027 |
| 15.1255199999998 | 134.991244092334 | 750 | 0.284091552032049 |
| 15.1511999999998 | 135.220039636567 | 750 | 0.284090638354078 |
| 15.1768799999998 | 135.448833856192 | 750 | 0.284089724683608 |
| 15.2025599999998 | 135.677626746709 | 750 | 0.284088811010143 |
| 15.2282399999998 | 135.906418309621 | 750 | 0.284087897337189 |
| 15.2539199999998 | 136.135208546419 | 750 | 0.284086983668205 |
| 15.2795999999998 | 136.363997454108 | 750 | 0.284086069996241 |
| 15.3052799999998 | 136.592785035686 | 750 | 0.284085156328243 |
| 15.3309599999998 | 136.821571288154 | 750 | 0.284084242657276 |
| 15.3566399999998 | 137.050356213012 | 750 | 0.284083328986816 |
| 15.3823199999998 | 137.279139811755 | 750 | 0.284082415320295 |
| 15.4079999999998 | 137.507922081386 | 750 | 0.284081501650820 |
| 15.4336799999998 | 137.736703023402 | 750 | 0.284080587981843 |
| 15.4593599999998 | 137.965482637808 | 750 | 0.284079674313371 |
| 15.4850399999998 | 138.194260924595 | 750 | 0.284078760645388 |
| 15.5107199999998 | 138.423037883768 | 750 | 0.284077846977902 |
| 15.5363999999998 | 138.651813518324 | 750 | 0.284076933317738 |
| 15.5620799999998 | 138.880587823764 | 750 | 0.284076019654638 |
| 15.5877599999998 | 139.109360800090 | 750 | 0.284075105988627 |
| 15.6134399999998 | 139.338132447294 | 750 | 0.284074192319705 |
| 15.6391199999998 | 139.566902768380 | 750 | 0.284073278654678 |
| 15.6647999999998 | 139.795671763347 | 750 | 0.284072364993530 |
| 15.6904799999998 | 140.024439429197 | 750 | 0.284071451329488 |
| 15.7161599999998 | 140.253205767421 | 750 | 0.284070537665924 |
| 15.7418399999998 | 140.481970779530 | 750 | 0.284069624006234 |
| 15.7675199999998 | 140.710734462516 | 750 | 0.284068710343658 |
| 15.7931999999998 | 140.939496819376 | 750 | 0.284067796684916 |
| 15.8188799999998 | 141.168257847117 | 750 | 0.284066883023311 |
| 15.8445599999998 | 141.397017545728 | 750 | 0.284065969358833 |
| 15.8702399999998 | 141.625775919722 | 750 | 0.284065055701551 |
| 15.8959199999998 | 141.854532966091 | 750 | 0.284064142044745 |
| 15.9215999999998 | 142.083288683328 | 750 | 0.284063228385068 |
| 15.9472799999998 | 142.312043074445 | 750 | 0.284062314729217 |
| 15.9729599999998 | 142.540796134928 | 750 | 0.284061401067182 |
| 15.9986399999998 | 142.769547869289 | 750 | 0.284060487408971 |
| 16.0243199999998 | 142.998298279025 | 750 | 0.284059573757865 |
| 16.0499999999998 | 143.227047358123 | 750 | 0.284058660100596 |
| 16.0756799999998 | 143.455795109598 | 750 | 0.284057746443820 |
| 16.1013599999998 | 143.684541533436 | 750 | 0.284056832787512 |
| 16.1270399999998 | 143.913286631150 | 750 | 0.284055919134989 |
| 16.1527199999998 | 144.142030398230 | 750 | 0.284055005476363 |
| 16.1783999999998 | 144.370772840675 | 750 | 0.284054091824773 |
| 16.2040799999998 | 144.599513953990 | 750 | 0.284053178170389 |
| 16.2297599999998 | 144.828253739666 | 750 | 0.284052264516469 |
| 16.2554399999998 | 145.056992197713 | 750 | 0.284051350863038 |
| 16.2811199999998 | 145.285729328125 | 750 | 0.284050437210084 |
| 16.3067999999998 | 145.514465132396 | 750 | 0.284049523560846 |
| 16.3324799999998 | 145.743199607536 | 750 | 0.284048609908835 |
| 16.3581599999998 | 145.971932755037 | 750 | 0.284047696257298 |
| 16.3838399999998 | 146.200664576396 | 750 | 0.284046782609460 |
| 16.4095199999998 | 146.429395068621 | 750 | 0.284045868958863 |
| 16.4351999999998 | 146.658124234702 | 750 | 0.284044955311954 |
| 16.4608799999998 | 146.886852071646 | 750 | 0.284044041662293 |
| 16.4865599999998 | 147.115578582452 | 750 | 0.284043128016323 |
| 16.5122399999998 | 147.344303764108 | 750 | 0.284042214367584 |
| 16.5379199999998 | 147.573027618127 | 750 | 0.284041300719326 |
| 16.5635999999998 | 147.801750142994 | 750 | 0.284040387068317 |
| 16.5892799999998 | 148.030471343228 | 750 | 0.284039473424199 |
| 16.6149599999998 | 148.259191215817 | 750 | 0.284038559780537 |
| 16.6406399999998 | 148.487909759252 | 750 | 0.284037646134127 |
| 16.6663199999998 | 148.716626976549 | 750 | 0.284036732491380 |
| 16.6919999999998 | 148.945342863189 | 750 | 0.284035818842713 |
| 16.7176799999998 | 149.174057423689 | 750 | 0.284034905197706 |
| 16.7433599999998 | 149.402770658042 | 750 | 0.284033991556335 |
| 16.7690399999997 | 149.631482564740 | 750 | 0.284033077915402 |
| 16.7947199999998 | 149.860193142292 | 750 | 0.284032164271771 |
| 16.8203999999997 | 150.088902390684 | 750 | 0.284031250625430 |
| 16.8460799999997 | 150.317610314435 | 750 | 0.284030336985875 |
| 16.8717599999997 | 150.546316909031 | 750 | 0.284029423343621 |
| 16.8974399999997 | 150.775022177470 | 750 | 0.284028509704956 |
| 16.9231199999997 | 151.003726116759 | 750 | 0.284027596063612 |
| 16.9487999999997 | 151.232428728387 | 750 | 0.284026682422711 |
| 16.9744799999997 | 151.461130012364 | 750 | 0.284025768782278 |
| 17.0001599999997 | 151.689829968685 | 750 | 0.284024855142300 |
| 17.0258399999997 | 151.918528598846 | 750 | 0.284023941505882 |
| 17.0515199999997 | 152.147225899851 | 750 | 0.284023027866805 |
| 17.0771999999997 | 152.375921874701 | 750 | 0.284022114231291 |
| 17.1028799999997 | 152.604616520381 | 750 | 0.284021200593100 |
| 17.1285599999997 | 152.833309838407 | 750 | 0.284020286955372 |
| 17.1542399999997 | 153.062001830269 | 750 | 0.284019373321178 |
| 17.1799199999997 | 153.290692492971 | 750 | 0.284018459684343 |
| 17.2055999999997 | 153.519381828009 | 750 | 0.284017546047956 |
| 17.2312799999997 | 153.748069836881 | 750 | 0.284016632415088 |
| 17.2569599999997 | 153.976756515088 | 750 | 0.284015718776510 |
| 17.2826399999997 | 154.205441868629 | 750 | 0.284014805144524 |
| 17.3083199999997 | 154.434125893006 | 750 | 0.284013891509916 |
| 17.3339999999997 | 154.662808591220 | 750 | 0.284012977878817 |
| 17.3596799999997 | 154.891489958752 | 750 | 0.284012064242017 |
| 17.3853599999997 | 155.120170001627 | 750 | 0.284011150611793 |
| 17.4110399999997 | 155.348848715324 | 750 | 0.284010236978938 |
| 17.4367199999997 | 155.577526101355 | 750 | 0.284009323346534 |
| 17.4623999999997 | 155.806202159714 | 750 | 0.284008409714571 |
| 17.4880799999997 | 156.034876891898 | 750 | 0.284007496086072 |
| 17.5137599999997 | 156.263550296415 | 750 | 0.284006582458014 |
| 17.5394399999997 | 156.492222371747 | 750 | 0.284005668827342 |
| 17.5651199999997 | 156.720893117907 | 750 | 0.284004755194093 |
| 17.5907999999997 | 156.949562539397 | 750 | 0.284003841567330 |
| 17.6164799999997 | 157.178230630197 | 750 | 0.284002927934946 |
| 17.6421599999997 | 157.406897397836 | 750 | 0.284002014312054 |
| 17.6678399999997 | 157.635562836286 | 750 | 0.284001100686557 |
| 17.6935199999997 | 157.864226944054 | 750 | 0.284000187055487 |
| 17.7191999999997 | 158.092889727149 | 750 | 0.283999273430855 |
| 17.7448799999997 | 158.321551181053 | 750 | 0.283998359803637 |
| 17.7705599999997 | 158.550211307280 | 750 | 0.283997446176860 |
| 17.7962399999997 | 158.778870107327 | 750 | 0.283996532553498 |
| 17.8219199999997 | 159.007527578179 | 750 | 0.283995618927559 |
| 17.8475999999997 | 159.236183721352 | 750 | 0.283994705302059 |
| 17.8732799999997 | 159.464838535328 | 750 | 0.283993791673996 |
| 17.8989599999997 | 159.693492024631 | 750 | 0.283992878052320 |
| 17.9246399999997 | 159.922144186246 | 750 | 0.283991964431058 |
| 17.9503199999997 | 160.150795017156 | 750 | 0.283991050804274 |
| 17.9759999999997 | 160.379444521887 | 750 | 0.283990137180890 |
| 18.0016799999997 | 160.608092700424 | 750 | 0.283989223560868 |
| 18.0273599999997 | 160.836739549771 | 750 | 0.283988309938319 |
| 18.0530399999997 | 161.065385072929 | 750 | 0.283987396319135 |
| 18.0787199999997 | 161.294029266882 | 750 | 0.283986482697407 |
| 18.1043999999997 | 161.522672133146 | 750 | 0.283985569076107 |
| 18.1300799999997 | 161.751313671707 | 750 | 0.283984655455211 |
| 18.1557599999997 | 161.979953884082 | 750 | 0.283983741837671 |
| 18.1814399999997 | 162.208592767254 | 750 | 0.283982828217612 |
| 18.2071199999997 | 162.437230322720 | 750 | 0.283981914597954 |
| 18.2327999999997 | 162.665866551997 | 750 | 0.283981000981636 |
| 18.2584799999997 | 162.894501452062 | 750 | 0.283980087362796 |
| 18.2841599999997 | 163.123135022926 | 750 | 0.283979173741470 |
| 18.3098399999997 | 163.351767269096 | 750 | 0.283978260126370 |
| 18.3355199999997 | 163.580398187555 | 750 | 0.283977346511658 |
| 18.3611999999997 | 163.809027776810 | 750 | 0.283976432894460 |
| 18.3868799999997 | 164.037656038353 | 750 | 0.283975519277657 |
| 18.4125599999997 | 164.266282972194 | 750 | 0.283974605661270 |
| 18.4382399999997 | 164.494908579831 | 750 | 0.283973692048172 |
| 18.4639199999997 | 164.723532856743 | 750 | 0.283972778429696 |
| 18.4895999999997 | 164.952155808960 | 750 | 0.283971864817395 |
| 18.5152799999997 | 165.180777431960 | 750 | 0.283970951202615 |
| 18.5409599999997 | 165.409397727241 | 750 | 0.283970037588222 |
| 18.5666399999997 | 165.638016696320 | 750 | 0.283969123977106 |
| 18.5923199999997 | 165.866634337677 | 750 | 0.283968210366368 |
| 18.6179999999997 | 166.095250648314 | 750 | 0.283967296750316 |
| 18.6436799999997 | 166.323865631233 | 750 | 0.283966383134667 |
| 18.6693599999997 | 166.552479290944 | 750 | 0.283965469527959 |
| 18.6950399999997 | 166.781091619931 | 750 | 0.283964555915949 |
| 18.7207199999997 | 167.009702621191 | 750 | 0.283963642304320 |
| 18.7463999999997 | 167.238312294736 | 750 | 0.283962728693094 |
| 18.7720799999997 | 167.466920640558 | 750 | 0.283961815082259 |
| 18.7977599999997 | 167.695527660156 | 750 | 0.283960901474633 |
| 18.8234399999997 | 167.924133350528 | 750 | 0.283959987864572 |
| 18.8491199999997 | 168.152737713169 | 750 | 0.283959074254887 |
| 18.8747999999997 | 168.381340748089 | 750 | 0.283958160645600 |
| 18.9004799999997 | 168.609942456785 | 750 | 0.283957247039514 |
| 18.9261599999997 | 168.838542836240 | 750 | 0.283956333430982 |
| 18.9518399999997 | 169.067141889476 | 750 | 0.283955419825652 |
| 18.9775199999997 | 169.295739613469 | 750 | 0.283954506217881 |
| 19.0031999999997 | 169.524336009734 | 750 | 0.283953592610503 |
| 19.0288799999997 | 169.752931076760 | 750 | 0.283952679000707 |
| 19.0545599999997 | 169.981524819055 | 750 | 0.283951765396873 |
| 19.0802399999997 | 170.210117232114 | 750 | 0.283950851790631 |
| 19.1059199999997 | 170.438708318935 | 750 | 0.283949938187540 |
| 19.1315999999997 | 170.667298075011 | 750 | 0.283949024579264 |
| 19.1572799999997 | 170.895886506359 | 750 | 0.283948110976926 |
| 19.1829599999997 | 171.124473609959 | 750 | 0.283947197374944 |
| 19.2086399999997 | 171.353059382811 | 750 | 0.283946283767795 |
| 19.2343199999996 | 171.581643829425 | 750 | 0.283945370163792 |
| 19.2599999999996 | 171.810226951299 | 750 | 0.283944456565676 |
| 19.2856799999996 | 172.038808742422 | 750 | 0.283943542962404 |
| 19.3113599999996 | 172.267389205791 | 750 | 0.283942629359488 |
| 19.3370399999996 | 172.495968341417 | 750 | 0.283941715756951 |
| 19.3627199999996 | 172.724546150799 | 750 | 0.283940802157528 |
| 19.3883999999996 | 172.953122629411 | 750 | 0.283939888552959 |
| 19.4140799999996 | 173.181697781783 | 750 | 0.283938974951514 |
| 19.4397599999996 | 173.410271606395 | 750 | 0.283938061350419 |
| 19.4654399999996 | 173.638844104764 | 750 | 0.283937147752431 |
| 19.4911199999996 | 173.867415273871 | 750 | 0.283936234152064 |
| 19.5167999999996 | 174.095985115214 | 750 | 0.283935320552044 |
| 19.5424799999996 | 174.324553628803 | 750 | 0.283934406952393 |
| 19.5681599999996 | 174.553120814627 | 750 | 0.283933493353086 |
| 19.5938399999996 | 174.781686674201 | 750 | 0.283932579756862 |
| 19.6195199999996 | 175.010251204507 | 750 | 0.283931666158271 |
| 19.6451999999996 | 175.238814407042 | 750 | 0.283930752560022 |
| 19.6708799999996 | 175.467376281817 | 750 | 0.283929838962134 |
| 19.6965599999996 | 175.695936830326 | 750 | 0.283928925367289 |
| 19.7222399999996 | 175.924496049567 | 750 | 0.283928011770096 |
| 19.7479199999996 | 176.153053941038 | 750 | 0.283927098173251 |
| 19.7735999999996 | 176.381610506238 | 750 | 0.283926184579434 |
| 19.7992799999996 | 176.610165742166 | 750 | 0.283925270983277 |
| 19.8249599999996 | 176.838719650315 | 750 | 0.283924357387452 |
| 19.8506399999996 | 177.067272230694 | 750 | 0.283923443791981 |
| 19.8763199999996 | 177.295823484804 | 750 | 0.283922530199530 |
| 19.9019999999996 | 177.524373409624 | 750 | 0.283921616604726 |
| 19.9276799999996 | 177.752922006670 | 750 | 0.283920703010272 |
| 19.9533599999996 | 177.981469275936 | 750 | 0.283919789416155 |
| 19.9790399999996 | 178.210015218921 | 750 | 0.283918875825029 |
| 20.0047199999996 | 178.438559832622 | 750 | 0.283917962231580 |
| 20.0303999999996 | 178.667103118532 | 750 | 0.283917048638453 |
| 20.0560799999996 | 178.895645076662 | 750 | 0.283916135045670 |
| 20.0817599999996 | 179.124185707005 | 750 | 0.283915221453218 |
| 20.1074399999996 | 179.352725011060 | 750 | 0.283914307863734 |
| 20.1331199999996 | 179.581262985823 | 750 | 0.283913394271937 |
| 20.1587999999996 | 179.809799631282 | 750 | 0.283912480677814 |
| 20.1844799999996 | 180.038334950460 | 750 | 0.283911567086674 |
| 20.2101599999996 | 180.266868943351 | 750 | 0.283910653498493 |
| 20.2358399999996 | 180.495401606933 | 750 | 0.283909739907986 |
| 20.2615199999996 | 180.723932942723 | 750 | 0.283908826317812 |
| 20.2871999999996 | 180.952462952215 | 750 | 0.283907912730574 |
| 20.3128799999996 | 181.180991632405 | 750 | 0.283906999141037 |
| 20.3385599999996 | 181.409518984793 | 750 | 0.283906085551817 |
| 20.3642399999996 | 181.638045010879 | 750 | 0.283905171965520 |
| 20.3899199999996 | 181.866569707659 | 750 | 0.283904258376929 |
| 20.4155999999996 | 182.095093075118 | 750 | 0.283903344786031 |
| 20.4412799999996 | 182.323615116283 | 750 | 0.283902431198070 |
| 20.4669599999996 | 182.552135831146 | 750 | 0.283901517613023 |
| 20.4926399999996 | 182.780655216684 | 750 | 0.283900604025669 |
| 20.5183199999996 | 183.009173274415 | 750 | 0.283899690438634 |
| 20.5439999999996 | 183.237690004326 | 750 | 0.283898776851896 |
| 20.5696799999996 | 183.466205406427 | 750 | 0.283897863265473 |
| 20.5953599999996 | 183.694719482219 | 750 | 0.283896949681942 |
| 20.6210399999996 | 183.923232228678 | 750 | 0.283896036096113 |
| 20.6467199999996 | 184.151743647322 | 750 | 0.283895122510596 |
| 20.6723999999996 | 184.380253738145 | 750 | 0.283894208925379 |
| 20.6980799999996 | 184.608762502645 | 750 | 0.283893295343025 |
| 20.7237599999996 | 184.837269939326 | 750 | 0.283892381760972 |
| 20.7494399999996 | 185.065776045159 | 750 | 0.283891468174062 |
| 20.7751199999996 | 185.294280824677 | 750 | 0.283890554590028 |
| 20.8007999999996 | 185.522784276365 | 750 | 0.283889641006287 |
| 20.8264799999996 | 185.751286401723 | 750 | 0.283888727425385 |
| 20.8521599999996 | 185.979787197746 | 750 | 0.283887813842222 |
| 20.8778399999996 | 186.208286665928 | 750 | 0.283886900259338 |
| 20.9035199999996 | 186.436784806279 | 750 | 0.283885986676751 |
| 20.9291999999996 | 186.665281618792 | 750 | 0.283885073094449 |
| 20.9548799999996 | 186.893777104967 | 750 | 0.283884159514965 |
| 20.9805599999996 | 187.122271261798 | 750 | 0.283883245933227 |
| 21.0062399999996 | 187.350764090780 | 750 | 0.283882332351761 |
| 21.0319199999996 | 187.579255590414 | 750 | 0.283881418768050 |
| 21.0575999999996 | 187.807745765219 | 750 | 0.283880505189685 |
| 21.0832799999996 | 188.036234612168 | 750 | 0.283879591611581 |
| 21.1089599999996 | 188.264722129766 | 750 | 0.283878678031231 |
| 21.1346399999996 | 188.493208321013 | 750 | 0.283877764453667 |
| 21.1603199999996 | 188.721693181397 | 750 | 0.283876850871341 |
| 21.1859999999996 | 188.950176715434 | 750 | 0.283875937291808 |
| 21.2116799999996 | 189.178658921608 | 750 | 0.283875023712534 |
| 21.2373599999996 | 189.407139799928 | 750 | 0.283874110133537 |
| 21.2630399999996 | 189.635619351891 | 750 | 0.283873196557304 |
| 21.2887199999996 | 189.864097574489 | 750 | 0.283872282978835 |
| 21.3143999999996 | 190.092574469222 | 750 | 0.283871369400628 |
| 21.3400799999996 | 190.321050036084 | 750 | 0.283870455822674 |
| 21.3657599999996 | 190.549524276593 | 750 | 0.283869542247484 |
| 21.3914399999996 | 190.777997186215 | 750 | 0.283868628667562 |
| 21.4171199999996 | 191.006468769468 | 750 | 0.283867715090382 |
| 21.4427999999996 | 191.234939026362 | 750 | 0.283866801515953 |
| 21.4684799999996 | 191.463407953867 | 750 | 0.283865887939278 |
| 21.4941599999996 | 191.691875553501 | 750 | 0.283864974362866 |
| 21.5198399999996 | 191.920341823748 | 750 | 0.283864060784226 |
| 21.5455199999996 | 192.148806769126 | 750 | 0.283863147210779 |
| 21.5711999999996 | 192.377270385119 | 750 | 0.283862233635112 |
| 21.5968799999996 | 192.605732673221 | 750 | 0.283861320059681 |
| 21.6225599999996 | 192.834193634952 | 750 | 0.283860406486969 |
| 21.6482399999996 | 193.062653267286 | 750 | 0.283859492912030 |
| 21.6739199999995 | 193.291111573233 | 750 | 0.283858579339782 |
| 21.6995999999995 | 193.519568548277 | 750 | 0.283857665762863 |
| 21.7252799999995 | 193.748024196930 | 750 | 0.283856752188631 |
| 21.7509599999995 | 193.976478517693 | 750 | 0.283855838614644 |
| 21.7766399999995 | 194.204931510558 | 750 | 0.283854925040891 |
| 21.8023199999995 | 194.433383175518 | 750 | 0.283854011467361 |
| 21.8279999999995 | 194.661833514092 | 750 | 0.283853097896514 |
| 21.8536799999995 | 194.890282524756 | 750 | 0.283852184325879 |
| 21.8793599999995 | 195.118730202993 | 750 | 0.283851270748164 |
| 21.9050399999995 | 195.347176559360 | 750 | 0.283850357180424 |
| 21.9307199999995 | 195.575621584792 | 750 | 0.283849443608023 |
| 21.9563999999995 | 195.804065282320 | 750 | 0.283848530035854 |
| 21.9820799999995 | 196.032507651928 | 750 | 0.283847616463895 |
| 22.0077599999995 | 196.260948693627 | 750 | 0.283846702892164 |
| 22.0334399999995 | 196.489388408917 | 750 | 0.283845789323071 |
| 22.0591199999995 | 196.717826793264 | 750 | 0.283844875749341 |
| 22.0847999999995 | 196.946263851205 | 750 | 0.283843962178255 |
| 22.1104799999995 | 197.174699581221 | 750 | 0.283843048607381 |
| 22.1361599999995 | 197.403133984816 | 750 | 0.283842135039116 |
| 22.1618399999995 | 197.631567057470 | 750 | 0.283841221466250 |
| 22.1875199999995 | 197.859998805207 | 750 | 0.283840307898393 |
| 22.2131999999995 | 198.088429223509 | 750 | 0.283839394328344 |
| 22.2388799999995 | 198.316858312368 | 750 | 0.283838480756099 |
| 22.2645599999995 | 198.545286076303 | 750 | 0.283837567188842 |
| 22.2902399999995 | 198.773712510797 | 750 | 0.283836653619395 |
| 22.3159199999995 | 199.002137617344 | 750 | 0.283835740050135 |
| 22.3415999999995 | 199.230561395956 | 750 | 0.283834826481081 |
| 22.3672799999995 | 199.458983848131 | 750 | 0.283833912914604 |
| 22.3929599999995 | 199.687404969336 | 750 | 0.283832999343540 |
| 22.4186399999995 | 199.915824765616 | 750 | 0.283832085777439 |
| 22.4443199999995 | 200.144243233939 | 750 | 0.283831172211512 |
| 22.4699999999995 | 200.372660371296 | 750 | 0.283830258641027 |
| 22.4956799999995 | 200.601076182207 | 750 | 0.283829345073102 |
| 22.5213599999995 | 200.829490663644 | 750 | 0.283828431502981 |
| 22.5470399999995 | 201.057903821657 | 750 | 0.283827517940154 |
| 22.5727199999995 | 201.286315648682 | 750 | 0.283826604372759 |
| 22.5983999999995 | 201.514726146239 | 750 | 0.283825690803188 |
| 22.6240799999995 | 201.743135317339 | 750 | 0.283824777236156 |
| 22.6497599999995 | 201.971543161974 | 750 | 0.283823863671643 |
| 22.6754399999995 | 202.199949677134 | 750 | 0.283822950104952 |
| 22.7011199999995 | 202.428354864314 | 750 | 0.283822036538420 |
| 22.7267999999995 | 202.656758722015 | 750 | 0.283821122969717 |
| 22.7524799999995 | 202.885161254758 | 750 | 0.283820209405875 |
| 22.7781599999995 | 203.113562458004 | 750 | 0.283819295839838 |
| 22.8038399999995 | 203.341962334783 | 750 | 0.283818382276312 |
| 22.8295199999995 | 203.570360880558 | 750 | 0.283817468708265 |
| 22.8551999999995 | 203.798758102869 | 750 | 0.283816555147376 |
| 22.8808799999995 | 204.027153992667 | 750 | 0.283815641579644 |
| 22.9065599999995 | 204.255548555976 | 750 | 0.283814728014391 |
| 22.9322399999995 | 204.483941791297 | 750 | 0.283813814449298 |
| 22.9579199999995 | 204.712333698621 | 750 | 0.283812900884354 |
| 22.9835999999995 | 204.940724277940 | 750 | 0.283811987319549 |
| 23.0092799999995 | 205.169113530773 | 750 | 0.283811073757217 |
| 23.0349599999995 | 205.397501452575 | 750 | 0.283810160190380 |
| 23.0606399999995 | 205.625888049398 | 750 | 0.283809246628327 |
| 23.0863199999995 | 205.854273316701 | 750 | 0.283808333064097 |
| 23.1119999999995 | 206.082657254477 | 750 | 0.283807419497685 |
| 23.1376799999995 | 206.311039867265 | 750 | 0.283806505936035 |
| 23.1633599999995 | 206.539421150521 | 750 | 0.283805592372200 |
| 23.1890399999995 | 206.767801105764 | 750 | 0.283804678808505 |
| 23.2147199999995 | 206.996179732985 | 750 | 0.283803765244938 |
| 23.2403999999995 | 207.224557032176 | 750 | 0.283802851681489 |
| 23.2660799999995 | 207.452933003347 | 750 | 0.283801938118174 |
| 23.2917599999995 | 207.681307646483 | 750 | 0.283801024554971 |
| 23.3174399999995 | 207.909680961594 | 750 | 0.283800110991898 |
| 23.3431199999995 | 208.138052947160 | 750 | 0.283799197426657 |
| 23.3687999999995 | 208.366423606193 | 750 | 0.283798283863811 |
| 23.3944799999995 | 208.594792938704 | 750 | 0.283797370303370 |
| 23.4201599999995 | 208.823160941657 | 750 | 0.283796456740747 |
| 23.4458399999995 | 209.051527618081 | 750 | 0.283795543180519 |
| 23.4715199999995 | 209.279892963438 | 750 | 0.283794629615846 |
| 23.4971999999995 | 209.508256982249 | 750 | 0.283793716053545 |
| 23.5228799999995 | 209.736619673014 | 750 | 0.283792802491356 |
| 23.5485599999995 | 209.964981038739 | 750 | 0.283791888933792 |
| 23.5742399999995 | 210.193341071881 | 750 | 0.283790975369533 |
| 23.5999199999995 | 210.421699778473 | 750 | 0.283790061807637 |
| 23.6255999999995 | 210.650057155485 | 750 | 0.283789148243567 |
| 23.6512799999995 | 210.878413207459 | 750 | 0.283788234684116 |
| 23.6769599999995 | 211.106767929854 | 750 | 0.283787321122495 |
| 23.7026399999995 | 211.335121324171 | 750 | 0.283786407560953 |
| 23.7283199999995 | 211.563473390421 | 750 | 0.283785493999504 |
| 23.7539999999995 | 211.791824127077 | 750 | 0.283784580435882 |
| 23.7796799999995 | 212.020173538681 | 750 | 0.283783666876845 |
| 23.8053599999995 | 212.248521620692 | 750 | 0.283782753315638 |
| 23.8310399999995 | 212.476868374612 | 750 | 0.283781839754498 |
| 23.8567199999995 | 212.705213798939 | 750 | 0.283780926191202 |
| 23.8823999999995 | 212.933557898191 | 750 | 0.283780012632445 |
| 23.9080799999995 | 213.161900667845 | 750 | 0.283779099071528 |
| 23.9337599999995 | 213.390242109402 | 750 | 0.283778185510677 |
| 23.9594399999995 | 213.618582222854 | 750 | 0.283777271949881 |
| 23.9851199999995 | 213.846921009721 | 750 | 0.283776358391383 |
| 24.0107999999995 | 214.075258466966 | 750 | 0.283775444830706 |
| 24.0364799999995 | 214.303594596109 | 750 | 0.283774531270096 |
| 24.0621599999995 | 214.531929395631 | 750 | 0.283773617707321 |
| 24.0878399999995 | 214.760262868543 | 750 | 0.283772704146811 |
| 24.1135199999994 | 214.988595013345 | 750 | 0.283771790586360 |
| 24.1391999999994 | 215.216925831532 | 750 | 0.283770877028160 |
| 24.1648799999994 | 215.445255318581 | 750 | 0.283769963465589 |
| 24.1905599999994 | 215.673583480526 | 750 | 0.283769049907481 |
| 24.2162399999994 | 215.901910311314 | 750 | 0.283768136344989 |
| 24.2419199999994 | 216.130235815488 | 750 | 0.283767222784751 |
| 24.2675999999994 | 216.358559993032 | 750 | 0.283766309226741 |
| 24.2932799999994 | 216.586882840934 | 750 | 0.283765395666569 |
| 24.3189599999994 | 216.815204360694 | 750 | 0.283764482106427 |
| 24.3446399999994 | 217.043524552305 | 750 | 0.283763568546305 |
| 24.3703199999994 | 217.271843415776 | 750 | 0.283762654986216 |
| 24.3959999999994 | 217.500160949585 | 750 | 0.283761741423962 |
| 24.4216799999994 | 217.728477158257 | 750 | 0.283760827866097 |
| 24.4473599999994 | 217.956792037268 | 750 | 0.283759914306070 |
| 24.4730399999994 | 218.185105588113 | 750 | 0.283759000746049 |
| 24.4987199999994 | 218.413417810803 | 750 | 0.283758087186049 |
| 24.5243999999994 | 218.641728705326 | 750 | 0.283757173626060 |
| 24.5500799999994 | 218.870038271675 | 750 | 0.283756260066070 |
| 24.5757599999994 | 219.098346509858 | 750 | 0.283755346506093 |
| 24.6014399999994 | 219.326653418348 | 750 | 0.283754432943939 |
| 24.6271199999994 | 219.554959000177 | 750 | 0.283753519383967 |
| 24.6527999999994 | 219.783263253824 | 750 | 0.283752605823991 |
| 24.6784799999994 | 220.011566179280 | 750 | 0.283751692264002 |
| 24.7041599999994 | 220.239867776555 | 750 | 0.283750778704013 |
| 24.7298399999994 | 220.468168047143 | 750 | 0.283749865146165 |
| 24.7555199999994 | 220.696466988031 | 750 | 0.283748951586150 |
| 24.7811999999994 | 220.924764599209 | 750 | 0.283748038023963 |
| 24.8068799999994 | 221.153060883691 | 750 | 0.283747124463907 |
| 24.8325599999994 | 221.381355839975 | 750 | 0.283746210903838 |
| 24.8582399999994 | 221.609649469556 | 750 | 0.283745297345885 |
| 24.8839199999994 | 221.837941769420 | 750 | 0.283744383785763 |
| 24.9095999999994 | 222.066232739557 | 750 | 0.283743470223466 |
| 24.9352799999994 | 222.294522382982 | 750 | 0.283742556663276 |
| 24.9609599999994 | 222.522810698191 | 750 | 0.283741643103059 |
| 24.9866399999994 | 222.751097686680 | 750 | 0.283740729544933 |
| 25.0123199999994 | 222.979383342412 | 750 | 0.283739815980364 |
| 25.0379999999994 | 223.207667675959 | 750 | 0.283738902424295 |
| 25.0636799999994 | 223.435950676728 | 750 | 0.283737988861768 |
| 25.0893599999994 | 223.664232350777 | 750 | 0.283737075301334 |
| 25.1150399999994 | 223.892512696583 | 750 | 0.283736161740848 |
| 25.1407199999994 | 224.120791712624 | 750 | 0.283735248178175 |
| 25.1663999999994 | 224.349069403446 | 750 | 0.283734334619702 |
[truncated: 890,539 more chars]
